# Efficacy and safety of continuous positive airway pressure on blood pressure in patients with obstructive sleep apnea: an overview of systematic reviews and meta analyses

**DOI:** 10.3389/fmed.2026.1785996

**Published:** 2026-03-24

**Authors:** Jingjing Liu, Yan Cui, Yongshi Liu, Mingwei Sima, Moxuan Han, Kejia Ma, Ziyang Yu, Donghui Yue, Yan Bi

**Affiliations:** 1 College of Traditional Chinese Medicine, Changchun University of Chinese Medicine, Changchun, Jilin, China; 2 College of Basic Medicine, Changchun University of Chinese Medicine, Changchun, Jilin, China

**Keywords:** blood pressure, continuous positive airway pressure, efficacy, obstructive sleep apnea, overview of systematic reviews and meta-analyses, safety

## Abstract

**Introduction:**

Obstructive sleep apnea (OSA) is a prevalent disorder associated with significant cardiovascular morbidity, including hypertension. Continuous positive airway pressure (CPAP) is the primary treatment for OSA and has been proposed as an adjunctive therapy for hypertension. However, evidence regarding its antihypertensive effects remains heterogeneous, with many studies exhibiting low methodological quality.

**Methods:**

A comprehensive computerized search of PubMed, Embase, the Cochrane Library, Web of Science, China National Knowledge Infrastructure (CNKI), VIP, Wanfang, and the China Biology Medicine disc (CBM) databases was conducted from their inception to December 1, 2025, to systematically identify systematic reviews and meta-analyses examining the effect of continuous positive airway pressure (CPAP) on blood pressure in patients with obstructive sleep apnea (OSA). A citation overlap matrix was constructed, and the corrected covered area (CCA) was calculated to assess the degree of overlap among primary studies. The ROBIS, AMSTAR-2, PRISMA 2020, and GRADE tools were used to evaluate the risk of bias, methodological quality, reporting quality, and certainty of evidence of the included systematic reviews/meta-analyses, respectively. Quantitative and qualitative analyses were conducted on the primary outcomes to gain a more comprehensive and in-depth understanding.

**Results:**

This umbrella review included a total of 17 systematic reviews/meta-analyses. The citation matrix analysis yielded a corrected coverage area of 14.2%, indicating a substantial degree of overlap among the primary studies, which may artificially inflate the perceived consistency of findings. Methodological quality assessment using AMSTAR-2 revealed a critical limitation that fundamentally shapes the interpretation of this overview: only 3 out of 17 included reviews were rated as high quality, while the remaining 14 (82.3%) were judged to be of low quality. This widespread methodological weakness—primarily driven by lack of pre-registered protocols, inadequate integration of risk of bias into conclusions, and poor reporting of funding sources—directly undermines the reliability of the conclusions drawn by these individual reviews and, by extension, the overall findings of this overview. The GRADE assessment of evidence certainty showed that among all evaluated outcomes, only 4 were rated as high quality, 29 as moderate, 51 as low, and 25 as very low.

**Discussion:**

Current evidence suggests that continuous positive airway pressure (CPAP) therapy is associated with modest reductions in blood pressure among patients with obstructive sleep apnea, particularly in nocturnal measures. The therapy appears generally well-tolerated with no serious adverse events reported. However, these findings should be interpreted with caution due to the predominantly low-to-very-low certainty of the evidence, substantial methodological weaknesses in the included reviews, and significant overlap of primary studies. The magnitude of blood pressure reduction may be influenced by factors such as CPAP adherence and baseline hypertension severity, although these could not be robustly explored due to limitations in the included literature. Future research should employ large-scale, long-term real-world studies with standardized reporting of CPAP intervention details (e.g., device type, adherence, pressure settings) and diverse outcome measures such as ambulatory blood pressure monitoring. Such studies are needed to clarify the differential efficacy of CPAP across distinct clinical subgroups and its long-term cardiovascular benefits, thereby informing more precise clinical practice guidelines.

**Systematic review registration:**

https://www.crd.york.ac.uk/PROSPERO/; CRD420251239607.

## Introduction

Obstructive sleep apnea (OSA) is a prevalent sleep-disordered breathing condition characterized by recurrent episodes of upper airway collapse during sleep, leading to intermittent hypoxemia, sympathetic nervous system overactivation, and sleep fragmentation (1). These pathophysiological disturbances are closely linked to the development and poor control of hypertension. Cross-sectional and longitudinal studies have shown an association between OSA and hypertension (2–4). Epidemiological and mechanistic studies have consistently identified OSA as one of the most common yet modifiable causes of secondary hypertension (5–7), underscoring the importance of addressing sleep-disordered breathing as an etiological approach to blood pressure (BP) management.

From a physiological standpoint, continuous positive airway pressure (CPAP) therapy offers a compelling rationale for BP reduction in patients with OSA. By delivering a continuous stream of positive pressure via a nasal or facial mask to maintain upper airway patency (8), CPAP effectively eliminates apneic events, corrects nocturnal hypoxemia, and mitigates sympathetic nervous system activation (9–11). These effects are expected to translate into improved hemodynamic stability, restoration of endothelial function, and reduced systemic inflammation (12–15), thereby exerting beneficial effects on BP regulation, particularly during sleep. Numerous large-scale, randomized controlled trials conducted globally have consistently demonstrated that CPAP therapy is effective and well-tolerated in the treatment of moderate-to-severe OSA (16–19), significantly improving daytime sleepiness and enhancing quality of life (20–22).

However, the translation of these physiological benefits into consistent and clinically meaningful BP reductions remains a subject of ongoing debate. Over the past two decades, a substantial body of evidence, including numerous randomized controlled trials and systematic reviews with meta-analyses (SRs/MAs), has evaluated the antihypertensive efficacy of CPAP in patients with OSA (23–25). While many of these studies report statistically significant reductions in BP, the magnitude of effect varies considerably across populations, and the clinical relevance of these changes is often modest. Current evidence is characterized by limited spatiotemporal coverage and significant heterogeneity, which manifests in multiple dimensions including the geographic and baseline characteristics of study populations, the stratification of OSA severity, adherence thresholds for CPAP therapy, intervention durations, and methods of blood pressure measurement (e.g., office, ambulatory, and home monitoring). Furthermore, the existing SRs/MAs exhibit significant heterogeneity in their methodological rigor, with some studies exhibiting deficiencies in methodological execution (e.g., adherence to PRISMA guidelines) and evidence quality assessment (e.g., application of the GRADE tool). This fragmentation of evidence, coupled with the overlapping inclusion of primary studies across multiple reviews, limits the ability of clinicians and guideline developers to draw definitive conclusions regarding the true clinical benefit of CPAP for BP control and creates “blind spots” in accurately determining the antihypertensive benefits of CPAP and identifying suitable patient populations.

Therefore, a higher-level synthesis—an overview of systematic reviews—is urgently needed to systematically collate, appraise, and integrate the available evidence without duplicating summaries of original research. By employing validated assessment tools such as ROBIS, AMSTAR-2, PRISMA 2020, and GRADE, this overview aims to evaluate the risk of bias, methodological and reporting quality, and certainty of evidence across existing SRs/MAs. Through rigorous assessments of their risk of bias, methodological quality, reporting standards, and the overall quality of the evidence bodies, we aim to clarify the strengths and key limitations of the current evidence landscape. Through the integration of quantitative re-analysis and qualitative synthesis, this study aims to achieve high-level evidence integration and evaluation across regions, over extended periods, and among multiple subgroups. It seeks to advance the field’s understanding from “accumulation of evidence quantity” to “leap in evidence quality,” thereby providing a solid evidence-based decision-making foundation for the optimized management of OSA-related hypertension and informing future research priorities.

## Materials and methods

### Protocol registration

To ensure research transparency and methodological rigor, the study protocol was registered on the internationally recognized systematic review registry PROSPERO (Registration number: CRD420251239607) prior to the commencement of formal literature searches. All key procedures implemented during the research, including the formulation of search strategies, criteria for literature inclusion and exclusion, and specific methods for data analysis, were conducted in accordance with the protocol registered beforehand.

### Inclusion criteria

(1) Study Types: Systematic Reviews and/or Meta-Analyses (SRs/MAs) that primarily employed continuous positive airway pressure (CPAP) as the main intervention and primarily enrolled patients with obstructive sleep apnea (OSA) comorbid with hypertension or elevated blood pressure. (2) Intervention and Control: The treatment group received CPAP as the primary intervention (which may be combined with conventional antihypertensive therapy), while the control group received sham CPAP, standard care, blank control, placebo, or other non-CPAP treatments (e.g., nocturnal supplemental oxygen) as the primary control measures. (3) Blinding: In the original studies, participants and/or researchers and/or outcome assessors were unaware of intervention allocation (including single-blind, double-blind, or triple-blind designs). (4) Allocation method: In the original study, participants were randomly assigned to either the CPAP group or the control group.

### Exclusion criteria

(1) Duplicate publication; (2) Unavailable full text or incomplete data; (3) Review articles; (4) Irrelevant to the research topic; (5) Clinical trials; (6) Animal studies.

### Search strategy

This review systematically searched eight databases, including PubMed, Embase, the Cochrane Library, Web of Science, CNKI, VIP, Wanfang, and CBM, from their inception to December 1, 2025. The search aimed to identify all systematic reviews and meta-analyses (SRs/MAs) in which continuous positive airway pressure (CPAP) served as the primary intervention for patients with obstructive sleep apnea (OSA) who also had comorbid hypertension or elevated blood pressure. The search terms and strategy were constructed as follows (using Embase as an example): Search:

1. ‘continuous positive airway pressure’/exp
2. ‘constant positive airway pressure’:ti,ab,kw OR ‘constant positive pressure breathing’:ti,ab,kw OR ‘constant positive pressure ventilation’:ti,ab,kw OR ‘continous positive airway pressure’:ti,ab,kw OR ‘continuous positive airway pressure ventilation’:ti,ab,kw OR ‘continuous positive pressure breathing’:ti,ab,kw
3. #1 OR #2
4. ‘hypertension’/exp.
5. ‘blood pressure,high’:ti,ab,kw OR ‘high blood pressure’:ti,ab,kw OR ‘hypertensive disease’:ti,ab,kw OR ‘hypertension’:ti,ab,kw
6. #4 OR #5
7. ‘sleep apnea syndromes’/exp.
8. ‘apnea during sleep’:ti,ab,kw OR ‘apnea syndrome’:ti,ab,kw OR ‘apnea syndromes’:ti,ab,kw OR ‘apnea, sleep’:ti,ab,kw OR ‘apneas during sleep’:ti,ab,kw OR ‘apnoea, sleep’:ti,ab,kw
9. ‘obstructive sleep apnea’/exp.
10. ‘obstructive apnea’:ti,ab,kw OR ‘obstructive apnea during sleep’:ti,ab,kw OR ‘obstructive apneas during sleep’:ti,ab,kw OR ‘obstructive apnoea’:ti,ab,kw OR ‘obstructive sleep apnea hypopnea syndrome’:ti,ab,kw OR ‘obstructive sleep apnea syndrome’:ti,ab,kw OR ‘obstructive sleep apneas’:ti,ab,kw
11. #7 OR #8 OR #9 OR #10
12. ‘meta analysis’/exp.
13. ‘meta analysis ‘:ti,ab,kw OR ‘metaanalysis ‘:ti,ab,kw OR ‘pooled analysis ‘:ti,ab,kw OR ‘data synthesis ‘:ti,ab,kw
14. ‘systematic review’/exp.
15. ‘systematic review ‘:ti,ab,kw OR ‘systematic literature review ‘:ti,ab,kw OR ‘systematic meta-analysis’:ti,ab,kw
16. #12 OR #13 OR #14 OR #15
17. #3 AND #6 AND #11 AND #16

### Literature screening and data extraction

Throughout the literature screening and data extraction phases, two systematically trained researchers will independently carry out the relevant work. First, based on predetermined search strategies, the researchers will conduct comprehensive searches in the target databases. After completing the preliminary literature search, both parties cross-checked the retrieved entries to ensure the completeness and consistency of the search process. In cases of disagreement during the comparison, the two individuals will analyze the reasons for the discrepancies through joint discussion. If a consensus cannot be reached after discussion, a third researcher will be introduced to participate in the assessment. Should disagreements persist thereafter, a senior researcher with advanced academic credentials will be invited to make the final adjudication on whether the literature meets the predefined inclusion and exclusion criteria. Subsequently, the researchers will perform a systematic data extraction from the included literature. The extraction process encompasses, but is not limited to, the following key elements from the literature: the first author, year of publication, the number of primary studies included in the systematic review or meta-analysis along with the total sample size, study design type, detailed content of the intervention(s), design of the control measure(s), the quality assessment tool(s) employed, relevant outcome measures, as well as the primary conclusions or viewpoints presented. The entire process is conducted with rigor to ensure objectivity and accuracy, thereby guaranteeing the reliability of subsequent analyses. To enable a comprehensive exploration of potential effect modifiers, the data extraction process was further extended to capture intervention-specific details. Where reported in the primary systematic reviews, we extracted information on CPAP usage duration (e.g., mean hours/night) and adherence rates. Additionally, we sought to identify whether the intervention involved fixed-pressure CPAP or auto-adjusting CPAP (auto-CPAP) to assess whether different device types were considered in the existing literature.

### Extraction of repetition rate

In systematic reviews and meta-analyses, comprehensive collection and analysis of numerous primary studies within the same field are standard practice. However, a given primary study is frequently included in multiple distinct systematic reviews or meta-analyses. This may lead to duplicate counting of data in subsequent evidence synthesis, thereby potentially compromising the accuracy of conclusions. To scientifically assess and control potential biases in the study results caused by such overlap, this study established an overlap matrix between the systematic reviews/meta-analyses and the included primary studies. The degree of duplication of primary studies across different systematic reviews/meta-analyses was quantitatively evaluated by calculating the Corrected Covered Area (CCA) index (26). The calculation formula for this indicator is: CCA = (n − r) / (r × c − r), where n represents the total cumulative number of primary studies included in the systematic reviews/meta-analyses accounting for duplicate records, r denotes the total number of unique primary studies actually covered after removing duplicates across all systematic reviews/meta-analyses, and c indicates the final number of systematic reviews/meta-analyses included in this study. Based on the commonly adopted criteria in the literature: a CCA ≤ 5% indicates a low degree of overlap between studies, 5% < CCA ≤ 10% suggests moderate overlap, 10% < CCA ≤ 15% represents high overlap, and a CCA > 15% reflects an extremely high degree of overlap of primary studies across systematic reviews/meta-analyses. This approach provides a basis for the appropriate handling of duplicate data in subsequent analyses.

### Quality assessment

#### Risk of bias assessment

For the purpose of systematically evaluating the methodological quality and potential bias of the literature included in the meta-analysis, the Risk Of Bias In Systematic reviews (ROBIS) tool was utilized to assess the risk of bias in the relevant studies (27). This evaluation process follows the ROBIS guidelines and consists of three progressive phases: (1) assessing the relevance of the study topic to the present research question; (2) identifying potential risks of bias across various stages in the conduct of the systematic review/meta-analysis; and (3) formulating an overall judgment on the level of bias risk in the meta-analysis. The ROBIS tool employs several key assessment questions at each stage, and evaluators are required to judge each question as “yes,” “probably yes,” “no,” “probably no,” or “no information” based on specific details reported in the literature. Based on the responses to questions at each stage, the overall risk of bias for the meta-analysis is ultimately classified as “low risk,” “high risk,” or “unclear.” This assessment will serve as an important basis for subsequent grading of evidence quality and interpretation of results.

#### Methodological quality assessment

This study employed the Assessment of Multiple Systematic Reviews 2 (AMSTAR-2) tool to evaluate the methodological quality of the included literature (28, 29). The tool consists of 16 items, among which items 2, 4, 7, 9, 11, 13, and 15 are considered critical. The assessment results for each item are categorized as “met,” “not met,” or “partially met.” Based on the number of deficiencies in critical and non-critical items, the methodological quality can be classified into four grades: high, moderate, low, or very low. This grading system provides a structured and detailed methodological framework for evaluating the reliability and validity of a systematic review.

#### Reporting quality assessment

This study employed the Preferred Reporting Items for Systematic Reviews and Meta-Analyses 2020 (PRISMA 2020) statement to assess the reporting quality of the included systematic reviews and meta-analyses (30, 31). The guideline comprises seven sections (Title, Abstract, Introduction, Methods, Results, Discussion, and Other Information), with a total of 27 items and 42 sub-items. Each item was scored based on the completeness of reporting: a fully reported item (Y) was assigned 1 point, a partially reported item (PY) 0.5 points, and an unreported item (N) 0 points. The maximum total score was 42 points. Based on the total score, reporting quality was classified into three levels: a score of ≥33 points (≥80%) indicated high quality (relatively complete reporting); a score of 25–32 points (60–79%) indicated moderate quality (reporting with some deficiencies); and a score of <25 points (<60%) indicated low quality (reporting with substantial information gaps).

#### Evidence quality assessment

This study employed the Grading of Recommendations, Assessment, Development, and Evaluation (GRADE) system to systematically assess the quality of evidence presented in the included systematic reviews and meta-analyses (SRs/MAs) (32). This grading system is primarily based on a comprehensive evaluation of multiple key dimensions, including limitations in study design, inconsistency among results, indirectness of evidence, imprecision in effect estimates, likelihood of publication bias, and the presence of large effects, among other factors. By systematically evaluating and appraising each of the above dimensions, the GRADE system categorizes the overall quality of evidence into four distinct levels: “high,” “moderate,” “low,” and “very low.” This grading outcome helps clarify the degree of confidence and reliability that current evidence provides in supporting clinical decision-making.

### Quantitative analysis

This study conducted quantitative analyses on the included systematic reviews and meta-analyses (SRs/MAs) under the premise that the original studies contained therein exhibited sufficient homogeneity in outcome definition, measurement methods, and effect size types, or could be integrated through standardized approaches. For the expression of effect sizes, risk ratio (RR) is used as the summary statistic for dichotomous outcomes, while standardized mean difference (SMD) is employed for continuous outcomes. Both are reported as point estimates along with their corresponding 95% confidence intervals (CI). To evaluate the heterogeneity among studies, we used the I^2^ statistic combined with hypothesis testing for comprehensive judgment. The specific criteria were as follows: when I^2^ ≤ 50% and the heterogeneity test *p*-value > 0.1, the heterogeneity was considered low, and a fixed-effects model was used for pooled analysis; when I^2^ > 50% or the *p*-value ≤ 0.1, significant heterogeneity was deemed to exist, and a random-effects model was applied. If significant heterogeneity (i.e., I^2^ > 50%) was observed, we further explored its potential sources through sensitivity analysis or subgroup analysis, aiming to obtain more robust statistical conclusions by identifying and explaining the heterogeneity. Ultimately, all analyses were conducted to form methodologically rigorous and reliable comprehensive conclusions.

## Results

### Results of literature screening

**Figure 1:**
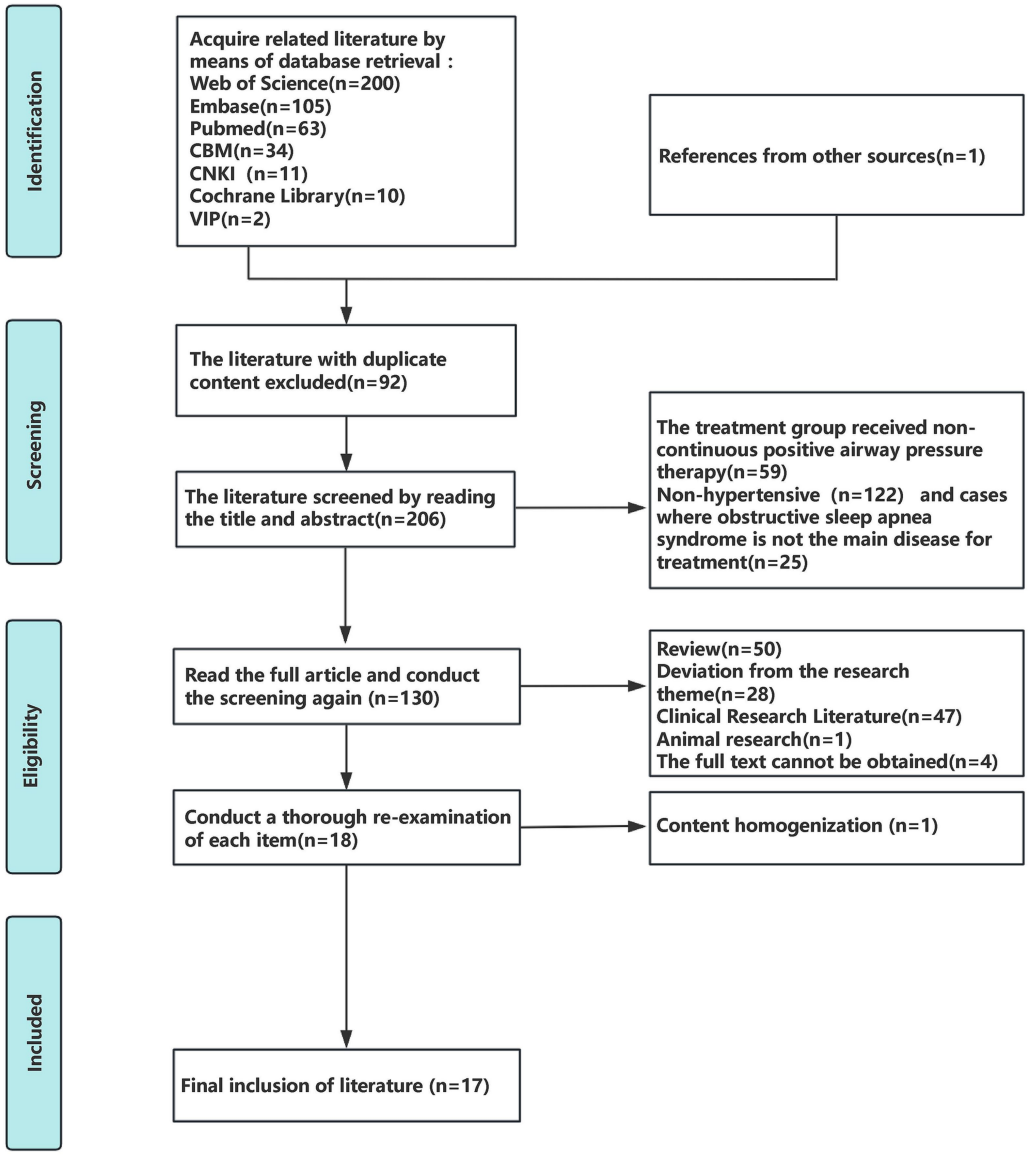
Literature screening process.

Initial screening retrieved 445 articles. Through other sources (manual screening of reference lists of relevant systematic reviews), 1 additional record was identified. After removing 92 duplicates using EndNote 20, the remaining records were subjected to title and abstract screening. A total of 122 publications on hypertension, 25 on non-obstructive sleep apnea, 59 with non-conforming interventions, 50 reviews, and 47 clinical trials were excluded. After a full-text review, 28 articles were excluded due to topic deviation, 1 due to content homogeneity, 4 due to incomplete data, and 1 animal study, resulting in the final inclusion of 17 articles (33–49). The screening process is illustrated in Figure 1.

### Basic characteristics of the included literature

The analysis included a total of 17 studies, comprising 12 English-language publications and 5 Chinese-language publications. Among these, 3 were theses/dissertations published between 2008 and 2013, and 14 were journal articles published between 2005 and 2007. All included studies compared CPAP therapy in the treatment group with control interventions (including sham CPAP, placebo, standard care, no treatment, or nocturnal supplemental oxygen) in the control group. Regarding outcome measures, 13 studies evaluated the 24-h average systolic blood pressure and 24-h average diastolic blood pressure (33, 36, 37, 39, 41–49); 12 studies reported the 24-h average daytime systolic blood pressure, 24-h average daytime diastolic blood pressure, 24-h average nighttime systolic blood pressure, and 24-h average nighttime diastolic blood pressure (36–38, 40, 42–49); 4 studies investigated 24-h mean arterial pressure (33, 34, 41, 42); 4 studies assessed systolic blood pressure and diastolic blood pressure (34, 35, 39, 40); 3 studies measured office systolic blood pressure and office diastolic blood pressure (42, 48, 49); Other metrics include Heart Rate (HR) (48), Risk of Cardiovascular Events (46), 2 studies assessing 24-h Mean Blood Pressure (24 h MBP) (36, 37), 2 studies assessing Daytime Mean Blood Pressure (36, 37), 2 studies assessing Nighttime Mean Blood Pressure (36, 37), Apnea Hypopnea Index (37), Percentage of Total Sleep Time with Oxygen Saturation <90% (37), Systolic Blood Pressure (SBP) in Hypertensive Patients (39) and Diastolic Blood Pressure (DBP) in Hypertensive Patients (39). The basic characteristics of the included studies are shown in Table 1.

**Table 1:** Characteristics of the included literature.

Author and year	Number of literatures/sample size	The types of original documents	Intervention measures		Bias risk measurement tool	Endpoint measure	The main conclusions of the author
			Treatment group	Control group			
Mo and He, 2007 (33)	7/471	RCT	CPAP	sham CPAP placebo Blank control	Jadad	⑤⑥⑱	Long-term CPAP therapy can reduce the 24-h mean diastolic blood pressure in patients with OSAHS.
Bazzano et al., 2007 (34)	10/587	RCT	CPAP	sham CPAP placebo standard care	N/A	⑭⑮⑱	CPAP decreases blood pressure among those with OSA and may help prevent hypertension.
Alajmi et al., 2007 (35)	16/818	RCT	CPAP	sham CPAP placebo standard care	N/A	⑭⑮	There was a trend for SBP reduction to be associated with CPAP compliance.
Haentjens et al., 2007 (36)	12/572	RCT	CPAP	sham CPAP placebo	N/A	①②③④⑤⑥⑦⑧⑨	CPAP therapy reduces 24-h ambulatory mean blood pressure in OSAS patients, with greater reductions observed in those with more severe OSAS and better nightly CPAP adherence. These blood pressure-lowering effects likely contribute to improved cardiovascular prognosis.
Wang, 2008 (37)	8/429	RCT	CPAP	sham CPAP	Cochrane	①②③④⑤⑥⑦⑧⑨⑩⑪	For patients with moderate-to-severe OSAHS, CPAP therapy reduces 24-h ambulatory blood pressure, including daytime diastolic, 24-h systolic, 24-h mean, and nighttime mean blood pressure, along with improvements in the hypopnea index and the percentage of sleep time with SpO₂ < 90%.
Montes et al., 2012 (38)	32/1948	RCT	CPAP	sham CPAP placebo standard care	N/A	①②③④	PAP treatment for OSA is associated with modest but significant reductions in diurnal and nocturnal SBP and DBP.
Yin, 2013 (39)	10/1182	RCT	CPAP	sham CPAP standard care	N/A	⑤⑥⑭⑮⑯⑰	CPAP treatment can effectively lower the blood pressure of patients with OSAHS.
Fava et al., 2014 (40)	31/1820	RCT	CPAP	sham CPAP placebo Standard care oral appliance Antihypertensive drugs	Jadad GRADE	①②③④⑭⑮	CPAP is associated with a significant but modest reduction in both systolic and diastolic BP, particularly during nighttime. The effect is greater in patients with more severe OSA and marked somnolence. Compared with antihypertensive drugs, CPAP has a weaker hypotensive effect and cannot be used as a standalone antihypertensive regimen. It is only recommended as an adjunctive therapy for patients with OSA complicated by hypertension.
Fu, 2014 (41)	9/610	RCT	CPAP	sham CPAP placebo Blank control	N/A	⑤⑥⑱	CPAP can reduce the systolic blood pressure, diastolic blood pressure and mean arterial pressure of patients with OSAHS.
Schein et al., 2014 (42)	16/1166	RCT	CPAP	sham CPAP	Cochrane	①②③④⑤⑥⑱⑲⑳	Treatment with CPAP promoted significantly but small reductions in blood pressure in individuals with OSA.
Li et al., 2015 (43)	11/1007	RCT	CPAP	placebo standard care	Jadad Cochrane	①②③④⑤⑥	CPAP is highly effective in improving hypertension in patients with obstructive sleep apnea syndrome.
Hu et al., 2015 (44)	7/794	RCT	CPAP	sham CPAP Standard care	Jadad	①②③④⑤⑥	CPAP therapy significantly reduces 24-h ambulatory blood pressure in OSAS patients with hypertension, with a particularly notable decrease in nocturnal systolic blood pressure. The blood pressure-lowering effect is even more pronounced in patients with resistant hypertension or those already taking antihypertensive medications.
Liu et al., 2016 (45)	5/446	RCT	CPAP	sham CPAP Standard care	Jadad	①②③④⑤⑥	For patients with obstructive sleep apnea complicated by resistant hypertension, continuous positive airway pressure therapy can effectively reduce 24-h ambulatory blood pressure and nocturnal diastolic blood pressure.
Sun et al., 2016 (46)	12/1720	RCT	CPAP	sham CPAP Standard care nocturnal supplemental oxygen	Cochrane	①②③④⑤⑥㉔	CPAP therapy was associated with a significantly decreased level of BP and cardiovascular events.
Labarca et al., 2021 (47)	10/606	RCT	CPAP	sham CPAP Standard care	Cochrane	①②③④⑤⑥	CPAP therapy improved BP, especially nighttime BP, in this population.
Shang et al., 2022 (48)	19/1904	RCT	CPAP	sham CPAP Blank control	Detsky Quality Assessment Scale	①②③④⑤⑥⑲⑳㉓	CPAP treatment was associated with BP reduction in patients with systemic hypertension and OSA.
Benning et al., 2025 (49)	75/10025	RCT	CPAP	sham CPAP placebo Standard care Blank control	Cochrane	①②③④⑤⑥⑲⑳	CPAP in OSA leads to a significant reduction in BP, especially in SBP and in nocturnal SBP.

①24-h average daytime systolic blood pressure; ②24-h average daytime diastolic blood pressure; ③24-h nocturnal systolic blood pressure; ④24-h nocturnal diastolic blood pressure; ⑤24-h average systolic blood pressure; ⑥24-h average diastolic blood pressure; ⑦24-h average blood pressure; ⑧Average blood pressure during the day; ⑨Average blood pressure at night; ⑩Apnea Hypopnea Index; ⑪Percentage of Total Sleep Time with Oxygen Saturation <90%;⑫Fixed effect model of blood pressure; ⑬Random effect model of blood pressure; ⑭systolic pressure; ⑮diastolic blood pressure; ⑯Systolic Blood Pressure in Hypertensive Patients; ⑰Diastolic Blood Pressure in Hypertensive Patients; ⑱ 24-h Mean Arterial Pressure; ⑲Office systolic blood pressure; ⑳Office diastolic blood pressure; ㉑Night-time mean arterial pressure; ㉒Daytime mean arterial pressure; ㉓Heart rate (HR); ㉔Risk of cardiovascular events; ㉕Change in aortic stiffness.

### Duplication rate of the original literature

This study included a total of 17 systematic reviews or meta-analyses (33–49), which collectively referenced 288 original studies from their source publications. After removing duplicates, 88 independent studies remained. The corrected covered area (CCA) was (288–88)/(88 × 17–88) ≈ 0.14, indicating a high degree of overlap. This reflects a certain level of duplication in the original studies among the included SAs/MAs.

### Reporting of CPAP usage duration and device type

Among the 17 included SRs/MAs, the reporting of intervention details pertinent to treatment efficacy was inconsistent. While the primary outcome was uniformly defined as the change in blood pressure, only a minority of the reviews systematically extracted or analyzed data on CPAP usage duration. Specifically, 5 reviews (36, 42, 46, 47, 49) discussed the potential dose–response relationship, noting that greater blood pressure reductions were often observed in patients with better adherence (e.g., using CPAP for ≥4 h per night). However, the pooled effect sizes were rarely stratified by usage thresholds. Critically, none of the included reviews considered or reported on the type of CPAP device used in the primary randomized controlled trials. The distinction between fixed-pressure CPAP and auto-CPAP, the latter being widely used in contemporary clinical practice, was absent from all methodological and analytical frameworks. This represents a significant evidence gap, as variations in device technology could influence patient comfort, long-term adherence, and ultimately, the magnitude of blood pressure reduction.

### Results of the risk of bias assessment

All included studies (33–49) were rated as “Low concern” in the Phase1 (study eligibility criteria) of the ROBIS tool. In Phase 2, all included studies (33–49) in Domain 1 (Eligibility Criteria) were rated as low risk of bias. In Domain 2 (Identification and Selection of Studies), 13 studies (34–38, 40–44, 47–49) were assessed as low risk of bias, one study (45) was rated as “unclear risk,” and three studies (33, 39, 46) were judged as “high risk.” In Domain 3 (Data Collection and Study Evaluation), 7 studies (37, 42–45, 47, 49) were assessed as low risk of bias, 9 studies (33–38, 41, 46, 48) were rated as “unclear risk,” and 1 study (39) was judged as “high risk.” In Domain 4 (Completeness of outcome data), all included studies (33–49) were rated as having a low risk of bias. In Phase 3 (Overall risk of bias assessment), 15 studies (33–38, 40–48) were judged to have a low risk of bias, while 2 studies (33, 39) were rated as having a “high risk.”

### Results of the methodological quality assessment

**Figure 2:**
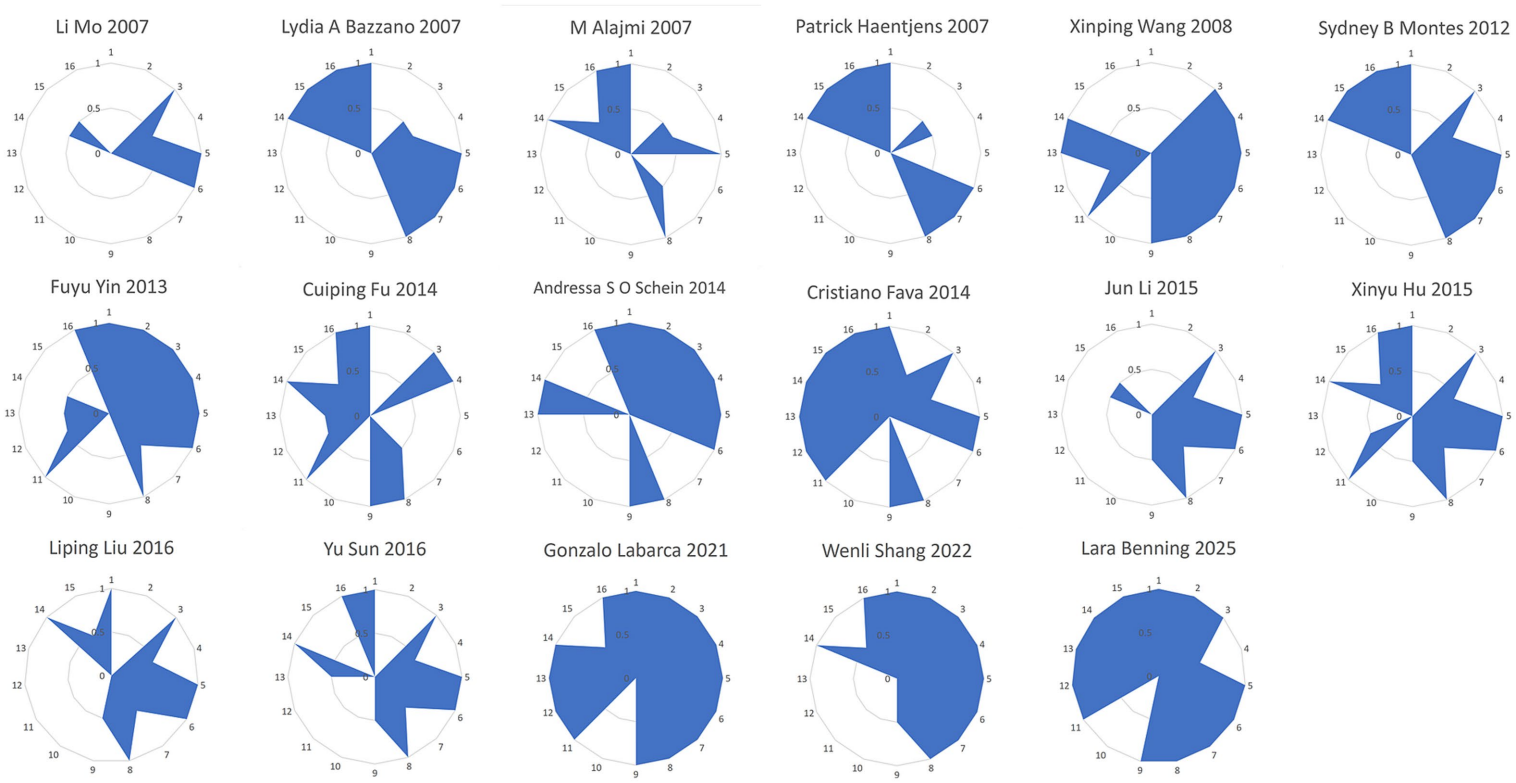
Radar chart of scores for each item of AMSTAR-2. Fully filled (1) indicates full compliance; partially filled (0.5) indicates partial.

In the included systematic reviews/meta-analyses (SRs/MAs), the results of the Assessment of Multiple Systematic Reviews 2 (AMSTAR-2) indicated that three studies were rated as high quality (40, 47, 49), while fourteen were rated as low quality (33–39, 41–46, 48). In terms of key items, Items 4 and 11 were fully reported (17 items/100%). The compliance rates for the remaining items were as follows: Item 7 (14 items/82%), Item 15 (13 items/76%), Item 9 (11 items/65%), Item 13 (9 items/53%), and Item 2 (11 items/65%). For non-critical items, Items 1, 3, 8, and 14 were fully reported (17 items/100%), while compliance varied for other items: Item 5 (15 items/88%) and Item 6 (15 items/88%), Item 12 (10 items/59%), Item 16 (14 items/82%), and Item 10 (0 items/0%). A detailed assessment of the methodological quality of the included studies is presented in Figure 2.

### Results of the reporting quality assessment

**Figure 3:**
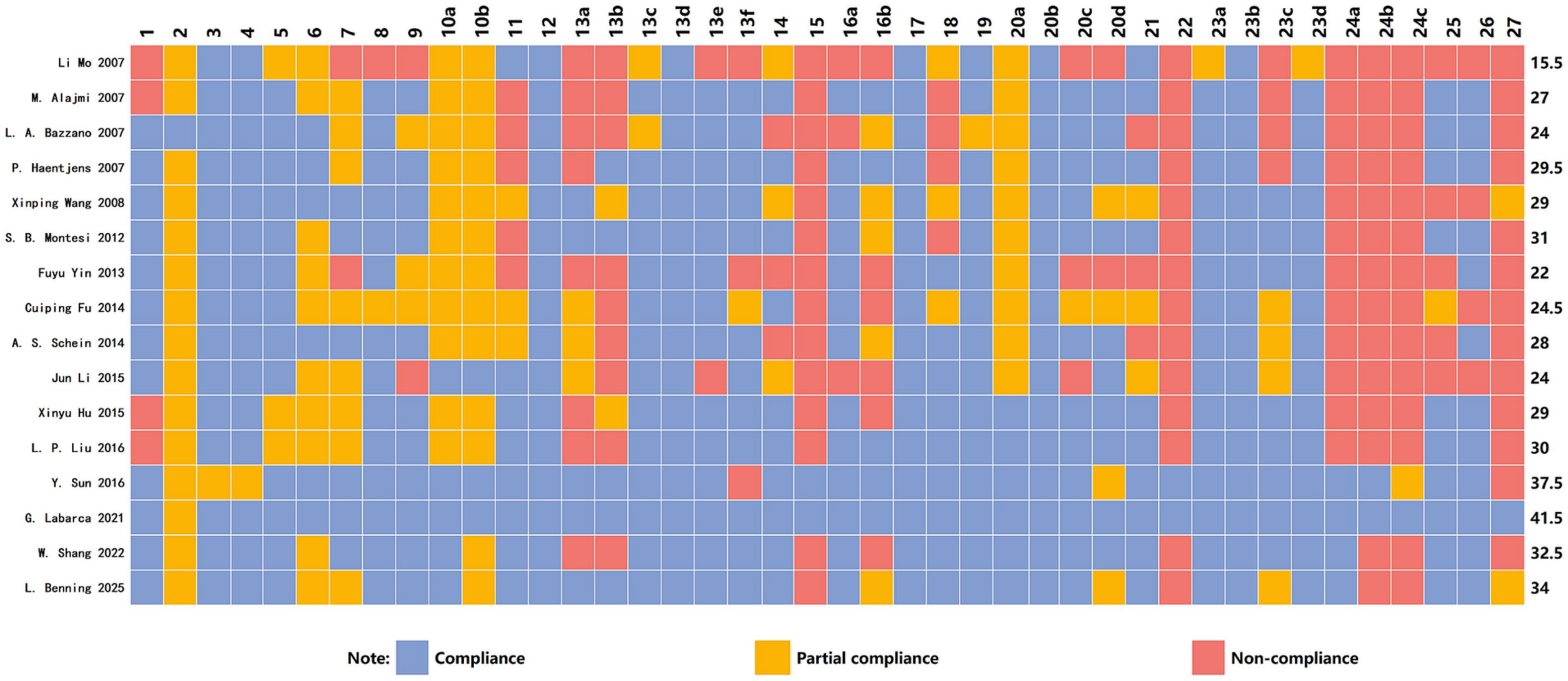
Cartesian heatmap of the scores of each item in PRISMA 2020.

The PRISMA 2020 checklist (maximum score: 42 points) provides comprehensive evaluation criteria for the abstract, introduction, methods, results, and discussion sections. The included studies scored between 15.5 and 41.5 points (mean = 29.1764) on the PRISMA 2020 assessment, with four classified as high-quality reports (40, 46, 47, 49), eight as moderate-quality reports (34, 36–38, 42, 44, 45, 48) and five as low-quality reports (33, 35, 39, 41, 43). Among the 42 checklist items, eight items (13a, 13b, 15, 22, 24a, 24b, 24c, 27) demonstrated poor reporting compliance, with an overall completion rate of ≤50% across the 20 studies. These weak items primarily encompass: synthesis methods, assessment of evidence certainty, results of evidence certainty, registration and study protocols, and accessibility of data, code, and other materials. Detailed distributions of the reported characteristics are presented in Figure 3.

### Results of the evidence quality assessment

We assessed the quality of evidence for 109 pooled outcome measures across the included studies using the GRADE methodology. The results showed that 4 outcome measures (3.6%) were rated as high-quality evidence, 29 (26.6%) as moderate-quality evidence, 51 (46.8%) as low-quality evidence, and 25 (22.9%) as very low-quality evidence. The complete GRADE evidence profiles for all outcome measures are presented in Table 2.

**Table 2:** Evidence quality assessment.

The included studies	Endpoint measure	Downgrading factor					Effect size	95%CI	I^2^/%	*p*	Evidence quality
		RB	IC	ID	IP	PB					
Mo and He, 2007 (33)	24-h mean diastolic blood pressure	−1^①^	0	0	0	0	WMD = −1.78	[−3.34,−0.22]	N/A	0.03	Medium
	24-h mean systolic blood pressure	0	−1^②^	0	−1^④^	0	WMD = −0.95	[−2.85, 0.94]	N/A	0.32	Low
	24-h mean blood pressure	−1^①^	−1^②^	−1^③^	0	0	WMD = −1.25	[−4.00, 1.49]	N/A	0.37	Extremely low
Bazzano et al., 2007 (34)	Systolic Blood Pressure	−1^①^	0	0	0	0	WMD = −2.46	[−4.31,−0.62]	N/A	0.009	Medium
	Diastolic Blood Pressure	−1^①^	0	0	0	0	WMD = −1.83	[−3.05,−0.61]	N/A	0.003	Medium
	Mean Arterial Pressure	−1^①^	0	0	0	0	WMD = −2.22	[−4.38,−0.05]	N/A	0.045	Medium
Alajmi et al., 2007 (35)	Systolic Blood Pressure	−1^①^	0	0	−1^④^	0	WMD = −1.38	[−3.60, 0.88]	N/A	0.23	Low
	Diastolic Blood Pressure	−1^①^	0	0	−1^④^	0	WMD = −1.52	[−3.1, 0.07]	N/A	0.06	Low
Haentjens et al., 2007 (36)	24-h ambulatory mean blood pressure	−1^①^	−1^②^	0	0	0	WMD = −1.69	[−2.69,−0.69]	41	<0.0001	Low
	24-h ambulatory systolic blood pressure	−1^①^	0	0	0	0	WMD = −1.77	[−3.00,−0.54]	19	0.005	Medium
	24-h ambulatory diastolic blood pressure	−1^①^	−1^②^	0	0	0	WMD = −1.79	[−2.87,−0.71]	43	<0.0001	Low
	Daytime mean blood pressure	−1^①^	−1^②^	0	−1^④^	−1^⑤^	WMD = −1.76	[−3.32, 0.20]	54	0.03	Extremely low
	Daytime systolic blood pressure	−1^①^	0	0	−1^④^	−1^⑤^	WMD = −2.25	[−4.25,−0.24]	0	0.03	Extremely low
	Daytime diastolic blood pressure	−1^①^	−1^②^	0	−1^④^	−1^⑤^	WMD = −2.87	[−4.36,−0.38]	36	0.02	Extremely low
	Nighttime mean blood pressure	−1^①^	0	0	0	−1^⑤^	WMD = −2.07	[−3.36,−0.78]	27	0.002	Low
	Nighttime systolic blood pressure	−1^①^	0	0	−1^④^	−1^⑤^	WMD = −2.82	[−5.48,−0.18]	13	0.04	Extremely low
	Nighttime diastolic blood pressure	−1^①^	−1^②^	0	−1^④^	−1^⑤^	WMD = −1.89	[−4.25, 0.46]	67	0.11	extremely low
Wang, 2008 (37)	Mean Daytime SBP	−1^①^	−1^②^	0	0	0	WMD = 0.71	[−0.31, 1.73]	52	0.12	Low
	Mean Daytime Diastolic Blood Pressure	−1^①^	−1^②^	0	0	0	WMD = 1.57	[0.17, 2.96]	58.2	0.09	Low
	Mean Nighttime Systolic Blood Pressure	−1^①^	−1^②^	0	−1^④^	−1^⑤^	WMD = 1.04	[−2.07, 4.14]	78.4	0.010	Extremely low
	Mean Nighttime Diastolic Blood Pressure	−1^①^	−1^②^	0	−1^④^	−1^⑤^	WMD = 1.09	[−1.91, 4.10]	88	0.0002	Extremely low
	24-Hour Mean Systolic Blood Pressure	−1^①^	0	0	0	−1^⑤^	WMD = 1.18	[0.49, 1.87]	0	0.65	Low
	24-Hour Mean Diastolic Blood Pressure	−1^①^	−1^②^	0	0	−1^⑤^	WMD = 1.11	[0.01, 2.20]	70	0.010	Extremely low
	24-Hour Mean Blood Pressure	−1^①^	0	0	0	−1^⑤^	WMD = 1.72	[1.18, 2.27]	29.5	0.24	Low
	Mean Daytime Blood Pressure	−1^①^	0	0	0	−1^⑤^	WMD = 0.53	[−0.02, 1.07]	38.9	0.19	Low
	Mean Nighttime Blood Pressure	−1^①^	0	0	0	−1^⑤^	WMD = 3.02	[2.40, 3.64]	0	0.47	Low
	Apnea Hypopnea Index	−1^①^	−1^②^	0	0	−1^⑤^	WMD = 45.73	[29.43, 62.04]	84	0.0003	Extremely low
	Percentage of Total Sleep Time with Oxygen Saturation <90%	−1^①^	0	0	0	−1^⑤^	WMD = 7.14	[4.96, 9.33]	0	0.64	Low
Montes et al., 2012 (38)	Diurnal Systolic Blood Pressure	−1^①^	0	0	0	0	WMD = −2.58	[−3.57,−1.59]	0	≤0.001	Medium
	Diurnal Diastolic Blood Pressure	−1^①^	0	0	0	0	WMD = −2.01	[−2.84, − 1.18]	22.6	≤0.001	Medium
	Nocturnal Systolic Blood Pressure	−1^①^	0	0	0	0	WMD = −4.09	[−6.24,−1.94]	5.7	≤0.001	Medium
	Nocturnal Diastolic Blood Pressure	−1^①^	0	0	−1^④^	0	WMD = −1.85	[−3.53,−0.17]	26.3	0.03	Low
Yin, 2013 (39)	Systolic Blood Pressure	−1^①^	0	0	0	0	WMD = −2.77	[−3.58,−1.95]	20	<0.00001	Medium
	Diastolic Blood Pressure	−1^①^	0	0	0	0	WMD = −3.13	[−3.53,−2.74]	3	<0.00001	Medium
	24-h Ambulatory Systolic Blood Pressure	−1^①^	0	0	0	0	WMD = −2.55	[−3.47,−1.63]	34	<0.00001	Medium
	24-h Ambulatory Diastolic Blood Pressure	−1^①^	0	0	0	0	WMD = −3.18	[−3.60,−2.76]	0	<0.00001	Medium
	Systolic Blood Pressure in Hypertensive Patients	−1^①^	0	0	−1^④^	0	WMD = −2.32	[−4.35,−0.30]	0	0.02	Low
	Diastolic Blood Pressure in Hypertensive Patients	−1^①^	0	0	−1^④^	0	WMD = −2.17	[−3.58,−0.77]	0	0.0002	Low
Fava et al., 2013 (40)	Systolic Blood Pressure	−1^①^	0	0	0	0	WMD = −2.6	[−3.5,−1.7]	N/A	<0.001	Medium
	Diastolic Blood Pressure	−1^①^	0	0	0	0	WMD = −2.0	[−2.7, −1.3]	N/A	<0.001	Medium
	Daytime Systolic BP	−1^①^	0	0	0	0	WMD = −2.2	[−2.9,−1.5]	N/A	<0.001	Medium
	Daytime Diastolic BP	−1^①^	0	0	0	0	WMD = −1.9	[−2.5,−1.3]	N/A	<0.001	Medium
	Nighttime Systolic BP	−1^①^	0	0	0	0	WMD = −3.8	[−4.6,−3.0]	N/A	<0.001	Medium
	Nighttime Diastolic BP	−1^①^	0	0	0	0	WMD = −1.8	[−2.4,−1.2]	N/A	<0.001	Medium
Fu, 2014 (41)	24-h Mean Systolic Blood Pressure	−1^①^	−1^②^	0	0	0	WMD = −2.57	[−3.60,−1.54]	96	<0.00001	Low
	24-h Mean Diastolic Blood Pressure	−1^①^	−1^②^	0	−1^④^	0	WMD = −4.91	[−9.40,−0.41]	100	0.03	Extremely low
	24-h Mean Arterial Pressure	−1^①^	−1^②^	0	−1^④^	0	WMD = −6.07	[−11.11,−1.03]	100	0.02	Extremely low
Schein et al., 2014 (42)	Office systolic blood pressure	−1^①^	0	0	0	0	WMD = −3.20	[−4.67, −1.72]	0	<0.0001	Medium
	Office diastolic blood pressure	−1^①^	−1^②^	0	−1^④^	0	WMD = −2.87	[−5.18, −0.55]	76	0.02	Extremely low
	24-h systolic blood pressure	−1^①^	−1^②^	0	−1^④^	0	WMD = −3.57	[−8.58, 1.44]	66	0.16	Extremely low
	24-h diastolic blood pressure	−1^①^	−1^②^	0	−1^④^	0	WMD = −3.46	[−6.75, −0.17]	71	0.04	Extremely low
	24-h mean arterial pressure	−1^①^	−1^②^	0	−1^④^	0	WMD = −3.56	[−6.79, −0.33]	74	0.03	Extremely low
	Daytime systolic blood pressure	−1^①^	0	0	−1^④^	0	WMD = −0.74	[−3.90, 2.41]	37	0.64	Low
	Daytime diastolic blood pressure	−1^①^	−1^②^	0	−1^④^	0	WMD = −1.86	[−4.55, 0.83]	61	0.18	Extremely low
	Night-time systolic blood pressure	−1^①^	−1^②^	0	−1^④^	−1^⑤^	WMD = −4.92	[−8.70, −1.14]	55	0.01	Extremely low
	Night-time diastolic blood pressure	−1^①^	−1^②^	0	−1^④^	0	WMD = −2.87	[−6.14, 0.40]	69	0.09	Extremely low
	Night-time mean arterial pressure	−1^①^	0	0	0	0	WMD = −2.56	[−4.43, −0.68]	31	0.008	Medium
	Daytime mean arterial pressure	−1^①^	−1^②^	0	−1^④^	0	WMD = −1.85	[−4.77, 1.07]	65	0.21	Extremely low
Li et al., 2015 (43)	Daytime systolic blood pressure change	−1^①^	−1^②^	0	0	0	WMD = −7.31	[−10.82, −3.80]	81	<0.0001	Low
	Daytime diastolic blood pressure change	−1^①^	−1^②^	0	0	0	WMD = −5.11	[−7.11, −3.11]	84	<0.0001	Low
	Night-time systolic blood pressure change	−1^①^	−1^②^	0	0	0	WMD = −9.34	[−12.37, −6.31]	77	<0.0001	Low
	Night-time diastolic blood pressure change	−1^①^	−1^②^	0	0	0	WMD = −6.35	[−9.15, −3.54]	83	<0.0001	Low
	24-h mean systolic blood pressure change	−1^①^	−1^②^	0	0	0	WMD = −5.82	[−8.32, −3.32]	79	<0.0001	Low
	24-h mean diastolic blood pressure change	−1^①^	−1^②^	0	0	0	WMD = −4.73	[−6.02, −3.44]	84	<0.0001	Low
Hu et al., 2015 (44)	24-h ambulatory systolic blood pressure net change	0	0	0	0	0	MD = −2.32	[−3.65, −1.00]	0	0.001	High
	24-h ambulatory diastolic blood pressure net change	0	0	0	0	0	MD = −1.98	[−2.82, −1.14]	21	<0.001	High
	Diurnal systolic blood pressure net change	−1^①^	−1^②^	0	−1^④^	0	MD = −3.58	[−8.04, 0.89]	89	0.117	Low
	Diurnal diastolic blood pressure net change	0	−1^②^	0	−1^④^	0	MD = −2.85	[−5.58,−0.12]	88	0.041	Low
	Nocturnal systolic blood pressure net change	0	0	0	0	0	MD = −2.74	[−4.26, −1.23]	0	<0.001	High
	Nocturnal diastolic blood pressure net change	0	0	0	0	0	MD = −1.75	[−2.79, −0.71]	0	0.001	High
Liu et al., 2016 (45)	24-h ambulatory systolic blood pressure net change	−1^①^	0	0	−1^④^	0	MD = −4.78	[−7.95, −1.61]	N/A	0.003	Low
	24-h ambulatory diastolic blood pressure net change	−1^①^	0	0	−1^④^	0	MD = −2.95	[−5.37, −0.53]	N/A	0.02	Low
	Daytime systolic blood pressure net change	−1^①^	−1^②^	0	−1^④^	0	MD = −3.15	[−9.20, 2.89]	N/A	0.31	Low
	Daytime diastolic blood pressure net change	−1^①^	−1^②^	0	−1^④^	0	MD = −2.51	[−6.23, 1.22]	N/A	0.19	Low
	Nighttime systolic blood pressure net change	−1^①^	−1^②^	0	−1^④^	0	MD = −1.89	[−4.14, 0.35]	N/A	0.10	Low
	Nighttime diastolic blood pressure net change	−1^①^	0	0	−1^④^	0	MD = −1.53	[−3.07, 0.00]	N/A	0.05	Low
Sun et al., 2016 (46)	24-h ambulatory systolic blood pressure net change	−1^①^	0	0	0	0	MD = −2.03	[−3.64, −0.42]	0	0.01	Medium
	24-h ambulatory diastolic blood pressure net change	−1^①^	0	0	0	0	MD = −1.79	[−2.89, −0.68]	0	0.001	Medium
	Daytime systolic blood pressure net change	−1^①^	−1^②^	0	−1^④^	0	MD = −1.41	[−3.80, 0.97]	43	0.25	Low
	Daytime diastolic blood pressure net change	−1^①^	0	0	0	0	MD = −1.43	[−2.67, −0.19]	0	0.02	Medium
	Nighttime systolic blood pressure net change	−1^①^	0	0	0	0	MD = −4.39	[−6.85, −1.93]	34	0.0005	Medium
	Nighttime diastolic blood pressure net change	−1^①^	0	0	0	0	MD = −1.64	[−2.88, −0.40]	0	0.009	Medium
	Clinic systolic blood pressure net change	−1^①^	0	0	0	0	MD = −4.53	[−6.07, −2.99]	70	<0.00001	Medium
	Clinic diastolic blood pressure net change	−1^①^	−1^②^	0	0	0	MD = −2.94	[−4.16, −1.72]	56	<0.00001	Low
	Risk of cardiovascular events	−1^①^	0	0	−1^④^	0	OR = 0.59	[0.36, 0.98]	0	0.04	Low
Labarca et al., 2021 (47)	Net change of 24-h ambulatory systolic blood pressure	−1^①^	−1^②^	0	−1^④^	−1^⑤^	MD = −5.06	[−7.98, −2.13]	69	<0.01	Extremely low
	Net change of 24-h ambulatory diastolic blood pressure	−1^①^	−1^②^	0	−1^④^	−1^⑤^	MD = −4.21	[−6.50, −1.93]	81	<0.01	Extremely low
	Net change of daytime systolic blood pressure	−1^①^	−1^②^	0	−1^④^	−1^⑤^	MD = −2.34	[−6.94, +2.27]	84	>0.05	Extremely low
	Net change of daytime diastolic blood pressure	−1^①^	−1^②^	0	−1^④^	−1^⑤^	MD = −2.14	[−4.96, −0.67]	78	<0.05	Extremely low
	Net change of nighttime systolic blood pressure	−1^①^	−1^②^	0	−1^④^	−1^⑤^	MD = −4.15	[−7.01, −1.29]	43	<0.01	Low
	Net change of nighttime diastolic blood pressure	−1^①^	−1^②^	0	−1^④^	−1^⑤^	MD = −1.95	[−3.32, −0.57]	40	<0.01	Extremely low
	Change in aortic stiffness	−1^①^	0	0	−1^④^	0	MD = −4.0	[−0.82, +0.02]	95	=0.05	Low
Shang et al., 2022 (48)	24-h systolic blood pressure	−1^①^	−1^②^	0	0	0	WMD = −5.01	[−6.94, 3.08]	75	<0.00001	Low
	24-h diastolic blood pressure	−1^①^	−1^②^	0	0	0	WMD = −3.30	[−4.32, −2.28]	60	<0.00001	Low
	Daytime systolic blood pressure	−1^①^	−1^②^	0	0	0	WMD = −4.34	[−6.27, −2.40]	80	<0.0001	Low
	Daytime diastolic blood pressure	−1^①^	−1^②^	0	0	0	WMD = −2.97	[−3.99, −1.95]	66	<0.00001	Low
	Nighttime systolic blood pressure	−1^①^	−1^②^	0	0	0	WMD = −3.55	[−5.08, −2.03]	67	<0.00001	Low
	Nighttime diastolic blood pressure	−1^①^	−1^②^	0	0	0	WMD = −2.33	[−3.27, −1.40]	57	<0.00001	Low
	Office systolic blood pressure	−1^①^	0	0	0	0	WMD = −3.67	[−5.76, −1.58]	0	0.0006	Medium
	Office diastolic blood pressure	−1^①^	0	0	0	0	WMD = −2.61	[−4.25, −0.97]	18	0.002	Medium
	Heart rate	−1^①^	0	0	0	0	WMD = −2.79	[−4.88, −0.71]	22	0.009	Medium
Benning et al., 2025 (49)	Office systolic blood pressure	−1^①^	−1^②^	0	0	0	MD = −2.5	[−3.8, −1.2]	N/A	<0.001	Low
	Office diastolic blood pressure	−1^①^	−1^②^	0	0	0	MD = −1.7	[−3.6, −1.6]	N/A	<0.001	Low

① Risk of bias (RB): Methodological quality of included studies was low, with biases in randomization, allocation concealment, and blinding. ② Inconsistency (IC): The heterogeneity was large and confidence interval overlap was low. ③ Indirectness (ID): The population was not broadly representative. ④ Imprecision (IP): Small sample size, 95% confidence intervals include null values. ⑤ Publication bias (PB): Few studies were included, the funnel plot was not symmetrical, Egger’s test found publication bias, results were positive, or there was no publication bias evaluation.

### Quantitative analysis

#### Symptom scores

##### 24-h average systolic blood pressure

**Figure 4:**
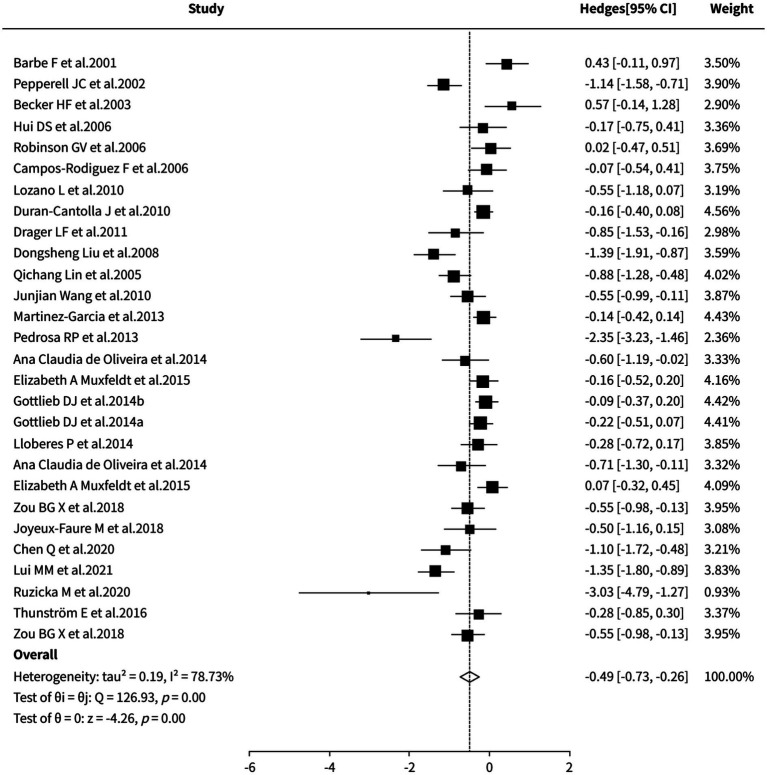
Meta-analysis of 24-h average systolic blood pressure.

A random-effects meta-analysis revealed that, compared to the control group (sham CPAP/placebo/standard care/blank control/nocturnal supplemental oxygen), CPAP treatment significantly reduced the 24-h mean systolic blood pressure in patients with obstructive sleep apnea (SMD = −0.49, 95% CI [−0.73, −0.26], *p* < 0.0001). However, significant heterogeneity was observed among studies (I^2^ = 78.73%). Sensitivity analysis, cumulative meta-analysis, and funnel plot results indicated that although the included studies exerted some influence on the stability of the pooled effect size and potential publication bias could not be ruled out, the primary conclusions remained essentially unchanged. Sensitivity and subgroup analyses were unable to specifically identify the source(s) of the aforementioned heterogeneity. Detailed results are shown in Figure 4.

##### 24-h average nighttime systolic blood pressure

**Figure 5:**
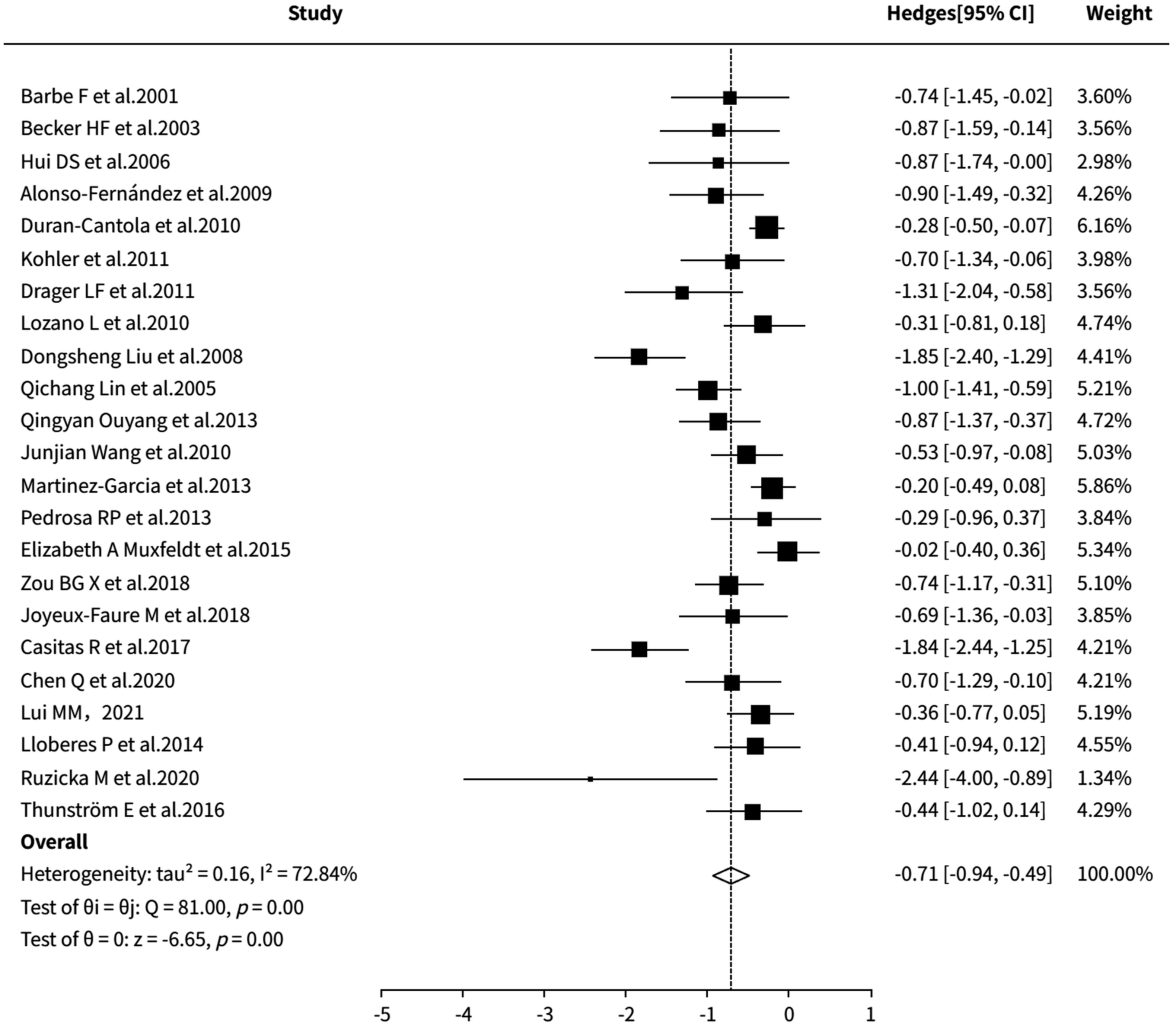
Meta-analysis of 24-h average nighttime systolic blood pressure.

The random-effects meta-analysis demonstrated that CPAP was significantly superior to sham control (including CPAP/placebo/standard care/no treatment/nocturnal supplemental oxygen) in reducing 24-h nocturnal systolic blood pressure (SMD = −0.71, 95% CI [−0.94, −0.49], *p* < 0.0001). However, substantial heterogeneity was observed across the included studies (I^2^ = 72.84%). Sensitivity and cumulative meta-analyses were unable to fully identify the exact sources of heterogeneity, indicating a certain degree of instability in the results. Therefore, the findings should be interpreted with caution in the context of clinical practice. Detailed results are presented in Figure 5.

##### 24-h average nighttime diastolic blood pressure

**Figure 6:**
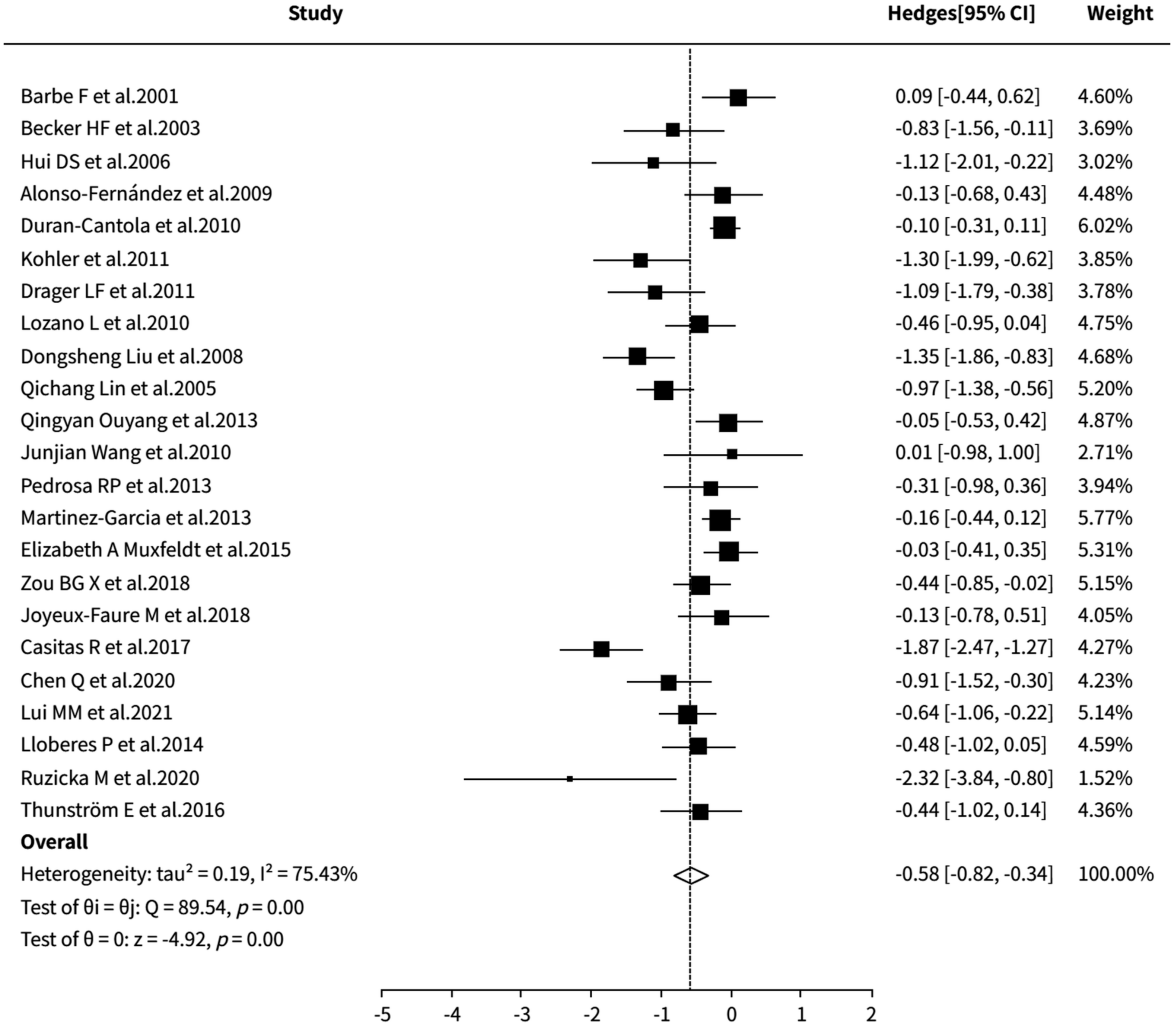
Meta-analysis of 24-h average nighttime diastolic blood pressure.

A random-effects meta-analysis revealed that CPAP therapy was significantly superior to control interventions (including sham CPAP, placebo, standard care, no treatment, or nocturnal supplemental oxygen) in reducing 24-h nocturnal diastolic blood pressure (SMD = −0.58, 95% CI [−0.82, −0.34], *p* < 0.0001). However, substantial heterogeneity was observed across the studies (I^2^ = 75.43%). Sensitivity analysis and cumulative meta-analysis both indicated stable effect sizes, yet the specific sources of heterogeneity could not be clearly identified. Detailed results are presented in Figure 6.

#### Office systolic blood pressure

**Figure 7:**
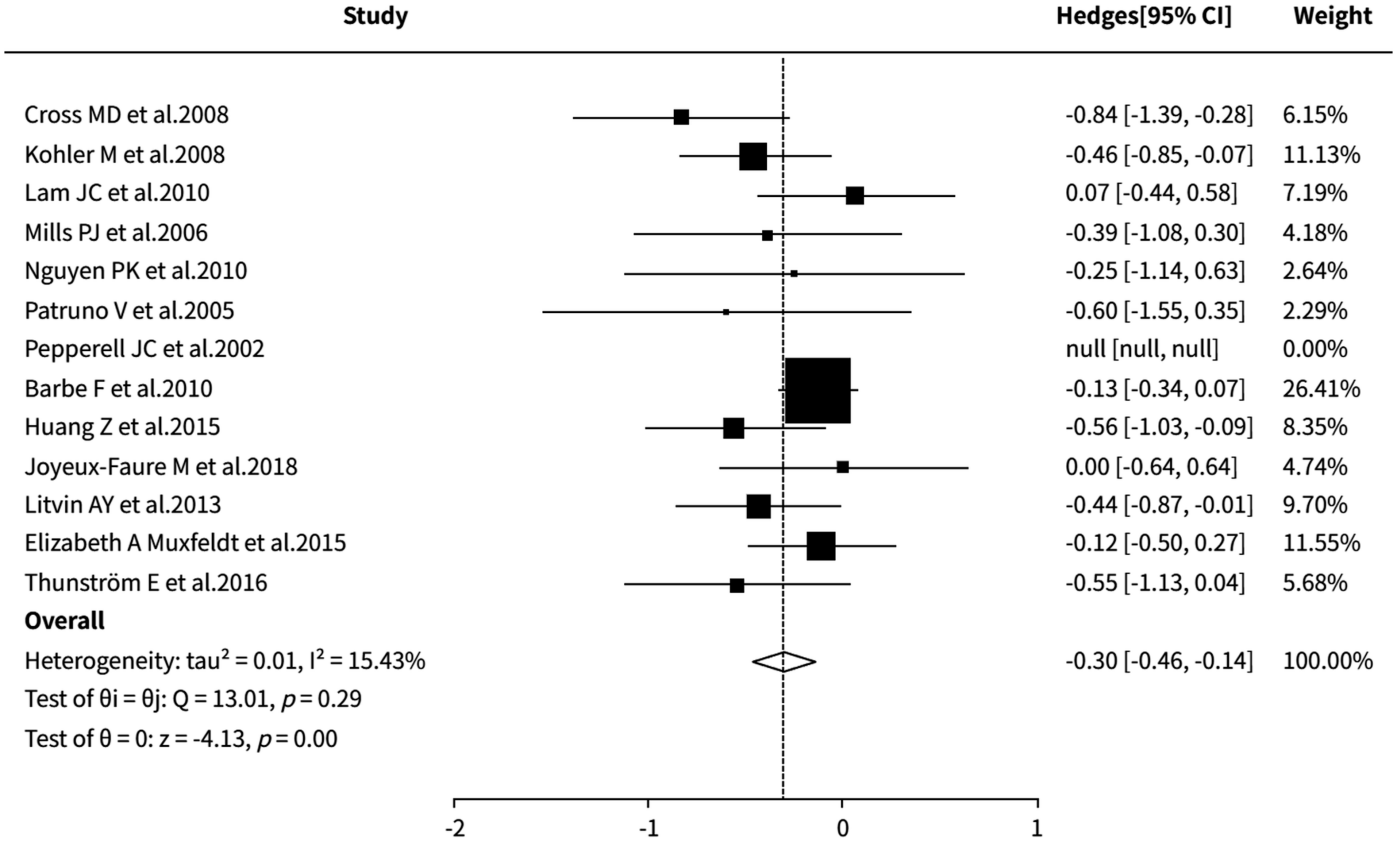
Meta-analysis of office systolic blood pressure.

The random-effects meta-analysis demonstrated that CPAP therapy was significantly superior to control groups (sham CPAP/placebo/standard care/no treatment/nocturnal supplemental oxygen) in reducing office systolic blood pressure (SMD = −0.30, 95% CI [−0.46, −0.14], *p* < 0.0001). The observed heterogeneity among studies was low (I^2^ = 15.43%), indicating relatively consistent results. Both sensitivity analysis and cumulative meta-analysis demonstrated stable effect sizes. Detailed results are presented in Figure 7.

#### Office diastolic blood pressure

**Figure 8:**
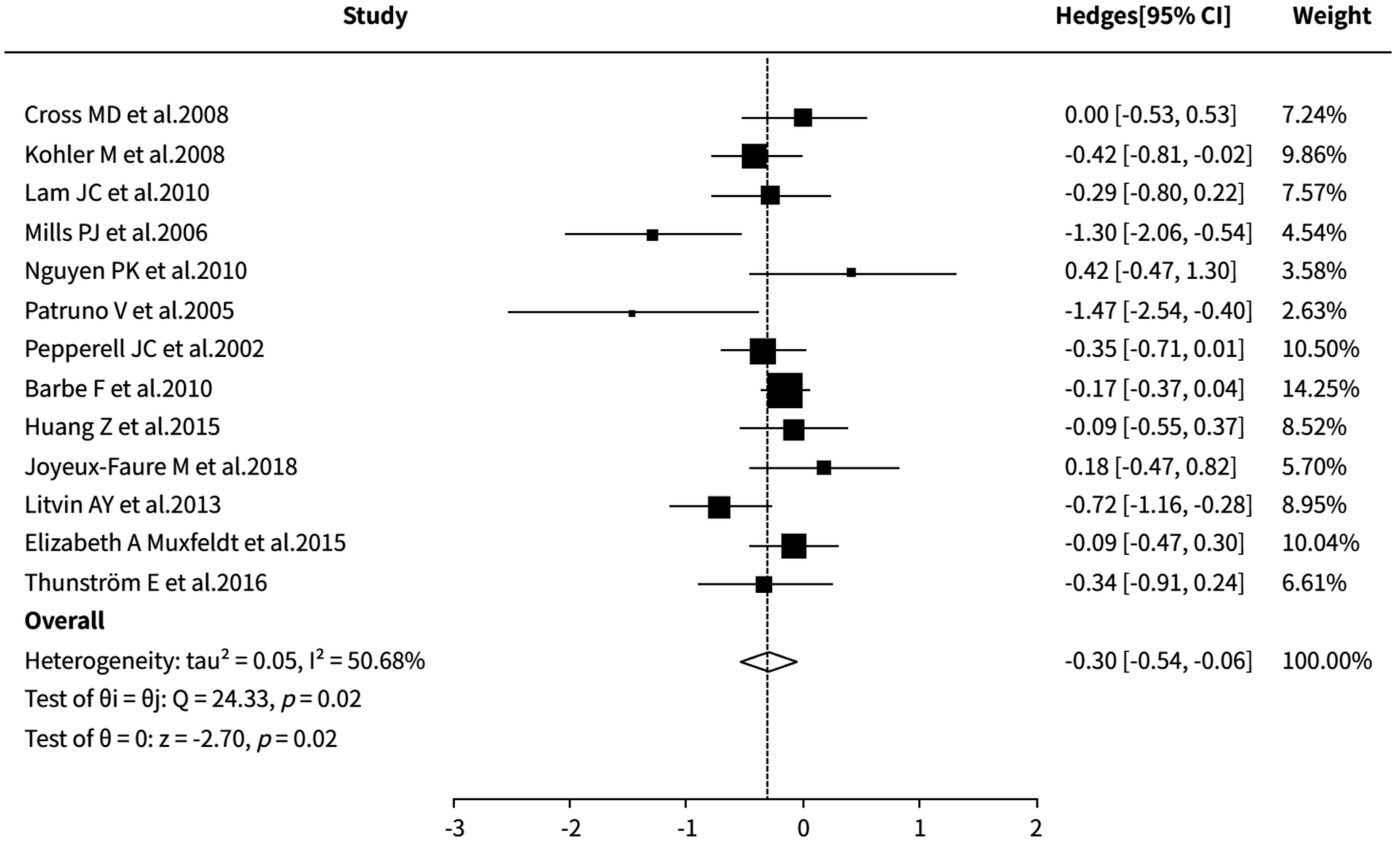
Meta-analysis of office diastolic blood pressure.

The random-effects meta-analysis revealed that CPAP therapy was significantly superior to control groups (sham CPAP/placebo/standard care/no treatment/nocturnal supplemental oxygen) in reducing office diastolic blood pressure (SMD = −0.30, 95% CI [−0.54, −0.06], *p* = 0.02). However, moderate heterogeneity was observed among the studies (I^2^ = 50.68%). Both sensitivity analysis and cumulative meta-analysis demonstrated stable effect sizes, but the specific sources of heterogeneity could not be clearly identified. Detailed results are shown in Figure 8.

### Qualitative analysis

#### 24-h mean blood pressure

Two systematic reviews/meta-analyses (36, 37) evaluated 24-h mean blood pressure as an outcome measure. The study found that continuous positive airway pressure (CPAP) therapy significantly reduced 24-h mean blood pressure (MBP) in patients with obstructive sleep apnea syndrome (OSAS) (37) Specifically, for every 10-unit increase in baseline apnea-hypopnea index (AHI), the 24-h MBP decreased by 0.89 mmHg, indicating a positive correlation between treatment-associated BP reduction and the severity of OSAS (36). Furthermore, each additional hour of nocturnal CPAP use was associated with an additional reduction in blood pressure of 1.39 mmHg, with more pronounced improvements in ambulatory blood pressure observed particularly in patients with severe disease and good compliance (36). This confirms that CPAP not only alleviates nocturnal blood pressure load but also exerts a sustained and stable positive regulatory effect on 24-h blood pressure through mechanisms such as relieving hypoxia, hypercapnia, and excessive sympathetic activation, thereby potentially improving cardiovascular outcomes (36, 37).

#### Daytime mean blood pressure

CPAP therapy demonstrates a statistically significant positive effect on improving daytime mean diastolic blood pressure, indicating its clinical value in blood pressure management for patients (37). This treatment achieved stable, modest yet clinically meaningful reductions in blood pressure during wakefulness, with a magnitude and heterogeneity similar to the improvements observed in nocturnal blood pressure (36). This effect not only stems from the amelioration of nocturnal respiratory events and hypoxia, but also exerts a sustained influence on blood pressure homeostasis across the entire 24-h period through multiple physiological mechanisms, including modulation of sympathetic nerve activity, improvement of endothelial function, and reduction of blood pressure variability (36). Overall, CPAP effectively reduces daytime blood pressure in patients with OSAS, providing an important pathophysiological basis for explaining its role in lowering the long-term risk of cardiovascular events (36).

#### Nighttime mean blood pressure

CPAP therapy can effectively mitigate the core pathophysiological mechanisms of obstructive sleep apnea–hypopnea syndrome (OSAHS), including intermittent hypoxia and the resulting sympathetic overactivation, by improving nocturnal respiratory events and sleep architecture disruption. Consequently, it exerts a positive regulatory effect on blood pressure levels during sleep in patients with OSAHS (36). This effect helps reduce nocturnal blood pressure burden and may further improve long-term cardiovascular prognosis in patients by lowering the risk of nocturnal cardiovascular events, providing high-level evidence to support the clinical value of CPAP in the management of OSAHS complicated with hypertension (37).

#### Systolic pressure

Nocturnal continuous positive airway pressure (CPAP) therapy exerts beneficial effects on obstructive sleep apnea (OSA)-related hypertension through multiple pathophysiological mechanisms, including alleviation of respiratory events, reduction of sympathetic tone, improvement of vascular endothelial function, and stabilization of hemodynamics. It should therefore be regarded as an essential component of integrated blood pressure management and hypertension prevention strategies (34). Although the direct effect of CPAP on systolic blood pressure is relatively limited, its antihypertensive effect may be more pronounced in patients with severe respiratory events or refractory hypertension, which may be associated with more marked nocturnal hypoxia and sympathetic activation (35). Evidence-based medicine has demonstrated that CPAP not only ameliorates respiratory events and sleep architecture in patients with OSAHS, but also effectively modulates systolic blood pressure through neuroendocrine and hemodynamic pathways, providing a critical foundation for the clinical management of OSAHS-related hypertension (39).

#### Diastolic blood pressure

CPAP exerts a more targeted regulatory effect in patients with pre-existing abnormal blood pressure regulation. The underlying mechanisms involve multiple pathophysiological pathways, including the elimination of nocturnal respiratory events, alleviation of hypoxia and hypercapnia, suppression of sympathetic overactivation, and improvement of vascular endothelial function. These collectively contribute to positive effects on nocturnal hemodynamic homeostasis, thereby laying the foundation for long-term diastolic blood pressure control (34). Furthermore, CPAP therapy can effectively reduce diastolic blood pressure levels in patients with OSAHS, an effect primarily attributed to its ability to correct nocturnal hypoxemia, suppress sympathetic overactivation, improve vascular endothelial function, and ultimately lower systemic vascular resistance (39).

## Discussion

### Summary of main findings and evidence quality

This overview systematically synthesized evidence from 17 SRs/MAs examining the effect of CPAP on blood pressure in patients with OSA. While our quantitative and qualitative analyses suggest that CPAP therapy is associated with modest reductions in several blood pressure parameters—particularly nocturnal systolic and diastolic blood pressure—these findings must be interpreted within the context of significant methodological limitations and low evidence certainty. Our pooled analyses indicated that CPAP use was associated with statistically significant reductions in 24-h systolic blood pressure (SMD = -0.49) nighttime systolic blood pressure (SMD = -0.71), and office blood pressure. However, the clinical significance of these modest effect sizes (typically in the range of 2–5 mmHg) remains debatable, especially given the low-to-very-low certainty of the evidence as assessed by GRADE. Only 4 out of 109 outcome measures were rated as high-quality evidence, with the majority downgraded due to methodological flaws in the original studies, inconsistency (high heterogeneity), and imprecision.

### Major methodological limitations

A paramount finding of this overview is the consistently low methodological quality of the included SRs/MAs. The AMSTAR-2 assessment revealed that 14 out of 17 reviews were of low quality, a critical limitation that suggests the evidence base we are synthesizing is built on a weak foundation. This low quality, driven by factors such as the absence of pre-registered protocols and inadequate integration of primary study risk of bias, means that the pooled effect sizes reported in the literature may be prone to bias. Furthermore, the high degree of overlap among primary studies (CCA = 14.2%) is a major concern. This substantial overlap indicates that the same set of original trials is being analyzed repeatedly across multiple reviews, artificially inflating the apparent consistency of conclusions. Consequently, the perceived robustness of the evidence for CPAP’s antihypertensive effect may be overstated.

### Clinical and statistical heterogeneity

The substantial statistical heterogeneity observed in many of our pooled analyses (e.g., I^2^ > 70% for 24-h BP outcomes) points to underlying clinical diversity that remains underexplored in the included literature. Based on multiple high-level evidence-based studies, continuous positive airway pressure (CPAP) therapy exhibits significant heterogeneity in its effects on blood pressure among patients with obstructive sleep apnea (OSA) (50). Factors such as baseline patient characteristics (OSA severity, burden of nocturnal hypoxemia, hypertension status), differences in blood pressure measurement modalities (ambulatory vs. office), and variations in study populations likely contribute to this heterogeneity. The inability to stratify by these crucial clinical variables across the included SRs/MAs limits our capacity to identify which specific OSA phenotypes are most likely to derive a clinically meaningful antihypertensive benefit from CPAP.

### The role of CPAP usage duration and device type in treatment efficacy

Beyond establishing the overall efficacy of CPAP, a nuanced understanding of its antihypertensive effects requires consideration of two critical and interrelated factors: treatment adherence and device technology. The findings from our quantitative synthesis, while confirming a significant blood pressure reduction, were characterized by substantial heterogeneity. A key contributor to this heterogeneity, which remains underexplored in the included SRs/MAs, is the variability in CPAP usage.

Consistent with a previous individual patient data meta-analysis (50) and a standard meta-analysis (51), our review suggests a dose–response relationship, wherein patients with higher nightly CPAP usage (typically ≥4 h) derive greater cardiovascular benefits. Further meta-analyses have substantiated that a positive correlation exists between improved treatment adherence and the magnitude of blood pressure reduction (52). Its core mechanism lies in correcting intermittent hypoxia and sleep fragmentation associated with respiratory events, thereby suppressing the overactivation of the sympathetic nervous system and the renin-angiotensin-aldosterone system (51). This therapeutic benefit is more evident in patient populations with higher baseline blood pressure, greater disease severity, and better adherence to treatment (51). The failure of most included reviews to consistently extract and analyze adherence data limits the precision of our pooled estimates and may obscure the true therapeutic potential of CPAP in highly compliant populations.

Furthermore, the technological evolution of CPAP devices presents an additional layer of complexity. The majority of the primary studies synthesized in this overview predate the widespread adoption of modern auto-CPAP devices. Auto-CPAP, by automatically adjusting pressure in response to flow limitation, snoring, and apneas, may improve patient comfort and long-term adherence compared to traditional fixed-pressure CPAP. However, the included SRs/MAs did not differentiate between these device types, creating a critical blind spot. It remains unclear whether auto-CPAP offers a superior blood pressure-lowering effect, either through enhanced adherence or more precise physiological correction. This omission underscores a disconnect between the evidence base and current clinical practice, where auto-CPAP is frequently the device of choice. Future overviews and primary research must prioritize the standardized reporting of usage duration and device type to enable more granular, clinically meaningful subgroup analyses and to guide personalized treatment strategies.

### Implications for research and practice

Despite these limitations, exploratory analyses hint at differential treatment effects across patient subgroups. The most consistent signal for a larger BP reduction appears in patients with resistant hypertension and those with higher baseline AHI and better CPAP adherence. These observations suggest that CPAP may function as a ‘phenotype-specific therapy,’ but this hypothesis requires confirmation in prospective trials that stratify randomization and analysis by these characteristics. In terms of safety, CPAP therapy is generally well-tolerated. Large-scale randomized controlled trials have reported no treatment-related serious adverse events, confirming its long-term safety profile. Treatment adherence was maintained during follow-up (53, 54). In terms of long-term cardiovascular implications, CPAP therapy has shown a trend toward reducing cardiovascular event risk (55) and improving non-dipper circadian blood pressure patterns (56). These observations support the possibility that cardiovascular benefits may be derived from CPAP’s impact on fundamental mechanisms linking OSAS to cardiovascular disease, such as reducing sympathetic activity and improving endothelial function (57). These observations, while promising, are derived from a body of evidence with substantial heterogeneity and low certainty, underscoring the need for more robust trials to confirm these potential benefits. Future efforts should focus on individualized optimization based on patient phenotypes (58–60).

To achieve this goal, future research must prioritize several key areas. First, improved methodological rigor in SRs/MAs is essential, including prospective registration, adherence to PRISMA guidelines, and systematic integration of GRADE assessments. Second, standardized reporting of CPAP interventions is urgently needed. Future primary studies and meta-analyses should report detailed information on device type (fixed vs. auto-CPAP), pressure settings, and objective adherence metrics (e.g., average hours of use per night) to enable nuanced dose–response analyses. Third, large-scale, long-term real-world studies incorporating diverse outcome measures such as ambulatory blood pressure monitoring are required to clarify the long-term cardiovascular benefits of CPAP and to identify patient subgroups most likely to respond.

### Hypotheses for future investigation

Based on our synthesis, we propose several hypotheses that warrant investigation in future prospective studies:

(1) The “Two-Phase Model” hypothesis: Blood pressure improvement with CPAP may involve a short-term “hemodynamic rapid response phase” (within weeks, directly reducing nocturnal blood pressure) and a long-term “neurovascular remodeling phase” (over months, improving all-day blood pressure homeostasis). (2) The “Dose-Response Curve Heterogeneity” hypothesis: The relationship between CPAP usage duration and blood pressure reduction may vary according to the patient’s OSA phenotype and cardiometabolic characteristics, with different subpopulations requiring different durations to reach therapeutic plateau.(3) The “Covert Cardiovascular Protection” hypothesis: Beyond direct blood pressure-lowering effects, CPAP may reduce target organ damage through mechanisms such as stabilizing blood pressure variability and restoring circadian rhythms. This protective effect could persist even in patients with limited blood pressure reduction and may operate independently of office blood pressure measurements. (4) The “Synergistic Effect” hypothesis: The combination of CPAP with drugs targeting specific pathological mechanisms or lifestyle interventions may produce synergistic effects in individuals with suboptimal antihypertensive responses.

### Limitations

This study also has certain limitations, which primarily stem from the methodological nature of the overview of systematic reviews itself: (1) Dependence on the evidence chain: The conclusions of this study fundamentally rely on the methodological quality and reporting completeness of the included systematic reviews/meta-analyses. Potential biases in the original studies may be indirectly transmitted to the current comprehensive synthesis through secondary evidence. (2) Timeliness and evolution: Methodological standards and clinical practice guidelines are continuously updated. Although the included studies fall within the acceptable timeframe for publication, the original evidence synthesized in these studies may already be outdated, limiting the contemporary relevance of the conclusions. (3) Heterogeneity in the level of synthesis: Differences across systematic reviews in terms of population definitions, intervention details, and outcome measures make it difficult to fully reconcile conceptual and measurement inconsistencies when integrating evidence at a higher level, which may affect the precise interpretation of the findings. (4) Underexplored intervention-level heterogeneity: The included SRs/MAs lacked consistent data on critical intervention-level variables, specifically CPAP usage duration and device type (fixed-pressure vs. auto-CPAP). The absence of this information prevented us from conducting subgroup analyses to explore their impact on blood pressure outcomes, which may be a significant source of the heterogeneity observed in our quantitative synthesis. (5)Inability to perform phenotype-specific subgroup analyses: As an overview of systematic reviews, this study is inherently constrained by the reporting completeness of the included SRs/MAs. Although we fully recognize the clinical value of subgroup analyses based on key phenotypes—such as OSA severity (e.g., mild vs. severe), hypertension subtype (e.g., resistant hypertension vs. non-resistant hypertension), and CPAP adherence—the majority of the included systematic reviews did not provide stratified data according to these critical variables. Consequently, we were unable to extract sufficient information to conduct secondary subgroup analyses that could elucidate differential treatment effects across distinct patient populations. This limitation underscores the need for future research to prioritize: (a) standardized reporting of stratified results in original systematic reviews and meta-analyses, and (b) the conduct of individual patient data meta-analyses (IPD-MAs) to more precisely identify the differential efficacy and long-term cardiovascular benefits of CPAP across diverse clinical phenotypes.

## Conclusion

This overview suggests that CPAP treatment for OSA-associated hypertension is associated with modest reductions in blood pressure, particularly in nocturnal measures. However, due to the predominantly low-to-very-low certainty of the evidence, substantial methodological weaknesses in the included reviews, and significant overlap of primary studies, these findings should be considered preliminary. The true magnitude of the antihypertensive effect, its clinical significance, and the patient populations most likely to benefit remain uncertain.

Future research should prioritize: (1) standardizing the reporting of device type (e.g., fixed-pressure vs. auto-CPAP) and treatment adherence (e.g., mean hours of use); (2) moving beyond average effect sizes to identify patient phenotypes most likely to benefit from specific CPAP modalities and usage thresholds; and (3) conducting large-scale, long-term real-world studies incorporating diverse outcome measures such as ambulatory blood pressure monitoring. Such studies are needed to confirm these findings and to inform more precise, evidence-based clinical practice guidelines for the management of hypertension in patients with OSA.

## Data Availability

The original contributions presented in the study are included in the article/Supplementary material, further inquiries can be directed to the corresponding authors.
