# Supplementary material for: Efficacy and safety of continuous positive airway pressure on blood pressure in patients with obstructive sleep apnea: an overview of systematic reviews and meta analyses
Source: Front Med (Lausanne). 2026 Mar 24;13:1785996. doi: 10.3389/fmed.2026.1785996 (PMC13055550; doi:10.3389/fmed.2026.1785996)
Supplement: Supplementary file 2 [file Table_2.DOCX]

Research Process and Research Data

Contents

[1.GRADE 2](#_Toc24073)

[2.AMSTAR-2 1](#_Toc18260)6

[3.PRISMA 2020 3](#_Toc7613)0

[4.ROBIS 8](#_Toc27087)1

[5.Citation Overlap Matrix and the Corrected Covered Area (CCA) 8](#_Toc17699)5

[6.Literature Screening. 8](#_Toc11321)8

[7..Search Query and Search Logic 13](#_Toc9394)4

GRADE

A:The conclusions of researcher Ph.D. Jingjing Liu

B:The conclusions of researcher Ph.D. Yan Cui.

C:In case of a difference of opinions, it shall be adjudicated by Professor Yongshi Liu.

D:Conclusive conclusion.

①Methodological quality of included studies was low, with biases in randomization, allocation concealment, and blinding. ②The heterogeneity was large and low confidence interval overlap. ③The population was not broadly representative. ④Small sample size, 95% confidence intervals include null values. ⑤Few studies were included, the funnel plot was not symmetrical, Egger’s test found that publication bias or results were positive, and there was no publication bias evaluation.

Study 33：莫莉 & 何权瀛.(2007).长期持续气道正压通气对阻塞性睡眠呼吸暂停低通气综合征患者血压影响的荟萃分析.中华医学杂志,87(17),1177-1180.

| Endpoint measure | Downgrading factor | A | B | C | D |
| --- | --- | --- | --- | --- | --- |
| 24-hour mean diastolic blood pressure | Risk of bias | 0 | -1^①^ | -1^①^ | -1^①^ |
|  | Inconsistency | 0 | 0 | - | 0 |
|  | Indirectness | 0 | 0 | - | 0 |
|  | Impression | 0 | -1^④^ | 0 | 0 |
|  | Publication bias | 0 | 0 | - | 0 |
| 24-hour mean systolic blood pressure | Risk of bias | 0 | -1^①^ | 0 | -1^①^ |
|  | Inconsistency | 0 | -1^②^ | -1^②^ | -1^②^ |
|  | Indirectness | 0 | 0 | - | 0 |
|  | Impression | -1^④^ | -1^④^ | - | -1^④^ |
|  | Publication bias | -1^⑤^ | 0 | 0 | 0 |
| 24-hour mean blood pressure | Risk of bias | -1^①^ | -1^①^ | - | -1^①^ |
|  | Inconsistency | 0 | -1^②^ | -1^②^ | -1^②^ |
|  | Indirectness | -1^③^ | 0 | -1^③^ | -1^③^ |
|  | Impression | 0 | -1^④^ | 0 | 0 |
|  | Publication bias | -1^⑤^ | 0 | 0 | 0 |

Study 34：Bazzano LA, Khan Z, Reynolds K, He J. Effect of nocturnal nasal continuous positive airway pressure on blood pressure in obstructive sleep apnea. Hypertension. 2007 Aug;50(2):417-23. doi: 10.1161/HYPERTENSIONAHA.106.085175. Epub 2007 Jun 4. PMID: 17548722.

| Endpoint measure | Downgrading factor | A | B | C | D |
| --- | --- | --- | --- | --- | --- |
| Systolic Blood Pressure | Risk of bias | -1^①^ | -1^①^ | - | -1^①^ |
|  | Inconsistency | 0 | 0 | - | 0 |
|  | Indirectness | 0 | 0 | - | 0 |
|  | Impression | 0 | 0 | - | 0 |
|  | Publication bias | -1^⑤^ | 0 | 0 | 0 |
| Diastolic Blood Pressure | Risk of bias | -1^①^ | -1^①^ | - | -1^①^ |
|  | Inconsistency | 0 | 0 | - | 0 |
|  | Indirectness | 0 | 0 | - | 0 |
|  | Impression | 0 | 0 | - | 0 |
|  | Publication bias | 0 | 0 | - | 0 |
| Mean Arterial Pressure | Risk of bias | -1^①^ | -1^①^ | - | -1^①^ |
|  | Inconsistency | 0 | 0 | - | 0 |
|  | Indirectness | 0 | 0 | - | 0 |
|  | Impression | 0 | 0 | - | 0 |
|  | Publication bias | 0 | 0 | - | 0 |

Study 35：Alaji M, Mulgrew AT, Fox J, Davidson W, Schulzer M, Mak E, Ryan CF, Fleetham J, Choi P, Ayas NT. Impact of continuous positive airway pressure therapy on blood pressure in patients with obstructive sleep apnea hypopnea: a meta-analysis of randomized controlled trials. Lung. 2007 Mar-Apr;185(2):67-72. doi: 10.1007/s00408-006-0117-x. Epub 2007 Mar 28. PMID: 17393240.

| Endpoint measure | Downgrading factor | A | B | C | D |
| --- | --- | --- | --- | --- | --- |
| Systolic Blood Pressure | Risk of bias | -1^①^ | -1^①^ | - | -1^①^ |
|  | Inconsistency | 0 | 0 | - | 0 |
|  | Indirectness | 0 | 0 | - | 0 |
|  | Impression | -1^④^ | -1^④^ | - | -1^④^ |
|  | Publication bias | 0 | 0 | - | 0 |
| Diastolic Blood Pressure | Risk of bias | -1^①^ | -1^①^ | - | -1^①^ |
|  | Inconsistency | 0 | 0 | - | 0 |
|  | Indirectness | 0 | 0 | - | 0 |
|  | Impression | -1^④^ | -1^④^ | - | -1^④^ |
|  | Publication bias | 0 | 0 | - | 0 |

Study 36：Haentjens P, Van Meerhaeghe A, Moscariello A, De Weerdt S, Poppe K, Dupont A, Velkeniers B. The impact of continuous positive airway pressure on blood pressure in patients with obstructive sleep apnea syndrome: evidence from a meta-analysis of placebo-controlled randomized trials. Arch Intern Med. 2007 Apr 23;167(8):757-64. doi: 10.1001/archinte.167.8.757. PMID: 17452537.

| Endpoint measure | Downgrading factor | A | B | C | D |
| --- | --- | --- | --- | --- | --- |
| 24-hour ambulatory mean blood pressure | Risk of bias | -1^①^ | -1^①^ | - | -1^①^ |
|  | Inconsistency | 0 | -1^②^ | -1^②^ | -1^②^ |
|  | Indirectness | 0 | 0 | - | 0 |
|  | Impression | 0 | -1^④^ | 0 | 0 |
|  | Publication bias | 0 | 0 | - | 0 |
| 24-hour ambulatory systolic blood pressure | Risk of bias | -1^①^ | 0 | -1^①^ | -1^①^ |
|  | Inconsistency | 0 | 0 | - | 0 |
|  | Indirectness | 0 | 0 | - | 0 |
|  | Impression | 0 | -1^④^ | 0 | 0 |
|  | Publication bias | 0 | 0 | - | 0 |
| 24-hour ambulatory diastolic blood pressure | Risk of bias | -1^①^ | -1^①^ | - | -1^①^ |
|  | Inconsistency | 0 | -1^②^ | -1^②^ | -1^②^ |
|  | Indirectness | 0 | 0 | - | 0 |
|  | Impression | 0 | -1^④^ | 0 | 0 |
|  | Publication bias | 0 | 0 | - | 0 |
| Daytime mean blood pressure | Risk of bias | -1^①^ | 0 | -1^①^ | -1^①^ |
|  | Inconsistency | -1^②^ | -1^②^ | - | -1^②^ |
|  | Indirectness | 0 | 0 | - | 0 |
|  | Impression | 0 | -1^④^ | -1^④^ | -1^④^ |
|  | Publication bias | 0 | -1^⑤^ | -1^⑤^ | -1^⑤^ |

| Daytime systolic blood pressure | Risk of bias | -1^①^ | 0 | -1^①^ | -1^①^ |
| --- | --- | --- | --- | --- | --- |
|  | Inconsistency | 0 | 0 | - | 0 |
|  | Indirectness | 0 | 0 | - | 0 |
|  | Impression | 0 | -1^④^ | -1^④^ | -1^④^ |
|  | Publication bias | 0 | -1^⑤^ | -1^⑤^ | -1^⑤^ |
| Daytime diastolic blood pressure | Risk of bias | -1^①^ | 0 | -1^①^ | -1^①^ |
|  | Inconsistency | 0 | -1^②^ | -1^②^ | -1^②^ |
|  | Indirectness | 0 | 0 | - | 0 |
|  | Impression | 0 | -1^④^ | -1^④^ | -1^④^ |
|  | Publication bias | 0 | -1^⑤^ | -1^⑤^ | -1^⑤^ |
| Nighttime mean blood pressure | Risk of bias | -1^①^ | 0 | -1^①^ | -1^①^ |
|  | Inconsistency | 0 | 0 | - | 0 |
|  | Indirectness | 0 | 0 | - | 0 |
|  | Impression | 0 | -1^④^ | 0 | 0 |
|  | Publication bias | 0 | -1^⑤^ | -1^⑤^ | -1^⑤^ |
| Nighttime systolic blood pressure | Risk of bias | -1^①^ | 0 | -1^①^ | -1^①^ |
|  | Inconsistency | 0 | 0 | - | 0 |
|  | Indirectness | 0 | 0 | - | 0 |
|  | Impression | 0 | -1^④^ | -1^④^ | -1^④^ |
|  | Publication bias | 0 | -1^⑤^ | -1^⑤^ | -1^⑤^ |
| Nighttime diastolic blood pressure | Risk of bias | -1^①^ | 0 | -1^①^ | -1^①^ |
|  | Inconsistency | -1^②^ | -1^②^ | - | -1^②^ |
|  | Indirectness | 0 | 0 | - | 0 |
|  | Impression | -1^④^ | -1^④^ | - | -1^④^ |
|  | Publication bias | 0 | -1^⑤^ | -1^⑤^ | -1^⑤^ |

Stuy 37：王馨平.(2008).持续正压气道通气对阻塞性睡眠呼吸暂停低通气综合征中重度患者24小时动态血压影响的有效性评价(硕士学位论文,兰州大学).硕士https://kns.cnki.net/kcms2/article/abstract?v=4J3GFaKSuTNMYmPcyan8kuVkV2VnEQNXwi7Wz3z1Pc6I3PkNDY1z6aiaiVIvonINk498u-A5yMuoYpkxMeJV59TOZsLyqQKkoaIwqUjsZbOyOJIz2KN4hP4M4Os-qnB96-Ep_LOT9mpzMORxYgJQEH3frLwo397E8hxuZ8YdG_9liLSTMRuegA==&uniplatform=NZKPT&language=CHS

| Endpoint measure | Downgrading factor | A | B | C | D |
| --- | --- | --- | --- | --- | --- |

| Mean Daytime Systolic Blood Pressure | Risk of bias | -1^①^ | 0 | -1^①^ | -1^①^ |
| --- | --- | --- | --- | --- | --- |
|  | Inconsistency | 0 | -1^②^ | -1^②^ | -1^②^ |
|  | Indirectness | 0 | 0 | - | 0 |
|  | Impression | 0 | -1^④^ | 0 | 0 |
|  | Publication bias | 0 | 0 | - | 0 |
| Mean Daytime Diastolic Blood Pressure | Risk of bias | -1^①^ | 0 | -1^①^ | -1^①^ |
|  | Inconsistency | 0 | -1^②^ | -1^②^ | -1^②^ |
|  | Indirectness | 0 | 0 | - | 0 |
|  | Impression | 0 | -1^④^ | 0 | 0 |
|  | Publication bias | 0 | 0 | - | 0 |
| Mean Nighttime Systolic Blood Pressure | Risk of bias | -1^①^ | -1^①^ | - | -1^①^ |
|  | Inconsistency | -1^②^ | -1^②^ | - | -1^②^ |
|  | Indirectness | 0 | 0 | - | 0 |
|  | Impression | 0 | -1^④^ | -1^④^ | -1^④^ |
|  | Publication bias | 0 | -1^⑤^ | -1^⑤^ | -1^⑤^ |
| Mean Nighttime Diastolic Blood Pressure | Risk of bias | -1^①^ | -1^①^ | - | -1^①^ |
|  | Inconsistency | -1^②^ | -1^②^ | - | -1^②^ |
|  | Indirectness | 0 | 0 | - | 0 |
|  | Impression | 0 | -1^④^ | -1^④^ | -1^④^ |
|  | Publication bias | 0 | -1^⑤^ | -1^⑤^ | -1^⑤^ |
| 24-Hour Mean Systolic Blood Pressure | Risk of bias | -1^①^ | -1^①^ | - | -1^①^ |
|  | Inconsistency | 0 | 0 | - | 0 |
|  | Indirectness | 0 | 0 | - | 0 |
|  | Impression | 0 | 0 | - | 0 |
|  | Publication bias | 0 | -1^⑤^ | -1^⑤^ | -1^⑤^ |
| 24-Hour Mean Diastolic Blood Pressure | Risk of bias | -1^①^ | -1^①^ | - | -1^①^ |
|  | Inconsistency | -1^②^ | -1^②^ | - | -1^②^ |
|  | Indirectness | 0 | 0 | - | 0 |
|  | Impression | 0 | -1^④^ | 0 | 0 |
|  | Publication bias | 0 | -1^⑤^ | -1^⑤^ | -1^⑤^ |
| 24-Hour Mean Blood Pressure | Risk of bias | -1^①^ | -1^①^ | - | -1^①^ |
|  | Inconsistency | 0 | 0 | - | 0 |
|  | Indirectness | 0 | 0 | - | 0 |
|  | Impression | 0 | 0 | - | 0 |
|  | Publication bias | 0 | -1^⑤^ | -1^⑤^ | -1^⑤^ |
| Mean Daytime Blood Pressure | Risk of bias | -1^①^ | -1^①^ | - | -1^①^ |
|  | Inconsistency | 0 | 0 | - | 0 |
|  | Indirectness | 0 | 0 | - | 0 |
|  | Impression | 0 | -1^④^ | 0 | 0 |
|  | Publication bias | 0 | -1^⑤^ | -1^⑤^ | -1^⑤^ |
| Mean Nighttime Blood Pressure | Risk of bias | -1^①^ | -1^①^ | - | -1^①^ |
|  | Inconsistency | 0 | 0 | - | 0 |
|  | Indirectness | 0 | 0 | - | 0 |
|  | Impression | 0 | 0 | - | 0 |
|  | Publication bias | 0 | -1^⑤^ | -1^⑤^ | -1^⑤^ |
| Apnea Hypopnea Index | Risk of bias | -1^①^ | -1^①^ | - | -1^①^ |
|  | Inconsistency | -1^②^ | -1^②^ | - | -1^②^ |
|  | Indirectness | 0 | 0 | - | 0 |
|  | Impression | 0 | -1^④^ | 0 | 0 |
|  | Publication bias | 0 | -1^⑤^ | -1^⑤^ | -1^⑤^ |
| Percentage of Total Sleep Time with Oxygen Saturation ＜90% | Risk of bias | -1^①^ | -1^①^ | - | -1^①^ |
|  | Inconsistency | 0 | 0 | - | 0 |
|  | Indirectness | 0 | 0 | - | 0 |
|  | Impression | 0 | 0 | - | 0 |
|  | Publication bias | 0 | -1^⑤^ | -1^⑤^ | -1^⑤^ |

Stuy 38：Montesi SB, Edwards BA, Malhotra A, Bakker JP. The effect of continuous positive airway pressure treatment on blood pressure: a systematic review and meta-analysis of randomized controlled trials. J Clin Sleep Med. 2012 Oct 15;8(5):587-96. doi: 10.5664/jcsm.2170. PMID: 23066375; PMCID: PMC3459209.

| Endpoint measure | Downgrading factor | A | B | C | D |
| --- | --- | --- | --- | --- | --- |

| Diurnal Systolic Blood Pressure | Risk of bias | -1^①^ | -1^①^ | - | -1^①^ |
| --- | --- | --- | --- | --- | --- |
|  | Inconsistency | 0 | 0 | - | 0 |
|  | Indirectness | 0 | 0 | - | 0 |
|  | Impression | 0 | 0 | - | 0 |
|  | Publication bias | 0 | -1^⑤^ | 0 | 0 |
| Diurnal Diastolic Blood Pressure | Risk of bias | -1^①^ | -1^①^ | - | -1^①^ |
|  | Inconsistency | 0 | 0 | - | 0 |
|  | Indirectness | 0 | 0 | - | 0 |
|  | Impression | 0 | 0 | - | 0 |
|  | Publication bias | 0 | -1^⑤^ | 0 | 0 |
| Nocturnal Systolic Blood Pressure | Risk of bias | -1^①^ | -1^①^ | - | -1^①^ |
|  | Inconsistency | 0 | 0 | - | 0 |
|  | Indirectness | 0 | 0 | - | 0 |
|  | Impression | 0 | 0 | - | 0 |
|  | Publication bias | 0 | -1^⑤^ | 0 | 0 |
| Nocturnal Diastolic Blood Pressure | Risk of bias | -1^①^ | -1^①^ | - | -1^①^ |
|  | Inconsistency | 0 | 0 | - | 0 |
|  | Indirectness | 0 | 0 | - | 0 |
|  | Impression | 0 | -1^④^ | -1^④^ | -1^④^ |
|  | Publication bias | 0 | -1^⑤^ | 0 | 0 |

Stuy 39：尹富禹.(2013).持续气道正压通气治疗对阻塞性睡眠呼吸暂停综合症患者血压影响的Meta分析(硕士学位论文,重庆医科大学).硕士https://kns.cnki.net/kcms2/article/abstract?v=4J3GFaKSuTPmLh0unht5Lbt4gn_kiAOM7gC0DseJPGSCqPEhA6t2QxzmaUk2fmfH-SiGw3kleN6lcGDMUlu0v22PL-jYY8Jc8p2P6pDv1BhxHfwlN6mdbnVeBWYKNsd0Z2AZ8M15hvkirOnT7EtdHfuxkVKxnHD9tr---8XnMV0XlEeM2XcP5Q==&uniplatform=NZKPT&language=CHS

| Endpoint measure | Downgrading factor | A | B | C | D |
| --- | --- | --- | --- | --- | --- |

| Systolic Blood Pressure | Risk of bias | -1^①^ | -1^①^ | - | -1^①^ |
| --- | --- | --- | --- | --- | --- |
|  | Inconsistency | 0 | 0 | - | 0 |
|  | Indirectness | 0 | 0 | - | 0 |
|  | Impression | 0 | 0 | - | 0 |
|  | Publication bias | 0 | -1^⑤^ | 0 | 0 |
| Diastolic Blood Pressure | Risk of bias | -1^①^ | -1^①^ | - | -1^①^ |
|  | Inconsistency | 0 | 0 | - | 0 |
|  | Indirectness | 0 | 0 | - | 0 |
|  | Impression | 0 | 0 | - | 0 |
|  | Publication bias | 0 | -1^⑤^ | 0 | 0 |
| 24-hour Ambulatory Systolic Blood Pressure | Risk of bias | -1^①^ | -1^①^ | - | -1^①^ |
|  | Inconsistency | 0 | 0 | - | 0 |
|  | Indirectness | 0 | 0 | - | 0 |
|  | Impression | 0 | 0 | - | 0 |
|  | Publication bias | 0 | -1^⑤^ | 0 | 0 |
| 24-hour Ambulatory Diastolic Blood Pressure | Risk of bias | -1^①^ | -1^①^ | - | -1^①^ |
|  | Inconsistency | 0 | 0 | - | 0 |
|  | Indirectness | 0 | 0 | - | 0 |
|  | Impression | 0 | 0 | - | 0 |
|  | Publication bias | 0 | -1^⑤^ | 0 | 0 |
| Systolic Blood Pressure in Hypertensive Patients | Risk of bias | -1^①^ | -1^①^ | - | -1^①^ |
|  | Inconsistency | 0 | 0 | - | 0 |
|  | Indirectness | 0 | 0 | - | 0 |
|  | Impression | 0 | -1^④^ | -1^④^ | -1^④^ |
|  | Publication bias | 0 | -1^⑤^ | 0 | 0 |
| Diastolic Blood Pressure in Hypertensive Patients | Risk of bias | -1^①^ | -1^①^ | - | -1^①^ |
|  | Inconsistency | 0 | 0 | - | 0 |
|  | Indirectness | 0 | 0 | - | 0 |
|  | Impression | 0 | -1^④^ | -1^④^ | -1^④^ |
|  | Publication bias | 0 | -1^⑤^ | 0 | 0 |

Stuy 40：符翠萍,朱芬,刘子龙,励雯静,吴晓丹,吴旭... & 李善群.(2014).持续气道正压通气对阻塞性睡眠呼吸暂停低通气综合征患者血压影响的荟萃分析.世界临床药物,35(06),358-363.https://doi.org/10.13683/j.wph.2014.06.015.

| Endpoint measure | Downgrading factor | A | B | C | D |
| --- | --- | --- | --- | --- | --- |

| 24-hour Mean Systolic Blood Pressure | Risk of bias | -1^①^ | -1^①^ | - | -1^①^ |
| --- | --- | --- | --- | --- | --- |
|  | Inconsistency | -1^②^ | -1^②^ | - | -1^②^ |
|  | Indirectness | 0 | 0 | - | 0 |
|  | Impression | 0 | -1^④^ | 0 | 0 |
|  | Publication bias | 0 | -1^⑤^ | 0 | 0 |
| 24-hour Mean Diastolic Blood Pressure | Risk of bias | -1^①^ | -1^①^ | - | -1^①^ |
|  | Inconsistency | -1^②^ | -1^②^ | - | -1^②^ |
|  | Indirectness | 0 | 0 | - | 0 |
|  | Impression | 0 | -1^④^ | -1^④^ | -1^④^ |
|  | Publication bias | 0 | -1^⑤^ | 0 | 0 |
| 24-hour Mean Arterial Pressure | Risk of bias | -1^①^ | -1^①^ | - | -1^①^ |
|  | Inconsistency | -1^②^ | -1^②^ | - | -1^②^ |
|  | Indirectness | 0 | 0 | - | 0 |
|  | Impression | 0 | -1^④^ | -1^④^ | -1^④^ |
|  | Publication bias | 0 | -1^⑤^ | 0 | 0 |

Stuy 41：Schein AS, Kerkhoff AC, Coronel CC, Plentz RD, Sbruzzi G. Continuous positive airway pressure reduces blood pressure in patients with obstructive sleep apnea; a systematic review and meta-analysis with 1000 patients. J Hypertens. 2014 Sep;32(9):1762-73. doi: 10.1097/HJH.0000000000000250. PMID: 24979300.

| Endpoint measure | Downgrading factor | A | B | C | D |
| --- | --- | --- | --- | --- | --- |

| Office systolic blood pressure | Risk of bias | -1^①^ | -1^①^ | - | -1^①^ |
| --- | --- | --- | --- | --- | --- |
|  | Inconsistency | 0 | 0 | - | 0 |
|  | Indirectness | 0 | 0 | - | 0 |
|  | Impression | 0 | 0 | - | 0 |
|  | Publication bias | 0 | -1^⑤^ | 0 | 0 |
| Office diastolic blood pressure | Risk of bias | -1^①^ | -1^①^ | - | -1^①^ |
|  | Inconsistency | -1^②^ | -1^②^ | - | -1^②^ |
|  | Indirectness | 0 | 0 | - | 0 |
|  | Impression | -1^⑤^ | -1^⑤^ | - | -1^⑤^ |
|  | Publication bias | 0 | -1 | 0 | 0 |
| 24-hour systolic blood pressure | Risk of bias | -1^①^ | -1^①^ | - | -1^①^ |
|  | Inconsistency | -1^②^ | -1^②^ | - | -1^②^ |
|  | Indirectness | 0 | 0 | - | 0 |
|  | Impression | 0 | -1^④^ | -1^④^ | -1^④^ |
|  | Publication bias | 0 | -1^⑤^ | 0 | 0 |
| 24-hour diastolic blood pressure | Risk of bias | -1^①^ | -1^①^ | - | -1^①^ |
|  | Inconsistency | 0 | -1^②^ | -1^②^ | -1^②^ |
|  | Indirectness | 0 | 0 | - | 0 |
|  | Impression | -1^④^ | -1^④^ | - | -1^④^ |
|  | Publication bias | 0 | -1^⑤^ | 0 | 0 |
| 24-hour mean arterial pressure | Risk of bias | -1^①^ | -1^①^ | - | -1^①^ |
|  | Inconsistency | -1^②^ | -1^②^ | - | -1^②^ |
|  | Indirectness | 0 | 0 | - | 0 |
|  | Impression | -1^④^ | -1^④^ | - | -1^④^ |
|  | Publication bias | 0 | -1^⑤^ | 0 | 0 |
| Daytime systolic blood pressure | Risk of bias | -1^①^ | -1^①^ | - | -1^①^ |
|  | Inconsistency | 0 | 0 | - | 0 |
|  | Indirectness | 0 | 0 | - | 0 |
|  | Impression | -1^④^ | -1^④^ | - | -1^④^ |
|  | Publication bias | 0 | -1^⑤^ | 0 | 0 |
| Daytime diastolic blood pressure | Risk of bias | -1^①^ | -1^①^ | - | -1^①^ |
|  | Inconsistency | -1^②^ | -1^②^ | - | -1^②^ |
|  | Indirectness | 0 | 0 | - | 0 |
|  | Impression | -1^④^ | -1^④^ | - | -1^④^ |
|  | Publication bias | 0 | -1^⑤^ | 0 | 0 |
| Night-time systolic blood pressure | Risk of bias | -1^①^ | -1^①^ | - | -1^①^ |
|  | Inconsistency | -1^②^ | -1^②^ | - | -1^②^ |
|  | Indirectness | 0 | 0 | - | 0 |
|  | Impression | 0 | -1^④^ | -1^④^ | -1^④^ |
|  | Publication bias | 0 | -1^⑤^ | -1^⑤^ | -1^⑤^ |
| Night-time diastolic blood pressure | Risk of bias | -1^①^ | -1^①^ | - | -1^①^ |
|  | Inconsistency | -1^②^ | -1^②^ | - | -1^②^ |
|  | Indirectness | 0 | 0 | - | 0 |
|  | Impression | -1^④^ | -1^④^ | - | -1^④^ |
|  | Publication bias | 0 | -1^⑤^ | 0 | 0 |
| Night-time mean arterial pressure | Risk of bias | -1^①^ | -1^①^ | - | -1^①^ |
|  | Inconsistency | 0 | 0 | - | 0 |
|  | Indirectness | 0 | 0 | - | 0 |
|  | Impression | 0 | 0 | - | 0 |
|  | Publication bias | 0 | -1^⑤^ | 0 | 0 |
| Daytime mean arterial pressure | Risk of bias | -1^①^ | -1^①^ | - | -1^①^ |
|  | Inconsistency | -1^②^ | -1^②^ | - | -1^②^ |
|  | Indirectness | 0 | 0 | - | 0 |
|  | Impression | -1^④^ | -1^④^ | - | -1^④^ |
|  | Publication bias | 0 | -1^⑤^ | 0 | 0 |

Stuy 42：李君,李晓艳,蒋学俊,万为国 & 刘万里.(2015).持续气道正压通气对阻塞性睡眠呼吸暂停综合征患者降压疗效的Meta分析.海南医学,26(06),909-914.

| Endpoint measure | Downgrading factor | A | B | C | D |
| --- | --- | --- | --- | --- | --- |

| Daytime systolic blood pressure change | Risk of bias | -1^①^ | -1^①^ | - | -1^①^ |
| --- | --- | --- | --- | --- | --- |
|  | Inconsistency | -1^②^ | -1^②^ | - | -1^②^ |
|  | Indirectness | 0 | 0 | - | 0 |
|  | Impression | 0 | -1^④^ | 0 | 0 |
|  | Publication bias | 0 | -1^⑤^ | 0 | 0 |
| Daytime diastolic blood pressure change | Risk of bias | -1^①^ | -1^①^ | - | -1^①^ |
|  | Inconsistency | -1^②^ | -1^②^ | - | -1^②^ |
|  | Indirectness | 0 | 0 | - | 0 |
|  | Impression | 0 | -1^④^ | 0 | 0 |
|  | Publication bias | 0 | -1^⑤^ | 0 | 0 |
| Night-time systolic blood pressure change | Risk of bias | -1^①^ | -1^①^ | - | -1^①^ |
|  | Inconsistency | -1^②^ | -1^②^ | - | -1^②^ |
|  | Indirectness | 0 | 0 | - | 0 |
|  | Impression | 0 | -1^④^ | 0 | 0 |
|  | Publication bias | 0 | -1^⑤^ | 0 | 0 |
| Night-time diastolic blood pressure change | Risk of bias | -1^①^ | -1^①^ | - | -1^①^ |
|  | Inconsistency | -1^②^ | -1^②^ | - | -1^②^ |
|  | Indirectness | 0 | 0 | - | 0 |
|  | Impression | 0 | -1^④^ | 0 | 0 |
|  | Publication bias | 0 | -1^⑤^ | 0 | 0 |
| 24-hour mean systolic blood pressure change | Risk of bias | -1^①^ | -1^①^ | - | -1^①^ |
|  | Inconsistency | -1^②^ | -1^②^ | - | -1^②^ |
|  | Indirectness | 0 | 0 | - | 0 |
|  | Impression | 0 | -1^④^ | 0 | 0 |
|  | Publication bias | 0 | -1^⑤^ | 0 | 0 |
| 24-hour mean diastolic blood pressure change | Risk of bias | -1^①^ | -1^①^ | - | -1^①^ |
|  | Inconsistency | -1^②^ | -1^②^ | - | -1^②^ |
|  | Indirectness | 0 | 0 | - | 0 |
|  | Impression | 0 | -1^④^ | 0 | 0 |
|  | Publication bias | 0 | -1^⑤^ | 0 | 0 |

Stuy 43：Hu X, Fan J, Chen S, Yin Y, Zrenner B. The role of continuous positive airway pressure in blood pressure control for patients with obstructive sleep apnea and hypertension: a meta-analysis of randomized controlled trials. J Clin Hypertens (Greenwich). 2015 Mar;17(3):215-22. doi: 10.1111/jch.12472. Epub 2015 Jan 13. PMID: 25582849; PMCID: PMC8032019.

| Endpoint measure | Downgrading factor | A | B | C | D |
| --- | --- | --- | --- | --- | --- |

| 24-hour ambulatory systolic blood pressure (SBP) net change | Risk of bias | 0 | 0 | - | 0 |
| --- | --- | --- | --- | --- | --- |
|  | Inconsistency | 0 | 0 | - | 0 |
|  | Indirectness | 0 | 0 | - | 0 |
|  | Impression | 0 | 0 | - | 0 |
|  | Publication bias | 0 | 0 | - | 0 |
| 24-hour ambulatory diastolic blood pressure (DBP) net change | Risk of bias | 0 | 0 | - | 0 |
|  | Inconsistency | 0 | 0 | - | 0 |
|  | Indirectness | 0 | 0 | - | 0 |
|  | Impression | 0 | 0 | - | 0 |
|  | Publication bias | 0 | 0 | - | 0 |
| Diurnal systolic blood pressure (SBP) net change | Risk of bias | -1^①^ | 0 | -1^①^ | -1^①^ |
|  | Inconsistency | -1^②^ | -1^②^ | - | -1^②^ |
|  | Indirectness | 0 | 0 | - | 0 |
|  | Impression | -1^④^ | -1^④^ | - | -1^④^ |
|  | Publication bias | 0 | 0 | - | 0 |
| Diurnal diastolic blood pressure (DBP) net change | Risk of bias | 0 | 0 | - | 0 |
|  | Inconsistency | -1^②^ | -1^②^ | - | -1^②^ |
|  | Indirectness | 0 | 0 | - | 0 |
|  | Impression | 0 | -1^④^ | -1^④^ | -1^④^ |
|  | Publication bias | 0 | 0 | - | 0 |
| Nocturnal systolic blood pressure (SBP) net change | Risk of bias | 0 | 0 | - | 0 |
|  | Inconsistency | 0 | 0 | - | 0 |
|  | Indirectness | 0 | 0 | - | 0 |
|  | Impression | 0 | 0 | - | 0 |
|  | Publication bias | 0 | 0 | - | 0 |
| Nocturnal diastolic blood pressure (DBP) net change | Risk of bias | 0 | 0 | - | 0 |
|  | Inconsistency | 0 | 0 | - | 0 |
|  | Indirectness | 0 | 0 | - | 0 |
|  | Impression | 0 | 0 | - | 0 |
|  | Publication bias | 0 | 0 | - | 0 |

Stuy 44：Liu L, Cao Q, Guo Z, Dai Q. Continuous Positive Airway Pressure in Patients With Obstructive Sleep Apnea and Resistant Hypertension: A Meta-Analysis of Randomized Controlled Trials. J Clin Hypertens (Greenwich). 2016 Feb;18(2):153-8. doi: 10.1111/jch.12639. Epub 2015 Aug 17. PMID: 26278919; PMCID: PMC8031627.

| Endpoint measure | Downgrading factor | A | B | C | D |
| --- | --- | --- | --- | --- | --- |

| 24-hour ambulatory systolic blood pressure (SBP) net change | Risk of bias | -1^①^ | -1^①^ | - | -1^①^ |
| --- | --- | --- | --- | --- | --- |
|  | Inconsistency | 0 | 0 | - | 0 |
|  | Indirectness | 0 | 0 | - | 0 |
|  | Impression | 0 | -1^④^ | -1^④^ | -1^④^ |
|  | Publication bias | 0 | 0 | - | 0 |
| 24-hour ambulatory diastolic blood pressure (DBP) net change | Risk of bias | -1^①^ | -1^①^ | - | -1^①^ |
|  | Inconsistency | 0 | 0 | - | 0 |
|  | Indirectness | 0 | 0 | - | 0 |
|  | Impression | 0 | -1^④^ | -1^④^ | -1^④^ |
|  | Publication bias | 0 | 0 | - | 0 |
| Daytime systolic blood pressure (SBP) net change | Risk of bias | -1^①^ | -1^①^ | - | -1^①^ |
|  | Inconsistency | -1^②^ | -1^②^ | - | -1^②^ |
|  | Indirectness | 0 | 0 | - | 0 |
|  | Impression | -1^④^ | -1^④^ | - | -1^④^ |
|  | Publication bias | 0 | 0 | - | 0 |
| Daytime diastolic blood pressure (DBP) net change | Risk of bias | -1^①^ | -1^①^ | - | -1^①^ |
|  | Inconsistency | -1^②^ | -1^②^ | - | -1^②^ |
|  | Indirectness | 0 | 0 | - | 0 |
|  | Impression | -1^④^ | -1^④^ | - | -1^④^ |
|  | Publication bias | 0 | 0 | - | 0 |
| Nighttime systolic blood pressure (SBP) net change | Risk of bias | -1^①^ | -1^①^ | - | -1^①^ |
|  | Inconsistency | -1^②^ | -1^②^ | - | -1^②^ |
|  | Indirectness | 0 | 0 | - | 0 |
|  | Impression | -1^④^ | -1^④^ | - | -1^④^ |
|  | Publication bias | 0 | 0 | - | 0 |
| Nighttime diastolic blood pressure (DBP) net change | Risk of bias | -1^①^ | -1^①^ | - | -1^①^ |
|  | Inconsistency | 0 | 0 | - | 0 |
|  | Indirectness | 0 | 0 | - | 0 |
|  | Impression | 0 | -1^④^ | -1^④^ | -1^④^ |
|  | Publication bias | 0 | 0 | - | 0 |

Stuy 45：Sun Y, Huang ZY, Sun QR, Qiu LP, Zhou TT, Zhou GH. CPAP therapy reduces blood pressure for patients with obstructive sleep apnoea: an update meta-analysis of randomized clinical trials. Acta Cardiol. 2016 Jun;71(3):275-80. doi: 10.2143/AC.71.3.3152087. PMID: 27594122.

| Endpoint measure | Downgrading factor | A | B | C | D |
| --- | --- | --- | --- | --- | --- |

| 24-hour ambulatory systolic blood pressure (SBP) net change | Risk of bias | 0 | -1^①^ | -1^①^ | -1^①^ |
| --- | --- | --- | --- | --- | --- |
|  | Inconsistency | 0 | 0 | - | 0 |
|  | Indirectness | 0 | 0 | - | 0 |
|  | Impression | 0 | 0 | - | 0 |
|  | Publication bias | 0 | -1^⑤^ | 0 | 0 |
| 24-hour ambulatory diastolic blood pressure (DBP) net change | Risk of bias | 0 | -1^①^ | -1^①^ | -1^①^ |
|  | Inconsistency | 0 | 0 | - | 0 |
|  | Indirectness | 0 | 0 | - | 0 |
|  | Impression | 0 | 0 | - | 0 |
|  | Publication bias | 0 | -1^⑤^ | 0 | 0 |
| Daytime systolic blood pressure (SBP) net change | Risk of bias | -1^①^ | -1^①^ | - | -1^①^ |
|  | Inconsistency | -1^②^ | -1^②^ | - | -1^②^ |
|  | Indirectness | 0 | 0 | - | 0 |
|  | Impression | -1^④^ | -1^④^ | - | -1^④^ |
|  | Publication bias | 0 | -1^⑤^ | 0 | 0 |
| Daytime diastolic blood pressure (DBP) net change | Risk of bias | 0 | -1^①^ | -1^①^ | -1^①^ |
|  | Inconsistency | 0 | 0 | - | 0 |
|  | Indirectness | 0 | 0 | - | 0 |
|  | Impression | 0 | -1^④^ | 0 | 0 |
|  | Publication bias | 0 | -1^⑤^ | 0 | 0 |
| Nighttime systolic blood pressure (SBP) net change | Risk of bias | 0 | -1^①^ | -1^①^ | -1^①^ |
|  | Inconsistency | 0 | 0 | - | 0 |
|  | Indirectness | 0 | 0 | - | 0 |
|  | Impression | 0 | -1^④^ | 0 | 0 |
|  | Publication bias | 0 | -1^⑤^ | 0 | 0 |
| Nighttime diastolic blood pressure (DBP) net change | Risk of bias | 0 | -1^①^ | -1^①^ | -1^①^ |
|  | Inconsistency | 0 | 0 | - | 0 |
|  | Indirectness | 0 | 0 | - | 0 |
|  | Impression | 0 | -1^④^ | 0 | 0 |
|  | Publication bias | 0 | -1^⑤^ | 0 | 0 |
| Clinic systolic blood pressure (SBP) net change | Risk of bias | 0 | -1^①^ | -1^①^ | -1^①^ |
|  | Inconsistency | -1^②^ | 0 | 0 | 0 |
|  | Indirectness | 0 | 0 | - | 0 |
|  | Impression | 0 | -1^④^ | 0 | 0 |
|  | Publication bias | 0 | -1^⑤^ | 0 | 0 |
| Clinic diastolic blood pressure (DBP) net change | Risk of bias | 0 | -1^①^ | -1^①^ | -1^①^ |
|  | Inconsistency | -1^②^ | -1^②^ | - | -1^②^ |
|  | Indirectness | 0 | 0 | - | 0 |
|  | Impression | 0 | -1^④^ | 0 | 0 |
|  | Publication bias | 0 | -1^⑤^ | 0 | 0 |
| Risk of cardiovascular events | Risk of bias | -1^①^ | -1^①^ | - | -1^①^ |
|  | Inconsistency | 0 | 0 | - | 0 |
|  | Indirectness | 0 | 0 | - | 0 |
|  | Impression | 0 | -1^④^ | -1^④^ | -1^④^ |
|  | Publication bias | 0 | -1^⑤^ | 0 | 0 |

Stuy 46：Labarca G, Schmidt A, Dreyse J, Jorquera J, Enos D, Torres G, Barbe F. Efficacy of continuous positive airway pressure (CPAP) in patients with obstructive sleep apnea (OSA) and resistant hypertension (RH): Systematic review and meta-analysis. Sleep Med Rev. 2021 Aug;58:101446. doi: 10.1016/j.smrv.2021.101446. Epub 2021 Jan 28. PMID: 33607443.

| Endpoint measure | Downgrading factor | A | B | C | D |
| --- | --- | --- | --- | --- | --- |

| Net change of 24-hour ambulatory systolic blood pressure (24-h SBP) | Risk of bias | -1^①^ | -1^①^ | - | -1^①^ |
| --- | --- | --- | --- | --- | --- |
|  | Inconsistency | -1^②^ | -1^②^ | - | -1^②^ |
|  | Indirectness | 0 | 0 | - | 0 |
|  | Impression | -1^④^ | -1^④^ | - | -1^④^ |
|  | Publication bias | -1^⑤^ | -1^⑤^ | - | -1^⑤^ |
| Net change of 24-hour ambulatory diastolic blood pressure (24-h DBP) | Risk of bias | -1^①^ | -1^①^ | - | -1^①^ |
|  | Inconsistency | -1^②^ | -1^②^ | - | -1^②^ |
|  | Indirectness | 0 | 0 | - | 0 |
|  | Impression | -1^④^ | -1^④^ | - | -1^④^ |
|  | Publication bias | -1^⑤^ | -1^⑤^ | - | -1^⑤^ |
| Net change of daytime systolic blood pressure (Daytime SBP) | Risk of bias | -1^①^ | -1^①^ | - | -1^①^ |
|  | Inconsistency | -1^②^ | -1^②^ | - | -1^②^ |
|  | Indirectness | 0 | 0 | - | 0 |
|  | Impression | -1^④^ | -1^④^ | - | -1^④^ |
|  | Publication bias | 0 | -1^⑤^ | -1^⑤^ | -1^⑤^ |
| Net change of daytime diastolic blood pressure (Daytime DBP) | Risk of bias | -1^①^ | -1^①^ | - | -1^①^ |
|  | Inconsistency | -1^②^ | -1^②^ | - | -1^②^ |
|  | Indirectness | 0 | 0 | - | 0 |
|  | Impression | -1^④^ | -1^④^ | - | -1^④^ |
|  | Publication bias | -1^⑤^ | -1^⑤^ | - | -1^⑤^ |
| Net change of nighttime systolic blood pressure (Nighttime SBP) | Risk of bias | -1^①^ | -1^①^ | - | -1^①^ |
|  | Inconsistency | -1^②^ | 0 | -1^②^ | -1^②^ |
|  | Indirectness | 0 | 0 | - | 0 |
|  | Impression | -1^④^ | -1^④^ | - | -1^④^ |
|  | Publication bias | 0 | 0 | - | 0 |
| Net change of nighttime diastolic blood pressure (Nighttime DBP) | Risk of bias | -1^①^ | -1^①^ | - | -1^①^ |
|  | Inconsistency | -1^②^ | 0 | -1^②^ | -1^②^ |
|  | Indirectness | 0 | 0 | - | 0 |
|  | Impression | 0 | -1^④^ | -1^④^ | -1^④^ |
|  | Publication bias | -1^⑤^ | 0 | -1^⑤^ | -1^⑤^ |
| Change in aortic stiffness | Risk of bias | -1^①^ | -1^①^ | - | -1^①^ |
|  | Inconsistency | 0 | 0 | - | 0 |
|  | Indirectness | 0 | -1^③^ | 0 | 0 |
|  | Impression | -1^④^ | -1^④^ | - | -1^④^ |
|  | Publication bias | 0 | -1^⑤^ | 0 | 0 |

Stuy 47：Shang W, Zhang Y, Liu L, Chen F, Wang G, Han D. Benefits of continuous positive airway pressure on blood pressure in patients with hypertension and obstructive sleep apnea: a meta-analysis. Hypertens Res. 2022 Nov;45(11):1802-1813. doi: 10.1038/s41440-022-00954-9. Epub 2022 Jun 14. PMID: 35701490.

| Endpoint measure | Downgrading factor | A | B | C | D |
| --- | --- | --- | --- | --- | --- |

| 24-hour systolic blood pressure (24-h SBP) | Risk of bias | -1^①^ | -1^①^ | - | -1^①^ |
| --- | --- | --- | --- | --- | --- |
|  | Inconsistency | -1^②^ | -1^②^ | - | -1^②^ |
|  | Indirectness | 0 | 0 | - | 0 |
|  | Impression | 0 | 0 | - | 0 |
|  | Publication bias | 0 | 0 | - | 0 |
| 24-hour diastolic blood pressure (24-h DBP) | Risk of bias | -1^①^ | -1^①^ | - | -1^①^ |
|  | Inconsistency | -1^②^ | -1^②^ | - | -1^②^ |
|  | Indirectness | 0 | 0 | - | 0 |
|  | Impression | 0 | 0 | - | 0 |
|  | Publication bias | 0 | 0 | - | 0 |
| Daytime systolic blood pressure (Day-SBP) | Risk of bias | -1^①^ | -1^①^ | - | -1^①^ |
|  | Inconsistency | -1^②^ | -1^②^ | - | -1^②^ |
|  | Indirectness | 0 | 0 | - | 0 |
|  | Impression | 0 | 0 | - | 0 |
|  | Publication bias | 0 | 0 | - | 0 |
| Daytime diastolic blood pressure (Day-DBP) | Risk of bias | -1^①^ | -1^①^ | - | -1^①^ |
|  | Inconsistency | -1^②^ | -1^②^ | - | -1^②^ |
|  | Indirectness | 0 | 0 | - | 0 |
|  | Impression | 0 | 0 | - | 0 |
|  | Publication bias | 0 | 0 | - | 0 |
| Nighttime systolic blood pressure (Night-SBP) | Risk of bias | -1^①^ | -1^①^ | - | -1^①^ |
|  | Inconsistency | -1^②^ | -1^②^ | - | -1^②^ |
|  | Indirectness | 0 | 0 | - | 0 |
|  | Impression | 0 | 0 | - | 0 |
|  | Publication bias | 0 | 0 | - | 0 |
| Nighttime diastolic blood pressure (Night-DBP) | Risk of bias | -1^①^ | -1^①^ | - | -1^①^ |
|  | Inconsistency | -1^②^ | -1^②^ | - | -1^②^ |
|  | Indirectness | 0 | 0 | - | 0 |
|  | Impression | 0 | 0 | - | 0 |
|  | Publication bias | 0 | 0 | - | 0 |
| Office systolic blood pressure (Office-SBP) | Risk of bias | -1^①^ | -1^①^ | - | -1^①^ |
|  | Inconsistency | 0 | 0 | - | 0 |
|  | Indirectness | 0 | 0 | - | 0 |
|  | Impression | 0 | 0 | - | 0 |
|  | Publication bias | 0 | 0 | - | 0 |
| Office diastolic blood pressure (Office-DBP) | Risk of bias | -1^①^ | -1^①^ | - | -1^①^ |
|  | Inconsistency | 0 | 0 | - | 0 |
|  | Indirectness | 0 | 0 | - | 0 |
|  | Impression | 0 | 0 | - | 0 |
|  | Publication bias | 0 | 0 | - | 0 |
| Heart rate (HR) | Risk of bias | -1^①^ | -1^①^ | - | -1^①^ |
|  | Inconsistency | 0 | 0 | - | 0 |
|  | Indirectness | 0 | 0 | - | 0 |
|  | Impression | 0 | 0 | - | 0 |
|  | Publication bias | 0 | 0 | - | 0 |

Stuy 48：Benning L, Herzig JJ, Mollet MS, Bradicich M, Pengo MF, Ulrich S, Schwarz EI. Effects of CPAP on Blood Pressure Parameter Across Different Severities of Obstructive Sleep Apnoea: A Meta-Analysis. J Sleep Res. 2025 Oct;34(5):e70072. doi: 10.1111/jsr.70072. Epub 2025 Apr 20. PMID: 40254559.

| Endpoint measure | Downgrading factor | A | B | C | D |
| --- | --- | --- | --- | --- | --- |

| Office systolic blood pressure (Office SBP) | Risk of bias | -1^①^ | -1^①^ | - | -1^①^ |
| --- | --- | --- | --- | --- | --- |
|  | Inconsistency | -1^②^ | -1^②^ | - | -1^②^ |
|  | Indirectness | 0 | 0 | - | 0 |
|  | Impression | 0 | 0 | - | 0 |
|  | Publication bias | 0 | 0 | - | 0 |
| Office diastolic blood pressure (Office DBP) | Risk of bias | -1^①^ | -1^①^ | - | -1^①^ |
|  | Inconsistency | -1^②^ | -1^②^ | - | -1^②^ |
|  | Indirectness | 0 | 0 | - | 0 |
|  | Impression | 0 | 0 | - | 0 |
|  | Publication bias | 0 | 0 | - | 0 |
| 24-hour systolic blood pressure (24-h SBP) | Risk of bias | -1^①^ | -1^①^ | - | -1^①^ |
|  | Inconsistency | 0 | -1^②^ | -1^②^ | -1^②^ |
|  | Indirectness | 0 | 0 | - | 0 |
|  | Impression | 0 | 0 | - | 0 |
|  | Publication bias | 0 | 0 | - | 0 |
| 24-hour diastolic blood pressure (24-h DBP) | Risk of bias | -1^①^ | -1^①^ | - | -1^①^ |
|  | Inconsistency | 0 | -1^②^ | -1^②^ | -1^②^ |
|  | Indirectness | 0 | 0 | - | 0 |
|  | Impression | 0 | 0 | - | 0 |
|  | Publication bias | 0 | 0 | - | 0 |
| Diurnal systolic blood pressure (Diurnal SBP) | Risk of bias | -1^①^ | -1^①^ | - | -1^①^ |
|  | Inconsistency | -1^②^ | -1^②^ | - | -1^②^ |
|  | Indirectness | 0 | 0 | - | 0 |
|  | Impression | 0 | 0 | - | 0 |
|  | Publication bias | 0 | 0 | - | 0 |
| Diurnal diastolic blood pressure (Diurnal DBP) | Risk of bias | -1^①^ | -1^①^ | - | -1^①^ |
|  | Inconsistency | -1^②^ | -1^②^ | - | -1^②^ |
|  | Indirectness | 0 | 0 | - | 0 |
|  | Impression | 0 | 0 | - | 0 |
|  | Publication bias | 0 | 0 | - | 0 |
| Nocturnal systolic blood pressure (Nocturnal SBP) | Risk of bias | -1^①^ | -1^①^ | - | -1^①^ |
|  | Inconsistency | 0 | -1^②^ | -1^②^ | -1^②^ |
|  | Indirectness | 0 | 0 | - | 0 |
|  | Impression | 0 | 0 | - | 0 |
|  | Publication bias | 0 | 0 | - | 0 |
| Nocturnal diastolic blood pressure (Nocturnal DBP) | Risk of bias | -1^①^ | -1^①^ | - | -1^①^ |
|  | Inconsistency | 0 | -1^②^ | -1^②^ | -1^②^ |
|  | Indirectness | 0 | 0 | - | 0 |
|  | Impression | 0 | 0 | - | 0 |
|  | Publication bias | 0 | 0 | - | 0 |

AMSTAR-2

Y: Yes; N: No; PY: Partial Yes.

A:The conclusions of researcher Ph.D. Jingjing Liu.

B:The conclusions of researcher Ph.D. Yan Cui.

C:In case of a difference of opinions, it shall be adjudicated by Professor Yongshi Liu.

D:Conclusive conclusion.

Study 33：莫莉 & 何权瀛.(2007).长期持续气道正压通气对阻塞性睡眠呼吸暂停低通气综合征患者血压影响的荟萃分析.中华医学杂志,87(17),1177-1180.

| Entry | | A | B | C | D |
| --- | --- | --- | --- | --- | --- |
| 1 | Did the research questions and inclusion criteria for the review include the components of PICO? | Y | Y | - | Y |
| 2 | Did the report of the review contain an explicit statement that the review methods were established prior to the conduct of the review and did the report justify any significantdeviations from the protocol? | N | N | - | N |
| 3 | Did the review authors explain their selection of the study designs for inclusion in the review? | Y | Y | - | Y |
| 4 | Did the review authors use a comprehensive literature search strategy? | PY | PY | - | PY |
| 5 | Did the review authors perform study selection in duplicate? | N | Y | Y | Y |
| 6 | Did the review authors perform data extraction in duplicate? | N | Y | Y | Y |
| 7 | Did the review authors provide a list of excluded studies and justify the exclusions? | PY | N | N | N |
| 8 | Did the review authors describe the included studies in adequate detail? | PY | Y | Y | Y |
| 9 | Did the review authors use a satisfactory technique for assessing the risk of bias (RoB) in individual studies that were included in the review? | PY | N | N | N |
| 10 | Did the review authors report on the sources of funding for the studies included in the review? | N | N | - | N |
| 11 | If meta-analysis was performed, did the review authors use appropriate methods for statistical combination of results? | Y | Y | - | Y |
| 12 | If meta-analysis was performed, did the review authors assess the potential impact of RoB in individual studies on the results of the meta-analysis or other evidence synthesis? | N | N | - | N |
| 13 | Did the review authors account for RoB in primary studies when interpreting/discussing the results of the review? | N | N | - | N |
| 14 | Did the review authors provide a satisfactory explanation for, and discussion of, any heterogeneity observed in the results of the review? | PY | PY | - | PY |
| 15 | If they performed quantitative synthesis did the review authors carry out an adequate investigation of publication bias (small study bias) and discuss its likely impact on the results of the review? | PY | Y | PY | PY |
| 16 | Did the review authors report any potential sources of conflict of interest, including any funding they received for conducting the review? | N | N | - | N |

Study 34：Bazzano L.A., Khan Z., Reynolds K., He J. (2007). Effect of nocturnal nasal continuous positive airway pressure on blood pressure in obstructive sleep apnea. Hypertension, 50(2), 417-423. http://dx.doi.org/10.1161/HYPERTENSIONAHA.106.085175

| Entry | | A | B | C | D |
| --- | --- | --- | --- | --- | --- |
| 1 | Did the research questions and inclusion criteria for the review include the components of PICO? | Y | Y | - | Y |
| 2 | Did the report of the review contain an explicit statement that the review methods were established prior to the conduct of the review and did the report justify any significantdeviations from the protocol? | N | N | - | N |
| 3 | Did the review authors explain their selection of the study designs for inclusion in the review? | Y | PY | PY | PY |
| 4 | Did the review authors use a comprehensive literature search strategy? | PY | PY | - | PY |
| 5 | Did the review authors perform study selection in duplicate? | Y | Y | - | Y |
| 6 | Did the review authors perform data extraction in duplicate? | N | Y | N | N |
| 7 | Did the review authors provide a list of excluded studies and justify the exclusions? | PY | N | PY | PY |
| 8 | Did the review authors describe the included studies in adequate detail? | Y | Y | - | Y |
| 9 | Did the review authors use a satisfactory technique for assessing the risk of bias (RoB) in individual studies that were included in the review? | N | PY | N | N |
| 10 | Did the review authors report on the sources of funding for the studies included in the review? | N | N | - | N |
| 11 | If meta-analysis was performed, did the review authors use appropriate methods for statistical combination of results? | Y | Y | - | Y |
| 12 | If meta-analysis was performed, did the review authors assess the potential impact of RoB in individual studies on the results of the meta-analysis or other evidence synthesis? | N | N | - | N |
| 13 | Did the review authors account for RoB in primary studies when interpreting/discussing the results of the review? | N | PY | N | N |
| 14 | Did the review authors provide a satisfactory explanation for, and discussion of, any heterogeneity observed in the results of the review? | PY | Y | Y | Y |
| 15 | If they performed quantitative synthesis did the review authors carry out an adequate investigation of publication bias (small study bias) and discuss its likely impact on the results of the review? | N | PY | PY | PY |
| 16 | Did the review authors report any potential sources of conflict of interest, including any funding they received for conducting the review? | Y | Y | - | Y |

Study 35：Alajmi M, Mulgrew AT, Fox J, Davidson W, Schulzer M, Mak E, Ryan CF, Fleetham J, Choi P, Ayas NT. Impact of continuous positive airway pressure therapy on blood pressure in patients with obstructive sleep apnea hypopnea: a meta-analysis of randomized controlled trials. Lung. 2007 Mar-Apr;185(2):67-72. doi: 10.1007/s00408-006-0117-x. Epub 2007 Mar 28. PMID: 17393240.

| Entry | | A | B | C | D |
| --- | --- | --- | --- | --- | --- |
| 1 | Did the research questions and inclusion criteria for the review include the components of PICO? | Y | Y | - | Y |
| 2 | Did the report of the review contain an explicit statement that the review methods were established prior to the conduct of the review and did the report justify any significantdeviations from the protocol? | N | N | - | N |
| 3 | Did the review authors explain their selection of the study designs for inclusion in the review? | Y | PY | PY | PY |
| 4 | Did the review authors use a comprehensive literature search strategy? | PY | PY | - | PY |
| 5 | Did the review authors perform study selection in duplicate? | Y | Y | - | Y |
| 6 | Did the review authors perform data extraction in duplicate? | Y | Y | - | Y |
| 7 | Did the review authors provide a list of excluded studies and justify the exclusions? | PY | N | Y | Y |
| 8 | Did the review authors describe the included studies in adequate detail? | Y | Y | - | Y |
| 9 | Did the review authors use a satisfactory technique for assessing the risk of bias (RoB) in individual studies that were included in the review? | N | PY | N | N |
| 10 | Did the review authors report on the sources of funding for the studies included in the review? | N | N | - | N |
| 11 | If meta-analysis was performed, did the review authors use appropriate methods for statistical combination of results? | Y | Y | - | Y |
| 12 | If meta-analysis was performed, did the review authors assess the potential impact of RoB in individual studies on the results of the meta-analysis or other evidence synthesis? | N | N | - | N |
| 13 | Did the review authors account for RoB in primary studies when interpreting/discussing the results of the review? | N | PY | N | N |
| 14 | Did the review authors provide a satisfactory explanation for, and discussion of, any heterogeneity observed in the results of the review? | PY | Y | Y | Y |
| 15 | If they performed quantitative synthesis did the review authors carry out an adequate investigation of publication bias (small study bias) and discuss its likely impact on the results of the review? | Y | Y | - | Y |
| 16 | Did the review authors report any potential sources of conflict of interest, including any funding they received for conducting the review? | PY | Y | Y | Y |

Study 36：Haentjens P., Van Meerhaeghe A., Moscariello A., De Weerdt S., Poppe K., Dupont A., Velkeniers B. (2007). The impact of continuous positive airway pressure on blood pressure in patients with obstructive sleep apnea syndrome: Evidence from a meta-analysis of placebo-controlled randomized trials. Archives of Internal Medicine, 167(8), 757-765. http://dx.doi.org/10.1001/archinte.167.8.757

| Entry | | A | B | C | D |
| --- | --- | --- | --- | --- | --- |
| 1 | Did the research questions and inclusion criteria for the review include the components of PICO? | Y | Y | - | Y |
| 2 | Did the report of the review contain an explicit statement that the review methods were established prior to the conduct of the review and did the report justify any significantdeviations from the protocol? | N | N | - | N |
| 3 | Did the review authors explain their selection of the study designs for inclusion in the review? | Y | PY | PY | PY |
| 4 | Did the review authors use a comprehensive literature search strategy? | PY | PY | - | PY |
| 5 | Did the review authors perform study selection in duplicate? | N | Y | N | N |
| 6 | Did the review authors perform data extraction in duplicate? | Y | Y | - | Y |
| 7 | Did the review authors provide a list of excluded studies and justify the exclusions? | Y | Y | - | Y |
| 8 | Did the review authors describe the included studies in adequate detail? | Y | Y | - | Y |
| 9 | Did the review authors use a satisfactory technique for assessing the risk of bias (RoB) in individual studies that were included in the review? | N | PY | N | N |
| 10 | Did the review authors report on the sources of funding for the studies included in the review? | N | N | - | N |
| 11 | If meta-analysis was performed, did the review authors use appropriate methods for statistical combination of results? | Y | Y | - | Y |
| 12 | If meta-analysis was performed, did the review authors assess the potential impact of RoB in individual studies on the results of the meta-analysis or other evidence synthesis? | N | N | - | N |
| 13 | Did the review authors account for RoB in primary studies when interpreting/discussing the results of the review? | N | PY | N | N |
| 14 | Did the review authors provide a satisfactory explanation for, and discussion of, any heterogeneity observed in the results of the review? | Y | Y | - | Y |
| 15 | If they performed quantitative synthesis did the review authors carry out an adequate investigation of publication bias (small study bias) and discuss its likely impact on the results of the review? | Y | Y | - | Y |
| 16 | Did the review authors report any potential sources of conflict of interest, including any funding they received for conducting the review? | Y | Y | - | Y |

Study 37：王馨平.(2008).持续正压气道通气对阻塞性睡眠呼吸暂停低通气综合征中重度患者24小时动态血压影响的有效性评价(硕士学位论文,兰州大学).硕士https://kns.cnki.net/kcms2/article/abstract?v=9jT59j8Ji06nE4qyuijwTxro1nn4sKFzXfUXT9i1VmdWAeVIlWc11wPPQkxQv7rjvDTMjL6abWjLORwxIxfjQSf00UEFImXxhxZh2y9uZOuWYb1g0HeecOiawN0gY8_PKrWea6GL7VVWoIxHkXzJgVkm_i5v5KU6JvNEKox8me0kYqH0ECdvEQ==&uniplatform=NZKPT&language=CHS

| Entry | | A | B | C | D |
| --- | --- | --- | --- | --- | --- |
| 1 | Did the research questions and inclusion criteria for the review include the components of PICO? | Y | Y | - | Y |
| 2 | Did the report of the review contain an explicit statement that the review methods were established prior to the conduct of the review and did the report justify any significantdeviations from the protocol? | N | N | - | N |
| 3 | Did the review authors explain their selection of the study designs for inclusion in the review? | Y | PY | Y | Y |
| 4 | Did the review authors use a comprehensive literature search strategy? | Y | Y | - | Y |
| 5 | Did the review authors perform study selection in duplicate? | Y | Y | - | Y |
| 6 | Did the review authors perform data extraction in duplicate? | Y | Y | - | Y |
| 7 | Did the review authors provide a list of excluded studies and justify the exclusions? | Y | PY | Y | Y |
| 8 | Did the review authors describe the included studies in adequate detail? | Y | Y | - | Y |
| 9 | Did the review authors use a satisfactory technique for assessing the risk of bias (RoB) in individual studies that were included in the review? | Y | PY | Y | Y |
| 10 | Did the review authors report on the sources of funding for the studies included in the review? | N | N | - | N |
| 11 | If meta-analysis was performed, did the review authors use appropriate methods for statistical combination of results? | Y | Y | - | Y |
| 12 | If meta-analysis was performed, did the review authors assess the potential impact of RoB in individual studies on the results of the meta-analysis or other evidence synthesis? | PY | PY | - | PY |
| 13 | Did the review authors account for RoB in primary studies when interpreting/discussing the results of the review? | Y | Y | - | Y |
| 14 | Did the review authors provide a satisfactory explanation for, and discussion of, any heterogeneity observed in the results of the review? | Y | PY | Y | Y |
| 15 | If they performed quantitative synthesis did the review authors carry out an adequate investigation of publication bias (small study bias) and discuss its likely impact on the results of the review? | N | N | - | N |
| 16 | Did the review authors report any potential sources of conflict of interest, including any funding they received for conducting the review? | N | N | - | N |

Study 38：Montesi SB, Edwards BA, Malhotra A, Bakker JP. The effect of continuous positive airway pressure treatment on blood pressure: a systematic review and meta-analysis of randomized controlled trials. J Clin Sleep Med. 2012 Oct 15;8(5):587-96. doi: 10.5664/jcsm.2170. PMID: 23066375; PMCID: PMC3459209.

| Entry | | A | B | C | D |
| --- | --- | --- | --- | --- | --- |
| 1 | Did the research questions and inclusion criteria for the review include the components of PICO? | Y | Y | - | Y |
| 2 | Did the report of the review contain an explicit statement that the review methods were established prior to the conduct of the review and did the report justify any significantdeviations from the protocol? | N | N | - | N |
| 3 | Did the review authors explain their selection of the study designs for inclusion in the review? | Y | Y | - | Y |
| 4 | Did the review authors use a comprehensive literature search strategy? | PY | Y | PY | PY |
| 5 | Did the review authors perform study selection in duplicate? | Y | Y | - | Y |
| 6 | Did the review authors perform data extraction in duplicate? | Y | Y | - | Y |
| 7 | Did the review authors provide a list of excluded studies and justify the exclusions? | Y | Y | - | Y |
| 8 | Did the review authors describe the included studies in adequate detail? | Y | Y | - | Y |
| 9 | Did the review authors use a satisfactory technique for assessing the risk of bias (RoB) in individual studies that were included in the review? | N | PY | N | N |
| 10 | Did the review authors report on the sources of funding for the studies included in the review? | N | N | - | N |
| 11 | If meta-analysis was performed, did the review authors use appropriate methods for statistical combination of results? | Y | Y | - | Y |
| 12 | If meta-analysis was performed, did the review authors assess the potential impact of RoB in individual studies on the results of the meta-analysis or other evidence synthesis? | N | PY | N | N |
| 13 | Did the review authors account for RoB in primary studies when interpreting/discussing the results of the review? | N | Y | N | N |
| 14 | Did the review authors provide a satisfactory explanation for, and discussion of, any heterogeneity observed in the results of the review? | Y | Y | - | Y |
| 15 | If they performed quantitative synthesis did the review authors carry out an adequate investigation of publication bias (small study bias) and discuss its likely impact on the results of the review? | N | Y | Y | Y |
| 16 | Did the review authors report any potential sources of conflict of interest, including any funding they received for conducting the review? | Y | Y | - | Y |

Study 39：尹富禹.(2013).持续气道正压通气治疗对阻塞性睡眠呼吸暂停综合症患者血压影响的Meta分析(硕士学位论文,重庆医科大学).硕士https://kns.cnki.net/kcms2/article/abstract?v=9jT59j8Ji07GJvRqP7QjpjiDaw-jA0h1ai-tr8BYJajFP31_bUzUPzkpsv0LekFbmNdEnS8Ai1TUf9ERpkq_kR5p8J6BT5_jzFYacmrQHDZmj5LeMpXUIBckm3pym6Te10sMgxTY_B9TslOBJKqpT5QiVi8y9eCBsO2D2sXmzSTpcXWsxIhAGg==&uniplatform=NZKPT&language=CHS

| Entry | | A | B | C | D |
| --- | --- | --- | --- | --- | --- |
| 1 | Did the research questions and inclusion criteria for the review include the components of PICO? | Y | Y | - | Y |
| 2 | Did the report of the review contain an explicit statement that the review methods were established prior to the conduct of the review and did the report justify any significantdeviations from the protocol? | N | N | - | Y |
| 3 | Did the review authors explain their selection of the study designs for inclusion in the review? | Y | Y | - | Y |
| 4 | Did the review authors use a comprehensive literature search strategy? | PY | PY | - | Y |
| 5 | Did the review authors perform study selection in duplicate? | Y | Y | - | Y |
| 6 | Did the review authors perform data extraction in duplicate? | Y | Y | Y | Y |
| 7 | Did the review authors provide a list of excluded studies and justify the exclusions? | PY | N | PY | PY |
| 8 | Did the review authors describe the included studies in adequate detail? | PY | Y | - | Y |
| 9 | Did the review authors use a satisfactory technique for assessing the risk of bias (RoB) in individual studies that were included in the review? | N | N | - | Y |
| 10 | Did the review authors report on the sources of funding for the studies included in the review? | N | N | - | N |
| 11 | If meta-analysis was performed, did the review authors use appropriate methods for statistical combination of results? | Y | Y | - | Y |
| 12 | If meta-analysis was performed, did the review authors assess the potential impact of RoB in individual studies on the results of the meta-analysis or other evidence synthesis? | N | N | - | PY |
| 13 | Did the review authors account for RoB in primary studies when interpreting/discussing the results of the review? | N | N | - | PY |
| 14 | Did the review authors provide a satisfactory explanation for, and discussion of, any heterogeneity observed in the results of the review? | PY | PY | - | PY |
| 15 | If they performed quantitative synthesis did the review authors carry out an adequate investigation of publication bias (small study bias) and discuss its likely impact on the results of the review? | PY | Y | - | N |
| 16 | Did the review authors report any potential sources of conflict of interest, including any funding they received for conducting the review? | N | N | - | Y |

Study 40：符翠萍,朱芬,刘子龙,励雯静,吴晓丹,吴旭... & 李善群.(2014).持续气道正压通气对阻塞性睡眠呼吸暂停低通气综合征患者血压影响的荟萃分析.世界临床药物,35(06),358-363.https://doi.org/10.13683/j.wph.2014.06.015.

| Entry | | A | B | C | D |
| --- | --- | --- | --- | --- | --- |
| 1 | Did the research questions and inclusion criteria for the review include the components of PICO? | Y | Y | - | Y |
| 2 | Did the report of the review contain an explicit statement that the review methods were established prior to the conduct of the review and did the report justify any significantdeviations from the protocol? | N | N | - | N |
| 3 | Did the review authors explain their selection of the study designs for inclusion in the review? | Y | Y | - | Y |
| 4 | Did the review authors use a comprehensive literature search strategy? | Y | Y | - | Y |
| 5 | Did the review authors perform study selection in duplicate? | PY | Y | N | N |
| 6 | Did the review authors perform data extraction in duplicate? | N | Y | N | N |
| 7 | Did the review authors provide a list of excluded studies and justify the exclusions? | PY | Y | PY | PY |
| 8 | Did the review authors describe the included studies in adequate detail? | Y | N | Y | Y |
| 9 | Did the review authors use a satisfactory technique for assessing the risk of bias (RoB) in individual studies that were included in the review? | N | PY | Y | Y |
| 10 | Did the review authors report on the sources of funding for the studies included in the review? | N | N | - | N |
| 11 | If meta-analysis was performed, did the review authors use appropriate methods for statistical combination of results? | Y | Y | - | Y |
| 12 | If meta-analysis was performed, did the review authors assess the potential impact of RoB in individual studies on the results of the meta-analysis or other evidence synthesis? | N | PY | PY | PY |
| 13 | Did the review authors account for RoB in primary studies when interpreting/discussing the results of the review? | N | PY | PY | PY |
| 14 | Did the review authors provide a satisfactory explanation for, and discussion of, any heterogeneity observed in the results of the review? | PY | Y | Y | Y |
| 15 | If they performed quantitative synthesis did the review authors carry out an adequate investigation of publication bias (small study bias) and discuss its likely impact on the results of the review? | PY | PY | - | PY |
| 16 | Did the review authors report any potential sources of conflict of interest, including any funding they received for conducting the review? | PY | Y | Y | Y |

Study 41：Schein A.S.O., Kerkhoff A.C., Coronel C.C., Plentz R.D.M., Sbruzzi G. (2014). Continuous positive airway pressure reduces blood pressure in patients with obstructive sleep apnea; A systematic review and meta-analysis with 1000 patients. Journal of Hypertension, 32(9), 1762-1773. http://dx.doi.org/10.1097/HJH.0000000000000250

| Entry | | A | B | C | D |
| --- | --- | --- | --- | --- | --- |
| 1 | Did the research questions and inclusion criteria for the review include the components of PICO? | Y | Y | - | Y |
| 2 | Did the report of the review contain an explicit statement that the review methods were established prior to the conduct of the review and did the report justify any significantdeviations from the protocol? | PY | Y | Y | Y |
| 3 | Did the review authors explain their selection of the study designs for inclusion in the review? | Y | Y | - | Y |
| 4 | Did the review authors use a comprehensive literature search strategy? | Y | Y | - | Y |
| 5 | Did the review authors perform study selection in duplicate? | Y | Y | - | Y |
| 6 | Did the review authors perform data extraction in duplicate? | Y | Y | - | Y |
| 7 | Did the review authors provide a list of excluded studies and justify the exclusions? | PY | N | N | N |
| 8 | Did the review authors describe the included studies in adequate detail? | Y | Y | - | Y |
| 9 | Did the review authors use a satisfactory technique for assessing the risk of bias (RoB) in individual studies that were included in the review? | Y | PY | Y | Y |
| 10 | Did the review authors report on the sources of funding for the studies included in the review? | N | N | - | N |
| 11 | If meta-analysis was performed, did the review authors use appropriate methods for statistical combination of results? | Y | Y | - | Y |
| 12 | If meta-analysis was performed, did the review authors assess the potential impact of RoB in individual studies on the results of the meta-analysis or other evidence synthesis? | N | PY | N | N |
| 13 | Did the review authors account for RoB in primary studies when interpreting/discussing the results of the review? | PY | Y | Y | Y |
| 14 | Did the review authors provide a satisfactory explanation for, and discussion of, any heterogeneity observed in the results of the review? | Y | Y | - | Y |
| 15 | If they performed quantitative synthesis did the review authors carry out an adequate investigation of publication bias (small study bias) and discuss its likely impact on the results of the review? | N | N | - | N |
| 16 | Did the review authors report any potential sources of conflict of interest, including any funding they received for conducting the review? | PY | Y | Y | Y |

Study 42：李君,李晓艳,蒋学俊,万为国 & 刘万里.(2015).持续气道正压通气对阻塞性睡眠呼吸暂停综合征患者降压疗效的Meta分析.海南医学,26(06),909-914.

| Entry | | A | B | C | D |
| --- | --- | --- | --- | --- | --- |
| 1 | Did the research questions and inclusion criteria for the review include the components of PICO? | Y | Y | - | Y |
| 2 | Did the report of the review contain an explicit statement that the review methods were established prior to the conduct of the review and did the report justify any significantdeviations from the protocol? | N | N | - | N |
| 3 | Did the review authors explain their selection of the study designs for inclusion in the review? | Y | Y | - | Y |
| 4 | Did the review authors use a comprehensive literature search strategy? | Y | PY | PY | PY |
| 5 | Did the review authors perform study selection in duplicate? | Y | Y | - | Y |
| 6 | Did the review authors perform data extraction in duplicate? | Y | Y | - | Y |
| 7 | Did the review authors provide a list of excluded studies and justify the exclusions? | PY | PY | - | PY |
| 8 | Did the review authors describe the included studies in adequate detail? | Y | Y | - | Y |
| 9 | Did the review authors use a satisfactory technique for assessing the risk of bias (RoB) in individual studies that were included in the review? | PY | PY | - | PY |
| 10 | Did the review authors report on the sources of funding for the studies included in the review? | N | N | - | N |
| 11 | If meta-analysis was performed, did the review authors use appropriate methods for statistical combination of results? | Y | Y | - | Y |
| 12 | If meta-analysis was performed, did the review authors assess the potential impact of RoB in individual studies on the results of the meta-analysis or other evidence synthesis? | N | PY | N | N |
| 13 | Did the review authors account for RoB in primary studies when interpreting/discussing the results of the review? | N | PY | N | N |
| 14 | Did the review authors provide a satisfactory explanation for, and discussion of, any heterogeneity observed in the results of the review? | PY | PY | - | PY |
| 15 | If they performed quantitative synthesis did the review authors carry out an adequate investigation of publication bias (small study bias) and discuss its likely impact on the results of the review? | PY | PY | - | PY |
| 16 | Did the review authors report any potential sources of conflict of interest, including any funding they received for conducting the review? | N | N | - | N |

Study 43：Hu X., Fan J., Chen S., Yin Y., Zrenner B. (2015). The Role of Continuous Positive Airway Pressure in Blood Pressure Control for Patients With Obstructive Sleep Apnea and Hypertension: A Meta-Analysis of Randomized Controlled Trials. Journal of Clinical Hypertension, 17(3), 215-222. http://dx.doi.org/10.1111/jch.12472

| Entry | | A | B | C | D |
| --- | --- | --- | --- | --- | --- |
| 1 | Did the research questions and inclusion criteria for the review include the components of PICO? | Y | Y | - | Y |
| 2 | Did the report of the review contain an explicit statement that the review methods were established prior to the conduct of the review and did the report justify any significantdeviations from the protocol? | N | N | - | N |
| 3 | Did the review authors explain their selection of the study designs for inclusion in the review? | Y | Y | - | Y |
| 4 | Did the review authors use a comprehensive literature search strategy? | PY | PY | - | PY |
| 5 | Did the review authors perform study selection in duplicate? | Y | Y | - | Y |
| 6 | Did the review authors perform data extraction in duplicate? | Y | Y | - | Y |
| 7 | Did the review authors provide a list of excluded studies and justify the exclusions? | PY | PY | - | PY |
| 8 | Did the review authors describe the included studies in adequate detail? | Y | Y | - | Y |
| 9 | Did the review authors use a satisfactory technique for assessing the risk of bias (RoB) in individual studies that were included in the review? | PY | PY | - | PY |
| 10 | Did the review authors report on the sources of funding for the studies included in the review? | N | N | - | N |
| 11 | If meta-analysis was performed, did the review authors use appropriate methods for statistical combination of results? | Y | Y | - | Y |
| 12 | If meta-analysis was performed, did the review authors assess the potential impact of RoB in individual studies on the results of the meta-analysis or other evidence synthesis? | N | PY | PY | PY |
| 13 | Did the review authors account for RoB in primary studies when interpreting/discussing the results of the review? | N | PY | N | N |
| 14 | Did the review authors provide a satisfactory explanation for, and discussion of, any heterogeneity observed in the results of the review? | Y | Y | - | Y |
| 15 | If they performed quantitative synthesis did the review authors carry out an adequate investigation of publication bias (small study bias) and discuss its likely impact on the results of the review? | PY | PY | - | PY |
| 16 | Did the review authors report any potential sources of conflict of interest, including any funding they received for conducting the review? | Y | Y | - | Y |

Study 44：Liu L., Cao Q., Guo Z., Dai Q. (2016). Continuous Positive Airway Pressure in Patients With Obstructive Sleep Apnea and Resistant Hypertension: A Meta-Analysis of Randomized Controlled Trials. Journal of Clinical Hypertension, 18(2), 153-158. http://dx.doi.org/10.1111/jch.12639

| Entry | | A | B | C | D |
| --- | --- | --- | --- | --- | --- |
| 1 | Did the research questions and inclusion criteria for the review include the components of PICO? | Y | Y | - | Y |
| 2 | Did the report of the review contain an explicit statement that the review methods were established prior to the conduct of the review and did the report justify any significantdeviations from the protocol? | N | N | - | N |
| 3 | Did the review authors explain their selection of the study designs for inclusion in the review? | Y | Y | - | Y |
| 4 | Did the review authors use a comprehensive literature search strategy? | PY | PY | - | PY |
| 5 | Did the review authors perform study selection in duplicate? | Y | Y | - | Y |
| 6 | Did the review authors perform data extraction in duplicate? | Y | Y | - | Y |
| 7 | Did the review authors provide a list of excluded studies and justify the exclusions? | PY | PY | - | PY |
| 8 | Did the review authors describe the included studies in adequate detail? | Y | Y | - | Y |
| 9 | Did the review authors use a satisfactory technique for assessing the risk of bias (RoB) in individual studies that were included in the review? | PY | PY | - | PY |
| 10 | Did the review authors report on the sources of funding for the studies included in the review? | N | N | - | N |
| 11 | If meta-analysis was performed, did the review authors use appropriate methods for statistical combination of results? | Y | Y | - | Y |
| 12 | If meta-analysis was performed, did the review authors assess the potential impact of RoB in individual studies on the results of the meta-analysis or other evidence synthesis? | N | PY | N | N |
| 13 | Did the review authors account for RoB in primary studies when interpreting/discussing the results of the review? | N | PY | N | N |
| 14 | Did the review authors provide a satisfactory explanation for, and discussion of, any heterogeneity observed in the results of the review? | PY | Y | Y | Y |
| 15 | If they performed quantitative synthesis did the review authors carry out an adequate investigation of publication bias (small study bias) and discuss its likely impact on the results of the review? | PY | PY | - | PY |
| 16 | Did the review authors report any potential sources of conflict of interest, including any funding they received for conducting the review? | PY | Y | Y | Y |

Study 45：Sun Y., Huang Z.-Y., Sun Q.-R., Qiu L.-P., Zhou T.-T., Zhou G.-H. (2016). CPAP therapy reduces blood pressure for patients with obstructive sleep apnoea: An update meta-analysis of randomized clinical trials. Acta Cardiologica, 71(3), 275-280. http://dx.doi.org/10.2143/AC.71.3.3152087

| Entry | | A | B | C | D |
| --- | --- | --- | --- | --- | --- |
| 1 | Did the research questions and inclusion criteria for the review include the components of PICO? | Y | Y | - | Y |
| 2 | Did the report of the review contain an explicit statement that the review methods were established prior to the conduct of the review and did the report justify any significantdeviations from the protocol? | N | N | - | N |
| 3 | Did the review authors explain their selection of the study designs for inclusion in the review? | Y | Y | - | Y |
| 4 | Did the review authors use a comprehensive literature search strategy? | PY | PY | - | PY |
| 5 | Did the review authors perform study selection in duplicate? | N | Y | Y | Y |
| 6 | Did the review authors perform data extraction in duplicate? | Y | Y | - | Y |
| 7 | Did the review authors provide a list of excluded studies and justify the exclusions? | PY | N | PY | PY |
| 8 | Did the review authors describe the included studies in adequate detail? | PY | Y | Y | Y |
| 9 | Did the review authors use a satisfactory technique for assessing the risk of bias (RoB) in individual studies that were included in the review? | PY | PY | - | PY |
| 10 | Did the review authors report on the sources of funding for the studies included in the review? | N | N | - | N |
| 11 | If meta-analysis was performed, did the review authors use appropriate methods for statistical combination of results? | Y | Y | - | Y |
| 12 | If meta-analysis was performed, did the review authors assess the potential impact of RoB in individual studies on the results of the meta-analysis or other evidence synthesis? | N | N | - | N |
| 13 | Did the review authors account for RoB in primary studies when interpreting/discussing the results of the review? | N | PY | PY | PY |
| 14 | Did the review authors provide a satisfactory explanation for, and discussion of, any heterogeneity observed in the results of the review? | N | PY | Y | Y |
| 15 | If they performed quantitative synthesis did the review authors carry out an adequate investigation of publication bias (small study bias) and discuss its likely impact on the results of the review? | N | N | - | N |
| 16 | Did the review authors report any potential sources of conflict of interest, including any funding they received for conducting the review? | PY | Y | Y | Y |

Study 46：Labarca G., Schmidt A., Dreyse J., Jorquera J., Enos D., Torres G., Barbe F. (2021). Efficacy of continuous positive airway pressure (CPAP) in patients with obstructive sleep apnea (OSA) and resistant hypertension (RH): Systematic review and meta-analysis. Sleep Medicine Reviews, 58. http://dx.doi.org/10.1016/j.smrv.2021.101446

| Entry | | A | B | C | D |
| --- | --- | --- | --- | --- | --- |
| 1 | Did the research questions and inclusion criteria for the review include the components of PICO? | Y | Y | - | Y |
| 2 | Did the report of the review contain an explicit statement that the review methods were established prior to the conduct of the review and did the report justify any significantdeviations from the protocol? | Y | Y | - | Y |
| 3 | Did the review authors explain their selection of the study designs for inclusion in the review? | Y | Y | - | Y |
| 4 | Did the review authors use a comprehensive literature search strategy? | Y | Y | - | Y |
| 5 | Did the review authors perform study selection in duplicate? | Y | Y | - | Y |
| 6 | Did the review authors perform data extraction in duplicate? | Y | Y | - | Y |
| 7 | Did the review authors provide a list of excluded studies and justify the exclusions? | PY | Y | Y | Y |
| 8 | Did the review authors describe the included studies in adequate detail? | Y | Y | - | Y |
| 9 | Did the review authors use a satisfactory technique for assessing the risk of bias (RoB) in individual studies that were included in the review? | Y | Y | - | Y |
| 10 | Did the review authors report on the sources of funding for the studies included in the review? | N | N | - | N |
| 11 | If meta-analysis was performed, did the review authors use appropriate methods for statistical combination of results? | Y | Y | - | Y |
| 12 | If meta-analysis was performed, did the review authors assess the potential impact of RoB in individual studies on the results of the meta-analysis or other evidence synthesis? | Y | Y | - | Y |
| 13 | Did the review authors account for RoB in primary studies when interpreting/discussing the results of the review? | Y | Y | - | Y |
| 14 | Did the review authors provide a satisfactory explanation for, and discussion of, any heterogeneity observed in the results of the review? | Y | Y | - | Y |
| 15 | If they performed quantitative synthesis did the review authors carry out an adequate investigation of publication bias (small study bias) and discuss its likely impact on the results of the review? | PY | PY | - | PY |
| 16 | Did the review authors report any potential sources of conflict of interest, including any funding they received for conducting the review? | Y | Y | - | Y |

Study 47：Shang W., Zhang Y., Liu L., Chen F., Wang G., Han D. (2022). Benefits of continuous positive airway pressure on blood pressure in patients with hypertension and obstructive sleep apnea: a meta-analysis. Hypertension Research, 45(11), 1802-1813. http://dx.doi.org/10.1038/s41440-022-00954-9

| Entry | | A | B | C | D |
| --- | --- | --- | --- | --- | --- |
| 1 | Did the research questions and inclusion criteria for the review include the components of PICO? | Y | Y | - | Y |
| 2 | Did the report of the review contain an explicit statement that the review methods were established prior to the conduct of the review and did the report justify any significantdeviations from the protocol? | Y | Y | - | Y |
| 3 | Did the review authors explain their selection of the study designs for inclusion in the review? | Y | Y | - | Y |
| 4 | Did the review authors use a comprehensive literature search strategy? | Y | Y | - | Y |
| 5 | Did the review authors perform study selection in duplicate? | Y | Y | - | Y |
| 6 | Did the review authors perform data extraction in duplicate? | Y | Y | - | Y |
| 7 | Did the review authors provide a list of excluded studies and justify the exclusions? | PY | Y | Y | Y |
| 8 | Did the review authors describe the included studies in adequate detail? | Y | Y | - | Y |
| 9 | Did the review authors use a satisfactory technique for assessing the risk of bias (RoB) in individual studies that were included in the review? | N | PY | PY | PY |
| 10 | Did the review authors report on the sources of funding for the studies included in the review? | N | N | - | N |
| 11 | If meta-analysis was performed, did the review authors use appropriate methods for statistical combination of results? | Y | Y | - | Y |
| 12 | If meta-analysis was performed, did the review authors assess the potential impact of RoB in individual studies on the results of the meta-analysis or other evidence synthesis? | N | PY | N | N |
| 13 | Did the review authors account for RoB in primary studies when interpreting/discussing the results of the review? | N | PY | N | N |
| 14 | Did the review authors provide a satisfactory explanation for, and discussion of, any heterogeneity observed in the results of the review? | PY | Y | Y | Y |
| 15 | If they performed quantitative synthesis did the review authors carry out an adequate investigation of publication bias (small study bias) and discuss its likely impact on the results of the review? | PY | PY | - | PY |
| 16 | Did the review authors report any potential sources of conflict of interest, including any funding they received for conducting the review? | PY | Y | Y | Y |

Study 48：Benning L., Herzig J.J., Mollet M.S., Bradicich M., Pengo M.F., Ulrich S., Schwarz E.I. (2025). Effects of CPAP on Blood Pressure Parameter Across Different Severities of Obstructive Sleep Apnoea: A Meta-Analysis. Journal of Sleep Research, 34(5). http://dx.doi.org/10.1111/jsr.70072

| Entry | | A | B | C | D |
| --- | --- | --- | --- | --- | --- |
| 1 | Did the research questions and inclusion criteria for the review include the components of PICO? | Y | Y | - | Y |
| 2 | Did the report of the review contain an explicit statement that the review methods were established prior to the conduct of the review and did the report justify any significantdeviations from the protocol? | PY | Y | Y | Y |
| 3 | Did the review authors explain their selection of the study designs for inclusion in the review? | Y | Y | - | Y |
| 4 | Did the review authors use a comprehensive literature search strategy? | PY | PY | - | PY |
| 5 | Did the review authors perform study selection in duplicate? | Y | Y | - | Y |
| 6 | Did the review authors perform data extraction in duplicate? | Y | Y | - | Y |
| 7 | Did the review authors provide a list of excluded studies and justify the exclusions? | PY | Y | Y | Y |
| 8 | Did the review authors describe the included studies in adequate detail? | Y | Y | - | Y |
| 9 | Did the review authors use a satisfactory technique for assessing the risk of bias (RoB) in individual studies that were included in the review? | Y | Y | - | Y |
| 10 | Did the review authors report on the sources of funding for the studies included in the review? | N | N | - | N |
| 11 | If meta-analysis was performed, did the review authors use appropriate methods for statistical combination of results? | Y | Y | - | Y |
| 12 | If meta-analysis was performed, did the review authors assess the potential impact of RoB in individual studies on the results of the meta-analysis or other evidence synthesis? | Y | Y | - | Y |
| 13 | Did the review authors account for RoB in primary studies when interpreting/discussing the results of the review? | PY | Y | Y | Y |
| 14 | Did the review authors provide a satisfactory explanation for, and discussion of, any heterogeneity observed in the results of the review? | Y | Y | - | Y |
| 15 | If they performed quantitative synthesis did the review authors carry out an adequate investigation of publication bias (small study bias) and discuss its likely impact on the results of the review? | PY | Y | Y | Y |
| 16 | Did the review authors report any potential sources of conflict of interest, including any funding they received for conducting the review? | Y | Y | - | Y |

PRISMA 2020

Y: Yes; N: No; PY: Partial Yes.

A:The conclusions of researcher Ph.D. Jingjing Liu.

B:The conclusions of researcher Ph.D. Yan Cui.

C:In case of a difference of opinions, it shall be adjudicated by Professor Yongshi Liu.

D:Conclusive conclusion.

Table 33：莫莉 & 何权瀛.(2007).长期持续气道正压通气对阻塞性睡眠呼吸暂停低通气综合征患者血压影响的荟萃分析.中华医学杂志,87(17),1177-1180.

| Section and topic | Item # | Checklist item | A | B | C | D |
| --- | --- | --- | --- | --- | --- | --- |
| **Title** | | | | | | |
| Title | 1 | Identify the report as a systematic review. | N | N | - | N |
| **Abstract** | | | | | | |
| Abstract | 2 | See the PRISMA 2020 for Abstracts checklist (table 2). | PY | N | PY | PY |
| **Introduction** | | | | | | |
| Rationale | 3 | Describe the rationale for the review in the context of existing knowledge | PY | Y | Y | Y |
| Objectives | 4 | Provide an explicit statement of the objective(s) or question(s) the review addresses. | Y | Y | - | Y |
| **Methods** | | | | | | |
| Eligibility criteria | 5 | Specify the inclusion and exclusion criteria for the review and how studies were grouped for the syntheses. | PY | PY | - | PY |
| Information sources | 6 | Specify all databases, registers, websites, organisations, reference lists and other sources searched or consulted to identify studies. Specify the date when each source was last searched or consulted. | PY | PY | - | PY |
| Search strategy | 7 | Present the full search strategies for all databases, registers and websites, including any filters and limits used | N | N | - | N |
| Selection process | 8 | Specify the methods used to decide whether a study met the inclusion criteria of the review, including how many reviewers screened each record and each report retrieved, whether they worked independently, and if applicable, details of automation tools  used in the process. | N | N | - | N |
| Data collection  process | 9 | Specify the methods used to collect data from reports, including how many reviewers collected data from each report, whether they worked independently, any processes for obtaining or confirming data from study investigators, and if applicable, details of automation tools used in the process. | N | N | - | N |
| Data items | 10a | List and define all outcomes for which data were sought. Specify whether all results that were compatible with each outcome domain in each study were sought (e.g. for all measures, time points, analyses), and if not, the methods used to decide which  results to collect. | PY | N | PY | PY |
|  | 10b | List and define all other variables for which data were sought (e.g. participant and intervention characteristics, funding sources). Describe any assumptions made about any missing or unclear information. | PY | N | PY | PY |
| Study risk of bias  assessment | 11 | Specify the methods used to assess risk of bias in the included studies, including details of the tool(s) used, how many reviewers assessed each study and whether they worked independently, and if applicable, details of automation tools used in the process. | N | PY | Y | Y |
| Effect measures | 12 | Specify for each outcome the effect measure(s) (e.g. risk ratio, mean difference) used in the synthesis or presentation of results. | Y | Y | - | Y |
| Synthesis methods | 13a | Describe the processes used to decide which studies were eligible for each synthesis (e.g. tabulating the study intervention characteristics and comparing against the planned groups for each synthesis (item #5)). | N | N | - | N |
|  | 13b | Describe any methods required to prepare the data for presentation or synthesis, such as handling of missing summary statistics, or data conversions. | N | N | - | N |
|  | 13c | Describe any methods used to tabulate or visually display results of individual studies and syntheses. | PY | PY | - | PY |
|  | 13d | Describe any methods used to synthesise results and provide a rationale for the choice(s). If meta-analysis was performed, describe the model(s), method(s) to identify the presence and extent of statistical heterogeneity, and software package(s) used. | Y | Y | - | Y |
|  | 13e | Describe any methods used to explore possible causes of heterogeneity among study results (e.g. subgroup analysis, meta regression). | N | N | - | N |
|  | 13f | Describe any sensitivity analyses conducted to assess robustness of the synthesised results. | N | N | - | N |
| Reporting bias  assessment | 14 | Describe any methods used to assess risk of bias due to missing results in a synthesis (arising from reporting biases). | PY | PY | - | PY |
| Certainty assessment | 15 | Describe any methods used to assess certainty (or confidence) in the body of evidence for an outcome. | N | N | - | N |
| **Results** | | | | | | |
| Study selection | 16a | Describe the results of the search and selection process, from the number of records identified in the search to the number of studies included in the review, ideally using a flow diagram (see fig 1). | PY | N | N | N |
|  | 16b | Cite studies that might appear to meet the inclusion criteria, but which were excluded, and explain why they were excluded. | N | N | N | N |
| Study characteristics | 17 | Cite each included study and present its characteristics. | Y | Y | - | Y |
| Risk of bias in studies | 18 | Present assessments of risk of bias for each included study. | N | PY | PY | PY |
| Results of individual  studies | 19 | For all outcomes, present, for each study: (a) summary statistics for each group (where appropriate) and (b) an effect estimate and its precision (e.g. confidence/credible interval), ideally using structured tables or plots. | PY | Y | Y | Y |
| Results of syntheses | 20a | For each synthesis, briefly summarise the characteristics and risk of bias among contributing studies. | PY | PY | - | PY |
|  | 20b | Present results of all statistical syntheses conducted. If meta-analysis was done, present for each the summary estimate and its precision (e.g. confidence/credible interval) and measures of statistical heterogeneity. If comparing groups, describe the direction of the effect. | Y | Y | - | Y |
|  | 20c | Present results of all investigations of possible causes of heterogeneity among study results. | N | N | - | N |
|  | 20d | Present results of all sensitivity analyses conducted to assess the robustness of the synthesised results. | N | N | - | N |
| Reporting biases | 21 | Present assessments of risk of bias due to missing results (arising from reporting biases) for each synthesis assessed. | PY | N | Y | Y |
| Certainty of evidence | 22 | Present assessments of certainty (or confidence) in the body of evidence for each outcome assessed. | N | N | - | N |
| **Discussion** | | | | | | |
| Discussion | 23a | Provide a general interpretation of the results in the context of other evidence. | PY | PY | - | PY |
|  | 23b | Discuss any limitations of the evidence included in the review. | PY | Y | Y | Y |
|  | 23c | Discuss any limitations of the review processes used. | N | PY | N | N |
|  | 23d | Discuss implications of the results for practice, policy, and future research. | N | Y | PY | PY |
| **Other information** | | | | | | |
| Registration and  protocol | 24a | Provide registration information for the review, including register name and registration number, or state that the review was not registered. | N | N | - | N |
|  | 24b | Indicate where the review protocol can be accessed, or state that a protocol was not prepared. | N | N | - | N |
|  | 24c | Describe and explain any amendments to information provided at registration or in the protocol. | N | N | - | N |
| Support | 25 | Describe sources of financial or non-financial support for the review, and the role of the funders or sponsors in the review. | N | N | - | N |
| Competing interests | 26 | Declare any competing interests of review authors. | N | N | - | N |
| Availability of data,  code, and other  materials | 27 | Report which of the following are publicly available and where they can be found: template data collection forms; data extracted from included studies; data used for all analyses; analytic code; any other materials used in the review. | N | N | - | N |

Table 34：Bazzano L.A., Khan Z., Reynolds K., He J. (2007). Effect of nocturnal nasal continuous positive airway pressure on blood pressure in obstructive sleep apnea. Hypertension, 50(2), 417-423. http://dx.doi.org/10.1161/HYPERTENSIONAHA.106.085175

| Section and topic | Item # | Checklist item | A | B | C | D |
| --- | --- | --- | --- | --- | --- | --- |
| **Title** | | | | | | |
| Title | 1 | Identify the report as a systematic review. | PY | N | Y | Y |
| **Abstract** | | | | | | |
| Abstract | 2 | See the PRISMA 2020 for Abstracts checklist (table 2). | PY | PY | Y | Y |
| **Introduction** | | | | | | |
| Rationale | 3 | Describe the rationale for the review in the context of existing knowledge | Y | Y | - | Y |
| Objectives | 4 | Provide an explicit statement of the objective(s) or question(s) the review addresses. | Y | Y | - | Y |
| **Methods** | | | | | | |
| Eligibility criteria | 5 | Specify the inclusion and exclusion criteria for the review and how studies were grouped for the syntheses. | PY | Y | Y | Y |
| Information sources | 6 | Specify all databases, registers, websites, organisations, reference lists and other sources searched or consulted to identify studies. Specify the date when each source was last searched or consulted. | PY | Y | Y | Y |
| Search strategy | 7 | Present the full search strategies for all databases, registers and websites, including any filters and limits used | N | PY | PY | PY |
| Selection process | 8 | Specify the methods used to decide whether a study met the inclusion criteria of the review, including how many reviewers screened each record and each report retrieved, whether they worked independently, and if applicable, details of automation tools  used in the process. | PY | Y | Y | Y |
| Data collection  process | 9 | Specify the methods used to collect data from reports, including how many reviewers collected data from each report, whether they worked independently, any processes for obtaining or confirming data from study investigators, and if applicable, details of automation tools used in the process. | N | PY | PY | PY |
| Data items | 10a | List and define all outcomes for which data were sought. Specify whether all results that were compatible with each outcome domain in each study were sought (e.g. for all measures, time points, analyses), and if not, the methods used to decide which  results to collect. | PY | PY | - | PY |
|  | 10b | List and define all other variables for which data were sought (e.g. participant and intervention characteristics, funding sources). Describe any assumptions made about any missing or unclear information. | PY | PY | - | PY |
| Study risk of bias  assessment | 11 | Specify the methods used to assess risk of bias in the included studies, including details of the tool(s) used, how many reviewers assessed each study and whether they worked independently, and if applicable, details of automation tools used in the process. | N | N | - | N |
| Effect measures | 12 | Specify for each outcome the effect measure(s) (e.g. risk ratio, mean difference) used in the synthesis or presentation of results. | Y | Y | - | Y |
| Synthesis methods | 13a | Describe the processes used to decide which studies were eligible for each synthesis (e.g. tabulating the study intervention characteristics and comparing against the planned groups for each synthesis (item #5)). | N | PY | N | N |
|  | 13b | Describe any methods required to prepare the data for presentation or synthesis, such as handling of missing summary statistics, or data conversions. | N | PY | N | N |
|  | 13c | Describe any methods used to tabulate or visually display results of individual studies and syntheses. | PY | PY | - | PY |
|  | 13d | Describe any methods used to synthesise results and provide a rationale for the choice(s). If meta-analysis was performed, describe the model(s), method(s) to identify the presence and extent of statistical heterogeneity, and software package(s) used. | Y | Y | - | Y |
|  | 13e | Describe any methods used to explore possible causes of heterogeneity among study results (e.g. subgroup analysis, meta regression). | Y | Y | - | Y |
|  | 13f | Describe any sensitivity analyses conducted to assess robustness of the synthesised results. | N | Y | Y | Y |
| Reporting bias  assessment | 14 | Describe any methods used to assess risk of bias due to missing results in a synthesis (arising from reporting biases). | N | N | - | N |
| Certainty assessment | 15 | Describe any methods used to assess certainty (or confidence) in the body of evidence for an outcome. | N | N | - | N |
| **Results** | | | | | | |
| Study selection | 16a | Describe the results of the search and selection process, from the number of records identified in the search to the number of studies included in the review, ideally using a flow diagram (see fig 1). | PY | N | N | N |
|  | 16b | Cite studies that might appear to meet the inclusion criteria, but which were excluded, and explain why they were excluded. | PY | PY | - | PY |
| Study characteristics | 17 | Cite each included study and present its characteristics. | Y | Y | - | Y |
| Risk of bias in studies | 18 | Present assessments of risk of bias for each included study. | N | N | - | N |
| Results of individual  studies | 19 | For all outcomes, present, for each study: (a) summary statistics for each group (where appropriate) and (b) an effect estimate and its precision (e.g. confidence/credible interval), ideally using structured tables or plots. | PY | PY | - | PY |
| Results of syntheses | 20a | For each synthesis, briefly summarise the characteristics and risk of bias among contributing studies. | PY | PY | - | PY |
|  | 20b | Present results of all statistical syntheses conducted. If meta-analysis was done, present for each the summary estimate and its precision (e.g. confidence/credible interval) and measures of statistical heterogeneity. If comparing groups, describe the direction of the effect. | Y | Y | - | Y |
|  | 20c | Present results of all investigations of possible causes of heterogeneity among study results. | Y | Y | - | Y |
|  | 20d | Present results of all sensitivity analyses conducted to assess the robustness of the synthesised results. | N | Y | Y | Y |
| Reporting biases | 21 | Present assessments of risk of bias due to missing results (arising from reporting biases) for each synthesis assessed. | N | N | - | N |
| Certainty of evidence | 22 | Present assessments of certainty (or confidence) in the body of evidence for each outcome assessed. | N | N | - | N |
| **Discussion** | | | | | | |
| Discussion | 23a | Provide a general interpretation of the results in the context of other evidence. | Y | Y | - | Y |
|  | 23b | Discuss any limitations of the evidence included in the review. | PY | Y | Y | Y |
|  | 23c | Discuss any limitations of the review processes used. | N | PY | N | N |
|  | 23d | Discuss implications of the results for practice, policy, and future research. | Y | Y | - | Y |
| **Other information** | | | | | | |
| Registration and  protocol | 24a | Provide registration information for the review, including register name and registration number, or state that the review was not registered. | N | N | - | N |
|  | 24b | Indicate where the review protocol can be accessed, or state that a protocol was not prepared. | N | N | - | N |
|  | 24c | Describe and explain any amendments to information provided at registration or in the protocol. | N | N | - | N |
| Support | 25 | Describe sources of financial or non-financial support for the review, and the role of the funders or sponsors in the review. | Y | Y | - | Y |
| Competing interests | 26 | Declare any competing interests of review authors. | Y | Y | - | Y |
| Availability of data,  code, and other  materials | 27 | Report which of the following are publicly available and where they can be found: template data collection forms; data extracted from included studies; data used for all analyses; analytic code; any other materials used in the review. | N | N | - | N |

Table 35：Alajmi M, Mulgrew AT, Fox J, Davidson W, Schulzer M, Mak E, Ryan CF, Fleetham J, Choi P, Ayas NT. Impact of continuous positive airway pressure therapy on blood pressure in patients with obstructive sleep apnea hypopnea: a meta-analysis of randomized controlled trials. Lung. 2007 Mar-Apr;185(2):67-72. doi: 10.1007/s00408-006-0117-x. Epub 2007 Mar 28. PMID: 17393240.

| Section and topic | Item # | Checklist item | A | B | C | D |
| --- | --- | --- | --- | --- | --- | --- |
| **Title** | | | | | | |
| Title | 1 | Identify the report as a systematic review. | N | N | - | N |
| **Abstract** | | | | | | |
| Abstract | 2 | See the PRISMA 2020 for Abstracts checklist (table 2). | PY | PY | - | PY |
| **Introduction** | | | | | | |
| Rationale | 3 | Describe the rationale for the review in the context of existing knowledge | Y | Y | - | Y |
| Objectives | 4 | Provide an explicit statement of the objective(s) or question(s) the review addresses. | Y | Y | - | Y |
| **Methods** | | | | | | |
| Eligibility criteria | 5 | Specify the inclusion and exclusion criteria for the review and how studies were grouped for the syntheses. | PY | Y | Y | Y |
| Information sources | 6 | Specify all databases, registers, websites, organisations, reference lists and other sources searched or consulted to identify studies. Specify the date when each source was last searched or consulted. | PY | Y | PY | PY |
| Search strategy | 7 | Present the full search strategies for all databases, registers and websites, including any filters and limits used | PY | PY | PY | PY |
| Selection process | 8 | Specify the methods used to decide whether a study met the inclusion criteria of the review, including how many reviewers screened each record and each report retrieved, whether they worked independently, and if applicable, details of automation tools  used in the process. | Y | Y | - | Y |
| Data collection  process | 9 | Specify the methods used to collect data from reports, including how many reviewers collected data from each report, whether they worked independently, any processes for obtaining or confirming data from study investigators, and if applicable, details of automation tools used in the process. | Y | Y | - | Y |
| Data items | 10a | List and define all outcomes for which data were sought. Specify whether all results that were compatible with each outcome domain in each study were sought (e.g. for all measures, time points, analyses), and if not, the methods used to decide which  results to collect. | PY | PY | - | PY |
|  | 10b | List and define all other variables for which data were sought (e.g. participant and intervention characteristics, funding sources). Describe any assumptions made about any missing or unclear information. | PY | PY | - | PY |
| Study risk of bias  assessment | 11 | Specify the methods used to assess risk of bias in the included studies, including details of the tool(s) used, how many reviewers assessed each study and whether they worked independently, and if applicable, details of automation tools used in the process. | N | N | - | N |
| Effect measures | 12 | Specify for each outcome the effect measure(s) (e.g. risk ratio, mean difference) used in the synthesis or presentation of results. | Y | Y | - | Y |
| Synthesis methods | 13a | Describe the processes used to decide which studies were eligible for each synthesis (e.g. tabulating the study intervention characteristics and comparing against the planned groups for each synthesis (item #5)). | N | PY | N | N |
|  | 13b | Describe any methods required to prepare the data for presentation or synthesis, such as handling of missing summary statistics, or data conversions. | PY | PY | N | N |
|  | 13c | Describe any methods used to tabulate or visually display results of individual studies and syntheses. | Y | Y | - | Y |
|  | 13d | Describe any methods used to synthesise results and provide a rationale for the choice(s). If meta-analysis was performed, describe the model(s), method(s) to identify the presence and extent of statistical heterogeneity, and software package(s) used. | Y | Y | - | Y |
|  | 13e | Describe any methods used to explore possible causes of heterogeneity among study results (e.g. subgroup analysis, meta regression). | Y | Y | - | Y |
|  | 13f | Describe any sensitivity analyses conducted to assess robustness of the synthesised results. | Y | Y | - | Y |
| Reporting bias  assessment | 14 | Describe any methods used to assess risk of bias due to missing results in a synthesis (arising from reporting biases). | Y | Y | - | Y |
| Certainty assessment | 15 | Describe any methods used to assess certainty (or confidence) in the body of evidence for an outcome. | N | N | - | N |
| **Results** | | | | | | |
| Study selection | 16a | Describe the results of the search and selection process, from the number of records identified in the search to the number of studies included in the review, ideally using a flow diagram (see fig 1). | Y | Y | - | Y |
|  | 16b | Cite studies that might appear to meet the inclusion criteria, but which were excluded, and explain why they were excluded. | PY | Y | Y | Y |
| Study characteristics | 17 | Cite each included study and present its characteristics. | Y | Y | - | Y |
| Risk of bias in studies | 18 | Present assessments of risk of bias for each included study. | N | N | - | N |
| Results of individual  studies | 19 | For all outcomes, present, for each study: (a) summary statistics for each group (where appropriate) and (b) an effect estimate and its precision (e.g. confidence/credible interval), ideally using structured tables or plots. | Y | Y | - | Y |
| Results of syntheses | 20a | For each synthesis, briefly summarise the characteristics and risk of bias among contributing studies. | PY | PY | - | PY |
|  | 20b | Present results of all statistical syntheses conducted. If meta-analysis was done, present for each the summary estimate and its precision (e.g. confidence/credible interval) and measures of statistical heterogeneity. If comparing groups, describe the direction of the effect. | Y | Y | - | Y |
|  | 20c | Present results of all investigations of possible causes of heterogeneity among study results. | Y | Y | - | Y |
|  | 20d | Present results of all sensitivity analyses conducted to assess the robustness of the synthesised results. | Y | Y | - | Y |
| Reporting biases | 21 | Present assessments of risk of bias due to missing results (arising from reporting biases) for each synthesis assessed. | Y | PY | Y | Y |
| Certainty of evidence | 22 | Present assessments of certainty (or confidence) in the body of evidence for each outcome assessed. | N | N | - | N |
| **Discussion** | | | | | | |
| Discussion | 23a | Provide a general interpretation of the results in the context of other evidence. | Y | Y | - | Y |
|  | 23b | Discuss any limitations of the evidence included in the review. | PY | Y | Y | Y |
|  | 23c | Discuss any limitations of the review processes used. | N | PY | N | N |
|  | 23d | Discuss implications of the results for practice, policy, and future research. | Y | Y | - | Y |
| **Other information** | | | | | | |
| Registration and  protocol | 24a | Provide registration information for the review, including register name and registration number, or state that the review was not registered. | N | N | - | N |
|  | 24b | Indicate where the review protocol can be accessed, or state that a protocol was not prepared. | N | N | - | N |
|  | 24c | Describe and explain any amendments to information provided at registration or in the protocol. | N | N | - | N |
| Support | 25 | Describe sources of financial or non-financial support for the review, and the role of the funders or sponsors in the review. | Y | Y | - | Y |
| Competing interests | 26 | Declare any competing interests of review authors. | Y | Y | - | Y |
| Availability of data,  code, and other  materials | 27 | Report which of the following are publicly available and where they can be found: template data collection forms; data extracted from included studies; data used for all analyses; analytic code; any other materials used in the review. | N | N | - | N |

Table36 ：Haentjens P., Van Meerhaeghe A., Moscariello A., De Weerdt S., Poppe K., Dupont A., Velkeniers B. (2007). The impact of continuous positive airway pressure on blood pressure in patients with obstructive sleep apnea syndrome: Evidence from a meta-analysis of placebo-controlled randomized trials. Archives of Internal Medicine, 167(8), 757-765. http://dx.doi.org/10.1001/archinte.167.8.757

| Section and topic | Item # | Checklist item | A | B | C | D |
| --- | --- | --- | --- | --- | --- | --- |
| **Title** | | | | | | |
| Title | 1 | Identify the report as a systematic review. | PY | N | Y | Y |
| **Abstract** | | | | | | |
| Abstract | 2 | See the PRISMA 2020 for Abstracts checklist (table 2). | PY | PY | - | PY |
| **Introduction** | | | | | | |
| Rationale | 3 | Describe the rationale for the review in the context of existing knowledge | Y | Y | - | Y |
| Objectives | 4 | Provide an explicit statement of the objective(s) or question(s) the review addresses. | Y | Y | - | Y |
| **Methods** | | | | | | |
| Eligibility criteria | 5 | Specify the inclusion and exclusion criteria for the review and how studies were grouped for the syntheses. | PY | Y | Y | Y |
| Information sources | 6 | Specify all databases, registers, websites, organisations, reference lists and other sources searched or consulted to identify studies. Specify the date when each source was last searched or consulted. | PY | Y | Y | Y |
| Search strategy | 7 | Present the full search strategies for all databases, registers and websites, including any filters and limits used | PY | PY | - | PY |
| Selection process | 8 | Specify the methods used to decide whether a study met the inclusion criteria of the review, including how many reviewers screened each record and each report retrieved, whether they worked independently, and if applicable, details of automation tools  used in the process. | N | Y | Y | Y |
| Data collection  process | 9 | Specify the methods used to collect data from reports, including how many reviewers collected data from each report, whether they worked independently, any processes for obtaining or confirming data from study investigators, and if applicable, details of automation tools used in the process. | PY | Y | Y | Y |
| Data items | 10a | List and define all outcomes for which data were sought. Specify whether all results that were compatible with each outcome domain in each study were sought (e.g. for all measures, time points, analyses), and if not, the methods used to decide which  results to collect. | PY | PY | - | PY |
|  | 10b | List and define all other variables for which data were sought (e.g. participant and intervention characteristics, funding sources). Describe any assumptions made about any missing or unclear information. | PY | PY | - | PY |
| Study risk of bias  assessment | 11 | Specify the methods used to assess risk of bias in the included studies, including details of the tool(s) used, how many reviewers assessed each study and whether they worked independently, and if applicable, details of automation tools used in the process. | N | N | - | N |
| Effect measures | 12 | Specify for each outcome the effect measure(s) (e.g. risk ratio, mean difference) used in the synthesis or presentation of results. | Y | Y | - | Y |
| Synthesis methods | 13a | Describe the processes used to decide which studies were eligible for each synthesis (e.g. tabulating the study intervention characteristics and comparing against the planned groups for each synthesis (item #5)). | N | PY | N | N |
|  | 13b | Describe any methods required to prepare the data for presentation or synthesis, such as handling of missing summary statistics, or data conversions. | Y | PY | Y | Y |
|  | 13c | Describe any methods used to tabulate or visually display results of individual studies and syntheses. | Y | Y | - | Y |
|  | 13d | Describe any methods used to synthesise results and provide a rationale for the choice(s). If meta-analysis was performed, describe the model(s), method(s) to identify the presence and extent of statistical heterogeneity, and software package(s) used. | Y | Y | - | Y |
|  | 13e | Describe any methods used to explore possible causes of heterogeneity among study results (e.g. subgroup analysis, meta regression). | Y | Y | - | Y |
|  | 13f | Describe any sensitivity analyses conducted to assess robustness of the synthesised results. | Y | Y | - | Y |
| Reporting bias  assessment | 14 | Describe any methods used to assess risk of bias due to missing results in a synthesis (arising from reporting biases). | Y | Y | - | Y |
| Certainty assessment | 15 | Describe any methods used to assess certainty (or confidence) in the body of evidence for an outcome. | N | N | - | N |
| **Results** | | | | | | |
| Study selection | 16a | Describe the results of the search and selection process, from the number of records identified in the search to the number of studies included in the review, ideally using a flow diagram (see fig 1). | Y | Y | - | Y |
|  | 16b | Cite studies that might appear to meet the inclusion criteria, but which were excluded, and explain why they were excluded. | PY | Y | Y | Y |
| Study characteristics | 17 | Cite each included study and present its characteristics. | Y | Y | - | Y |
| Risk of bias in studies | 18 | Present assessments of risk of bias for each included study. | N | N | -- | N |
| Results of individual  studies | 19 | For all outcomes, present, for each study: (a) summary statistics for each group (where appropriate) and (b) an effect estimate and its precision (e.g. confidence/credible interval), ideally using structured tables or plots. | Y | Y | - | Y |
| Results of syntheses | 20a | For each synthesis, briefly summarise the characteristics and risk of bias among contributing studies. | PY | PY | - | PY |
|  | 20b | Present results of all statistical syntheses conducted. If meta-analysis was done, present for each the summary estimate and its precision (e.g. confidence/credible interval) and measures of statistical heterogeneity. If comparing groups, describe the direction of the effect. | Y | Y | - | Y |
|  | 20c | Present results of all investigations of possible causes of heterogeneity among study results. | Y | Y | - | Y |
|  | 20d | Present results of all sensitivity analyses conducted to assess the robustness of the synthesised results. | Y | Y | - | Y |
| Reporting biases | 21 | Present assessments of risk of bias due to missing results (arising from reporting biases) for each synthesis assessed. | Y | PY | Y | Y |
| Certainty of evidence | 22 | Present assessments of certainty (or confidence) in the body of evidence for each outcome assessed. | N | N | - | N |
| **Discussion** | | | | | | |
| Discussion | 23a | Provide a general interpretation of the results in the context of other evidence. | Y | Y | - | Y |
|  | 23b | Discuss any limitations of the evidence included in the review. | PY | Y | Y | Y |
|  | 23c | Discuss any limitations of the review processes used. | N | PY | N | N |
|  | 23d | Discuss implications of the results for practice, policy, and future research. | Y | Y | - | Y |
| **Other information** | | | | | | |
| Registration and  protocol | 24a | Provide registration information for the review, including register name and registration number, or state that the review was not registered. | N | N | - | N |
|  | 24b | Indicate where the review protocol can be accessed, or state that a protocol was not prepared. | N | N | - | N |
|  | 24c | Describe and explain any amendments to information provided at registration or in the protocol. | N | N | - | N |
| Support | 25 | Describe sources of financial or non-financial support for the review, and the role of the funders or sponsors in the review. | Y | Y | - | Y |
| Competing interests | 26 | Declare any competing interests of review authors. | Y | Y | - | Y |
| Availability of data,  code, and other  materials | 27 | Report which of the following are publicly available and where they can be found: template data collection forms; data extracted from included studies; data used for all analyses; analytic code; any other materials used in the review. | N | N | - | N |

Table 37：王馨平.(2008).持续正压气道通气对阻塞性睡眠呼吸暂停低通气综合征中重度患者24小时动态血压影响的有效性评价(硕士学位论文,兰州大学).硕士https://kns.cnki.net/kcms2/article/abstract?v=9jT59j8Ji06nE4qyuijwTxro1nn4sKFzXfUXT9i1VmdWAeVIlWc11wPPQkxQv7rjvDTMjL6abWjLORwxIxfjQSf00UEFImXxhxZh2y9uZOuWYb1g0HeecOiawN0gY8_PKrWea6GL7VVWoIxHkXzJgVkm_i5v5KU6JvNEKox8me0kYqH0ECdvEQ==&uniplatform=NZKPT&language=CHS

| Section and topic | Item # | Checklist item | A | B | C | D |
| --- | --- | --- | --- | --- | --- | --- |
| **Title** | | | | | | |
| Title | 1 | Identify the report as a systematic review. | Y | PY | Y | Y |
| **Abstract** | | | | | | |
| Abstract | 2 | See the PRISMA 2020 for Abstracts checklist (table 2). | PY | PY | - | PY |
| **Introduction** | | | | | | |
| Rationale | 3 | Describe the rationale for the review in the context of existing knowledge | Y | Y | - | Y |
| Objectives | 4 | Provide an explicit statement of the objective(s) or question(s) the review addresses. | Y | Y | - | Y |
| **Methods** | | | | | | |
| Eligibility criteria | 5 | Specify the inclusion and exclusion criteria for the review and how studies were grouped for the syntheses. | PY | Y | Y | Y |
| Information sources | 6 | Specify all databases, registers, websites, organisations, reference lists and other sources searched or consulted to identify studies. Specify the date when each source was last searched or consulted. | PY | Y | Y | Y |
| Search strategy | 7 | Present the full search strategies for all databases, registers and websites, including any filters and limits used | Y | Y | - | Y |
| Selection process | 8 | Specify the methods used to decide whether a study met the inclusion criteria of the review, including how many reviewers screened each record and each report retrieved, whether they worked independently, and if applicable, details of automation tools  used in the process. | Y | Y | - | Y |
| Data collection  process | 9 | Specify the methods used to collect data from reports, including how many reviewers collected data from each report, whether they worked independently, any processes for obtaining or confirming data from study investigators, and if applicable, details of automation tools used in the process. | Y | Y | - | Y |
| Data items | 10a | List and define all outcomes for which data were sought. Specify whether all results that were compatible with each outcome domain in each study were sought (e.g. for all measures, time points, analyses), and if not, the methods used to decide which  results to collect. | PY | PY | - | PY |
|  | 10b | List and define all other variables for which data were sought (e.g. participant and intervention characteristics, funding sources). Describe any assumptions made about any missing or unclear information. | PY | PY | - | PY |
| Study risk of bias  assessment | 11 | Specify the methods used to assess risk of bias in the included studies, including details of the tool(s) used, how many reviewers assessed each study and whether they worked independently, and if applicable, details of automation tools used in the process. | PY | PY | - | PY |
| Effect measures | 12 | Specify for each outcome the effect measure(s) (e.g. risk ratio, mean difference) used in the synthesis or presentation of results. | Y | Y | - | Y |
| Synthesis methods | 13a | Describe the processes used to decide which studies were eligible for each synthesis (e.g. tabulating the study intervention characteristics and comparing against the planned groups for each synthesis (item #5)). | N | PY | Y | Y |
|  | 13b | Describe any methods required to prepare the data for presentation or synthesis, such as handling of missing summary statistics, or data conversions. | N | PY | PY | PY |
|  | 13c | Describe any methods used to tabulate or visually display results of individual studies and syntheses. | N | Y | Y | Y |
|  | 13d | Describe any methods used to synthesise results and provide a rationale for the choice(s). If meta-analysis was performed, describe the model(s), method(s) to identify the presence and extent of statistical heterogeneity, and software package(s) used. | Y | Y | - | Y |
|  | 13e | Describe any methods used to explore possible causes of heterogeneity among study results (e.g. subgroup analysis, meta regression). | N | PY | Y | Y |
|  | 13f | Describe any sensitivity analyses conducted to assess robustness of the synthesised results. | PY | Y | Y | Y |
| Reporting bias  assessment | 14 | Describe any methods used to assess risk of bias due to missing results in a synthesis (arising from reporting biases). | PY | PY | - | PY |
| Certainty assessment | 15 | Describe any methods used to assess certainty (or confidence) in the body of evidence for an outcome. | N | N | - | N |
| **Results** | | | | | | |
| Study selection | 16a | Describe the results of the search and selection process, from the number of records identified in the search to the number of studies included in the review, ideally using a flow diagram (see fig 1). | Y | Y | - | Y |
|  | 16b | Cite studies that might appear to meet the inclusion criteria, but which were excluded, and explain why they were excluded. | PY | Y | PY | PY |
| Study characteristics | 17 | Cite each included study and present its characteristics. | Y | Y | - | Y |
| Risk of bias in studies | 18 | Present assessments of risk of bias for each included study. | PY | PY | - | PY |
| Results of individual  studies | 19 | For all outcomes, present, for each study: (a) summary statistics for each group (where appropriate) and (b) an effect estimate and its precision (e.g. confidence/credible interval), ideally using structured tables or plots. | Y | Y | - | Y |
| Results of syntheses | 20a | For each synthesis, briefly summarise the characteristics and risk of bias among contributing studies. | PY | PY | - | PY |
|  | 20b | Present results of all statistical syntheses conducted. If meta-analysis was done, present for each the summary estimate and its precision (e.g. confidence/credible interval) and measures of statistical heterogeneity. If comparing groups, describe the direction of the effect. | Y | Y | - | Y |
|  | 20c | Present results of all investigations of possible causes of heterogeneity among study results. | N | PY | Y | Y |
|  | 20d | Present results of all sensitivity analyses conducted to assess the robustness of the synthesised results. | N | Y | PY | PY |
| Reporting biases | 21 | Present assessments of risk of bias due to missing results (arising from reporting biases) for each synthesis assessed. | PY | PY | - | PY |
| Certainty of evidence | 22 | Present assessments of certainty (or confidence) in the body of evidence for each outcome assessed. | N | N | - | N |
| **Discussion** | | | | | | |
| Discussion | 23a | Provide a general interpretation of the results in the context of other evidence. | Y | Y | - | Y |
|  | 23b | Discuss any limitations of the evidence included in the review. | Y | Y | - | Y |
|  | 23c | Discuss any limitations of the review processes used. | N | PY | Y | Y |
|  | 23d | Discuss implications of the results for practice, policy, and future research. | Y | Y | - | Y |
| **Other information** | | | | | | |
| Registration and  protocol | 24a | Provide registration information for the review, including register name and registration number, or state that the review was not registered. | N | N | - | N |
|  | 24b | Indicate where the review protocol can be accessed, or state that a protocol was not prepared. | N | N | - | N |
|  | 24c | Describe and explain any amendments to information provided at registration or in the protocol. | N | N | - | N |
| Support | 25 | Describe sources of financial or non-financial support for the review, and the role of the funders or sponsors in the review. | N | N | - | N |
| Competing interests | 26 | Declare any competing interests of review authors. | N | N | - | N |
| Availability of data,  code, and other  materials | 27 | Report which of the following are publicly available and where they can be found: template data collection forms; data extracted from included studies; data used for all analyses; analytic code; any other materials used in the review. | PY | N | PY | PY |

Table 38：Montesi SB, Edwards BA, Malhotra A, Bakker JP. The effect of continuous positive airway pressure treatment on blood pressure: a systematic review and meta-analysis of randomized controlled trials. J Clin Sleep Med. 2012 Oct 15;8(5):587-96. doi: 10.5664/jcsm.2170. PMID: 23066375; PMCID: PMC3459209.

| Section and topic | Item # | Checklist item | A | B | C | D |
| --- | --- | --- | --- | --- | --- | --- |
| **Title** | | | | | | |
| Title | 1 | Identify the report as a systematic review. | Y | PY | Y | Y |
| **Abstract** | | | | | | |
| Abstract | 2 | See the PRISMA 2020 for Abstracts checklist (table 2). | PY | PY | - | PY |
| **Introduction** | | | | | | |
| Rationale | 3 | Describe the rationale for the review in the context of existing knowledge | Y | Y | - | Y |
| Objectives | 4 | Provide an explicit statement of the objective(s) or question(s) the review addresses. | Y | Y | - | Y |
| **Methods** | | | | | | |
| Eligibility criteria | 5 | Specify the inclusion and exclusion criteria for the review and how studies were grouped for the syntheses. | PY | Y | Y | Y |
| Information sources | 6 | Specify all databases, registers, websites, organisations, reference lists and other sources searched or consulted to identify studies. Specify the date when each source was last searched or consulted. | PY | PY | - | PY |
| Search strategy | 7 | Present the full search strategies for all databases, registers and websites, including any filters and limits used | Y | Y | - | Y |
| Selection process | 8 | Specify the methods used to decide whether a study met the inclusion criteria of the review, including how many reviewers screened each record and each report retrieved, whether they worked independently, and if applicable, details of automation tools  used in the process. | PY | Y | Y | Y |
| Data collection  process | 9 | Specify the methods used to collect data from reports, including how many reviewers collected data from each report, whether they worked independently, any processes for obtaining or confirming data from study investigators, and if applicable, details of automation tools used in the process. | Y | Y | - | Y |
| Data items | 10a | List and define all outcomes for which data were sought. Specify whether all results that were compatible with each outcome domain in each study were sought (e.g. for all measures, time points, analyses), and if not, the methods used to decide which  results to collect. | PY | PY | - | PY |
|  | 10b | List and define all other variables for which data were sought (e.g. participant and intervention characteristics, funding sources). Describe any assumptions made about any missing or unclear information. | PY | PY | - | PY |
| Study risk of bias  assessment | 11 | Specify the methods used to assess risk of bias in the included studies, including details of the tool(s) used, how many reviewers assessed each study and whether they worked independently, and if applicable, details of automation tools used in the process. | N | N | - | N |
| Effect measures | 12 | Specify for each outcome the effect measure(s) (e.g. risk ratio, mean difference) used in the synthesis or presentation of results. | Y | Y | - | Y |
| Synthesis methods | 13a | Describe the processes used to decide which studies were eligible for each synthesis (e.g. tabulating the study intervention characteristics and comparing against the planned groups for each synthesis (item #5)). | N | PY | Y | Y |
|  | 13b | Describe any methods required to prepare the data for presentation or synthesis, such as handling of missing summary statistics, or data conversions. | Y | PY | Y | Y |
|  | 13c | Describe any methods used to tabulate or visually display results of individual studies and syntheses. | Y | Y | - | Y |
|  | 13d | Describe any methods used to synthesise results and provide a rationale for the choice(s). If meta-analysis was performed, describe the model(s), method(s) to identify the presence and extent of statistical heterogeneity, and software package(s) used. | Y | Y | - | Y |
|  | 13e | Describe any methods used to explore possible causes of heterogeneity among study results (e.g. subgroup analysis, meta regression). | Y | Y | - | Y |
|  | 13f | Describe any sensitivity analyses conducted to assess robustness of the synthesised results. | Y | Y | - | Y |
| Reporting bias  assessment | 14 | Describe any methods used to assess risk of bias due to missing results in a synthesis (arising from reporting biases). | Y | Y | - | Y |
| Certainty assessment | 15 | Describe any methods used to assess certainty (or confidence) in the body of evidence for an outcome. | N | N | - | N |
| **Results** | | | | | | |
| Study selection | 16a | Describe the results of the search and selection process, from the number of records identified in the search to the number of studies included in the review, ideally using a flow diagram (see fig 1). | Y | Y | - | Y |
|  | 16b | Cite studies that might appear to meet the inclusion criteria, but which were excluded, and explain why they were excluded. | PY | Y | PY | PY |
| Study characteristics | 17 | Cite each included study and present its characteristics. | Y | Y | - | Y |
| Risk of bias in studies | 18 | Present assessments of risk of bias for each included study. | N | N | - | N |
| Results of individual  studies | 19 | For all outcomes, present, for each study: (a) summary statistics for each group (where appropriate) and (b) an effect estimate and its precision (e.g. confidence/credible interval), ideally using structured tables or plots. | Y | Y | - | Y |
| Results of syntheses | 20a | For each synthesis, briefly summarise the characteristics and risk of bias among contributing studies. | PY | PY | - | PY |
|  | 20b | Present results of all statistical syntheses conducted. If meta-analysis was done, present for each the summary estimate and its precision (e.g. confidence/credible interval) and measures of statistical heterogeneity. If comparing groups, describe the direction of the effect. | Y | Y | - | Y |
|  | 20c | Present results of all investigations of possible causes of heterogeneity among study results. | Y | Y | - | Y |
|  | 20d | Present results of all sensitivity analyses conducted to assess the robustness of the synthesised results. | Y | Y | - | Y |
| Reporting biases | 21 | Present assessments of risk of bias due to missing results (arising from reporting biases) for each synthesis assessed. | Y | PY | Y | Y |
| Certainty of evidence | 22 | Present assessments of certainty (or confidence) in the body of evidence for each outcome assessed. | N | N | - | N |
| **Discussion** | | | | | | |
| Discussion | 23a | Provide a general interpretation of the results in the context of other evidence. | Y | Y | - | Y |
|  | 23b | Discuss any limitations of the evidence included in the review. | Y | Y | - | Y |
|  | 23c | Discuss any limitations of the review processes used. | N | PY | Y | Y |
|  | 23d | Discuss implications of the results for practice, policy, and future research. | Y | Y | - | Y |
| **Other information** | | | | | | |
| Registration and  protocol | 24a | Provide registration information for the review, including register name and registration number, or state that the review was not registered. | N | N | - | N |
|  | 24b | Indicate where the review protocol can be accessed, or state that a protocol was not prepared. | N | N | - | N |
|  | 24c | Describe and explain any amendments to information provided at registration or in the protocol. | N | N | - | N |
| Support | 25 | Describe sources of financial or non-financial support for the review, and the role of the funders or sponsors in the review. | Y | PY | Y | Y |
| Competing interests | 26 | Declare any competing interests of review authors. | Y | Y | - | Y |
| Availability of data,  code, and other  materials | 27 | Report which of the following are publicly available and where they can be found: template data collection forms; data extracted from included studies; data used for all analyses; analytic code; any other materials used in the review. | N | N | - | N |

Table 39：尹富禹.(2013).持续气道正压通气治疗对阻塞性睡眠呼吸暂停综合症患者血压影响的Meta分析(硕士学位论文,重庆医科大学).硕士https://kns.cnki.net/kcms2/article/abstract?v=9jT59j8Ji07GJvRqP7QjpjiDaw-jA0h1ai-tr8BYJajFP31_bUzUPzkpsv0LekFbmNdEnS8Ai1TUf9ERpkq_kR5p8J6BT5_jzFYacmrQHDZmj5LeMpXUIBckm3pym6Te10sMgxTY_B9TslOBJKqpT5QiVi8y9eCBsO2D2sXmzSTpcXWsxIhAGg==&uniplatform=NZKPT&language=CHS

| Section and topic | Item # | Checklist item | A | B | C | D |
| --- | --- | --- | --- | --- | --- | --- |
| **Title** | | | | | | |
| Title | 1 | Identify the report as a systematic review. | Y | Y | - | Y |
| **Abstract** | | | | | | |
| Abstract | 2 | See the PRISMA 2020 for Abstracts checklist (table 2). | PY | PY | - | PY |
| **Introduction** | | | | | | |
| Rationale | 3 | Describe the rationale for the review in the context of existing knowledge | Y | Y | - | Y |
| Objectives | 4 | Provide an explicit statement of the objective(s) or question(s) the review addresses. | Y | Y | - | Y |
| **Methods** | | | | | | |
| Eligibility criteria | 5 | Specify the inclusion and exclusion criteria for the review and how studies were grouped for the syntheses. | PY | Y | Y | Y |
| Information sources | 6 | Specify all databases, registers, websites, organisations, reference lists and other sources searched or consulted to identify studies. Specify the date when each source was last searched or consulted. | PY | PY | - | PY |
| Search strategy | 7 | Present the full search strategies for all databases, registers and websites, including any filters and limits used | N | N | - | N |
| Selection process | 8 | Specify the methods used to decide whether a study met the inclusion criteria of the review, including how many reviewers screened each record and each report retrieved, whether they worked independently, and if applicable, details of automation tools  used in the process. | N | Y | Y | Y |
| Data collection  process | 9 | Specify the methods used to collect data from reports, including how many reviewers collected data from each report, whether they worked independently, any processes for obtaining or confirming data from study investigators, and if applicable, details of automation tools used in the process. | PY | PY | - | PY |
| Data items | 10a | List and define all outcomes for which data were sought. Specify whether all results that were compatible with each outcome domain in each study were sought (e.g. for all measures, time points, analyses), and if not, the methods used to decide which  results to collect. | PY | PY | - | PY |
|  | 10b | List and define all other variables for which data were sought (e.g. participant and intervention characteristics, funding sources). Describe any assumptions made about any missing or unclear information. | PY | PY | - | PY |
| Study risk of bias  assessment | 11 | Specify the methods used to assess risk of bias in the included studies, including details of the tool(s) used, how many reviewers assessed each study and whether they worked independently, and if applicable, details of automation tools used in the process. | N | N | - | N |
| Effect measures | 12 | Specify for each outcome the effect measure(s) (e.g. risk ratio, mean difference) used in the synthesis or presentation of results. | Y | Y | - | Y |
| Synthesis methods | 13a | Describe the processes used to decide which studies were eligible for each synthesis (e.g. tabulating the study intervention characteristics and comparing against the planned groups for each synthesis (item #5)). | N | PY | N | N |
|  | 13b | Describe any methods required to prepare the data for presentation or synthesis, such as handling of missing summary statistics, or data conversions. | N | N | - | N |
|  | 13c | Describe any methods used to tabulate or visually display results of individual studies and syntheses. | Y | Y | - | Y |
|  | 13d | Describe any methods used to synthesise results and provide a rationale for the choice(s). If meta-analysis was performed, describe the model(s), method(s) to identify the presence and extent of statistical heterogeneity, and software package(s) used. | PY | Y | Y | Y |
|  | 13e | Describe any methods used to explore possible causes of heterogeneity among study results (e.g. subgroup analysis, meta regression). | N | PY | Y | Y |
|  | 13f | Describe any sensitivity analyses conducted to assess robustness of the synthesised results. | N | N | - | N |
| Reporting bias  assessment | 14 | Describe any methods used to assess risk of bias due to missing results in a synthesis (arising from reporting biases). | N | N | - | N |
| Certainty assessment | 15 | Describe any methods used to assess certainty (or confidence) in the body of evidence for an outcome. | N | N | - | N |
| **Results** | | | | | | |
| Study selection | 16a | Describe the results of the search and selection process, from the number of records identified in the search to the number of studies included in the review, ideally using a flow diagram (see fig 1). | Y | Y | - | Y |
|  | 16b | Cite studies that might appear to meet the inclusion criteria, but which were excluded, and explain why they were excluded. | N | N | - | N |
| Study characteristics | 17 | Cite each included study and present its characteristics. | Y | Y | - | Y |
| Risk of bias in studies | 18 | Present assessments of risk of bias for each included study. | PY | Y | Y | Y |
| Results of individual  studies | 19 | For all outcomes, present, for each study: (a) summary statistics for each group (where appropriate) and (b) an effect estimate and its precision (e.g. confidence/credible interval), ideally using structured tables or plots. | PY | Y | Y | Y |
| Results of syntheses | 20a | For each synthesis, briefly summarise the characteristics and risk of bias among contributing studies. | PY | PY | - | PY |
|  | 20b | Present results of all statistical syntheses conducted. If meta-analysis was done, present for each the summary estimate and its precision (e.g. confidence/credible interval) and measures of statistical heterogeneity. If comparing groups, describe the direction of the effect. | Y | Y | - | Y |
|  | 20c | Present results of all investigations of possible causes of heterogeneity among study results. | N | PY | N | N |
|  | 20d | Present results of all sensitivity analyses conducted to assess the robustness of the synthesised results. | N | N | - | N |
| Reporting biases | 21 | Present assessments of risk of bias due to missing results (arising from reporting biases) for each synthesis assessed. | N | N | - | N |
| Certainty of evidence | 22 | Present assessments of certainty (or confidence) in the body of evidence for each outcome assessed. | N | N | - | N |
| **Discussion** | | | | | | |
| Discussion | 23a | Provide a general interpretation of the results in the context of other evidence. | Y | Y | - | Y |
|  | 23b | Discuss any limitations of the evidence included in the review. | Y | Y | - | Y |
|  | 23c | Discuss any limitations of the review processes used. | N | PY | Y | Y |
|  | 23d | Discuss implications of the results for practice, policy, and future research. | Y | Y | - | Y |
| **Other information** | | | | | | |
| Registration and  protocol | 24a | Provide registration information for the review, including register name and registration number, or state that the review was not registered. | N | N | - | N |
|  | 24b | Indicate where the review protocol can be accessed, or state that a protocol was not prepared. | N | N | - | N |
|  | 24c | Describe and explain any amendments to information provided at registration or in the protocol. | N | N | - | N |
| Support | 25 | Describe sources of financial or non-financial support for the review, and the role of the funders or sponsors in the review. | N | N | - | N |
| Competing interests | 26 | Declare any competing interests of review authors. | Y | Y | - | Y |
| Availability of data,  code, and other  materials | 27 | Report which of the following are publicly available and where they can be found: template data collection forms; data extracted from included studies; data used for all analyses; analytic code; any other materials used in the review. | N | N | - | N |

Table 40：符翠萍,朱芬,刘子龙,励雯静,吴晓丹,吴旭... & 李善群.(2014).持续气道正压通气对阻塞性睡眠呼吸暂停低通气综合征患者血压影响的荟萃分析.世界临床药物,35(06),358-363.https://doi.org/10.13683/j.wph.2014.06.015.

| Section and topic | Item # | Checklist item | A | B | C | D |
| --- | --- | --- | --- | --- | --- | --- |
| **Title** | | | | | | |
| Title | 1 | Identify the report as a systematic review. | N | PY | Y | Y |
| **Abstract** | | | | | | |
| Abstract | 2 | See the PRISMA 2020 for Abstracts checklist (table 2). | PY | PY | - | PY |
| **Introduction** | | | | | | |
| Rationale | 3 | Describe the rationale for the review in the context of existing knowledge | PY | Y | Y | Y |
| Objectives | 4 | Provide an explicit statement of the objective(s) or question(s) the review addresses. | Y | Y | - | Y |
| **Methods** | | | | | | |
| Eligibility criteria | 5 | Specify the inclusion and exclusion criteria for the review and how studies were grouped for the syntheses. | PY | Y | Y | Y |
| Information sources | 6 | Specify all databases, registers, websites, organisations, reference lists and other sources searched or consulted to identify studies. Specify the date when each source was last searched or consulted. | PY | PY | - | PY |
| Search strategy | 7 | Present the full search strategies for all databases, registers and websites, including any filters and limits used | PY | N | PY | PY |
| Selection process | 8 | Specify the methods used to decide whether a study met the inclusion criteria of the review, including how many reviewers screened each record and each report retrieved, whether they worked independently, and if applicable, details of automation tools  used in the process. | PY | PY | - | PY |
| Data collection  process | 9 | Specify the methods used to collect data from reports, including how many reviewers collected data from each report, whether they worked independently, any processes for obtaining or confirming data from study investigators, and if applicable, details of automation tools used in the process. | N | PY | PY | PY |
| Data items | 10a | List and define all outcomes for which data were sought. Specify whether all results that were compatible with each outcome domain in each study were sought (e.g. for all measures, time points, analyses), and if not, the methods used to decide which  results to collect. | PY | PY | - | PY |
|  | 10b | List and define all other variables for which data were sought (e.g. participant and intervention characteristics, funding sources). Describe any assumptions made about any missing or unclear information. | PY | PY | - | PY |
| Study risk of bias  assessment | 11 | Specify the methods used to assess risk of bias in the included studies, including details of the tool(s) used, how many reviewers assessed each study and whether they worked independently, and if applicable, details of automation tools used in the process. | N | PY | PY | PY |
| Effect measures | 12 | Specify for each outcome the effect measure(s) (e.g. risk ratio, mean difference) used in the synthesis or presentation of results. | Y | Y | - | Y |
| Synthesis methods | 13a | Describe the processes used to decide which studies were eligible for each synthesis (e.g. tabulating the study intervention characteristics and comparing against the planned groups for each synthesis (item #5)). | N | Y | PY | PY |
|  | 13b | Describe any methods required to prepare the data for presentation or synthesis, such as handling of missing summary statistics, or data conversions. | N | N | - | N |
|  | 13c | Describe any methods used to tabulate or visually display results of individual studies and syntheses. | Y | Y | - | Y |
|  | 13d | Describe any methods used to synthesise results and provide a rationale for the choice(s). If meta-analysis was performed, describe the model(s), method(s) to identify the presence and extent of statistical heterogeneity, and software package(s) used. | Y | PY | Y | Y |
|  | 13e | Describe any methods used to explore possible causes of heterogeneity among study results (e.g. subgroup analysis, meta regression). | PY | Y | Y | Y |
|  | 13f | Describe any sensitivity analyses conducted to assess robustness of the synthesised results. | PY | PY | - | PY |
| Reporting bias  assessment | 14 | Describe any methods used to assess risk of bias due to missing results in a synthesis (arising from reporting biases). | PY | N | Y | Y |
| Certainty assessment | 15 | Describe any methods used to assess certainty (or confidence) in the body of evidence for an outcome. | N | N | - | N |
| **Results** | | | | | | |
| Study selection | 16a | Describe the results of the search and selection process, from the number of records identified in the search to the number of studies included in the review, ideally using a flow diagram (see fig 1). | Y | Y | - | Y |
|  | 16b | Cite studies that might appear to meet the inclusion criteria, but which were excluded, and explain why they were excluded. | PY | N | N | N |
| Study characteristics | 17 | Cite each included study and present its characteristics. | Y | Y | - | Y |
| Risk of bias in studies | 18 | Present assessments of risk of bias for each included study. | N | PY | PY | PY |
| Results of individual  studies | 19 | For all outcomes, present, for each study: (a) summary statistics for each group (where appropriate) and (b) an effect estimate and its precision (e.g. confidence/credible interval), ideally using structured tables or plots. | Y | Y | - | Y |
| Results of syntheses | 20a | For each synthesis, briefly summarise the characteristics and risk of bias among contributing studies. | PY | PY | - | PY |
|  | 20b | Present results of all statistical syntheses conducted. If meta-analysis was done, present for each the summary estimate and its precision (e.g. confidence/credible interval) and measures of statistical heterogeneity. If comparing groups, describe the direction of the effect. | Y | Y | - | Y |
|  | 20c | Present results of all investigations of possible causes of heterogeneity among study results. | PY | Y | PY | PY |
|  | 20d | Present results of all sensitivity analyses conducted to assess the robustness of the synthesised results. | PY | PY | - | PY |
| Reporting biases | 21 | Present assessments of risk of bias due to missing results (arising from reporting biases) for each synthesis assessed. | PY | PY | - | PY |
| Certainty of evidence | 22 | Present assessments of certainty (or confidence) in the body of evidence for each outcome assessed. | N | N | - | N |
| **Discussion** | | | | | | |
| Discussion | 23a | Provide a general interpretation of the results in the context of other evidence. | Y | Y | - | Y |
|  | 23b | Discuss any limitations of the evidence included in the review. | PY | Y | Y | Y |
|  | 23c | Discuss any limitations of the review processes used. | N | Y | PY | PY |
|  | 23d | Discuss implications of the results for practice, policy, and future research. | Y | Y | - | Y |
| **Other information** | | | | | | |
| Registration and  protocol | 24a | Provide registration information for the review, including register name and registration number, or state that the review was not registered. | N | N | - | N |
|  | 24b | Indicate where the review protocol can be accessed, or state that a protocol was not prepared. | N | N | - | N |
|  | 24c | Describe and explain any amendments to information provided at registration or in the protocol. | N | N | - | N |
| Support | 25 | Describe sources of financial or non-financial support for the review, and the role of the funders or sponsors in the review. | PY | PY | - | PY |
| Competing interests | 26 | Declare any competing interests of review authors. | N | N | - | N |
| Availability of data,  code, and other  materials | 27 | Report which of the following are publicly available and where they can be found: template data collection forms; data extracted from included studies; data used for all analyses; analytic code; any other materials used in the review. | N | N | - | N |

Table 41：Schein A.S.O., Kerkhoff A.C., Coronel C.C., Plentz R.D.M., Sbruzzi G. (2014). Continuous positive airway pressure reduces blood pressure in patients with obstructive sleep apnea; A systematic review and meta-analysis with 1000 patients. Journal of Hypertension, 32(9), 1762-1773. http://dx.doi.org/10.1097/HJH.0000000000000250

| Section and topic | Item # | Checklist item | A | B | C | D |
| --- | --- | --- | --- | --- | --- | --- |
| **Title** | | | | | | |
| Title | 1 | Identify the report as a systematic review. | Y | Y | - | Y |
| **Abstract** | | | | | | |
| Abstract | 2 | See the PRISMA 2020 for Abstracts checklist (table 2). | PY | PY | - | PY |
| **Introduction** | | | | | | |
| Rationale | 3 | Describe the rationale for the review in the context of existing knowledge | Y | Y | - | Y |
| Objectives | 4 | Provide an explicit statement of the objective(s) or question(s) the review addresses. | Y | Y | - | Y |
| **Methods** | | | | | | |
| Eligibility criteria | 5 | Specify the inclusion and exclusion criteria for the review and how studies were grouped for the syntheses. | PY | Y | Y | Y |
| Information sources | 6 | Specify all databases, registers, websites, organisations, reference lists and other sources searched or consulted to identify studies. Specify the date when each source was last searched or consulted. | PY | Y | Y | Y |
| Search strategy | 7 | Present the full search strategies for all databases, registers and websites, including any filters and limits used | PY | Y | Y | Y |
| Selection process | 8 | Specify the methods used to decide whether a study met the inclusion criteria of the review, including how many reviewers screened each record and each report retrieved, whether they worked independently, and if applicable, details of automation tools  used in the process. | Y | Y | - | Y |
| Data collection  process | 9 | Specify the methods used to collect data from reports, including how many reviewers collected data from each report, whether they worked independently, any processes for obtaining or confirming data from study investigators, and if applicable, details of automation tools used in the process. | Y | Y | - | Y |
| Data items | 10a | List and define all outcomes for which data were sought. Specify whether all results that were compatible with each outcome domain in each study were sought (e.g. for all measures, time points, analyses), and if not, the methods used to decide which  results to collect. | PY | PY | - | PY |
|  | 10b | List and define all other variables for which data were sought (e.g. participant and intervention characteristics, funding sources). Describe any assumptions made about any missing or unclear information. | PY | PY | - | PY |
| Study risk of bias  assessment | 11 | Specify the methods used to assess risk of bias in the included studies, including details of the tool(s) used, how many reviewers assessed each study and whether they worked independently, and if applicable, details of automation tools used in the process. | PY | PY | - | PY |
| Effect measures | 12 | Specify for each outcome the effect measure(s) (e.g. risk ratio, mean difference) used in the synthesis or presentation of results. | Y | Y | - | Y |
| Synthesis methods | 13a | Describe the processes used to decide which studies were eligible for each synthesis (e.g. tabulating the study intervention characteristics and comparing against the planned groups for each synthesis (item #5)). | N | Y | PY | PY |
|  | 13b | Describe any methods required to prepare the data for presentation or synthesis, such as handling of missing summary statistics, or data conversions. | N | N | - | N |
|  | 13c | Describe any methods used to tabulate or visually display results of individual studies and syntheses. | Y | Y | - | Y |
|  | 13d | Describe any methods used to synthesise results and provide a rationale for the choice(s). If meta-analysis was performed, describe the model(s), method(s) to identify the presence and extent of statistical heterogeneity, and software package(s) used. | Y | Y | - | Y |
|  | 13e | Describe any methods used to explore possible causes of heterogeneity among study results (e.g. subgroup analysis, meta regression). | N | Y | Y | Y |
|  | 13f | Describe any sensitivity analyses conducted to assess robustness of the synthesised results. | Y | Y | - | Y |
| Reporting bias  assessment | 14 | Describe any methods used to assess risk of bias due to missing results in a synthesis (arising from reporting biases). | N | N | - | N |
| Certainty assessment | 15 | Describe any methods used to assess certainty (or confidence) in the body of evidence for an outcome. | N | N | - | N |
| **Results** | | | | | | |
| Study selection | 16a | Describe the results of the search and selection process, from the number of records identified in the search to the number of studies included in the review, ideally using a flow diagram (see fig 1). | Y | Y | - | Y |
|  | 16b | Cite studies that might appear to meet the inclusion criteria, but which were excluded, and explain why they were excluded. | PY | PY | - | PY |
| Study characteristics | 17 | Cite each included study and present its characteristics. | Y | Y | - | Y |
| Risk of bias in studies | 18 | Present assessments of risk of bias for each included study. | Y | Y | - | Y |
| Results of individual  studies | 19 | For all outcomes, present, for each study: (a) summary statistics for each group (where appropriate) and (b) an effect estimate and its precision (e.g. confidence/credible interval), ideally using structured tables or plots. | Y | Y | - | Y |
| Results of syntheses | 20a | For each synthesis, briefly summarise the characteristics and risk of bias among contributing studies. | Y | PY | PY | PY |
|  | 20b | Present results of all statistical syntheses conducted. If meta-analysis was done, present for each the summary estimate and its precision (e.g. confidence/credible interval) and measures of statistical heterogeneity. If comparing groups, describe the direction of the effect. | Y | Y | - | Y |
|  | 20c | Present results of all investigations of possible causes of heterogeneity among study results. | PY | Y | Y | Y |
|  | 20d | Present results of all sensitivity analyses conducted to assess the robustness of the synthesised results. | Y | Y | - | Y |
| Reporting biases | 21 | Present assessments of risk of bias due to missing results (arising from reporting biases) for each synthesis assessed. | N | N | - | N |
| Certainty of evidence | 22 | Present assessments of certainty (or confidence) in the body of evidence for each outcome assessed. | N | N | - | N |
| **Discussion** | | | | | | |
| Discussion | 23a | Provide a general interpretation of the results in the context of other evidence. | Y | Y | - | Y |
|  | 23b | Discuss any limitations of the evidence included in the review. | Y | Y | - | Y |
|  | 23c | Discuss any limitations of the review processes used. | N | Y | PY | PY |
|  | 23d | Discuss implications of the results for practice, policy, and future research. | Y | Y | - | Y |
| **Other information** | | | | | | |
| Registration and  protocol | 24a | Provide registration information for the review, including register name and registration number, or state that the review was not registered. | N | N | - | N |
|  | 24b | Indicate where the review protocol can be accessed, or state that a protocol was not prepared. | N | N | - | N |
|  | 24c | Describe and explain any amendments to information provided at registration or in the protocol. | N | N | - | N |
| Support | 25 | Describe sources of financial or non-financial support for the review, and the role of the funders or sponsors in the review. | N | N | - | N |
| Competing interests | 26 | Declare any competing interests of review authors. | Y | Y | - | Y |
| Availability of data,  code, and other  materials | 27 | Report which of the following are publicly available and where they can be found: template data collection forms; data extracted from included studies; data used for all analyses; analytic code; any other materials used in the review. | N | N | - | N |

Table 42：李君,李晓艳,蒋学俊,万为国 & 刘万里.(2015).持续气道正压通气对阻塞性睡眠呼吸暂停综合征患者降压疗效的Meta分析.海南医学,26(06),909-914.

| Section and topic | Item # | Checklist item | A | B | C | D |
| --- | --- | --- | --- | --- | --- | --- |
| **Title** | | | | | | |
| Title | 1 | Identify the report as a systematic review. | N | Y | Y | Y |
| **Abstract** | | | | | | |
| Abstract | 2 | See the PRISMA 2020 for Abstracts checklist (table 2). | PY | PY | - | PY |
| **Introduction** | | | | | | |
| Rationale | 3 | Describe the rationale for the review in the context of existing knowledge | Y | Y | - | Y |
| Objectives | 4 | Provide an explicit statement of the objective(s) or question(s) the review addresses. | Y | Y | - | Y |
| **Methods** | | | | | | |
| Eligibility criteria | 5 | Specify the inclusion and exclusion criteria for the review and how studies were grouped for the syntheses. | PY | Y | Y | Y |
| Information sources | 6 | Specify all databases, registers, websites, organisations, reference lists and other sources searched or consulted to identify studies. Specify the date when each source was last searched or consulted. | PY | PY | - | PY |
| Search strategy | 7 | Present the full search strategies for all databases, registers and websites, including any filters and limits used | PY | N | PY | PY |
| Selection process | 8 | Specify the methods used to decide whether a study met the inclusion criteria of the review, including how many reviewers screened each record and each report retrieved, whether they worked independently, and if applicable, details of automation tools  used in the process. | Y | PY | Y | Y |
| Data collection  process | 9 | Specify the methods used to collect data from reports, including how many reviewers collected data from each report, whether they worked independently, any processes for obtaining or confirming data from study investigators, and if applicable, details of automation tools used in the process. | N | N | - | N |
| Data items | 10a | List and define all outcomes for which data were sought. Specify whether all results that were compatible with each outcome domain in each study were sought (e.g. for all measures, time points, analyses), and if not, the methods used to decide which  results to collect. | PY | Y | Y | Y |
|  | 10b | List and define all other variables for which data were sought (e.g. participant and intervention characteristics, funding sources). Describe any assumptions made about any missing or unclear information. | PY | N | Y | Y |
| Study risk of bias  assessment | 11 | Specify the methods used to assess risk of bias in the included studies, including details of the tool(s) used, how many reviewers assessed each study and whether they worked independently, and if applicable, details of automation tools used in the process. | Y | PY | Y | Y |
| Effect measures | 12 | Specify for each outcome the effect measure(s) (e.g. risk ratio, mean difference) used in the synthesis or presentation of results. | Y | Y | Y | Y |
| Synthesis methods | 13a | Describe the processes used to decide which studies were eligible for each synthesis (e.g. tabulating the study intervention characteristics and comparing against the planned groups for each synthesis (item #5)). | N | Y | PY | PY |
|  | 13b | Describe any methods required to prepare the data for presentation or synthesis, such as handling of missing summary statistics, or data conversions. | N | N | - | N |
|  | 13c | Describe any methods used to tabulate or visually display results of individual studies and syntheses. | Y | Y | - | Y |
|  | 13d | Describe any methods used to synthesise results and provide a rationale for the choice(s). If meta-analysis was performed, describe the model(s), method(s) to identify the presence and extent of statistical heterogeneity, and software package(s) used. | Y | PY | Y | Y |
|  | 13e | Describe any methods used to explore possible causes of heterogeneity among study results (e.g. subgroup analysis, meta regression). | N | N | - | N |
|  | 13f | Describe any sensitivity analyses conducted to assess robustness of the synthesised results. | Y | Y | - | Y |
| Reporting bias  assessment | 14 | Describe any methods used to assess risk of bias due to missing results in a synthesis (arising from reporting biases). | PY | PY | - | PY |
| Certainty assessment | 15 | Describe any methods used to assess certainty (or confidence) in the body of evidence for an outcome. | N | N | - | N |
| **Results** | | | | | | |
| Study selection | 16a | Describe the results of the search and selection process, from the number of records identified in the search to the number of studies included in the review, ideally using a flow diagram (see fig 1). | PY | N | N | N |
|  | 16b | Cite studies that might appear to meet the inclusion criteria, but which were excluded, and explain why they were excluded. | PY | N | N | N |
| Study characteristics | 17 | Cite each included study and present its characteristics. | Y | Y | - | Y |
| Risk of bias in studies | 18 | Present assessments of risk of bias for each included study. | Y | PY | Y | Y |
| Results of individual  studies | 19 | For all outcomes, present, for each study: (a) summary statistics for each group (where appropriate) and (b) an effect estimate and its precision (e.g. confidence/credible interval), ideally using structured tables or plots. | Y | Y | - | Y |
| Results of syntheses | 20a | For each synthesis, briefly summarise the characteristics and risk of bias among contributing studies. | Y | PY | PY | PY |
|  | 20b | Present results of all statistical syntheses conducted. If meta-analysis was done, present for each the summary estimate and its precision (e.g. confidence/credible interval) and measures of statistical heterogeneity. If comparing groups, describe the direction of the effect. | Y | Y | - | Y |
|  | 20c | Present results of all investigations of possible causes of heterogeneity among study results. | N | N | - | N |
|  | 20d | Present results of all sensitivity analyses conducted to assess the robustness of the synthesised results. | Y | PY | Y | Y |
| Reporting biases | 21 | Present assessments of risk of bias due to missing results (arising from reporting biases) for each synthesis assessed. | PY | PY | - | PY |
| Certainty of evidence | 22 | Present assessments of certainty (or confidence) in the body of evidence for each outcome assessed. | N | N | - | N |
| **Discussion** | | | | | | |
| Discussion | 23a | Provide a general interpretation of the results in the context of other evidence. | Y | Y | - | Y |
|  | 23b | Discuss any limitations of the evidence included in the review. | Y | Y | - | Y |
|  | 23c | Discuss any limitations of the review processes used. | N | PY | PY | PY |
|  | 23d | Discuss implications of the results for practice, policy, and future research. | Y | Y | - | Y |
| **Other information** | | | | | | |
| Registration and  protocol | 24a | Provide registration information for the review, including register name and registration number, or state that the review was not registered. | N | N | - | N |
|  | 24b | Indicate where the review protocol can be accessed, or state that a protocol was not prepared. | N | N | - | N |
|  | 24c | Describe and explain any amendments to information provided at registration or in the protocol. | N | N | - | N |
| Support | 25 | Describe sources of financial or non-financial support for the review, and the role of the funders or sponsors in the review. | N | N | - | N |
| Competing interests | 26 | Declare any competing interests of review authors. | N | N | - | N |
| Availability of data,  code, and other  materials | 27 | Report which of the following are publicly available and where they can be found: template data collection forms; data extracted from included studies; data used for all analyses; analytic code; any other materials used in the review. | N | N | - | N |

Table 43：Hu X., Fan J., Chen S., Yin Y., Zrenner B. (2015). The Role of Continuous Positive Airway Pressure in Blood Pressure Control for Patients With Obstructive Sleep Apnea and Hypertension: A Meta-Analysis of Randomized Controlled Trials. Journal of Clinical Hypertension, 17(3), 215-222. http://dx.doi.org/10.1111/jch.12472

| Section and topic | Item # | Checklist item | A | B | C | D |
| --- | --- | --- | --- | --- | --- | --- |
| **Title** | | | | | | |
| Title | 1 | Identify the report as a systematic review. | N | N | - | N |
| **Abstract** | | | | | | |
| Abstract | 2 | See the PRISMA 2020 for Abstracts checklist (table 2). | PY | PY | - | PY |
| **Introduction** | | | | | | |
| Rationale | 3 | Describe the rationale for the review in the context of existing knowledge | Y | Y | - | Y |
| Objectives | 4 | Provide an explicit statement of the objective(s) or question(s) the review addresses. | Y | Y | - | Y |
| **Methods** | | | | | | |
| Eligibility criteria | 5 | Specify the inclusion and exclusion criteria for the review and how studies were grouped for the syntheses. | PY | PY | - | PY |
| Information sources | 6 | Specify all databases, registers, websites, organisations, reference lists and other sources searched or consulted to identify studies. Specify the date when each source was last searched or consulted. | PY | PY | - | PY |
| Search strategy | 7 | Present the full search strategies for all databases, registers and websites, including any filters and limits used | PY | N | N | N |
| Selection process | 8 | Specify the methods used to decide whether a study met the inclusion criteria of the review, including how many reviewers screened each record and each report retrieved, whether they worked independently, and if applicable, details of automation tools  used in the process. | Y | PY | Y | Y |
| Data collection  process | 9 | Specify the methods used to collect data from reports, including how many reviewers collected data from each report, whether they worked independently, any processes for obtaining or confirming data from study investigators, and if applicable, details of automation tools used in the process. | Y | PY | Y | Y |
| Data items | 10a | List and define all outcomes for which data were sought. Specify whether all results that were compatible with each outcome domain in each study were sought (e.g. for all measures, time points, analyses), and if not, the methods used to decide which  results to collect. | PY | PY | - | PY |
|  | 10b | List and define all other variables for which data were sought (e.g. participant and intervention characteristics, funding sources). Describe any assumptions made about any missing or unclear information. | PY | PY | - | PY |
| Study risk of bias  assessment | 11 | Specify the methods used to assess risk of bias in the included studies, including details of the tool(s) used, how many reviewers assessed each study and whether they worked independently, and if applicable, details of automation tools used in the process. | Y | PY | Y | Y |
| Effect measures | 12 | Specify for each outcome the effect measure(s) (e.g. risk ratio, mean difference) used in the synthesis or presentation of results. | Y | Y | - | Y |
| Synthesis methods | 13a | Describe the processes used to decide which studies were eligible for each synthesis (e.g. tabulating the study intervention characteristics and comparing against the planned groups for each synthesis (item #5)). | N | N | - | N |
|  | 13b | Describe any methods required to prepare the data for presentation or synthesis, such as handling of missing summary statistics, or data conversions. | PY | PY | - | PY |
|  | 13c | Describe any methods used to tabulate or visually display results of individual studies and syntheses. | Y | Y | - | Y |
|  | 13d | Describe any methods used to synthesise results and provide a rationale for the choice(s). If meta-analysis was performed, describe the model(s), method(s) to identify the presence and extent of statistical heterogeneity, and software package(s) used. | Y | Y | - | Y |
|  | 13e | Describe any methods used to explore possible causes of heterogeneity among study results (e.g. subgroup analysis, meta regression). | Y | Y | - | Y |
|  | 13f | Describe any sensitivity analyses conducted to assess robustness of the synthesised results. | Y | Y | - | Y |
| Reporting bias  assessment | 14 | Describe any methods used to assess risk of bias due to missing results in a synthesis (arising from reporting biases). | Y | PY | Y | Y |
| Certainty assessment | 15 | Describe any methods used to assess certainty (or confidence) in the body of evidence for an outcome. | N | N | - | N |
| **Results** | | | | | | |
| Study selection | 16a | Describe the results of the search and selection process, from the number of records identified in the search to the number of studies included in the review, ideally using a flow diagram (see fig 1). | Y | Y | - | Y |
|  | 16b | Cite studies that might appear to meet the inclusion criteria, but which were excluded, and explain why they were excluded. | PY | N | N | N |
| Study characteristics | 17 | Cite each included study and present its characteristics. | Y | Y | - | Y |
| Risk of bias in studies | 18 | Present assessments of risk of bias for each included study. | Y | PY | Y | Y |
| Results of individual  studies | 19 | For all outcomes, present, for each study: (a) summary statistics for each group (where appropriate) and (b) an effect estimate and its precision (e.g. confidence/credible interval), ideally using structured tables or plots. | Y | Y | - | Y |
| Results of syntheses | 20a | For each synthesis, briefly summarise the characteristics and risk of bias among contributing studies. | Y | PY | Y | Y |
|  | 20b | Present results of all statistical syntheses conducted. If meta-analysis was done, present for each the summary estimate and its precision (e.g. confidence/credible interval) and measures of statistical heterogeneity. If comparing groups, describe the direction of the effect. | Y | Y | - | Y |
|  | 20c | Present results of all investigations of possible causes of heterogeneity among study results. | Y | Y | - | Y |
|  | 20d | Present results of all sensitivity analyses conducted to assess the robustness of the synthesised results. | Y | Y | - | Y |
| Reporting biases | 21 | Present assessments of risk of bias due to missing results (arising from reporting biases) for each synthesis assessed. | Y | PY | Y | Y |
| Certainty of evidence | 22 | Present assessments of certainty (or confidence) in the body of evidence for each outcome assessed. | N | N | - | N |
| **Discussion** | | | | | | |
| Discussion | 23a | Provide a general interpretation of the results in the context of other evidence. | Y | Y | - | Y |
|  | 23b | Discuss any limitations of the evidence included in the review. | Y | Y | - | Y |
|  | 23c | Discuss any limitations of the review processes used. | N | PY | Y | Y |
|  | 23d | Discuss implications of the results for practice, policy, and future research. | Y | Y | - | Y |
| **Other information** | | | | | | |
| Registration and  protocol | 24a | Provide registration information for the review, including register name and registration number, or state that the review was not registered. | N | N | - | N |
|  | 24b | Indicate where the review protocol can be accessed, or state that a protocol was not prepared. | N | N | - | N |
|  | 24c | Describe and explain any amendments to information provided at registration or in the protocol. | N | N | - | N |
| Support | 25 | Describe sources of financial or non-financial support for the review, and the role of the funders or sponsors in the review. | PY | Y | Y | Y |
| Competing interests | 26 | Declare any competing interests of review authors. | Y | Y | - | Y |
| Availability of data,  code, and other  materials | 27 | Report which of the following are publicly available and where they can be found: template data collection forms; data extracted from included studies; data used for all analyses; analytic code; any other materials used in the review. | N | N | - | N |

Table 44：Liu L., Cao Q., Guo Z., Dai Q. (2016). Continuous Positive Airway Pressure in Patients With Obstructive Sleep Apnea and Resistant Hypertension: A Meta-Analysis of Randomized Controlled Trials. Journal of Clinical Hypertension, 18(2), 153-158. http://dx.doi.org/10.1111/jch.12639

| Section and topic | Item # | Checklist item | A | B | C | D |
| --- | --- | --- | --- | --- | --- | --- |
| **Title** | | | | | | |
| Title | 1 | Identify the report as a systematic review. | N | N | - | N |
| **Abstract** | | | | | | |
| Abstract | 2 | See the PRISMA 2020 for Abstracts checklist (table 2). | PY | PY | - | PY |
| **Introduction** | | | | | | |
| Rationale | 3 | Describe the rationale for the review in the context of existing knowledge | Y | Y | - | Y |
| Objectives | 4 | Provide an explicit statement of the objective(s) or question(s) the review addresses. | Y | Y | - | Y |
| **Methods** | | | | | | |
| Eligibility criteria | 5 | Specify the inclusion and exclusion criteria for the review and how studies were grouped for the syntheses. | PY | PY | - | PY |
| Information sources | 6 | Specify all databases, registers, websites, organisations, reference lists and other sources searched or consulted to identify studies. Specify the date when each source was last searched or consulted. | PY | PY | - | PY |
| Search strategy | 7 | Present the full search strategies for all databases, registers and websites, including any filters and limits used | PY | N | N | PY |
| Selection process | 8 | Specify the methods used to decide whether a study met the inclusion criteria of the review, including how many reviewers screened each record and each report retrieved, whether they worked independently, and if applicable, details of automation tools  used in the process. | Y | PY | Y | Y |
| Data collection  process | 9 | Specify the methods used to collect data from reports, including how many reviewers collected data from each report, whether they worked independently, any processes for obtaining or confirming data from study investigators, and if applicable, details of automation tools used in the process. | Y | PY | Y | Y |
| Data items | 10a | List and define all outcomes for which data were sought. Specify whether all results that were compatible with each outcome domain in each study were sought (e.g. for all measures, time points, analyses), and if not, the methods used to decide which  results to collect. | PY | PY | - | PY |
|  | 10b | List and define all other variables for which data were sought (e.g. participant and intervention characteristics, funding sources). Describe any assumptions made about any missing or unclear information. | PY | PY | - | PY |
| Study risk of bias  assessment | 11 | Specify the methods used to assess risk of bias in the included studies, including details of the tool(s) used, how many reviewers assessed each study and whether they worked independently, and if applicable, details of automation tools used in the process. | Y | PY | Y | Y |
| Effect measures | 12 | Specify for each outcome the effect measure(s) (e.g. risk ratio, mean difference) used in the synthesis or presentation of results. | Y | Y | - | Y |
| Synthesis methods | 13a | Describe the processes used to decide which studies were eligible for each synthesis (e.g. tabulating the study intervention characteristics and comparing against the planned groups for each synthesis (item #5)). | N | N | - | N |
|  | 13b | Describe any methods required to prepare the data for presentation or synthesis, such as handling of missing summary statistics, or data conversions. | N | N | - | N |
|  | 13c | Describe any methods used to tabulate or visually display results of individual studies and syntheses. | Y | Y | - | Y |
|  | 13d | Describe any methods used to synthesise results and provide a rationale for the choice(s). If meta-analysis was performed, describe the model(s), method(s) to identify the presence and extent of statistical heterogeneity, and software package(s) used. | Y | Y | - | Y |
|  | 13e | Describe any methods used to explore possible causes of heterogeneity among study results (e.g. subgroup analysis, meta regression). | Y | Y | - | Y |
|  | 13f | Describe any sensitivity analyses conducted to assess robustness of the synthesised results. | Y | Y | - | Y |
| Reporting bias  assessment | 14 | Describe any methods used to assess risk of bias due to missing results in a synthesis (arising from reporting biases). | Y | PY | Y | Y |
| Certainty assessment | 15 | Describe any methods used to assess certainty (or confidence) in the body of evidence for an outcome. | N | N | - | N |
| **Results** | | | | | | |
| Study selection | 16a | Describe the results of the search and selection process, from the number of records identified in the search to the number of studies included in the review, ideally using a flow diagram (see fig 1). | Y | Y | - | Y |
|  | 16b | Cite studies that might appear to meet the inclusion criteria, but which were excluded, and explain why they were excluded. | PY | N | Y | Y |
| Study characteristics | 17 | Cite each included study and present its characteristics. | Y | Y | Y | Y |
| Risk of bias in studies | 18 | Present assessments of risk of bias for each included study. | Y | PY | Y | Y |
| Results of individual  studies | 19 | For all outcomes, present, for each study: (a) summary statistics for each group (where appropriate) and (b) an effect estimate and its precision (e.g. confidence/credible interval), ideally using structured tables or plots. | Y | Y | - | Y |
| Results of syntheses | 20a | For each synthesis, briefly summarise the characteristics and risk of bias among contributing studies. | Y | PY | Y | Y |
|  | 20b | Present results of all statistical syntheses conducted. If meta-analysis was done, present for each the summary estimate and its precision (e.g. confidence/credible interval) and measures of statistical heterogeneity. If comparing groups, describe the direction of the effect. | Y | Y | - | Y |
|  | 20c | Present results of all investigations of possible causes of heterogeneity among study results. | PY | Y | Y | Y |
|  | 20d | Present results of all sensitivity analyses conducted to assess the robustness of the synthesised results. | Y | Y | - | Y |
| Reporting biases | 21 | Present assessments of risk of bias due to missing results (arising from reporting biases) for each synthesis assessed. | Y | PY | Y | Y |
| Certainty of evidence | 22 | Present assessments of certainty (or confidence) in the body of evidence for each outcome assessed. | N | N | N | N |
| **Discussion** | | | | | | |
| Discussion | 23a | Provide a general interpretation of the results in the context of other evidence. | Y | Y | - | Y |
|  | 23b | Discuss any limitations of the evidence included in the review. | Y | Y | - | Y |
|  | 23c | Discuss any limitations of the review processes used. | N | PY | Y | Y |
|  | 23d | Discuss implications of the results for practice, policy, and future research. | Y | Y | - | Y |
| **Other information** | | | | | | |
| Registration and  protocol | 24a | Provide registration information for the review, including register name and registration number, or state that the review was not registered. | N | N | - | N |
|  | 24b | Indicate where the review protocol can be accessed, or state that a protocol was not prepared. | N | N | - | N |
|  | 24c | Describe and explain any amendments to information provided at registration or in the protocol. | N | N | - | N |
| Support | 25 | Describe sources of financial or non-financial support for the review, and the role of the funders or sponsors in the review. | Y | Y | - | Y |
| Competing interests | 26 | Declare any competing interests of review authors. | Y | Y | - | Y |
| Availability of data,  code, and other  materials | 27 | Report which of the following are publicly available and where they can be found: template data collection forms; data extracted from included studies; data used for all analyses; analytic code; any other materials used in the review. | N | N | - | N |

Table 45：Sun Y., Huang Z.-Y., Sun Q.-R., Qiu L.-P., Zhou T.-T., Zhou G.-H. (2016). CPAP therapy reduces blood pressure for patients with obstructive sleep apnoea: An update meta-analysis of randomized clinical trials. Acta Cardiologica, 71(3), 275-280. http://dx.doi.org/10.2143/AC.71.3.3152087

| Section and topic | Item # | Checklist item | A | B | C | D |
| --- | --- | --- | --- | --- | --- | --- |
| **Title** | | | | | | |
| Title | 1 | Identify the report as a systematic review. | Y | Y | - | Y |
| **Abstract** | | | | | | |
| Abstract | 2 | See the PRISMA 2020 for Abstracts checklist (table 2). | PY | PY | - | PY |
| **Introduction** | | | | | | |
| Rationale | 3 | Describe the rationale for the review in the context of existing knowledge | PY | PY | - | PY |
| Objectives | 4 | Provide an explicit statement of the objective(s) or question(s) the review addresses. | PY | PY | - | PY |
| **Methods** | | | | | | |
| Eligibility criteria | 5 | Specify the inclusion and exclusion criteria for the review and how studies were grouped for the syntheses. | Y | Y | - | Y |
| Information sources | 6 | Specify all databases, registers, websites, organisations, reference lists and other sources searched or consulted to identify studies. Specify the date when each source was last searched or consulted. | Y | Y | - | Y |
| Search strategy | 7 | Present the full search strategies for all databases, registers and websites, including any filters and limits used | Y | Y | - | Y |
| Selection process | 8 | Specify the methods used to decide whether a study met the inclusion criteria of the review, including how many reviewers screened each record and each report retrieved, whether they worked independently, and if applicable, details of automation tools  used in the process. | Y | Y | - | Y |
| Data collection  process | 9 | Specify the methods used to collect data from reports, including how many reviewers collected data from each report, whether they worked independently, any processes for obtaining or confirming data from study investigators, and if applicable, details of automation tools used in the process. | Y | Y | - | Y |
| Data items | 10a | List and define all outcomes for which data were sought. Specify whether all results that were compatible with each outcome domain in each study were sought (e.g. for all measures, time points, analyses), and if not, the methods used to decide which  results to collect. | Y | Y | - | Y |
|  | 10b | List and define all other variables for which data were sought (e.g. participant and intervention characteristics, funding sources). Describe any assumptions made about any missing or unclear information. | Y | Y | - | Y |
| Study risk of bias  assessment | 11 | Specify the methods used to assess risk of bias in the included studies, including details of the tool(s) used, how many reviewers assessed each study and whether they worked independently, and if applicable, details of automation tools used in the process. | Y | Y | - | Y |
| Effect measures | 12 | Specify for each outcome the effect measure(s) (e.g. risk ratio, mean difference) used in the synthesis or presentation of results. | Y | Y | - | Y |
| Synthesis methods | 13a | Describe the processes used to decide which studies were eligible for each synthesis (e.g. tabulating the study intervention characteristics and comparing against the planned groups for each synthesis (item #5)). | Y | Y | - | Y |
|  | 13b | Describe any methods required to prepare the data for presentation or synthesis, such as handling of missing summary statistics, or data conversions. | Y | Y | - | Y |
|  | 13c | Describe any methods used to tabulate or visually display results of individual studies and syntheses. | Y | Y | - | Y |
|  | 13d | Describe any methods used to synthesise results and provide a rationale for the choice(s). If meta-analysis was performed, describe the model(s), method(s) to identify the presence and extent of statistical heterogeneity, and software package(s) used. | Y | Y | - | Y |
|  | 13e | Describe any methods used to explore possible causes of heterogeneity among study results (e.g. subgroup analysis, meta regression). | Y | Y | - | Y |
|  | 13f | Describe any sensitivity analyses conducted to assess robustness of the synthesised results. | PY | N | N | N |
| Reporting bias  assessment | 14 | Describe any methods used to assess risk of bias due to missing results in a synthesis (arising from reporting biases). | Y | Y | - | Y |
| Certainty assessment | 15 | Describe any methods used to assess certainty (or confidence) in the body of evidence for an outcome. | Y | Y | - | Y |
| **Results** | | | | | | |
| Study selection | 16a | Describe the results of the search and selection process, from the number of records identified in the search to the number of studies included in the review, ideally using a flow diagram (see fig 1). | Y | Y | - | Y |
|  | 16b | Cite studies that might appear to meet the inclusion criteria, but which were excluded, and explain why they were excluded. | Y | Y | - | Y |
| Study characteristics | 17 | Cite each included study and present its characteristics. | Y | Y | - | Y |
| Risk of bias in studies | 18 | Present assessments of risk of bias for each included study. | Y | Y | - | Y |
| Results of individual  studies | 19 | For all outcomes, present, for each study: (a) summary statistics for each group (where appropriate) and (b) an effect estimate and its precision (e.g. confidence/credible interval), ideally using structured tables or plots. | Y | Y | - | Y |
| Results of syntheses | 20a | For each synthesis, briefly summarise the characteristics and risk of bias among contributing studies. | Y | Y | - | Y |
|  | 20b | Present results of all statistical syntheses conducted. If meta-analysis was done, present for each the summary estimate and its precision (e.g. confidence/credible interval) and measures of statistical heterogeneity. If comparing groups, describe the direction of the effect. | Y | Y | - | Y |
|  | 20c | Present results of all investigations of possible causes of heterogeneity among study results. | Y | Y | - | Y |
|  | 20d | Present results of all sensitivity analyses conducted to assess the robustness of the synthesised results. | PY | PY | - | PY |
| Reporting biases | 21 | Present assessments of risk of bias due to missing results (arising from reporting biases) for each synthesis assessed. | Y | Y | - | Y |
| Certainty of evidence | 22 | Present assessments of certainty (or confidence) in the body of evidence for each outcome assessed. | Y | Y | - | Y |
| **Discussion** | | | | | | |
| Discussion | 23a | Provide a general interpretation of the results in the context of other evidence. | Y | Y | - | Y |
|  | 23b | Discuss any limitations of the evidence included in the review. | Y | Y | - | Y |
|  | 23c | Discuss any limitations of the review processes used. | Y | Y | - | Y |
|  | 23d | Discuss implications of the results for practice, policy, and future research. | Y | Y | - | Y |
| **Other information** | | | | | | |
| Registration and  protocol | 24a | Provide registration information for the review, including register name and registration number, or state that the review was not registered. | Y | Y | - | Y |
|  | 24b | Indicate where the review protocol can be accessed, or state that a protocol was not prepared. | Y | Y | - | Y |
|  | 24c | Describe and explain any amendments to information provided at registration or in the protocol. | PY | N | PY | PY |
| Support | 25 | Describe sources of financial or non-financial support for the review, and the role of the funders or sponsors in the review. | Y | Y | - | Y |
| Competing interests | 26 | Declare any competing interests of review authors. | Y | Y | - | Y |
| Availability of data,  code, and other  materials | 27 | Report which of the following are publicly available and where they can be found: template data collection forms; data extracted from included studies; data used for all analyses; analytic code; any other materials used in the review. | PY | Y | N | N |

Table 46：Labarca G., Schmidt A., Dreyse J., Jorquera J., Enos D., Torres G., Barbe F. (2021). Efficacy of continuous positive airway pressure (CPAP) in patients with obstructive sleep apnea (OSA) and resistant hypertension (RH): Systematic review and meta-analysis. Sleep Medicine Reviews, 58. http://dx.doi.org/10.1016/j.smrv.2021.101446

| Section and topic | Item # | Checklist item | A | B | C | D |
| --- | --- | --- | --- | --- | --- | --- |
| **Title** | | | | | | |
| Title | 1 | Identify the report as a systematic review. | Y | Y | - | Y |
| **Abstract** | | | | | | |
| Abstract | 2 | See the PRISMA 2020 for Abstracts checklist (table 2). | PY | PY | - | PY |
| **Introduction** | | | | | | |
| Rationale | 3 | Describe the rationale for the review in the context of existing knowledge | Y | Y | - | Y |
| Objectives | 4 | Provide an explicit statement of the objective(s) or question(s) the review addresses. | Y | Y | - | Y |
| **Methods** | | | | | | |
| Eligibility criteria | 5 | Specify the inclusion and exclusion criteria for the review and how studies were grouped for the syntheses. | Y | Y | - | Y |
| Information sources | 6 | Specify all databases, registers, websites, organisations, reference lists and other sources searched or consulted to identify studies. Specify the date when each source was last searched or consulted. | PY | Y | Y | Y |
| Search strategy | 7 | Present the full search strategies for all databases, registers and websites, including any filters and limits used | PY | Y | Y | Y |
| Selection process | 8 | Specify the methods used to decide whether a study met the inclusion criteria of the review, including how many reviewers screened each record and each report retrieved, whether they worked independently, and if applicable, details of automation tools  used in the process. | Y | Y | - | Y |
| Data collection  process | 9 | Specify the methods used to collect data from reports, including how many reviewers collected data from each report, whether they worked independently, any processes for obtaining or confirming data from study investigators, and if applicable, details of automation tools used in the process. | Y | Y | - | Y |
| Data items | 10a | List and define all outcomes for which data were sought. Specify whether all results that were compatible with each outcome domain in each study were sought (e.g. for all measures, time points, analyses), and if not, the methods used to decide which  results to collect. | PY | Y | Y | Y |
|  | 10b | List and define all other variables for which data were sought (e.g. participant and intervention characteristics, funding sources). Describe any assumptions made about any missing or unclear information. | PY | PY | Y | Y |
| Study risk of bias  assessment | 11 | Specify the methods used to assess risk of bias in the included studies, including details of the tool(s) used, how many reviewers assessed each study and whether they worked independently, and if applicable, details of automation tools used in the process. | Y | Y | - | Y |
| Effect measures | 12 | Specify for each outcome the effect measure(s) (e.g. risk ratio, mean difference) used in the synthesis or presentation of results. | Y | Y | - | Y |
| Synthesis methods | 13a | Describe the processes used to decide which studies were eligible for each synthesis (e.g. tabulating the study intervention characteristics and comparing against the planned groups for each synthesis (item #5)). | N | Y | Y | Y |
|  | 13b | Describe any methods required to prepare the data for presentation or synthesis, such as handling of missing summary statistics, or data conversions. | N | PY | Y | Y |
|  | 13c | Describe any methods used to tabulate or visually display results of individual studies and syntheses. | Y | Y | - | Y |
|  | 13d | Describe any methods used to synthesise results and provide a rationale for the choice(s). If meta-analysis was performed, describe the model(s), method(s) to identify the presence and extent of statistical heterogeneity, and software package(s) used. | Y | Y | - | Y |
|  | 13e | Describe any methods used to explore possible causes of heterogeneity among study results (e.g. subgroup analysis, meta regression). | Y | Y | - | Y |
|  | 13f | Describe any sensitivity analyses conducted to assess robustness of the synthesised results. | Y | Y | - | Y |
| Reporting bias  assessment | 14 | Describe any methods used to assess risk of bias due to missing results in a synthesis (arising from reporting biases). | Y | Y | - | Y |
| Certainty assessment | 15 | Describe any methods used to assess certainty (or confidence) in the body of evidence for an outcome. | Y | Y | - | Y |
| **Results** | | | | | | |
| Study selection | 16a | Describe the results of the search and selection process, from the number of records identified in the search to the number of studies included in the review, ideally using a flow diagram (see fig 1). | Y | Y | - | Y |
|  | 16b | Cite studies that might appear to meet the inclusion criteria, but which were excluded, and explain why they were excluded. | Y | Y | - | Y |
| Study characteristics | 17 | Cite each included study and present its characteristics. | Y | Y | - | Y |
| Risk of bias in studies | 18 | Present assessments of risk of bias for each included study. | Y | Y | - | Y |
| Results of individual  studies | 19 | For all outcomes, present, for each study: (a) summary statistics for each group (where appropriate) and (b) an effect estimate and its precision (e.g. confidence/credible interval), ideally using structured tables or plots. | Y | Y | - | Y |
| Results of syntheses | 20a | For each synthesis, briefly summarise the characteristics and risk of bias among contributing studies. | Y | Y | - | Y |
|  | 20b | Present results of all statistical syntheses conducted. If meta-analysis was done, present for each the summary estimate and its precision (e.g. confidence/credible interval) and measures of statistical heterogeneity. If comparing groups, describe the direction of the effect. | Y | Y | - | Y |
|  | 20c | Present results of all investigations of possible causes of heterogeneity among study results. | Y | Y | - | Y |
|  | 20d | Present results of all sensitivity analyses conducted to assess the robustness of the synthesised results. | Y | Y | - | Y |
| Reporting biases | 21 | Present assessments of risk of bias due to missing results (arising from reporting biases) for each synthesis assessed. | PY | Y | Y | Y |
| Certainty of evidence | 22 | Present assessments of certainty (or confidence) in the body of evidence for each outcome assessed. | Y | Y | - | Y |
| **Discussion** | | | | | | |
| Discussion | 23a | Provide a general interpretation of the results in the context of other evidence. | Y | Y | - | Y |
|  | 23b | Discuss any limitations of the evidence included in the review. | Y | Y | - | Y |
|  | 23c | Discuss any limitations of the review processes used. | N | Y | Y | Y |
|  | 23d | Discuss implications of the results for practice, policy, and future research. | Y | Y | - | Y |
| **Other information** | | | | | | |
| Registration and  protocol | 24a | Provide registration information for the review, including register name and registration number, or state that the review was not registered. | Y | Y | - | Y |
|  | 24b | Indicate where the review protocol can be accessed, or state that a protocol was not prepared. | N | Y | Y | Y |
|  | 24c | Describe and explain any amendments to information provided at registration or in the protocol. | N | PY | Y | Y |
| Support | 25 | Describe sources of financial or non-financial support for the review, and the role of the funders or sponsors in the review. | Y | Y | - | Y |
| Competing interests | 26 | Declare any competing interests of review authors. | Y | Y | - | Y |
| Availability of data,  code, and other  materials | 27 | Report which of the following are publicly available and where they can be found: template data collection forms; data extracted from included studies; data used for all analyses; analytic code; any other materials used in the review. | PY | PY | Y | Y |

Table 47：Shang W., Zhang Y., Liu L., Chen F., Wang G., Han D. (2022). Benefits of continuous positive airway pressure on blood pressure in patients with hypertension and obstructive sleep apnea: a meta-analysis. Hypertension Research, 45(11), 1802-1813. http://dx.doi.org/10.1038/s41440-022-00954-9

| Section and topic | Item # | Checklist item | A | B | C | D |
| --- | --- | --- | --- | --- | --- | --- |
| **Title** | | | | | | |
| Title | 1 | Identify the report as a systematic review. | N | Y | Y | Y |
| **Abstract** | | | | | | |
| Abstract | 2 | See the PRISMA 2020 for Abstracts checklist (table 2). | PY | PY | - | PY |
| **Introduction** | | | | | | |
| Rationale | 3 | Describe the rationale for the review in the context of existing knowledge | Y | Y | - | Y |
| Objectives | 4 | Provide an explicit statement of the objective(s) or question(s) the review addresses. | Y | Y | - | Y |
| **Methods** | | | | | | |
| Eligibility criteria | 5 | Specify the inclusion and exclusion criteria for the review and how studies were grouped for the syntheses. | PY | Y | Y | Y |
| Information sources | 6 | Specify all databases, registers, websites, organisations, reference lists and other sources searched or consulted to identify studies. Specify the date when each source was last searched or consulted. | PY | PY | - | PY |
| Search strategy | 7 | Present the full search strategies for all databases, registers and websites, including any filters and limits used | PY | N | Y | Y |
| Selection process | 8 | Specify the methods used to decide whether a study met the inclusion criteria of the review, including how many reviewers screened each record and each report retrieved, whether they worked independently, and if applicable, details of automation tools  used in the process. | Y | PY | Y | Y |
| Data collection  process | 9 | Specify the methods used to collect data from reports, including how many reviewers collected data from each report, whether they worked independently, any processes for obtaining or confirming data from study investigators, and if applicable, details of automation tools used in the process. | Y | PY | Y | Y |
| Data items | 10a | List and define all outcomes for which data were sought. Specify whether all results that were compatible with each outcome domain in each study were sought (e.g. for all measures, time points, analyses), and if not, the methods used to decide which  results to collect. | PY | Y | Y | Y |
|  | 10b | List and define all other variables for which data were sought (e.g. participant and intervention characteristics, funding sources). Describe any assumptions made about any missing or unclear information. | PY | PY | - | PY |
| Study risk of bias  assessment | 11 | Specify the methods used to assess risk of bias in the included studies, including details of the tool(s) used, how many reviewers assessed each study and whether they worked independently, and if applicable, details of automation tools used in the process. | Y | PY | Y | Y |
| Effect measures | 12 | Specify for each outcome the effect measure(s) (e.g. risk ratio, mean difference) used in the synthesis or presentation of results. | Y | Y | - | Y |
| Synthesis methods | 13a | Describe the processes used to decide which studies were eligible for each synthesis (e.g. tabulating the study intervention characteristics and comparing against the planned groups for each synthesis (item #5)). | N | N | - | N |
|  | 13b | Describe any methods required to prepare the data for presentation or synthesis, such as handling of missing summary statistics, or data conversions. | N | N | - | N |
|  | 13c | Describe any methods used to tabulate or visually display results of individual studies and syntheses. | Y | Y | - | Y |
|  | 13d | Describe any methods used to synthesise results and provide a rationale for the choice(s). If meta-analysis was performed, describe the model(s), method(s) to identify the presence and extent of statistical heterogeneity, and software package(s) used. | Y | Y | - | Y |
|  | 13e | Describe any methods used to explore possible causes of heterogeneity among study results (e.g. subgroup analysis, meta regression). | Y | Y | - | Y |
|  | 13f | Describe any sensitivity analyses conducted to assess robustness of the synthesised results. | Y | Y | - | Y |
| Reporting bias  assessment | 14 | Describe any methods used to assess risk of bias due to missing results in a synthesis (arising from reporting biases). | Y | Y | - | Y |
| Certainty assessment | 15 | Describe any methods used to assess certainty (or confidence) in the body of evidence for an outcome. | N | N | - | N |
| **Results** | | | | | | |
| Study selection | 16a | Describe the results of the search and selection process, from the number of records identified in the search to the number of studies included in the review, ideally using a flow diagram (see fig 1). | Y | Y | - | Y |
|  | 16b | Cite studies that might appear to meet the inclusion criteria, but which were excluded, and explain why they were excluded. | PY | N | N | N |
| Study characteristics | 17 | Cite each included study and present its characteristics. | Y | Y | - | Y |
| Risk of bias in studies | 18 | Present assessments of risk of bias for each included study. | Y | PY | Y | Y |
| Results of individual  studies | 19 | For all outcomes, present, for each study: (a) summary statistics for each group (where appropriate) and (b) an effect estimate and its precision (e.g. confidence/credible interval), ideally using structured tables or plots. | Y | Y | - | Y |
| Results of syntheses | 20a | For each synthesis, briefly summarise the characteristics and risk of bias among contributing studies. | Y | PY | Y | Y |
|  | 20b | Present results of all statistical syntheses conducted. If meta-analysis was done, present for each the summary estimate and its precision (e.g. confidence/credible interval) and measures of statistical heterogeneity. If comparing groups, describe the direction of the effect. | Y | Y | - | Y |
|  | 20c | Present results of all investigations of possible causes of heterogeneity among study results. | Y | Y | - | Y |
|  | 20d | Present results of all sensitivity analyses conducted to assess the robustness of the synthesised results. | Y | Y | - | Y |
| Reporting biases | 21 | Present assessments of risk of bias due to missing results (arising from reporting biases) for each synthesis assessed. | Y | Y | - | Y |
| Certainty of evidence | 22 | Present assessments of certainty (or confidence) in the body of evidence for each outcome assessed. | N | N | - | N |
| **Discussion** | | | | | | |
| Discussion | 23a | Provide a general interpretation of the results in the context of other evidence. | Y | Y | - | Y |
|  | 23b | Discuss any limitations of the evidence included in the review. | Y | Y | - | Y |
|  | 23c | Discuss any limitations of the review processes used. | N | Y | Y | Y |
|  | 23d | Discuss implications of the results for practice, policy, and future research. | Y | Y | - | Y |
| **Other information** | | | | | | |
| Registration and  protocol | 24a | Provide registration information for the review, including register name and registration number, or state that the review was not registered. | Y | Y | - | Y |
|  | 24b | Indicate where the review protocol can be accessed, or state that a protocol was not prepared. | N | N | - | N |
|  | 24c | Describe and explain any amendments to information provided at registration or in the protocol. | N | N | - | N |
| Support | 25 | Describe sources of financial or non-financial support for the review, and the role of the funders or sponsors in the review. | Y | Y | - | Y |
| Competing interests | 26 | Declare any competing interests of review authors. | Y | Y | - | Y |
| Availability of data,  code, and other  materials | 27 | Report which of the following are publicly available and where they can be found: template data collection forms; data extracted from included studies; data used for all analyses; analytic code; any other materials used in the review. | N | N | - | N |

ROBIS

P: Pass. F: Fail. L: Low risk of bias. H: High risk of bias. Un: Unclear risk of bias.

A:The conclusions of researcher Ph.D. Jingjing Liu.

B:The conclusions of researcher Ph.D. Yan Cui.

C:In case of a difference of opinions, it shall be adjudicated by Professor Yongshi Liu.

D:Conclusive conclusion.

Study 33：莫莉 & 何权瀛.(2007).长期持续气道正压通气对阻塞性睡眠呼吸暂停低通气综合征患者血压影响的荟萃分析.中华医学杂志,87(17),1177-1180.

| Phase | | A | B | C | D |
| --- | --- | --- | --- | --- | --- |
| Phase 1: Assessing Relevance | | P | P | - | P |
| Phase 2: Identifying Concerns with Review Process | Study Eligibility Criteria | L | L | - | L |
|  | Identification and Selection of Studies | UN | H | H | H |
|  | Data Collection and Study Appraisal | UN | UN | UN | UN |
|  | Synthesis and Findings | L | L | - | L |
| Phase 3: Judging Risk of Bias | | L | H | H | H |

Study 34：Bazzano L.A., Khan Z., Reynolds K., He J. (2007). Effect of nocturnal nasal continuous positive airway pressure on blood pressure in obstructive sleep apnea. Hypertension, 50(2), 417-423. http://dx.doi.org/10.1161/HYPERTENSIONAHA.106.085175

| Phase | | A | B | C | D |
| --- | --- | --- | --- | --- | --- |
| Phase 1: Assessing Relevance | | P | P | - | P |
| Phase 2: Identifying Concerns with Review Process | Study Eligibility Criteria | L | L | - | L |
|  | Identification and Selection of Studies | L | L | - | L |
|  | Data Collection and Study Appraisal | UN | UN | - | UN |
|  | Synthesis and Findings | L | L | - | L |
| Phase 3: Judging Risk of Bias | | L | L | - | L |

Study 35：Alajmi M, Mulgrew AT, Fox J, Davidson W, Schulzer M, Mak E, Ryan CF, Fleetham J, Choi P, Ayas NT. Impact of continuous positive airway pressure therapy on blood pressure in patients with obstructive sleep apnea hypopnea: a meta-analysis of randomized controlled trials. Lung. 2007 Mar-Apr;185(2):67-72. doi: 10.1007/s00408-006-0117-x. Epub 2007 Mar 28. PMID: 17393240.

| Phase | | A | B | C | D |
| --- | --- | --- | --- | --- | --- |
| Phase 1: Assessing Relevance | | P | P | - | P |
| Phase 2: Identifying Concerns with Review Process | Study Eligibility Criteria | L | L | - | L |
|  | Identification and Selection of Studies | L | L | - | L |
|  | Data Collection and Study Appraisal | UN | UN | - | UN |
|  | Synthesis and Findings | L | L | - | L |
| Phase 3: Judging Risk of Bias | | L | L | - | L |

Study 36：Haentjens P., Van Meerhaeghe A., Moscariello A., De Weerdt S., Poppe K., Dupont A., Velkeniers B. (2007). The impact of continuous positive airway pressure on blood pressure in patients with obstructive sleep apnea syndrome: Evidence from a meta-analysis of placebo-controlled randomized trials. Archives of Internal Medicine, 167(8), 757-765. http://dx.doi.org/10.1001/archinte.167.8.757

| Phase | | A | B | C | D |
| --- | --- | --- | --- | --- | --- |
| Phase 1: Assessing Relevance | | P | P | - | P |
| Phase 2: Identifying Concerns with Review Process | Study Eligibility Criteria | L | L | - | L |
|  | Identification and Selection of Studies | L | L | - | L |
|  | Data Collection and Study Appraisal | UN | UN | - | UN |
|  | Synthesis and Findings | L | L | - | L |
| Phase 3: Judging Risk of Bias | | L | L | - | L |

Study 37：王馨平.(2008).持续正压气道通气对阻塞性睡眠呼吸暂停低通气综合征中重度患者24小时动态血压影响的有效性评价(硕士学位论文,兰州大学).硕士https://kns.cnki.net/kcms2/article/abstract?v=9jT59j8Ji06nE4qyuijwTxro1nn4sKFzXfUXT9i1VmdWAeVIlWc11wPPQkxQv7rjvDTMjL6abWjLORwxIxfjQSf00UEFImXxhxZh2y9uZOuWYb1g0HeecOiawN0gY8_PKrWea6GL7VVWoIxHkXzJgVkm_i5v5KU6JvNEKox8me0kYqH0ECdvEQ==&uniplatform=NZKPT&language=CHS

| Phase | | A | B | C | D |
| --- | --- | --- | --- | --- | --- |
| Phase 1: Assessing Relevance | | P | P | - | P |
| Phase 2: Identifying Concerns with Review Process | Study Eligibility Criteria | L | L | - | L |
|  | Identification and Selection of Studies | L | L | - | L |
|  | Data Collection and Study Appraisal | L | UN | L | L |
|  | Synthesis and Findings | L | L | - | L |
| Phase 3: Judging Risk of Bias | | L | L | - | L |

Study 38：Montesi SB, Edwards BA, Malhotra A, Bakker JP. The effect of continuous positive airway pressure treatment on blood pressure: a systematic review and meta-analysis of randomized controlled trials. J Clin Sleep Med. 2012 Oct 15;8(5):587-96. doi: 10.5664/jcsm.2170. PMID: 23066375; PMCID: PMC3459209.

| Phase | | A | B | C | D |
| --- | --- | --- | --- | --- | --- |
| Phase 1: Assessing Relevance | | P | P | - | P |
| Phase 2: Identifying Concerns with Review Process | Study Eligibility Criteria | L | L | - | L |
|  | Identification and Selection of Studies | L | L | - | L |
|  | Data Collection and Study Appraisal | UN | UN | - | UN |
|  | Synthesis and Findings | L | L | - | L |
| Phase 3: Judging Risk of Bias | | L | L | - | L |

Study 39：尹富禹.(2013).持续气道正压通气治疗对阻塞性睡眠呼吸暂停综合症患者血压影响的Meta分析(硕士学位论文,重庆医科大学).硕士https://kns.cnki.net/kcms2/article/abstract?v=9jT59j8Ji07GJvRqP7QjpjiDaw-jA0h1ai-tr8BYJajFP31_bUzUPzkpsv0LekFbmNdEnS8Ai1TUf9ERpkq_kR5p8J6BT5_jzFYacmrQHDZmj5LeMpXUIBckm3pym6Te10sMgxTY_B9TslOBJKqpT5QiVi8y9eCBsO2D2sXmzSTpcXWsxIhAGg==&uniplatform=NZKPT&language=CHS

| Phase | | A | B | C | D |
| --- | --- | --- | --- | --- | --- |
| Phase 1: Assessing Relevance | | P | P | - | P |
| Phase 2: Identifying Concerns with Review Process | Study Eligibility Criteria | L | L | - | L |
|  | Identification and Selection of Studies | L | UN | H | H |
|  | Data Collection and Study Appraisal | UN | UN | H | H |
|  | Synthesis and Findings | L | L | - | L |
| Phase 3: Judging Risk of Bias | | L | UN | H | H |

Study 40：符翠萍,朱芬,刘子龙,励雯静,吴晓丹,吴旭... & 李善群.(2014).持续气道正压通气对阻塞性睡眠呼吸暂停低通气综合征患者血压影响的荟萃分析.世界临床药物,35(06),358-363.https://doi.org/10.13683/j.wph.2014.06.015.

| Phase | | A | B | C | D |
| --- | --- | --- | --- | --- | --- |
| Phase 1: Assessing Relevance | | P | P | - | P |
| Phase 2: Identifying Concerns with Review Process | Study Eligibility Criteria | L | L | - | L |
|  | Identification and Selection of Studies | L | UN | L | L |
|  | Data Collection and Study Appraisal | UN | UN | - | UN |
|  | Synthesis and Findings | L | L | - | L |
| Phase 3: Judging Risk of Bias | | L | UN | L | L |

Study 41：Schein A.S.O., Kerkhoff A.C., Coronel C.C., Plentz R.D.M., Sbruzzi G. (2014). Continuous positive airway pressure reduces blood pressure in patients with obstructive sleep apnea; A systematic review and meta-analysis with 1000 patients. Journal of Hypertension, 32(9), 1762-1773. http://dx.doi.org/10.1097/HJH.0000000000000250

| Phase | | A | B | C | D |
| --- | --- | --- | --- | --- | --- |
| Phase 1: Assessing Relevance | | P | P | - | P |
| Phase 2: Identifying Concerns with Review Process | Study Eligibility Criteria | L | L | - | L |
|  | Identification and Selection of Studies | L | L | - | L |
|  | Data Collection and Study Appraisal | L | UN | L | L |
|  | Synthesis and Findings | L | UN | L | L |
| Phase 3: Judging Risk of Bias | | L | UN | L | L |

Study 42：李君,李晓艳,蒋学俊,万为国 & 刘万里.(2015).持续气道正压通气对阻塞性睡眠呼吸暂停综合征患者降压疗效的Meta分析.海南医学,26(06),909-914.

| Phase | | A | B | C | D |
| --- | --- | --- | --- | --- | --- |
| Phase 1: Assessing Relevance | | P | P | - | P |
| Phase 2: Identifying Concerns with Review Process | Study Eligibility Criteria | L | L | - | L |
|  | Identification and Selection of Studies | L | UN | L | L |
|  | Data Collection and Study Appraisal | L | UN | L | L |
|  | Synthesis and Findings | L | UN | L | L |
| Phase 3: Judging Risk of Bias | | L | UN | L | L |

Study 43：Hu X., Fan J., Chen S., Yin Y., Zrenner B. (2015). The Role of Continuous Positive Airway Pressure in Blood Pressure Control for Patients With Obstructive Sleep Apnea and Hypertension: A Meta-Analysis of Randomized Controlled Trials. Journal of Clinical Hypertension, 17(3), 215-222. http://dx.doi.org/10.1111/jch.12472

| Phase | | A | B | C | D |
| --- | --- | --- | --- | --- | --- |
| Phase 1: Assessing Relevance | | P | P | - | P |
| Phase 2: Identifying Concerns with Review Process | Study Eligibility Criteria | L | L | - | L |
|  | Identification and Selection of Studies | L | UN | L | L |
|  | Data Collection and Study Appraisal | L | UN | L | L |
|  | Synthesis and Findings | L | L | - | L |
| Phase 3: Judging Risk of Bias | | L | L | - | L |

Study 44：Liu L., Cao Q., Guo Z., Dai Q. (2016). Continuous Positive Airway Pressure in Patients With Obstructive Sleep Apnea and Resistant Hypertension: A Meta-Analysis of Randomized Controlled Trials. Journal of Clinical Hypertension, 18(2), 153-158. http://dx.doi.org/10.1111/jch.12639

| Phase | | A | B | C | D |
| --- | --- | --- | --- | --- | --- |
| Phase 1: Assessing Relevance | | P | P | - | P |
| Phase 2: Identifying Concerns with Review Process | Study Eligibility Criteria | L | L | - | L |
|  | Identification and Selection of Studies | L | UN | UN | UN |
|  | Data Collection and Study Appraisal | L | UN | L | L |
|  | Synthesis and Findings | L | L | - | L |
| Phase 3: Judging Risk of Bias | | L | L | - | L |

Study 45：Sun Y., Huang Z.-Y., Sun Q.-R., Qiu L.-P., Zhou T.-T., Zhou G.-H. (2016). CPAP therapy reduces blood pressure for patients with obstructive sleep apnoea: An update meta-analysis of randomized clinical trials. Acta Cardiologica, 71(3), 275-280. http://dx.doi.org/10.2143/AC.71.3.3152087

| Phase | | A | B | C | D |
| --- | --- | --- | --- | --- | --- |
| Phase 1: Assessing Relevance | | P | P | - | P |
| Phase 2: Identifying Concerns with Review Process | Study Eligibility Criteria | L | L | - | L |
|  | Identification and Selection of Studies | L | UN | H | H |
|  | Data Collection and Study Appraisal | L | UN | UN | UN |
|  | Synthesis and Findings | L | L | - | L |
| Phase 3: Judging Risk of Bias | | L | L | - | L |

Study 46：Labarca G., Schmidt A., Dreyse J., Jorquera J., Enos D., Torres G., Barbe F. (2021). Efficacy of continuous positive airway pressure (CPAP) in patients with obstructive sleep apnea (OSA) and resistant hypertension (RH): Systematic review and meta-analysis. Sleep Medicine Reviews, 58. http://dx.doi.org/10.1016/j.smrv.2021.101446

| Phase | | A | B | C | D |
| --- | --- | --- | --- | --- | --- |
| Phase 1: Assessing Relevance | | P | P | - | P |
| Phase 2: Identifying Concerns with Review Process | Study Eligibility Criteria | L | L | - | L |
|  | Identification and Selection of Studies | L | L | - | L |
|  | Data Collection and Study Appraisal | L | L | - | L |
|  | Synthesis and Findings | L | L | - | L |
| Phase 3: Judging Risk of Bias | | L | L | - | L |

Study 47：Shang W., Zhang Y., Liu L., Chen F., Wang G., Han D. (2022). Benefits of continuous positive airway pressure on blood pressure in patients with hypertension and obstructive sleep apnea: a meta-analysis. Hypertension Research, 45(11), 1802-1813. http://dx.doi.org/10.1038/s41440-022-00954-9

| Phase | | A | B | C | D |
| --- | --- | --- | --- | --- | --- |
| Phase 1: Assessing Relevance | | P | P | - | P |
| Phase 2: Identifying Concerns with Review Process | Study Eligibility Criteria | L | L | - | L |
|  | Identification and Selection of Studies | L | UN | L | L |
|  | Data Collection and Study Appraisal | L | UN | UN | UN |
|  | Synthesis and Findings | L | L | - | L |
| Phase 3: Judging Risk of Bias | | L | UN | L | L |

Study 48：Benning L., Herzig J.J., Mollet M.S., Bradicich M., Pengo M.F., Ulrich S., Schwarz E.I. (2025). Effects of CPAP on Blood Pressure Parameter Across Different Severities of Obstructive Sleep Apnoea: A Meta-Analysis. Journal of Sleep Research, 34(5). http://dx.doi.org/10.1111/jsr.70072

| Phase | | A | B | C | D |
| --- | --- | --- | --- | --- | --- |
| Phase 1: Assessing Relevance | | P | P | - | P |
| Phase 2: Identifying Concerns with Review Process | Study Eligibility Criteria | L | L | - | L |
|  | Identification and Selection of Studies | L | L | - | L |
|  | Data Collection and Study Appraisal | L | L | - | L |
|  | Synthesis and Findings | L | L | - | L |
| Phase 3: Judging Risk of Bias | | L | L | - | L |

**Citation Overlap Matrix and the Corrected Covered Area (CCA)**

|  | 1 | 2 | 3 | 4 | 5 | 6 | 7 | 8 | 9 | 10 | 11 | 12 | 13 | 14 | 15 | 16 |
| --- | --- | --- | --- | --- | --- | --- | --- | --- | --- | --- | --- | --- | --- | --- | --- | --- |
| Engleman HM,19961 19:378-81. |  | √ |  | √ |  | √ |  |  |  |  |  |  |  |  |  | √ |
| Dimsdale JE,,2000 144-147 |  |  |  | √ | √ | √ |  |  | √ |  |  |  |  |  |  | √ |
| Barbe F,2001 134:1015-23 |  | √ | √ | √ | √ | √ |  | √ | √ |  |  |  |  |  |  | √ |
| Faccenda JF，2001 163(2):344–348 | √ | √ |  | √ |  | √ |  |  |  |  |  |  |  |  |  | √ |
| Monasterio C,2001 164:939-43 |  | √ | √ |  |  | √ |  | √ |  |  |  |  |  |  |  | √ |
| Barnes M,2002 165:773-780. | √ | √ | √ | √ |  | √ |  |  |  |  |  |  |  |  |  | √ |
| Pepperell JC，2002 359:204-10. | √ | √ | √ | √ | √ | √ | √ | √ | √ | √ |  |  |  |  |  | √ |
| Becker HF,2003 107:68-73 | √ | √ | √ | √ | √ | √ | √ |  | √ | √ |  |  |  |  |  | √ |
| Kaneko Y,2003 |  | √ | √ |  |  |  |  | √ |  |  |  |  |  |  |  | √ |
| Barnes M,2004 170:656-64 |  | √ | √ | √ |  | √ |  |  |  |  |  |  |  |  |  | √ |
| Coughlin S,2004 |  | √ |  |  |  |  |  |  |  |  |  |  |  |  |  | √ |
| Hermida RC,2004 | √ |  |  |  |  |  |  |  |  |  |  |  |  |  |  |  |
| Ip MS,2004 169:348 –353. |  | √ |  |  |  | √ |  |  |  |  |  |  |  |  |  | √ |
| Mansfield DR,2004 |  | √ |  |  |  |  |  |  |  |  |  |  |  |  |  | √ |
| Arias MA,2005 |  | √ |  |  |  |  |  |  |  |  |  |  |  |  |  |  |
| Patruno V，2005 20:107–116. |  |  |  |  |  |  |  |  | √ |  |  |  |  |  |  | √ |
| Usui K, 2005 |  |  | √ |  |  |  |  |  |  |  |  |  |  |  |  | √ |
| 林其昌，2005 |  |  |  |  |  |  |  |  |  | √ |  |  |  |  |  | √ |
| Alonso-Fernandez A,2006 27:207-15. |  |  |  |  |  | √ |  |  |  |  |  |  |  |  |  |  |
| AriasMA,,2006 |  |  |  | √ |  |  |  |  |  |  |  |  |  |  |  | √ |
| Campos-Rodiguez F,2006 129:1459-1467 | √ | √ | √ | √ |  | √ | √ | √ | √ | √ | √ |  |  |  | √ | √ |
| Hui DS，2006 61:1083-90 |  |  |  |  | √ | √ | √ |  | √ | √ |  |  |  |  | √ | √ |
| Mills PJ,2006 100:343-8 |  | √ |  |  | √ | √ |  | √ | √ |  |  |  |  |  |  | √ |
| Norman D,2006 47:840-5 |  |  |  | √ | √ | √ |  |  |  |  |  |  |  |  |  | √ |
| Robinson GV,2006 27:1229-35. | √ | √ | √ | √ | √ | √ | √ |  | √ |  | √ |  | √ |  | √ | √ |
| Coughlin SR,2007 29:720-7 |  |  |  |  |  | √ |  |  |  |  |  |  |  |  |  |  |
| Drager LF,2007 176:706-12. |  |  |  |  |  | √ |  |  |  |  |  |  |  |  |  |  |
| Lam B,2007 62:354-9. |  |  |  |  |  | √ |  |  |  |  |  |  |  |  |  | √ |
| Cross MD,2008 63:578-83. |  |  |  |  |  | √ |  |  | √ |  |  |  |  |  |  | √ |
| Kohler M,2008 32:1488-96 |  |  |  |  |  | √ |  |  | √ |  |  |  |  |  |  | √ |
| 刘冬生，2008 |  |  |  |  |  |  |  |  |  | √ |  |  |  |  |  | √ |
| Alonso-Fernandez A 2009 ;64:581-6 |  |  |  |  |  | √ |  |  | √ |  |  |  |  |  |  | √ |
| Comondore VR,2009 ;187:17-22 |  |  |  |  |  | √ |  |  |  |  |  |  |  |  |  | √ |
| 冯洁美，2009 8(1):105-106 |  |  |  |  |  |  |  | √ |  |  |  |  |  |  |  | √ |
| Oliveira W,2009 95:1872-8 |  |  |  |  |  | √ |  |  |  |  |  |  |  |  |  | √ |
| Barbe F,2010 181:718-26. |  |  |  |  |  | √ | √ |  |  |  |  |  |  |  | √ | √ |
| 李爱民，2010 19(2): 107-109 |  |  |  |  |  |  |  | √ |  |  |  |  |  |  |  | √ |
| Duran-Cantolla J,2010 ;341:c5991 |  |  |  |  |  | √ | √ |  | √ | √ | √ |  | √ |  | √ | √ |
| Lam JC,2010 35:138-45 |  |  |  |  |  | √ |  |  | √ |  |  |  |  |  |  | √ |
| Lozano L,2010 28:2161-8. |  |  |  |  |  | √ | √ |  |  | √ | √ | √ |  | √ | √ | √ |
| Nguyen PK,2010 12:50 |  |  |  |  |  | √ |  |  | √ |  |  |  |  |  |  | √ |
| 汪俊剑，2010 9(3): 246-248. |  |  |  |  |  |  |  |  |  | √ |  |  |  |  |  | √ |
| Drager LF,2011 ;57:549-55 |  |  |  |  |  | √ |  |  |  | √ |  |  | √ |  |  | √ |
| Kohler M,2011 184:1192-9 |  |  |  |  |  | √ |  |  | √ |  |  |  |  |  |  | √ |
| Sharma SK,2011 365:2277-86. |  |  |  |  |  | √ | √ |  |  |  |  |  |  |  |  | √ |
| 汪俊剑，2011 51(39): 93-94 |  |  |  |  |  |  |  | √ |  |  |  |  |  |  |  | √ |
| Craig SE，2012 |  |  |  |  |  |  |  |  |  |  |  |  |  |  |  |  |
| Hoyos CM 2012 |  |  |  |  |  |  |  |  |  |  |  |  |  |  |  | √ |
| Weaver TE 2012 |  |  |  |  |  |  |  |  |  |  |  |  |  |  |  |  |
| Zhao Q,2012 16(2):341-7 |  |  |  |  |  |  | √ |  |  |  |  |  |  |  |  | √ |
| Litvin AY,2013 ;9:229–35. |  |  |  |  |  |  |  |  |  |  |  |  |  |  | √ | √ |
| Martinez-Garcia，2013 310(22):2407-2415 |  |  |  |  |  |  |  |  |  |  | √ | √ | √ | √ | √ | √ |
| Pedrosa RP，2013 144(5)1487-1494 |  |  |  |  |  |  |  |  |  |  | √ | √ | √ | √ | √ | √ |
| 欧阳清彦，2013 |  |  |  |  |  |  |  |  |  | √ |  |  |  |  |  | √ |
| Ana Claudia de Oliveira,2014 |  |  |  |  |  |  |  |  |  |  |  | √ |  | √ |  | √ |
| Gottlieb DJ, 2014 370: 2276-85. |  |  |  |  |  |  |  |  |  |  |  |  | √ |  |  | √ |
| Lloberes P,2014 1657 |  |  |  |  |  |  |  |  |  |  | √ |  | √ | √ | √ | √ |
| McMillan A，2014 |  |  |  |  |  |  |  |  |  |  |  |  | √ |  |  | √ |
| Martinez-Garcia MA 2015 |  |  |  |  |  |  |  |  |  |  |  |  |  |  |  | √ |
| Elizabeth A Muxfeldt,2015 |  |  |  |  |  |  |  |  |  |  |  | √ | √ | √ | √ | √ |
| Huang Z,2015 28: 300-6. |  |  |  |  |  |  |  |  |  |  |  |  | √ |  | √ | √ |
| Hoyos CM,2015 10: 1222-8 |  |  |  |  |  |  |  |  |  |  |  |  | √ |  |  | √ |
| Pamidi S 2015 |  |  |  |  |  |  |  |  |  |  |  |  |  |  |  | √ |
| Parra O,2015 24: 47-53. |  |  |  |  |  |  |  |  |  |  |  |  | √ |  |  | √ |
| Paz y Mar H 2016 |  |  |  |  |  |  |  |  |  |  |  |  |  |  |  | √ |
| McEvoy RD 2016 |  |  |  |  |  |  |  |  |  |  |  |  |  |  |  | √ |
| Salord N 2016 |  |  |  |  |  |  |  |  |  |  |  |  |  |  |  | √ |
| Shaw JE 2016 |  |  |  |  |  |  |  |  |  |  |  |  |  |  |  | √ |
| Thunström E, 2016 ;193:310–20. |  |  |  |  |  |  |  |  |  |  |  |  |  |  | √ | √ |
| Campos-Rodriguez F,2017 |  |  |  |  |  |  |  |  |  |  |  |  |  |  |  | √ |
| Casitas R,2017 50:1701261. |  |  |  |  |  |  |  |  |  |  |  |  |  |  | √ | √ |
| Lam JCM 2017 |  |  |  |  |  |  |  |  |  |  |  |  |  |  |  | √ |
| Gupta A 2018 |  |  |  |  |  |  |  |  |  |  |  |  |  |  |  | √ |
| Joyeux-Faure M,2018 9:318. |  |  |  |  |  |  |  |  |  |  |  |  |  | √ | √ | √ |
| Zou BG X,2018 11(11):11965e72. |  |  |  |  |  |  |  |  |  |  |  |  |  | √ | √ | √ |
| Javaheri S 2019 |  |  |  |  |  |  |  |  |  |  |  |  |  |  |  | √ |
| Lui MMS 2020 Sep;24(3):817-824. |  |  |  |  |  |  |  |  |  |  |  |  |  |  |  | √ |
| Ponce S 2019 |  |  |  |  |  |  |  |  |  |  |  |  |  |  |  | √ |
| Chen Q，2020 29:21–30. |  |  |  |  |  |  |  |  |  |  |  |  |  |  | √ | √ |
| Ruzicka M,2020 ;2:258–64. |  |  |  |  |  |  |  |  |  |  |  |  |  |  | √ | √ |
| Sánchez-de-la-Torre M 2020 |  |  |  |  |  |  |  |  |  |  |  |  |  |  |  | √ |
| Lui MM,2021 58:2003687. |  |  |  |  |  |  |  |  |  |  |  |  |  |  | √ | √ |

**Literature Screening**

**animal research**

| 常悦 | 2016 | 丹参酮ⅡA对慢性间歇性低氧所致高血压大鼠心肌ET-1及其受体的影响 硕士, 河北医科大学 |
| --- | --- | --- |

**Non-hypertension**

|  | 2006 | Polysomnography in patients with obstructive sleep apnea: an evidence-based analysis |
| --- | --- | --- |
|  | 2011 | Effects of an In-Patient Individualized Rehabilitation Program on Severity and Symptoms of Obstructive Sleep Apnea Syndrome (OSAS):Results of a Randomized Controlled Pilot Study. |
|  | 2014 | The Phenotyping and Genotyping of Taiwanese Patients With Obstructive Sleep Apnea. |
|  | 2017 | Effect of Continuous Positive Airway Pressure (CPAP) Treatment on Cognitive Ability in HIV+ Individuals With Obstructive Sleep Apnea (OSA): A Pilot Study. |
|  | 2021 | Brain Tissue Integrity and Autonomic Function Alterations in Childhood Obstructive Sleep Apnea and Attention-deficit/Hyperactivity Disorder, and After Adenotonsillectomy. |
|  | 2022 | The Efficacy of Continuous Positive Airway Pressure in Achieving Asthma Control in Patients With Severe Asthma and Obstructive Sleep Apnea. |
|  | 2023 | The Effect of Zolpidem on CPAP Acclimatization in Patients With OSA: A Crossover, Randomized, Double-blinded, Placebo-controlled Trial. |
| Abuzaid, A. S., H. S. Al Ashry, A. Elbadawi, H. Ld, M. Saad, I. Y. Elgendy, A. Elgendy, A. N. Mahmoud, A. Mentias, A. Barakat and C. Lal | 2017 | Meta-Analysis of Cardiovascular Outcomes With Continuous Positive Airway Pressure Therapy in Patients With Obstructive Sleep Apnea |
| Aggarwal, S., R. Nadeem, R. S. Loomba, M. Nida and D. Vieira | 2014 | The Effects of Continuous Positive Airways Pressure Therapy on Cardiovascular End Points in Patients With Sleep-Disordered Breathing and Heart Failure: A Meta-Analysis of Randomized Controlled Trials |
| Ayappa, I., J. Sunderram, K. Black, A. Twumasi, I. Udasin, D. Harrison, J. L. Carson, S. E. Lu and D. M. Rapoport | 2015 | A comparison of CPAP and CPAPFLEX in the treatment of obstructive sleep apnea in World Trade Center responders: study protocol for a randomized controlled trial |
| Balk, E. M., G. P. Adam, W. Cao, M. Reddy Bhuma, C. D’Ambrosio and T. A. Trikalinos | 2024 | Long-term effects on clinical event, mental health, and related outcomes of CPAP for obstructive sleep apnea: a systematic review |
| Basoglu, O. K. | 2020 | Hyperlipidaemia and OSA |
| Bazan, V., I. Vicente, L. Lozano, R. Villuendas, M. González, R. Adeliño, F. Bisbal, A. Sarrias, J. Abad, J. Sanz-Santos, F. Padilla and A. Bayés-Genís | 2021 | Previously Undetected Obstructive Sleep Apnea in Patients With New-Onset Atrial Fibrillation |
| Berezin, L., M. Nagappa, K. Poorzargar, A. Saripella, J. Ariaratnam, N. Butris, M. Englesakis and F. Chung | 2023 | The effectiveness of positive airway pressure therapy in reducing postoperative adverse outcomes in surgical patients with obstructive sleep apnea: A systematic review and meta-analysis |
| Berger, M., G. Solelhac, C. Horvath, R. Heinzer and A. K. Brill | 2021 | Treatment-emergent central sleep apnea associated with non-positive airway pressure therapies in obstructive sleep apnea patients: A systematic review |
| Bilal, N., N. Dikmen, F. Bozkus, A. Sungur, S. Sarica, I. Orhan and A. Samur | 2018 | Obstructive sleep apnea is associated with increased QT corrected interval dispersion: the effects of continuous positive airway pressure |
| Borel, A. L., R. Tamisier, P. Böhme, P. Priou, A. Avignon, P. Y. Benhamou, H. Hanaire, J. L. Pépin, L. Kessler, P. Valensi, P. Darmon and F. Gagnadoux | 2019 | Obstructive sleep apnoea syndrome in patients living with diabetes: Which patients should be screened? |
| Bravata, D. M., V. McClain, C. Austin, J. Ferguson, N. Burrus, E. J. Miech, M. S. Matthias, N. Chumbler, S. Ofner, B. Foresman, J. Sico, C. A. V. Fragoso, L. S. Williams, R. Agarwal, J. Concato and H. K. Yaggi | 2017 | Diagnosing and managing sleep apnea in patients with chronic cerebrovascular disease: a randomized trial of a home-based strategy |
| Bretton, D. J., T. Gaisl, C. Schlatzer and M. Kohler | 2015 | Comparison of the effects of continuous positive airway pressure and mandibular advancement devices on sleepiness in patients with obstructive sleep apnoea: a network meta-analysis |
| Brozyna-Tkaczyk, K., W. Myslinski and J. Mosiewicz | 2021 | The Assessment of Endothelial Dysfunction among OSA Patients after CPAP Treatment |
| Bubu, O. M., A. G. Andrade, O. Q. Umasabor-Bubu, M. M. Hogan, A. D. Turner, M. J. de Leon, G. Ogedegbe, I. Ayappa, G. Jean-Louis, M. L. Jackson, A. W. Varga and R. S. Osorio | 2020 | Obstructive sleep apnea, cognition and Alzheimer's disease: A systematic review integrating three decades of multidisciplinary research |
| Cabral, T. D. D., R. M. Knoll, J. Plane, P. Viana, B. D. Vendramini, J. E. P. Conrado, V. Caruso and P. Huyett | 2025 | Positive Airway Pressure Therapy for Obstructive Sleep Apnea in High-Risk Pregnancy: A Meta-Analysis |
| Cattazzo, F., M. F. Pengo, A. Giontella, D. Soranna, J. Karalliedde, L. Gnudi, P. Minuz, C. Lombardi, G. Parati and C. Fava | 2021 | Effect of continuous positive airway pressure treatment on glycemic and lipid profiles in patients with obstructive sleep APNEA: A systematic review and meta-analysis |
| Chaiard, J. and T. E. Weaver | 2019 | Update on Research and Practices in Major Sleep Disorders: Part I. Obstructive Sleep Apnea Syndrome |
| Chalegre, S. T., O. L. Lins, T. C. Lustosa, M. V. França, T. L. G. Couto, L. F. Drager, G. Lorenzi, M. S. Bittencourt and R. P. Pedrosa | 2021 | Impact of CPAP on arterial stiffness in patients with obstructive sleep apnea: a meta-analysis of randomized trials |
| Chen, B. X., M. L. Guo, Y. Peker, N. Salord, L. F. Drager, G. Lorenzi, X. D. Tang and Y. Li | 2022 | Effect of Continuous Positive Airway Pressure on Lipid Profiles in Obstructive Sleep Apnea: A Meta-Analysis |
| Cheong, A. J. Y., S. K. X. Wang, C. Y. Woon, K. H. Yap, K. J. Y. Ng, F. W. X. Xu, U. Alkan, A. C. W. Ng, A. See, S. R. H. Loh, T. Aung and S. T. Toh | 2023 | Obstructive sleep apnoea and glaucoma: a systematic review and meta-analysis |
| Chou, K. T., C. C. Huang, Y. M. Chen, K. C. Su, G. M. Shiao, Y. C. Lee, W. L. Chan and H. B. Leu | 2012 | Sleep Apnea and Risk of Deep Vein Thrombosis: A Non-randomized, Pair-matched Cohort Study |
| Clerget, A., A. Kanbar and M. Abdessater | 2020 | Urinary tract symptoms and erectile dysfunction in obstructive sleep apnea: Systematic review |
| Corso, R., V. Russotto, C. Gregoretti and D. Cattano | 2018 | Perioperative management of obstructive sleep apnea: a systematic review |
| Cuspidi, C., M. Tadic, C. Sala, E. Gherbesi, G. Grassi and G. Mancia | 2020 | Obstructive sleep apnoea syndrome and left ventricular hypertrophy: a meta-analysis of echocardiographic studies |
| De Luca, M., M. Zese, G. A. Silverii, B. Ragghianti, G. Bandini, P. Forestieri, M. A. Zappa, G. Navarra, D. Foschi, M. Musella, G. Sarro, V. Pilone, E. Facchiano, M. Foletto, S. Olmi, M. Raffelli, R. Bellini, P. Gentileschi, M. R. Cerbone, I. Grandone, G. Berardi, N. Di Lorenzo, M. Lucchese, L. Piazza, G. Casella, E. Manno, A. Zaccaroni, A. Balani, E. Mannucci and M. Monami | 2023 | Bariatric Surgery for Patients with Overweight/Obesity. A Comprehensive Grading Methodology and Network Metanalysis of Randomized Controlled Trials on Weight Loss Outcomes and Adverse Events |
| Deng, G., Z. D. Qiu, D. Y. Li, Y. Fang and S. M. Zhang | 2016 | Effects of continuous positive airway pressure therapy on plasma aldosterone levels in patients with obstructive sleep apnea: A meta-analysis |
| Di Lorenzo, B., C. Scala, A. A. Mangoni, S. Zoroddu, P. Paliogiannis, P. Pirina, A. G. Fois, C. Carru and A. Zinellu | 2024 | A Systematic Review and Meta-Analysis of Mean Platelet Volume and Platelet Distribution Width in Patients with Obstructive Sleep Apnoea Syndrome |
| Dipalma, G., A. M. Inchingolo, P. Avantario, A. Mancini, M. Campanelli, D. Di Venere, A. Palermo, F. Inchingolo, M. Corsalini and A. D. Inchingolo | 2025 | Comparative Efficacy of Continuous Positive Airway Pressure and Mandibular Advancement Devices in the Treatment of Obstructive Sleep Apnea: A Systematic Review |
| Doonan, R. J., P. Scheffler, M. Lalli, R. J. Kimoff, E. T. Petridou, M. E. Daskalopoulos and S. S. Daskalopoulou | 2011 | Increased arterial stiffness in obstructive sleep apnea: a systematic review |
| Drager, L. F., T. M. Tavoni, V. M. Silva, R. D. Santos, R. P. Pedrosa, L. A. Bortolotto, C. G. Vinagre, V. Y. Polotsky, G. Lorenzi and R. C. Maranhao | 2018 | Obstructive sleep apnea and effects of continuous positive airway pressure on triglyceride-rich lipoprotein metabolism |
| Duss, S. B., A. K. Brill, S. Baillieul, T. Horvath, F. Zubler, D. Flügel, G. Kägi, G. Benz, C. Bernasconi, S. R. Ott and et al. | 2021 | Effect of early sleep apnoea treatment with adaptive servo-ventilation in acute stroke patients on cerebral lesion evolution and neurological outcomes: study protocol for a multicentre, randomized controlled, rater-blinded, clinical trial (eSATIS: early Sleep Apnoea Treatment in Stroke) |
| Ephros, H. D., M. Madani and S. C. Yalamanchili | 2010 | Surgical treatment of snoring & obstructive sleep apnoea |
| Fan, J., C. J. Tan, L. L. Cao, S. L. Luo, S. X. Sun, S. H. Wang, W. J. Li, Y. C. Pan, T. Y. Wu, Q. S. Dai, Z. Liu, J. Liu and B. B. Yu | 2025 | The impact of continuous positive airway pressure therapy on the recurrence of atrial fibrillation in patients with obstructive sleep apnea after pulmonary vein isolation |
| Fu, N. Y., X. T. Tan and J. He | 2025 | Association between elevated cystatin C levels and obstructive sleep apnea hypopnea syndrome: a systematic review and updated meta-analysis |
| Gagnadoux, F., M. Jouvenot, N. Meslier, P. Priou and W. Trzepizur | 2017 | Therapeutic alternatives to continuous positive airway pressure for obstructive sleep apnea-hypopnea syndrome |
| Golshah, A., M. M. Imani, M. Sadeghi, M. K. Chalkhooshg, A. B. Bruehl, L. S. Bahmani and S. Brand | 2023 | Effect of Continuous Positive Airway Pressure on Changes of Plasma/Serum Ghrelin and Evaluation of These Changes between Adults with Obstructive Sleep Apnea and Controls: A Meta-Analysis |
| Grewal, N., D. Gordon, S. Bajaj, C. Gyimah, M. Hassan, U. Fatima and P. P. Mehrotra | 2024 | Impact of Obstructive Sleep Apnea Treatment on Cardiovascular Disease Associated Mortality and Morbidity: A Systematic Review |
| Guggino, J., R. Tamisier, C. Betry, S. Coumes, C. Arvieux, N. Wion, F. Reche, J. L. Pépin and A. L. Borel | 2021 | Bariatric surgery short-term outcomes in patients with obstructive sleep apnoea: the Severe Obesity Outcome Network prospective cohort |
| Guimaraes, T. M., D. Poyares, L. O. E. Silva, G. Luz, G. Coelho, C. Dal Fabbro, S. Tufik and L. Bittencourt | 2021 | The treatment of mild OSA with CPAP or mandibular advancement device and the effect on blood pressure and endothelial function after one year of treatment |
| Hamilton, G. S. and S. A. Joosten | 2017 | Obstructive sleep apnoea and obesity |
| He, H., T. Lachlan and F. Osman | 2024 | Treated obstructive sleep apnoea and incident arrhythmias |
| Hwang, D., J. W. Chang, A. V. Benjafield, M. E. Crocker, C. Kelly, K. A. Becker, J. B. Kim, R. R. Woodrum, J. Liang and S. F. Derose | 2018 | Effect of Telemedicine Education and Telemonitoring on Continuous Positive Airway Pressure Adherence The Tele-OSA Randomized Trial |
| Iftikhar, I. H., L. Bittencourt, S. D. Youngstedt, N. Ayas, P. Cistulli, R. Schwab, M. W. Durkin and U. J. Magalang | 2017 | Comparative efficacy of CPAP, MADs, exercise-training, and dietary weight loss for sleep apnea: a network meta-analysis |
| Iftikhar, I. H., C. W. Valentine, L. R. Bittencourt, D. L. Cohen, A. C. Fedson, T. Gíslason, T. Penzel, C. L. Phillips, L. Yu-sheng, A. I. Pack and U. J. Magalang | 2014 | Effects of continuous positive airway pressure on blood pressure in patients with resistant hypertension and obstructive sleep apnea: a meta-analysis |
| Imran, T. F., M. Ghazipura, S. Liu, T. Hossain, H. Ashtyani, B. Kim, J. Michael Gaziano and L. Djoussé | 2016 | Effect of continuous positive airway pressure treatment on pulmonary artery pressure in patients with isolated obstructive sleep apnea: a meta-analysis |
| Juric, J. S., S. Juric and I. Markovic | 2022 | INFLUENCE OF CONTINUOUS POSITIVE AIRWAY PRESSURE TREATMENT ON AUDITORY EVENT-RELATED POTENTIALS P300 |
| Khan, S. U., C. A. Duran, H. Rahman, M. Lekkala, M. A. Saleem and E. Kaluski | 2018 | A meta-analysis of continuous positive airway pressure therapy in prevention of cardiovascular events in patients with obstructive sleep apnoea |
| Kotha, S., K. Joshi, M. Pappuswamy and G. Meganathan | 2025 | Impact of sleep disorders on mental health: A systematic review |
| Labarca, G., R. Cruz and J. Jorquera | 2018 | Continuous Positive Airway Pressure in Patients With Obstructive Sleep Apnea and Non-Alcoholic Steatohepatitis: A Systematic Review and Meta-Analysis |
| Labarca, G., T. Reyes, J. Jorquera, J. Dreyse and L. Drake | 2018 | CPAP in patients with obstructive sleep apnea and type 2 diabetes mellitus: Systematic review and meta-analysis |
| Li, H., Y. Y. Pan, Y. K. Lou, Y. J. Zhang, L. R. Yin, J. E. Sanderson and F. Fang | 2022 | The Effects of Continuous Positive Airway Pressure Therapy for Secondary Cardiovascular Prevention in Patients with Obstructive Sleep Apnoea: A Systematic Review and Meta-Analysis |
| Li, X., X. Zhou, X. Xu, J. Dai, C. Chen, L. Ma, J. Li, W. Mao and M. Zhu | 2021 | Effects of continuous positive airway pressure treatment in obstructive sleep apnea patients with atrial fibrillation: A meta-analysis |
| Lin, M. T., P. L. Lee, C. W. Yu, T. F. Shih, W. D. Wu, W. Y. Shau, H. J. Hsu, K. C. Huang, C. L. Chen, C. T. Tang and et al. | 2019 | The effect of continuous positive airway pressure treatment on abdominal adiposity in severe obstructive sleep apnea: evidence from randomized, active controlled trials |
| Liu, T., Y. Zhan, Y. Wang, Q. Li and H. Mao | 2021 | Obstructive sleep apnea syndrome and risk of renal impairment: a systematic review and meta-analysis with trial sequential analysis |
| Louise, B. J., F. Carys and M. Philip | 2020 | Narrative review of sleep and pulmonary hypertension |
| Madbouly, E. M., R. Nadeem, M. Nida, J. Molnar, S. Aggarwal and R. Loomba | 2014 | The role of severity of obstructive sleep apnea measured by apnea-hypopnea index in predicting compliance with pressure therapy, a meta-analysis |
| Mayer, G., H. Frohnhofen, M. Jokisch, D. M. Hermann and J. Gronewold | 2024 | Associations of sleep disorders with all-cause MCI/dementia and different types of dementia – clinical evidence, potential pathomechanisms and treatment options: A narrative review |
| McDaid, C., K. H. Durée, S. C. Griffin, H. L. Weatherly, J. R. Stradling, R. J. Davies, M. J. Sculpher and M. E. Westwood | 2009 | A systematic review of continuous positive airway pressure for obstructive sleep apnoea-hypopnoea syndrome |
| Migueis, D., A. Urel, C. C. Santos, A. Accetta and M. Burla | 2022 | The cardiovascular, metabolic, fetal and neonatal effects of CPAP use in pregnant women: a systematic review |
| Mihaela, V., E. Bendelic, A. Bikov, A. Ceasovschih and C. Alexandru | 2025 | BURDEN OF OPHTHALMOLOGIC DISORDERS IN OBSTRUCTIVE SLEEP APNEA |
| Miller, M. A. and F. P. Cappuccio | 2021 | A systematic review of COVID-19 and obstructive sleep apnoea |
| Mineiro, M. A., P. M. da Silva, M. Alves, A. L. Papoila, M. J. M. Gomes and J. Cardoso | 2017 | The role of sleepiness on arterial stiffness improvement after CPAP therapy in males with obstructive sleep apnea: a prospective cohort study |
| Mineiro, M. A., P. M. da Silva, M. Alves, D. Virella, M. J. M. Gomes and J. Cardoso | 2016 | Use of CPAP to reduce arterial stiffness in moderate-to-severe obstructive sleep apnoea, without excessive daytime sleepiness (STIFFSLEEP): an observational cohort study protocol |
| Mir, E., A. Rehman, A. Ahad, J. A. Malik and S. Hamid | 2024 | Obstructive Sleep Apnoea: A Systematic Review of Advances in the Last 1 Year |
| Mok, Y., C. W. Tan, H. S. Wong, C. H. How, K. L. A. Tan and P. P. Hsu | 2017 | Obstructive sleep apnoea and Type 2 diabetes mellitus: are they connected? |
| Mokhlesi, B., J. F. Masa, J. L. Brozek, I. Gurubhagavatula, P. B. Murphy, A. J. Piper, A. Tulaimat, M. Afshar, J. S. Balachandran, R. A. Dweik, R. R. Grunstein, N. Hart, R. Kaw, G. Lorenzi, S. Pamidi, B. K. Patel, S. P. Patil, J. L. Pépin, I. Soghier, M. T. Kakazu, M. Teodorescu and R. Amer Thoracic Soc Assembly Sleep | 2019 | Evaluation and Management of Obesity Hypoventilation Syndrome An Official American Thoracic Society Clinical Practice Guideline |
| Nct | 2008 | Effects of CPAP on Cardiovascular Risk in Obstructive Sleep Apnea (OSA) and Metabolic Syndrome |
| Nct | 2022 | Effects of Continuous Positive Airway Pressure Therapy Withdrawal in Patients With Obstructive Sleep Apnea: a Randomized Trial |
| Nugent, R., A. Wee, L. Kearney and C. de Costa | 2023 | The effectiveness of continuous positive airway pressure for treating obstructive sleep apnoea in pregnancy: A systematic review |
| Pan, Y.-Y., Y. Deng, X. Xu, Y.-P. Liu and H.-G. Liu | 2015 | Effects of Continuous Positive Airway Pressure on Cognitive Deficits in Middle-aged Patients with Obstructive Sleep Apnea Syndrome:A Meta-analysis of Randomized Controlled Trials |
| Patil, S. P., M. E. Billings, G. Bourjeily, N. A. Collop, D. J. Gottlieb, K. G. Johnson, R. J. Kimoff and A. I. Pack | 2024 | Long-term health outcomes for patients with obstructive sleep apnea: placing the Agency for Healthcare Research and Quality report in context-a multisociety commentary |
| Pearse, S. G. and M. R. Cowie | 2016 | Sleep-disordered breathing in heart failure |
| Ruan, B. D., M. Nagappa, M. Rashid-Kolvear, K. V. Zhang, R. Waseem, M. Englesakis and F. C. Chung | 2023 | The effectiveness of supplemental oxygen and high-flow nasal cannula therapy in patients with obstructive sleep apnea in different clinical settings: A systematic review and meta-analysis |
| Sanka, S., H. Moparty, S. Kaur, S. Singh, P. K. Devarakonda, P. Kamani, I. Iyer and S. Reddy | 2024 | A CASE OF S1S2S3 SYNDROME ASSOCIATED WITH OBSTRUCTIVE SLEEP APNEA. |
| Sarvananda, S., S. Earnshaw, I. Hughes, P. Sivakumaran and K. B. Sriram | 2025 | Effect of positive airway pressure treatment on pulmonary artery pressure in obstructive sleep apnoea and/or obesity hypoventilation syndrome with pulmonary hypertension: a systematic review and meta-analysis |
| Sheth, U., R. S. Monson, B. Prasad, A. S. Sahni, S. Matani, T. Mercado, M. A. Smith, M. A. Carlucci, K. K. Danielson and S. Reutrakul | 2021 | Association of continuous positive airway pressure adherence with complications in patients with type 2 diabetes and obstructive sleep apnea |
| Simon, B., B. Gabor, I. Barta, C. Paska, G. B. Nagy, E. Vizi and B. Antus | 2020 | Effect of 5-year continuous positive airway pressure treatment on the lipid profile of patients with obstructive sleep apnea: A pilot study |
| Singh, M., K. Deokar, S. Dutta, B. P. Sinha and C. D. S. Katoch | 2025 | Impact of positive airway pressure therapy on intraocular pressure in obstructive sleep apnea: A systematic review |
| Sun, X., J. Luo and Y. Wang | 2021 | Comparing the effects of supplemental oxygen therapy and continuous positive airway pressure on patients with obstructive sleep apnea: a meta-analysis of randomized controlled trials |
| Sun, X., J. Luo and Y. Xiao | 2014 | Continuous positive airway pressure is associated with a decrease in pulmonary artery pressure in patients with obstructive sleep apnoea: a meta-analysis |
| Tadic, M., E. Gherbesi, A. Faggiano, C. Sala, S. Carugo and C. Cuspidi | 2022 | The impact of continuous positive airway pressure on cardiac mechanics: Findings from a meta-analysis of echocardiographic studies |
| Tan, E. S. J. and C. H. Lee | 2021 | Obstructive Sleep Apnea and Arrhythmias in the Elderly |
| Tanayapong, P. and S. T. Kuna | 2021 | Sleep disordered breathing as a cause and consequence of stroke: A review of pathophysiological and clinical relationships |
| Teo, Y. H., R. B. Han, S. Leong, Y. N. Teo, N. L. Syn, C. F. Wee, B. K. J. Tan, R. C. C. Wong, P. Chai, P. Kojodjojo, W. K. Kong, C. H. Lee, C. H. Sia and T. C. Yeo | 2022 | Prevalence, types and treatment of bradycardia in obstructive sleep apnea - A systematic review and meta-analysis |
| Truong, K. and C. Guilleminault | 2018 | Sleep disordered breathing in pregnant women: maternal and fetal risk, treatment considerations, and future perspectives |
| Wang, X., Y. Zhang, Z. M. Dong, J. Y. Fan, S. P. Nie and Y. X. Wei | 2018 | Effect of continuous positive airway pressure on long-term cardiovascular outcomes in patients with coronary artery disease and obstructive sleep apnea: a systematic review and meta-analysis |
| Wang, Y., Y. N. Lin, L. Y. Zhang, C. X. Li, S. Q. Li, H. P. Li, L. Zhang, N. Li, Y. R. Yan and Q. Y. Li | 2022 | Changes of circulating biomarkers of inflammation and glycolipid metabolism by CPAP in OSA patients: a meta-analysis of time-dependent profiles |
| Wang, Y. X., J. M. Luo, R. Huang and Y. Xiao | 2023 | [Continuous positive airway pressure therapy affects the recurrence of atrial fibrillation in patients with obstructive sleep apnea: a systematic review and meta-analysis] |
| Wu, Q. Q., X. J. Ma, Y. Y. Wang, J. F. Jin, J. Li and S. M. Guo | 2023 | Efficacy of continuous positive airway pressure on NT-pro-BNP in obstructive sleep apnea patients: a meta-analysis |
| Wu, S. Q., Q. C. Liao, X. X. Xu, L. Sun, J. Wang and R. Chen | 2016 | Effect of CPAP therapy on C-reactive protein and cognitive impairment in patients with obstructive sleep apnea hypopnea syndrome |
| Yang, D., L. Li, J. L. Dong, W. X. Yang and Z. H. Liu | 2023 | Effects of continuous positive airway pressure on cardiac events and metabolic components in patients with moderate to severe obstructive sleep apnea and coronary artery disease: a meta-analysis |
| Zhang, D. M., J. M. Luo, Y. X. Qiao and Y. Xiao | 2016 | Continuous positive airway pressure therapy in non-sleepy patients with obstructive sleep apnea: results of a meta-analysis |
| Zhang, Y., J. G. Weed, R. Ren, X. D. Tang and W. Zhang | 2017 | Prevalence of obstructive sleep apnea in patients with posttraumatic stress disorder and its impact on adherence to continuous positive airway pressure therapy: a meta-analysis |
| 曾丽娟, 郑珍珍, 朱金儒, 曾煜, 刘旺, 陈日垦, 何啟忠 and 成俊芬 | 2021 | 延续护理对持续正压通气治疗阻塞性睡眠呼吸暂停低通气综合征患者依从性及疗效的Meta分析 |
| 陈淳, 丁健 and 谢晋 | 2019 | CPAP治疗对阻塞性睡眠呼吸暂停综合征患者血清炎症标志物影响的Meta分析 |
| 陈华娇, 余维 and 李兵 | 2016 | 持续气道正压通气治疗中重度OSAHS患者疗效的Meta分析 |
| 陈梦, 姚晓光, 洪静 and 努尔古丽.买买提 | 2021 | CPAP对OSAHS患者外周血TNF-α影响的Meta分析 |
| 陈忠云, 谢嬛 and 杨叔禹 | 2017 | 持续气道正压通气治疗对2型糖尿病合并阻塞性睡眠呼吸暂停综合征患者影响的Meta分析 |
| 陈子盛, 傅应云, 刘盛国 and 何正强 | 2012 | 持续气道正压通气治疗阻塞性睡眠呼吸暂停综合征对其胰岛素抵抗影响的meta分析 |
| 程杰龙, 肖源, 詹碧鸣 and 胡建新 | 2018 | 持续气道正压通气治疗阻塞性睡眠呼吸暂停对心房颤动射频消融术疗效影响的Meta分析 |
| 董欢霁, 郭雪君, 徐卫国, 罗勇 and 许小幸 | 2008 | 持续气道正压通气治疗阻塞性睡眠呼吸暂停综合征患者的主观嗜睡程度和心理变化的Meta分析 |
| 范晟, 艾波, 廖永德 and 吴骁伟 | 2018 | 持续性气道正压通气对阻塞性睡眠呼吸暂停患者内皮功能紊乱疗效的随机对照Meta分析 |
| 顾洁 and 陈小东 | 2009 | 阻塞性睡眠呼吸暂停综合征与胰岛素抵抗相关性的荟萃分析 |
| 李慧, 徐健, 钟定 and 李莉 | 2012 | 持续气道正压通气对中国阻塞性睡眠呼吸暂停低通气综合征患者胰岛素抵抗影响的荟萃分析 |
| 李松桃, 方明亮, 郭华, 胡玲, 代平, 余阗, 王莹 and 杨炜 | 2019 | 持续气道正压通气对阻塞性睡眠呼吸暂停综合征患者心血管事件影响的meta分析 |
| 罗英, 宁莉萍, 史菲菲, 何梅, 杨卜凡 and 崔丽君 | 2024 | 阻塞性睡眠呼吸暂停患者持续气道正压通气治疗依从性及影响因素的Meta分析 |
| 王少丽, 史大卓 and 王承龙 | 2012 | 持续气道正压通气或上呼吸道手术治疗对合并阻塞性睡眠呼吸暂停冠心病患者预后影响的系统评价 |
| 王宇鑫, 罗金梅, 黄蓉 and 肖毅 | 2023 | 持续气道正压通气对合并阻塞性睡眠呼吸暂停的心房颤动患者射频消融后复发的影响的荟萃分析 |
| 徐健, 黄平, 宋冰 and 陈济明 | 2013 | 持续气道正压通气对阻塞性睡眠呼吸暂停综合征患者勃起功能障碍疗效的meta分析 |
| 许婷 | 2012 | 自动压力模式与固定压力模式CPAP治疗阻塞性睡眠呼吸暂停疗效的随机对照试验的系统评价 |
| 许婷, 李涛平, 冼乐武, 李丹青 and 王媛媛 | 2011 | 自动压力模式与固定压力模式持续气道正压通气治疗阻塞性睡眠呼吸暂停综合征疗效的系统评价 |
| 叶红, 李涛平, 冯媛, 申海燕 and 刘爱华 | 2009 | 持续气道正压通气治疗对阻塞性睡眠呼吸暂停低通气综合征患者疗效的系统评价 |
| 张慧 | 2022 | 持续正压通气治疗对合并阻塞性睡眠呼吸暂停的射血分数减低型心衰患者左室重构和心功能的影响 |
| 张婷, 江灿, 黄庆玲, 雷莉, 毛丹丹, 王彦婷 and 乐发国 | 2018 | 持续正压通气与口腔矫正器治疗阻塞性睡眠呼吸暂停低通气综合征疗效的Meta分析 |
| 张宇, 陈邓, 朱丽娜, 徐达, 王海娇 and 刘凌 | 2017 | 持续气道正压通气治疗癫与阻塞性睡眠呼吸暂停综合征共病疗效的系统评价 |

**Non-continuous positive airway pressure ventilation**

|  | 2016 | Effect of Strength Training on Sleep Apnea in the Elderly. |
| --- | --- | --- |
|  | 2021 | Investigation of the Effects of Respiratory Muscle Training Combined With Aerobic Exercise in Persons With Obstructive Sleep Apnea Syndrome. |
| Altay, S., S. Firat, Y. Peker and T. Collaborators | 2023 | A Narrative Review of the Association of Obstructive Sleep Apnea with Hypertension: How to Treat Both When They Coexist? |
| Bahgat, A., M. Elwany, C. Vicini, Y. Bahgat, G. Magliulo, A. Greco, A. De Virgilio, A. Pace, M. G. Bellizzi, E. Croce, L. Gatti, A. Maniaci, J. R. Lechien, A. Caranti, S. Gargula, L. A. Vaira, H. Dos Santos and G. Iannella | 2025 | Effect of targeted multilevel sleep surgery on resistant hypertension in patients with severe obstructive sleep apnea |
| Bakker, J. P., B. A. Edwards, S. P. Gautam, S. B. Montesi, J. Durán-Cantolla, F. Aizpuru, F. Barbé, M. Sánchez-de-la-Torre and A. Malhotra | 2014 | Blood pressure improvement with continuous positive airway pressure is independent of obstructive sleep apnea severity |
| Bartolucci, M. L., S. I. Parenti, F. Bortolotti, G. Corazza, L. Solidoro, C. Paganelli and G. Alessandri-Bonetti | 2023 | The Effect of Bite Raise on AHI Values in Adult Patients Affected by OSA: A Systematic Review with Meta-Regression |
| Battisha, A., A. Kahlon and D. K. Kalra | 2025 | Sleep-Disordered Breathing and Hypertension-A Systematic Review |
| Birling, Y., Y. Wu and M. Rahimi | 2025 | Chinese herbal medicine for obstructive sleep apnoea: a systematic review with meta-analysis |
| Bortolotti, F., G. Corazza, M. L. Bartolucci, S. I. Parenti, C. Paganelli and G. Alessandri-Bonetti | 2022 | Dropout and adherence of obstructive sleep apnoea patients to mandibular advancement device therapy: A systematic review of randomised controlled trials with meta-analysis and meta-regression |
| Boyd, S. B., R. Chigurupati, J. E. Cillo, G. Eskes, R. Goodday, T. Meisami, C. F. Viozzi, P. Waite and J. Wilson | 2019 | Maxillomandibular Advancement Improves Multiple Health-Related and Functional Outcomes in Patients With Obstructive Sleep Apnea: A Multicenter Study |
| Brennan, H. L. and S. D. Kirby | 2023 | The role of artificial intelligence in the treatment of obstructive sleep apnea |
| Chen, T. A., S. T. Mao, H. C. Lin, W. T. Liu, K. W. Tam, C. Y. Tsai and Y. C. Kuan | 2023 | Effects of inspiratory muscle training on blood pressure- and sleep-related outcomes in patients with obstructive sleep apnea: a meta-analysis of randomized controlled trials |
| Dameer, A. M., M. L. Jackson, G. Kennedy and S. R. Robinson | 2019 | Literature review on the association between obstructive sleep apnoea and organ damage |
| de Sousa, A. S., A. P. da Rocha, D. R. B. Tavares, J. E. F. Okazaki, M. V. D. Santana, V. F. M. Trevisani and A. Pinto | 2024 | Respiratory muscle training for obstructive sleep apnea: Systematic review and meta-analysis |
| de Vries, G. E., P. J. Wijkstra, E. J. Houwerzijl, H. A. M. Kerstjens and A. Hoekema | 2018 | Cardiovascular effects of oral appliance therapy in obstructive sleep apnea: A systematic review and meta-analysis |
| El-Solh, A. A., E. Gould, K. Aibangbee, T. Jimerson and R. Hartling | 2025 | Current perspectives on the use of GLP-1 receptor agonists in obesity-related obstructive sleep apnea: a narrative review |
| Evans, S., M. Berg, M. Bruschettini and R. Soll | 2022 | Doxapram for the prevention and treatment of apnea in preterm infants |
| Feng, X., Y. Zhang, Y. Shi, R. Ren, F. Lei, M. V. Vitiello and X. Tang | 2025 | Clinical characteristics of obstructive sleep apnoea patients with residual sleepiness |
| Fiedorczuk, P., A. Stróżyński and E. Olszewska | 2020 | Is the oxidative stress in obstructive sleep apnea associated with cardiovascular complications?— systematic review |
| Figard, C., R. Ben Messaoud, M. Joyeux-Faure, M. Destors, R. Tamisier, C. Khouri and J. L. Pépin | 2025 | Effect of sleep apnoea interventions on multiple health outcomes: an umbrella review of meta-analyses of randomised controlled trials |
| Georgoulis, M., N. Yiannakouris, I. Kechribari, K. Lamprou, E. Perraki, E. Vagiakis and M. D. Kontogianni | 2023 | Sustained improvements in the cardiometabolic profile of patients with obstructive sleep apnea after a weight-loss Mediterranean diet/lifestyle intervention: 12-month follow-up (6 months post- intervention) of the "MIMOSA" randomized clinical trial |
| Gjerde, K., S. Lehmann, M. E. Berge, A. K. Johansson and A. Johansson | 2016 | Oral appliance treatment in moderate and severe obstructive sleep apnoea patients non-adherent to CPAP |
| Hajipour, M., B. Baumann, A. Azarbarzin, A. J. H. Allen, Y. Liu, S. Fels, S. Goodfellow, A. Singh, R. Jen and N. T. Ayas | 2023 | Association of alternative polysomnographic features with patient outcomes in obstructive sleep apnea: a systematic review |
| Hilmisson, H. and S. Magnusdottir | 2019 | Beyond the apnea hypopnea index (AHI): importance of sleep quality management of obstructive sleep apnea (OSA) and related mortality in patients with cardiovascular disease |
| Huang, Z. W., Z. H. Liu, Z. H. Zhao, Q. Zhao, Q. Luo and Y. Tang | 2016 | Effects of Continuous Positive Airway Pressure on Lipidaemia and High-sensitivity C-reactive Protein Levels in Non-obese Patients with Coronary Artery Disease and Obstructive Sleep Apnoea |
| Iftikhar, I. H., E. R. Hays, M. A. Iverson, U. J. Magalang and A. K. Maas | 2013 | Effect of oral appliances on blood pressure in obstructive sleep apnea: a systematic review and meta-analysis |
| Jaafar, M., A. Godhamgaonkar, S. Alsanjari and M. Protty | 2025 | The role of cardiac magnetic resonance imaging in obstructive sleep apnea: a systematic scoping review |
| Jennum, P., P. Tonnesen, R. Ibsen and J. Kjellberg | 2017 | Obstructive sleep apnea: effect of comorbidities and positive airway pressure on all-cause mortality |
| Jonas, D. E., H. R. Amick, C. Feltner, R. P. Weber, M. Arvanitis, A. Stine, L. Lux and R. P. Harris | 2017 | Screening for Obstructive Sleep Apnea in Adults Evidence Report and Systematic Review for the US Preventive Services Task Force |
| Ken-Dror, G., C. H. Fry, P. Murray, D. Fluck and T. S. Han | 2021 | Changes in cortisol levels by continuous positive airway pressure in patients with obstructive sleep apnoea: Meta-analysis of 637 individuals |
| Kuna, S. T., R. R. Townsend, B. T. Keenan, D. Maislin, T. Gislason, B. Benediktsdóttir, S. Gudmundsdóttir, E. S. Arnardóttir, A. Sifferman, B. Staley, F. M. Pack, X. F. Guo, R. J. Schwab, G. Maislin, J. A. Chirinos and A. I. Pack | 2019 | Blood pressure response to treatment of obese vs non-obese adults with sleep apnea |
| Le, K. D. R., K. Le and F. Foo | 2024 | The Impact of Glucagon-like Peptide 1 Receptor Agonists on Obstructive Sleep Apnoea: A Scoping Review |
| Li, K. K. | 2011 | Maxillomandibular Advancement for Obstructive Sleep Apnea |
| Li, M. X., H. Lin, Q. R. Yang, X. L. Zhang, Q. Zhou, J. K. Shi and F. F. Ge | 2025 | Glucagon-like peptide-1 receptor agonists for the treatment of obstructive sleep apnea: a meta-analysis |
| Li, X. M., Z. L. Dong, Y. X. Wan and Z. P. Wang | 2010 | Sildenafil versus continuous positive airway pressure for erectile dysfunction in men with obstructive sleep apnea: a meta-analysis |
| Lv, Z. T., W. X. Jiang, J. M. Huang, J. M. Zhang and A. M. Chen | 2016 | The Clinical Effect of Acupuncture in the Treatment of Obstructive Sleep Apnea: A Systematic Review and Meta-Analysis of Randomized Controlled Trials |
| Maier, L. E., B. A. Matenchuk, A. Vucenovic, A. Sivak, M. H. Davenport and C. D. Steinback | 2022 | Influence of Obstructive Sleep Apnea Severity on Muscle Sympathetic Nerve Activity and Blood Pressure: a Systematic Review and Meta-Analysis |
| Mason, M., E. J. Welsh and I. Smith | 2013 | Drug therapy for obstructive sleep apnoea in adults |
| McEvoy, R. D. and M. Kohler | 2018 | Con: continuous positive airway pressure and cardiovascular prevention |
| Middleton, S., W. Vermeulen, K. Byth, C. E. Sullivan and P. G. Middleton | 2009 | Treatment of obstructive sleep apnoea in Samoa progressively reduces daytime blood pressure over 6 months |
| Murtaza, S., R. Sohail, B. Akram, S. Tahira, M. Zaman and S. N. Mehmood | 2025 | Evaluating the Efficacy of Sodium Glucose Cotransporter-2 Inhibitors in Management of Obstructive Sleep Apnea; a Systematic Review and Meta-Analysis |
| Nagappa, M., H. Abdullah, N. Siddiqui and F. Chung | 2016 | DIAGNOSIS AND TREATMENT OF OBSTRUCTIVE SLEEP APNEA DURING PREGNANCY, A SYSTEMATIC REVIEW |
| Ni, Y. N., H. Yang and R. J. Thomas | 2021 | The role of acetazolamide in sleep apnea at sea level: A systematic review and meta-analysis |
| Pagel, J. F. | 2007 | Obstructive sleep apnea (OSA) in primary care: evidence-based practice |
| Pengo, M. F., D. Soranna, A. Giontella, E. Perger, P. Mattaliano, E. I. Schwarz, C. Lombardi, G. Bilo, A. Zambon, J. Steier, G. Parati, P. Minuz and C. Fava | 2020 | Obstructive sleep apnoea treatment and blood pressure: which phenotypes predict a response? A systematic review and meta-analysis |
| Shamsuzzaman, A. S., B. J. Gersh and V. K. Somers | 2003 | Obstructive sleep apnea: implications for cardiac and vascular disease |
| Shamsuzzaman, A. S. M., B. J. Gersh and V. K. Somers | 2003 | Obstructive sleep apnea - Implications for cardiac and vascular disease |
| Shantha, G. P. and S. B. Pancholy | 2015 | Effect of renal sympathetic denervation on apnea-hypopnea index in patients with obstructive sleep apnea: a systematic review and meta-analysis |
| Sharples, L. D., A. L. Clutterbuck-James, M. J. Glover, M. S. Bennett, R. Chadwick, M. A. Pittman and T. G. Quinnell | 2016 | Meta-analysis of randomised controlled trials of oral mandibular advancement devices and continuous positive airway pressure for obstructive sleep apnoea-hypopnoea |
| Stanek, A., K. Brozyna-Tkaczyk and W. Myslinski | 2021 | Oxidative Stress Markers among Obstructive Sleep Apnea Patients |
| Tang, W. Z., K. J. Huang, H. Y. Xu, Q. Y. Cai, Y. P. Song, T. Q. Fan, Y. Zhang, T. H. Liu and Y. B. Li | 2025 | Obstructive sleep apnea-associated hypertensive disorders in pregnancy: a literature review and clinical management strategies |
| Thomasouli, M. A., E. M. Brady, M. J. Davies, A. P. Hall, K. Khunti, D. H. Morris and L. J. Gray | 2013 | The impact of diet and lifestyle management strategies for obstructive sleep apnoea in adults: a systematic review and meta-analysis of randomised controlled trials |
| Verbraecken, J., M. Dieltjens, S. O. de Beeck, A. Vroegop, M. Braem, O. Vanderveken and W. Randerath | 2022 | Non-CPAP therapy for obstructive sleep apnoea |
| West, S. D. and C. Turnbull | 2016 | Eye disorders associated with obstructive sleep apnoea |
| Wolf, J., J. Lewicka and K. Narkiewicz | 2007 | Obstructive sleep apnea: an update on mechanisms and cardiovascular consequences |
| Zhang, J., Z. Chen, Y. Deng, X. Teng, X. Ling, C. He, H. Zhang and D. Lu | 2024 | Impacts of renal denervation on blood pressure in patients with obstructive sleep apnea |
| 王曼 | 2016 | 多种干预措施治疗阻塞性睡眠呼吸暂停低通气综合征疗效及安全性的meta分析 |
| 魏才杰, 夏晓黎, 张宁, 马艳萍, 王亚锋, 马娇娇 and 张帆 | 2022 | 双水平气道正压通气治疗慢性阻塞性肺疾病合并阻塞性睡眠呼吸暂停低通气综合征有效性的Meta分析 |
| 赵力博, 赵哲, 刘敏, 何子君, 房凤凤, 蔡伟梦, 范利 and 刘霖 | 2023 | 老年男性阻塞性睡眠呼吸暂停综合征相关高血压的影响因素分析及风险预测积分评定系统构建 |

**Non-obstructive sleep apnea**

|  | 2025 | Remission of Type 2 Diabetes With Mediterranean Diet, Physical Activity and Psychological Support: a Randomized Clinical Trial. |
| --- | --- | --- |
| Atkins, T., D. Marks, C. Dowsett, P. Glasziou and L. Albarqouni | 2025 | Trial-based economic evaluations of non-drug interventions in the Royal Australian College of General Practitioners (RACGP) Handbook of Non-Drug Interventions in primary care: a systemic review |
| Bacan, G., A. Ribeiro-Silva, V. A. S. Oliveira, C. R. L. Cardoso and G. F. Salles | 2022 | Refractory Hypertension: a Narrative Systematic Review with Emphasis on Prognosis |
| Basamh, M. | 2021 | Primary de-differentiated liposarcoma of the colon with a concurrent kidney nodule: A case report & review of literature |
| Benjamin, E. J., S. M. Al-Khatib, P. Desvigne-Nickens, A. Alonso, L. Djoussé, D. E. Forman, A. M. Gillis, J. M. L. Hendriks, M. T. Hills, P. Kirchhof, M. S. Link, G. M. Marcus, R. Mehra, K. T. Murray, R. Parkash, I. L. Piña, S. Redline, M. Rienstra, P. Sanders, V. K. Somers, D. R. Van Wagoner, P. J. Wang, L. S. Cooper and A. S. Go | 2021 | Research priorities in the secondary prevention of atrial fibrillation: A national heart, lung, and blood institute virtual workshop report |
| Bhardwaj, N. and M. Siuba | 2020 | Obesity, COVID-19, and modified interventions in the ICU: Emerging lessons from a case study |
| Burton, J. K., L. E. Craig, S. Q. Yong, N. Siddiqi, E. A. Teale, R. Woodhouse, A. J. Barugh, A. M. Shepherd, A. Brunton, S. C. Freeman, A. J. Sutton and T. J. Quinn | 2021 | Non-pharmacological interventions for preventing delirium in hospitalised non-ICU patients |
| Charitakis, E., E. Dragioti, M. Stratinaki, D. Korela, S. Tzeis, H. Almroth, I. Liuba, A. H. Jönsson, G. Charalambous, L. O. Karlsson and D. Tsartsalis | 2023 | Predictors of recurrence after catheter ablation and electrical cardioversion of atrial fibrillation: an umbrella review of meta-analyses |
| Dharia, S., Z. A. R. A. Arain and I. M. Donskoy | 2023 | PROLONGED CENTRAL APNEA WITH AN RSV INFECTION |
| Edell, M. H. and R. Grad | 2018 | Top 20 Research Studies of 2017 for Primary Care Physicians |
| Ghasemi, H., S. Kazemian, S. A. Nejadghaderi and M. Shafie | 2023 | Takotsubo syndrome and COVID-19: A systematic review |
| Holthof, K., P. O. Bridevaux and I. Frésard | 2022 | Underlying lung disease and exposure to terrestrial moderate and high altitude: personalised risk assessment |
| Hong, K. L. and B. M. Glover | 2018 | The impact of lifestyle intervention on atrial fibrillation |
| Lee, H. M., H. Y. Kim, J. D. Suh, K. D. Han, J. K. Kim, Y. C. Lim, S. C. Hong and J. H. Cho | 2018 | Uvulopalatopharyngoplasty reduces the incidence of cardiovascular complications caused by obstructive sleep apnea: results from the national insurance service survey 2007-2014 |
| Nct | 2016 | nHFOV vs nCPAP: effects on Gas Exchange for the Treatment of Neonates Recovering From RDS |
| Ponce de Leon-Ballesteros, G., G. Romero-Velez, K. Higa, J. Himpens, M. O’ Kane, A. Torres, G. Prager and M. F. Herrera | 2024 | Single Anastomosis Duodeno-Ileostomy with Sleeve Gastrectomy/Single Anastomosis Duodenal Switch (SADI-S/SADS) IFSO Position Statement—Update 2023 |
| Sachdeva, A., R. N Ogbonna and T. Chatterjee | 2022 | PULMONARY EMBOLISM AND HEART FAILURE FROM EXOGENOUS TESTOSTERONE USE |
| Sánchez-De-La-Torre, M., E. Gracia-Lavedan, I. D. Benitez, A. Sánchez-De-La-Torre, A. Moncusí-Moix, G. Torres, K. Loffler, R. Woodman, R. Adams, G. Labarca, J. Dreyse, C. Eulenburg, E. Thunström, H. Glantz, Y. Peker, C. Anderson, D. McEvoy and F. Barbé | 2023 | Adherence to CPAP Treatment and the Risk of Recurrent Cardiovascular Events: A Meta-Analysis |
| Smith, N. A., G. Martin and B. Marginson | 2022 | Preoperative assessment and prehabilitation in patients with obesity undergoing non-bariatric surgery: A systematic review |
| Tota, V., M. Dagonnier, D. Wery, L. Binet, N. Nagy, V. Durieux, M. Diaz, J. A. Elosegi and S. Holbrechts | 2021 | Antiphospholipid syndrome-induced ischemic stroke following pembrolizumab: Case report and systematic review |
| Umeda, A., K. Miyagawa, A. Mochida, H. Takeda, K. Takeda, Y. Okada and D. Gozal | 2020 | Effects of Normoxic Recovery on Intima-Media Thickness of Aorta and Pulmonary Artery Following Intermittent Hypoxia in Mice |
| Wells, D., Y. Moustafa and G. R. Orozco | 2022 | Insomnia treatment in an elderly patient with Schizophrenia. Illustrative case, challenges and literature review |
| Yuen, K. C. J., L. S. Blevins and J. W. Findling | 2020 | Important Management Considerations In Patients With Pituitary Disorders During The Time Of The Covid-19 Pandemic |
| 马林沁 | 2017 | 慢性间歇性低氧复合胰岛素抵抗介导的动脉粥样硬化过程中SREBP-1c/FAS信号通路的作用及益气活血中药的干预效应研究 |
| 谭赟 | 2022 | OSA风险基因位点及炎症因子研究 |

**clinical research**

|  | 2008 | Effects of CPAP on Cardiovascular Risk Profile in Patients With Severe Obstructive Sleep Apnea and Metabolic Syndrome. |
| --- | --- | --- |
|  | 2010 | Usefulness of a Telemedicine System for OSA Patients Follow-up With High Cardiovascular Risk. |
| Akinmoju, O. D., G. Olatunji, E. Kokori, I. J. Ogieuhi, A. E. Babalola, E. S. Obi, C. S. Anthony, O. G. Toluwanibukun, A. Akingbola, A. E. Alao and et al. | 2025 | Comparative Efficacy of Continuous Positive Airway Pressure and Antihypertensive Medications in Obstructive Sleep Apnea-Related Hypertension: a Narrative Review |
| Barbé, F., J. Durán-Cantolla, F. Capote, M. de la Peña, E. Chiner, J. F. Masa, M. Gonzalez, J. M. Marín, F. Garcia-Rio, J. D. de Atauri, J. Terán, M. Mayos, C. Monasterio, F. del Campo, S. Gomez, M. Sanchez de la Torre, M. Martinez, J. M. Montserrat, S. Spanish and G. Breathing | 2010 | Long-term Effect of Continuous Positive Airway Pressure in Hypertensive Patients with Sleep Apnea |
| Barbé, F., J. Durán-Cantolla, M. Sánchez-de-la-Torre, M. Martinez-Alonso, C. Carmona, A. Barceló, E. Chiner, J. F. Masa, M. Gonzalez, J. M. Marín, F. Garcia-Rio, J. D. de Atauri, J. Terán, M. Mayos, M. de la Peña, C. Monasterio, F. del Campo, J. M. Montserrat and N. Spanish Sleep Breathing | 2012 | Effect of Continuous Positive Airway Pressure on the Incidence of Hypertension and Cardiovascular Events in Nonsleepy Patients With Obstructive Sleep Apnea A Randomized Controlled Trial |
| Barnas, M., M. Maskey-Warzechowska, P. Bielicki, M. Kumor and R. Chazan | 2017 | Diurnal and nocturnal serum melatonin concentrations after treatment with continuous positive airway pressure in patients with obstructive sleep apnea |
| Campos-Rodriguez, F., M. Gonzalez-Martinez, A. Sanchez-Armengol, B. Jurado-Gamez, J. Cordero-Guevara, N. Reyes-Nuñez, M. F. Troncoso, A. Abad-Fernandez, J. Teran-Santos, J. Caballero-Rodriguez, M. Martin-Romero, A. Encabo-Motiño, L. Sacristan-Bou, J. Navarro-Esteva, M. Somoza-Gonzalez, J. F. Masa, M. A. Sanchez-Quiroga, B. Jara-Chinarro, B. Orosa-Bertol, M. A. Martinez-Garcia and N. Spanish Sleep | 2017 | Effect of continuous positive airway pressure on blood pressure and metabolic profile in women with sleep apnoea |
| Cardoso, C. R. L., C. N. Roderjan, A. H. Cavalcanti, A. F. Cortez, E. S. Muxfeldt and G. F. Salles | 2020 | Effects of continuous positive airway pressure treatment on aortic stiffness in patients with resistant hypertension and obstructive sleep apnea: A randomized controlled trial |
| Cardoso, C. R. L. and G. F. Salles | 2023 | Prognostic importance of obstructive sleep apnea and CPAP treatment for cardiovascular and mortality outcomes in patients with resistant hypertension: a prospective cohort study |
| Chen, Q., Y. B. Cheng, M. Shen, B. Yin, H. H. Yi, J. Feng, M. Li, Q. Y. Li, Y. Li and J. G. Wang | 2020 | A randomized controlled trial on ambulatory blood pressure lowering effect of CPAP in patients with obstructive sleep apnea and nocturnal hypertension |
| Crinion, S. J., J. Kleinerova, B. Kent, G. Nolan, C. T. Taylor, S. Ryan and W. T. McNicholas | 2021 | Non-dipping nocturnal blood pressure correlates with obstructive sleep apnoea severity in normotensive subjects and may reverse with therapy |
| Ducuara-Izquierdo, L. Y., J. D. Saraza-Perea, L. N. Castillo-Hoyos, M. F. Serna-Orozco and H. A. Payan-Salcedo | 2025 | Effects of daily-use positive pressure devices on physical performance in patients with obstructive sleep apnea: an exploratory systematic review |
| Durán-Cantolla, J., F. Aizpuru, J. M. Montserrat, E. Ballester, J. Terán-Santos, J. I. Aguirregomoscorta, M. Gonzalez, P. Lloberes, J. F. Masa, M. De la Peña, S. Carrizo, M. Mayos, F. Barbé and G. Spanish Sleep Breathing | 2010 | Continuous positive airway pressure as treatment for systemic hypertension in people with obstructive sleep apnoea: randomised controlled trial |
| Fichera, G., V. Ronsivalle, G. Zappalà, P. Campagna, V. Quinzi and A. L. Giudice | 2021 | Mandibular advancement devices (Mad) as a treatment alternative for obstructive sleep apnea syndrome (osas) |
| Green, M., G. Ken-Dror, D. Fluck, C. Sada, P. Sharma, C. H. Fry and T. S. Han | 2021 | Meta-analysis of changes in the levels of catecholamines and blood pressure with continuous positive airway pressure therapy in obstructive sleep apnea |
| Han, S. H. and S. A. Lee | 2020 | Acute effects of continuous positive airway pressure in patients with obstructive sleep apnea |
| Jain, S., I. Gurubhagavatula, R. Townsend, S. T. Kuna, K. Teff, T. A. Wadden, J. Chittams, A. L. Hanlon, G. Maislin, H. Saif, P. Broderick, Z. Ahmad, A. I. Pack and J. A. Chirinos | 2017 | Effect of CPAP, Weight Loss, or CPAP Plus Weight Loss on Central Hemodynamics and Arterial Stiffness |
| Jain, S., P. Yingchoncharoen and K. Nugent | 2023 | Effect of Continuous Positive Airway Pressure Therapy in Patient with Obstructive Sleep Apnea and Pulmonary Hypertension: A literature review |
| Ken-Dror, G., M. Wood, D. Fluck, P. Sharma, C. H. Fry and T. S. Han | 2021 | Continuous positive airway pressure therapy reduces the levels of catecholamines and blood pressure in pseudophaeochromocytoma with coexisting obstructive sleep apnoea |
| Lee, Y. C., Y. C. Chang, L. W. Tseng, W. N. Lin, C. T. Lu, L. A. Lee, T. J. Fang, W. N. Cheng and H. Y. Li | 2024 | Continuous Positive Airway Pressure Treatment and Hypertensive Adverse Outcomes in Pregnancy A : Systematic Review and Meta-Analysis |
| Lettau, F., E. I. Schwarz, J. R. Stradling and M. Kohler | 2017 | Blood Pressure Variability in Obstructive Sleep Apnoea: Data from 4 Randomised Controlled CPAP Withdrawal Trials |
| Lin, X., G. Chen, J. Qi, X. Chen, J. Zhao and Q. Lin | 2016 | Effect of continuous positive airway pressure on arterial stiffness in patients with obstructive sleep apnea and hypertension: a meta-analysis |
| Liu, J., J. Xu, S. Guan and W. Wang | 2024 | Effects of different treatments on metabolic syndrome in patients with obstructive sleep apnea: a meta-analysis |
| Lozano, L., J. L. Tovar, G. Sampol, O. Romero, M. J. Jurado, A. Segarra, E. Espinel, J. Ríos, M. D. Untoria and P. Lloberes | 2010 | Continuous positive airway pressure treatment in sleep apnea patients with resistant hypertension: a randomized, controlled trial |
| Lui, M. M. S., H. F. Tse, D. C. L. Lam, K. K. Lau, C. W. S. Chan and M. S. M. Ip | 2021 | Continuous positive airway pressure improves blood pressure and serum cardiovascular biomarkers in obstructive sleep apnoea and hypertension |
| Martínez-García, M. A., F. Capote, F. Campos-Rodríguez, P. Lloberes, M. J. D. de Atauri, M. Somoza, J. F. Masa, M. González, L. Sacristàn, F. Barbé, J. Durán-Cantolla, F. Aizpuru, E. Mañas, B. Barreiro, M. Mosteiro, J. J. Cebrián, M. de la Peña, F. García-Río, A. Maimó, J. Zapater, C. Hernández, N. G. SanMarti and J. M. Montserrat | 2013 | Effect of CPAP on Blood Pressure in Patients With Obstructive Sleep Apnea and Resistant Hypertension The HIPARCO Randomized Clinical Trial |
| Navarro-Soriano, C., M. A. Martínez-García, G. Torres, F. Barbé, C. Caballero-Eraso, P. Lloberes, T. D. Cambriles, M. Somoza, J. F. Masa, M. González, E. Mañas, M. de la Peña, F. García-Río, J. M. Montserrat, A. Muriel, G. Oscullo, L. F. Olmos, A. García-Ortega, D. Calhoun, F. Campos-Rodriguez and N. Spanish Sleep | 2019 | Effect of continuous positive airway pressure in patients with true refractory hypertension and sleep apnea: a post-hoc intention-to-treat analysis of the HIPARCO randomized clinical trial |
| Navarro-Soriano, C., M. A. Martínez-García, G. Torres, F. Barbé, M. Sánchez-de-la-Torre, C. Caballero-Eraso, P. Lloberes, T. D. Cambriles, M. Somoza, J. F. Masa, M. González, E. Mañas, M. de la Peña, F. García-Río, J. M. Montserrat, A. Muriel, G. Oscullo, A. García-Ortega, T. Posadas, F. Campos-Rodríguez and N. Spanish Sleep | 2021 | Long -term Effect of CPAP Treatment on Cardiovascular Events in Patients With Resistant Hypertension and Sleep Apnea. Data From the HIPARCO-2 Study |
| Pak, V. M., D. G. Maislin, B. T. Keenan, R. R. Townsend, B. Benediktsdottir, S. B. Dunbar, A. I. Pack, T. Gislason and S. T. Kuna | 2021 | Changes in sleepiness and 24-h blood pressure following 4 months of CPAP treatment are not mediated by ICAM-1 |
| Pépin, J. L., R. Tamisier, G. Barone-Rochette, S. H. Launois, P. Lévy and J. P. Baguet | 2010 | Comparison of Continuous Positive Airway Pressure and Valsartan in Hypertensive Patients with Sleep Apnea |
| Pleava, R., S. Mihaicuta, C. L. Serban, C. Ardelean, I. Marincu, D. Gaita and S. Frent | 2020 | Long-Term Effects of Continuous Positive Airway Pressure (CPAP) Therapy on Obesity and Cardiovascular Comorbidities in Patients with Obstructive Sleep Apnea and Resistant Hypertension-An Observational Study |
| Sánchez-de-la-Torre, M., E. Gracia-Lavedan, I. D. Benítez, A. Zapater, G. Torres, A. Sánchez-de-la-Torre, A. Aldoma, J. de Batlle, A. Targa, J. Abad, J. Duran-Cantolla, A. Urrutia, O. Mediano, M. J. Masdeu, E. Ordax-Carbajo, J. F. Masa, M. De la Peña, M. Mayos, R. Coloma, J. M. Montserrat, E. Chiner, O. Mínguez, L. Pascual, A. Cortijo, D. Martínez, M. Dalmases, C. H. Lee, R. D. McEvoy, F. Barbé and N. Spanish Sleep | 2022 | Long-Term Effect of Obstructive Sleep Apnea and Continuous Positive Airway Pressure Treatment on Blood Pressure in Patients with Acute Coronary Syndrome A Clinical Trial |
| Sánchez-de-la-Torre, M., A. Khalyfa, A. Sánchez-de-la-Torre, M. Martinez-Alonso, M. A. Martinez-García, A. Barceló, P. Lloberes, F. Campos-Rodriguez, F. Capote, M. J. Diaz-de-Atauri, M. Somoza, M. González, J. F. Masa, D. Gozal, F. Barbé and N. Spanish Sleep | 2015 | Precision Medicine in Patients With Resistant Hypertension and Obstructive Sleep Apnea Blood Pressure Response to Continuous Positive Airway Pressure Treatment |
| Sánchez-de-la-Torre, M., A. Sánchez-de-la-Torre, S. Bertran, J. Abad, J. Duran-Cantolla, V. Cabriada, O. Mediano, M. J. Masdeu, M. L. Alonso, J. F. Masa, A. Barceló, M. de la Peña, M. Mayos, R. Coloma, J. M. Montserrat, E. Chiner, S. Perelló, G. Rubinós, O. Mínguez, L. Pascual, A. Cortijo, D. Martínez, A. Aldomá, M. Dalmases, R. D. McEvoy, F. Barbé and N. Spanish Steep | 2020 | Effect of obstructive sleep apnoea and its treatment with continuous positive airway pressure on the prevalence of cardiovascular events in patients with acute coronary syndrome (ISAACC study): a randomised controlled trial |
| Sapiña-Beltrán, E., G. Torres, I. Benítez, F. Santamaría-Martos, J. Durán-Cantolla, C. Egea, M. Sánchez-de-la-Torre, F. Barbé, M. Dalmases, F. Aizpuru, J. M. Montserrat, E. Ballester, J. I. Aguirregomoscorta, M. Gonzalez, P. Lloberes, J. F. Masa, M. de la Peña, S. Carrizo, M. Mayos and G. Spanish Sleep Breathing | 2019 | Differential blood pressure response to continuous positive airway pressure treatment according to the circadian pattern in hypertensive patients with obstructive sleep apnoea |
| Seidel, M., M. F. Kiziler, M. Mathiakis, S. Bertram, S. Wang, F. S. Seibert, N. Babel and T. H. Westhoff | 2024 | PREDICTORS OF BLOOD PRESSURE RESPONSE TO CPAP TREATMENT IN PATIENTS WITH SLEEP APNEA |
| Shirahama, R., T. Tanigawa, Y. Ida, K. Fukuhisa, R. Tanaka, K. Tomooka, F. Y. Lan, A. Ikeda, H. Wada and S. N. Kales | 2021 | Long-term effect of continuous positive airway pressure therapy on blood pressure in patients with obstructive sleep apnea |
| Targa, A. D. S., G. Torres, I. D. Benítez, M. Henríquez-Beltrán, R. Vaca, L. P. Arnó, O. Mínguez, M. Aguilà, D. Martínez, L. Pinilla, A. G. Gonzalez, S. B. Garriga, M. Sanchez-de-la-Torre and F. Barbé | 2025 | Effect of continuous positive airway pressure on blood pressure in normotensive individuals with obstructive sleep apnoea: a randomised trial |
| Torres, G., M. Sánchez-de-la-Torre, E. Gracia-Lavedan, I. D. Benitez, D. Martinez, M. Dalmases, L. Pinilla, O. Minguez, R. Vaca, L. Pascual, M. Aguilá, A. Cortijo, C. Gort, M. A. Martinez-Garcia, O. Mediano, S. R. Peralta, A. M. Fortuna-Gutierrez, P. P. Marquez, L. F. Drager, M. Cabrini, S. de Barros, J. F. Masa, J. C. Peñafiel, M. Felez, S. Vázquez, J. Abad, F. García-Rio, R. Casitas, C. H. Lee and F. Barbé | 2024 | Long-term effect of obstructive sleep apnoea management on blood pressure in patients with resistant hypertension: the SARAH study |
| Treptow, E., J. L. Pepin, S. Bailly, P. Levy, C. Bosc, M. Destors, H. Woehrle and R. Tamisier | 2019 | Reduction in sympathetic tone in patients with obstructive sleep apnoea: is fixed CPAP more effective than APAP? A randomised, parallel trial protocol |
| Varounis, C., V. Katsi, I. E. Kallikazaros, D. Tousoulis, C. Stefanadis, J. Parissis, J. Lekakis, C. Siristatidis, A. J. Manolis and T. Makris | 2014 | Effect of CPAP on blood pressure in patients with obstructive sleep apnea and resistant hypertension: a systematic review and meta-analysis |
| Yorgun, H., G. Kabakçi, U. Canpolat, E. Kirmizigül, L. Sahiner, A. H. Ates, M. A. Sendur, E. B. Kaya, A. U. Demir, K. Aytemir, L. Tokgözoglu and A. Oto | 2014 | Predictors of Blood Pressure Reduction With Nocturnal Continuous Positive Airway Pressure Therapy in Patients With Obstructive Sleep Apnea and Prehypertension |
| Zaharie, A. M., S. M. Aştefanei, A. E. Cristea and O. C. Deleanu | 2017 | ANOTHER APPROACH OF RESISTANT HYPERTENSION |
| Zhao, Q., Z. Liu, D. McEvoy, Q. Luo, Z. Zhao, H. Zhang and Y. Wang | 2010 | Effectiveness of continuous positive airway pressure on blood pressure in patients with obstructive sleep apnoea |
| Zhao, Y. Y., R. Wang, K. J. Gleason, E. F. Lewis, S. F. Quan, C. M. Toth, Y. Song, M. Morrical, M. Rueschman, M. A. Mittleman and S. Redline | 2022 | Effect of continuous positive airway pressure treatment on ambulatory blood pressures in high-risk sleep apnea patients: a randomized controlled trial |
| Zou, B., X. Y. Guo, Y. Liu, R. Zou, G. M. Li, J. H. Liu, J. F. Song and Z. N. Xiong | 2018 | Randomized controlled trial of continuous positive airway pressure treatment of resistant hypertensive patients combined with obstructive sleep apnea/hypopnea syndrome |
| 徐健, 黄平, 李志莹 and 陈济明 | 2014 | 持续气道正压通气对阻塞性睡眠呼吸暂停低通气综合征患者外周血IL-6影响的Meta分析 |

**Homogenization of content**

| 胡鑫渝 | 2015 | 持续正压通气治疗阻塞性睡眠呼吸暂停综合征合并高血压的降压疗效 |
| --- | --- | --- |

**To be included**

| Alajmi, M., A. T. Mulgrew, J. Fox, W. Davidson, M. Schulzer, E. Mak, C. F. Ryan, J. Fleetham, P. Choi and N. T. Ayas | 2007I | mpact of continuous positive airway pressure therapy on blood pressure in patients with obstructive sleep apnea hypopnea: a meta-analysis of randomized controlled trials |
| --- | --- | --- |
| Bazzano, L. A., Z. Khan, K. Reynolds and J. He | 2007 | Effect of nocturnal nasal continuous positive airway pressure on blood pressure in obstructive sleep apnea |
| Benning, L., J. J. Herzig, M. S. Mollet, M. Bradicich, M. F. Pengo, S. Ulrich and E. I. Schwarz | 2025 | Effects of CPAP on Blood Pressure Parameter Across Different Severities of Obstructive Sleep Apnoea: A Meta-Analysis |
| Haentjens, P., A. Van Meerhaeghe, A. Moscariello, S. De Weerdt, K. Poppe, A. Dupont and B. Velkeniers | 2007 | The impact of continuous positive airway pressure on blood pressure in patients with obstructive sleep apnea syndrome: evidence from a meta-analysis of placebo-controlled randomized trials |
| Hu, X., J. Fan, S. Chen, Y. Yin and B. Zrenner | 2015 | The role of continuous positive airway pressure in blood pressure control for patients with obstructive sleep apnea and hypertension: a meta-analysis of randomized controlled trialsThe role of continuous positive airway pressure in blood pressure control for patients with obstructive sleep apnea and hypertension: a meta-analysis of randomized controlled trials |
| Labarca, G., A. Schmidt, J. Dreyse, J. Jorquera, D. Enos, G. Torres and F. Barbe | 2021 | Efficacy of continuous positive airway pressure (CPAP) in patients with obstructive sleep apnea (OSA) and resistant hypertension (RH): Systematic review and meta-analysis |
| Liu, L. P., Q. A. Cao, Z. Z. Guo and Q. Y. Dai | 2016 | Continuous Positive Airway Pressure in Patients With Obstructive Sleep Apnea and Resistant Hypertension: A Meta-Analysis of Randomized Controlled Trials |
| Montesi, S. B., B. A. Edwards, A. Malhotra and J. P. Bakker | 2012 | The effect of continuous positive airway pressure treatment on blood pressure: a systematic review and meta-analysis of randomized controlled trials |
| Schein, A. S., A. C. Kerkhoff, C. C. Coronel, R. D. Plentz and G. Sbruzzi | 2014 | Continuous positive airway pressure reduces blood pressure in patients with obstructive sleep apnea; a systematic review and meta-analysis with 1000 patients |
| Shang, W., Y. Zhang, L. Liu, F. Chen, G. Wang and D. Han | 2022 | Benefits of continuous positive airway pressure on blood pressure in patients with hypertension and obstructive sleep apnea: a meta-analysis |
| Sun, Y., Z. Y. Huang, Q. R. Sun, L. P. Qiu, T. T. Zhou and G. H. Zhou | 2016 | CPAP therapy reduces blood pressure for patients with obstructive sleep apnoea: an update meta-analysis of randomized clinical trials |
| 符翠萍, 朱芬, 刘子龙, 励雯静, 吴晓丹, 吴旭, 张舒琪, 白春学, 周新 and 李善群 | 2014 | 持续气道正压通气对阻塞性睡眠呼吸暂停低通气综合征患者血压影响的荟萃分析 |
| 李君, 李晓艳, 蒋学俊, 万为国 and 刘万里 | 2015 | 持续气道正压通气对阻塞性睡眠呼吸暂停综合征患者降压疗效的Meta分析 |
| 莫莉 and 何权瀛 | 2007 | 长期持续气道正压通气对阻塞性睡眠呼吸暂停低通气综合征患者血压影响的荟萃分析 |
| 王馨平 | 2008 | 持续正压气道通气对阻塞性睡眠呼吸暂停低通气综合征中重度患者24小时动态血压影响的有效性评价 |
| 尹富禹 | 2013 | 持续气道正压通气治疗对阻塞性睡眠呼吸暂停综合症患者血压影响的Meta分析 |

**Unable to obtain the full text**

| Benning, L., J. J. Herzig, M. Mollet, M. Bradicich, M. Kohler, S. Ulrich and E. I. Schwarz | 2024 | CPAP effects on blood pressure in different OSA phenotypes - a meta-analysis |
| --- | --- | --- |
| Pengo, M., E. I. Schwarz, F. Barbe, L. Drager, C. Fava, M. S. M. Ip, M. A. Martinez Garcia, D. McEvoy, Y. Peker, C. L. Phillips, D. Soranna, J. Steier, J. Stradling, A. Zambon and G. Parati | 2022 | Effect of CPAP therapy on blood pressure in patients with OSA: a worldwide individual patient data meta-analysis |
| 缪碧芳 | 2016 | 持续气道正压通气治疗对阻塞性睡眠呼吸暂停低通气综合征合并高血压患者疗效的系统评价 |
| 王萍悦, 张琴, 张然, 孙晴晴 and 王赞 | 2015 | 睡眠呼吸障碍与心血管疾病. 第六届中国睡眠医学论坛论文集 |

****Redundant Paper****

|  | 2022 | Effects of Continuous Positive Airway Pressure Therapy Withdrawal in Patients With Obstructive Sleep Apnea: A Randomized Trial. |
| --- | --- | --- |
| Alajmi, M., A. T. Mulgrew, J. Fox, W. Davidson, M. Schulzer, E. Mak, C. F. Ryan, J. Fleetham, P. Choi and N. T. Ayas | 2007 | Impact of continuous positive airway pressure therapy on blood pressure in patients with obstructive sleep apnea hypopnea: A meta-analysis of randomized controlled trials |
| Ando, S. | 2018 | Influence of hypoxia induced by sleep disordered breathing in case of hypertension and atrial fibrillation |
| Bakker, J. P., B. A. Edwards, S. P. Gautam, S. B. Montesi, J. Durán-Cantolla, F. Aizpuru, F. Barbé, M. Sánchez-de-la-Torre and A. Malhotra | 2014 | Blood Pressure Improvement with Continuous Positive Airway Pressure is Independent of Obstructive Sleep Apnea Severity |
| Basamh, M. | 2021 | Primary de-differentiated liposarcoma of the colon with a concurrent kidney nodule: A case report & review of literature |
| Battisha, A., A. Kahlon and D. K. Kalra | 2025 | Sleep-Disordered Breathing and Hypertension-A Systematic Review |
| Bazzano, L. A., Z. Khan, K. Reynolds and J. He | 2007 | Effect of nocturnal nasal continuous positive airway pressure on blood pressure in obstructive sleep apnea |
| Borel, A. L., R. Tamisier, P. Böhme, P. Priou, A. Avignon, P. Y. Benhamou, H. Hanaire, J. L. Pépin, L. Kessler, P. Valensi, P. Darmon and F. Gagnadoux | 2019 | Obstructive sleep apnoea syndrome in patients living with diabetes: Which patients should be screened? |
| Burton, J. K., L. E. Craig, S. Q. Yong, N. Siddiqi, E. A. Teale, R. Woodhouse, A. J. Barugh, A. M. Shepherd, A. Brunton, S. C. Freeman, A. J. Sutton and T. J. Quinn | 2021 | Non-pharmacological interventions for preventing delirium in hospitalised non-ICU patients |
| Cattazzo, F., M. F. Pengo, A. Giontella, D. Soranna, J. Karalliedde, L. Gnudi, P. Minuz, C. Lombardi, G. Parati and C. Fava | 2021 | EFFECT OF CONTINUOUS POSITIVE AIRWAY PRESSURE TREATMENT ON GLYCEMIC AND LIPID PROFILES IN PATIENTS WITH OBSTRUCTIVE SLEEP APNEA: A SYSTEMATIC REVIEW AND META-ANALYSIS |
| Chen, T. A., S. T. Mao, H. C. Lin, W. T. Liu, K. W. Tam, C. Y. Tsai and Y. C. Kuan | 2023 | Effects of inspiratory muscle training on blood pressure- and sleep-related outcomes in patients with obstructive sleep apnea: a meta-analysis of randomized controlled trials |
| Corso, R., V. Russotto, C. Gregoretti and D. Cattano | 2018 | Perioperative management of obstructive sleep apnea: a systematic review |
| Durán-Cantolla, J., F. Aizpuru, C. Martínez-Null and F. Barbé-Illa | 2009 | Obstructive sleep apnea/hypopnea and systemic hypertension |
| Feldstein, C. A. | 2016 | Blood pressure effects of CPAP in nonresistant and resistant hypertension associated with OSA: A systematic review of randomized clinical trials |
| Green, M., G. Ken-Dror, D. Fluck, C. Sada, P. Sharma, C. H. Fry and T. S. Han | 2021 | Meta-analysis of changes in the levels of catecholamines and blood pressure with continuous positive airway pressure therapy in obstructive sleep apnea |
| Green, M., G. Ken-Dror, D. Fluck, C. Sada, P. Sharma, C. H. Fry and T. S. Han | 2021 | Meta-analysis of changes in the levels of catecholamines and blood pressure with continuous positive airway pressure therapy in obstructive sleep apnea |
| Grewal, N., D. Gordon, S. Bajaj, C. Gyimah, M. Hassan, U. Fatima and P. P. Mehrotra | 2024 | Impact of Obstructive Sleep Apnea Treatment on Cardiovascular Disease Associated Mortality and Morbidity: A Systematic Review |
| Grewal, N., D. Gordon, S. Bajaj, C. Gyimah, M. Hassan, U. Fatima and P. P. Mehrotra | 2024 | Impact of Obstructive Sleep Apnea Treatment on Cardiovascular Disease Associated Mortality and Morbidity: A Systematic Review |
| Haentjens, P., A. Van Meerhaeghe, A. Moscariello, S. De Weerdt, K. Poppe, A. Dupont and B. Velkeniers | 2007 | The impact of continuous positive airway pressure on blood pressure in patients with obstructive sleep apnea syndrome - Evidence from a meta-analysis of placebo-controlled randomized trials |
| Hilmisson, H. and S. Magnusdottir | 2019 | Beyond the apnea hypopnea index (AHI): importance of sleep quality management of obstructive sleep apnea (OSA) and related mortality in patients with cardiovascular disease |
| Hu, X. Y., J. Q. Fan, S. J. Chen, Y. H. Yin and B. Zrenner | 2015 | The Role of Continuous Positive Airway Pressure in Blood Pressure Control for Patients With Obstructive Sleep Apnea and Hypertension: A Meta-Analysis of Randomized Controlled Trials |
| Iftikhar, I. H., E. R. Hays, M. A. Iverson, U. J. Magalang and A. K. Maas | 2013 | Effect of Oral Appliances on Blood Pressure in Obstructive Sleep Apnea: A Systematic Review and Meta-analysis |
| Iftikhar, I. H., C. Valentine, L. Bittencourt, D. Cohen, A. C. Fedson, T. Gislason, T. Penzel, C. L. Phillips, L. Yu-Sheng, A. I. Pack, U. J. Magalang and Sagic | 2014 | Effects Of Continuous Positive Airway Pressure On Blood Pressure In Patients With Resistant Hypertension And Obstructive Sleep Apnea: A Meta-Analysis |
| Iftikhar, I. H., C. W. Valentine, L. R. A. Bittencourt, D. L. Cohen, A. C. Fedson, T. Gíslason, T. Penzel, C. L. Phillips, Y. S. Lin, A. I. Pack and U. J. Magalang | 2014 | Effects of continuous positive airway pressure on blood pressure in patients with resistant hypertension and obstructive sleep apnea: ameta-analysis |
| Imran, T. F., M. Ghazipura, S. Liu, T. Hossain, H. Ashtyani, B. Kim, J. M. Gaziano and L. Djoussé | 2016 | Effect of continuous positive airway pressure treatment on pulmonary artery pressure in patients with isolated obstructive sleep apnea: a meta-analysis |
| Jaafar, M., A. Godhamgaonkar, S. Alsanjari and M. Protty | 2025 | The role of cardiac magnetic resonance imaging in obstructive sleep apnea: a systematic scoping review |
| Ken-Dror, G., C. H. Fry, P. Murray, D. Fluck and T. S. Han | 2021 | Changes in cortisol levels by continuous positive airway pressure in patients with obstructive sleep apnoea: Meta-analysis of 637 individuals |
| Ken-Dror, G., C. H. Fry, P. Murray, D. Fluck and T. S. Han | 2021 | Changes in cortisol levels by continuous positive airway pressure in patients with obstructive sleep apnoea: Meta-analysis of 637 individuals |
| Kou, C., X. Zhao, X. Lin, X. Fan, Q. Wang and J. Yu | 2022 | Effect of different treatments for obstructive sleep apnoea on blood pressure |
| Kou, C. K., X. Zhao, X. Lin, X. Fan, Q. Y. Wang and J. Yu | 2022 | Effect of different treatments for obstructive sleep apnoea on blood pressure |
| Kovács, D. K., N. Gede, L. Szabó, P. Hegyi, Z. Szakács, B. Faludi, A. Sebok, A. Garami, M. Solymár, D. Kósa, L. Hanák, Z. Rumbus and M. Balaskó | 2022 | Weight reduction added to CPAP decreases blood pressure and triglyceride level in OSA: Systematic review and meta-analysis |
| Kovács, D. K., N. Gede, L. Szabó, P. Hegyi, Z. Szakács, B. Faludi, Á. Sebők, A. Garami, M. Solymár, D. Kósa, L. Hanák, Z. Rumbus and M. Balaskó | 2022 | Weight reduction added to CPAP decreases blood pressure and triglyceride level in OSA: Systematic review and meta-analysis |
| Labarca, G., R. Cruz and J. Jorquera | 2018 | Continuous Positive Airway Pressure in Patients With Obstructive Sleep Apnea and Non-Alcoholic Steatohepatitis: A Systematic Review and Meta-Analysis |
| Labarca, G., T. Reyes, J. Jorquera, J. Dreyse and L. Drake | 2018 | CPAP in patients with obstructive sleep apnea and type 2 diabetes mellitus: Systematic review and meta-analysis |
| Labarca, G., A. Schmidt, J. Dreyse, J. Jorquera, D. Enos, G. Torres and F. Barbe | 2021 | Efficacy of continuous positive airway pressure (CPAP) in patients with obstructive sleep apnea (OSA) and resistant hypertension (RH): Systematic review and meta-analysis |
| Labarca, G., A. Schmidt, J. Dreyse, J. Jorquera, D. Enos, G. Torres and F. Barbe | 2021 | Efficacy of continuous positive airway pressure (CPAP) in patients with obstructive sleep apnea (OSA) and resistant hypertension (RH): Systematic review and meta-analysis |
| Lee, Y. C., Y. C. Chang, L. W. Tseng, W. N. Lin, C. T. Lu, L. A. Lee, T. J. Fang, W. N. Cheng and H. Y. Li | 2024 | Continuous Positive Airway Pressure Treatment and Hypertensive Adverse Outcomes in Pregnancy A Systematic Review and Meta-Analysis |
| Lee, Y. C., Y. C. Chang, L. W. Tseng, W. N. Lin, C. T. Lu, L. A. Lee, T. J. Fang, W. N. Cheng and H. Y. Li | 2024 | Continuous Positive Airway Pressure Treatment and Hypertensive Adverse Outcomes in Pregnancy: A Systematic Review and Meta-Analysis |
| Lei, Q., Y. H. Lv, K. Li, L. Ma, G. D. Du, Y. Xiang and X. Q. Li | 2017 | Effects of continuous positive airway pressure on blood pressure in patients with resistant hypertension and obstructive sleep apnea: a systematic review and meta-analysis of six randomized controlled trials |
| Lin, X., G. P. Chen, J. C. Qi, X. F. Chen, J. M. Zhao and Q. C. Lin | 2016 | Effect of continuous positive airway pressure on arterial stiffness in patients with obstructive sleep apnea and hypertension: a meta-analysis |
| Liu, J. N., J. H. Xu, S. B. Guan and W. Wang | 2024 | Effects of different treatments on metabolic syndrome in patients with obstructive sleep apnea: a meta-analysis |
| Liu, L., Q. Cao, Z. Guo and Q. Dai | 2016 | Continuous Positive Airway Pressure in Patients With Obstructive Sleep Apnea and Resistant Hypertension: A Meta-Analysis of Randomized Controlled Trials |
| Lv, M. J., J. Mao, S. K. Wang, C. Y. Zhang, C. T. Qian, R. T. Zhu, S. K. Xiong, Y. Zhang and L. R. Guo | 2024 | Effect of continuous positive airway pressure on cardiometabolic risk factors in patients with obstructive sleep apnea: A systematic review and meta-analysis |
| Maier, L. E., B. A. Matenchuk, A. Vucenovic, A. Sivak, M. H. Davenport and C. D. Steinback | 2022 | Influence of Obstructive Sleep Apnea Severity on Muscle Sympathetic Nerve Activity and Blood Pressure: a Systematic Review and Meta-Analysis |
| Mason, M., E. J. Welsh and I. Smith | 2013 | Drug therapy for obstructive sleep apnoea in adults |
| McDaid, C., K. H. Duree, S. C. Griffin, H. L. A. Weatherly, J. R. Stradling, R. J. O. Davies, M. J. Sculpher and M. E. Westwood | 2009 | A systematic review of continuous positive airway pressure for obstructive sleep apnoea-hypopnoea syndrome |
| Miller, M. A. and F. P. Cappuccio | 2021 | A systematic review of COVID-19 and obstructive sleep apnoea |
| Mo, L. and Q. He | 2007 | Effect of long-term continuous positive airway pressure ventilation on blood pressure in patients with obstructive sleep apnea hypopnea syndrome: A meta-analysis of clinical trials |
| Mo, L. and Q. Y. He | 2007 | [Effect of long-term continuous positive airway pressure ventilation on blood pressure in patients with obstructive sleep apnea hypopnea syndrome: a meta-analysis of clinical trials] |
| Montesi, S. B., B. A. Edwards, A. Malhotra and J. P. Bakker | 2012 | The Effect of Continuous Positive Airway Pressure Treatment on Blood Pressure: A Systematic Review and Meta-Analysis of Randomized Controlled Trials |
| Nugent, R., A. Wee, L. Kearney and C. de Costa | 2023 | The effectiveness of continuous positive airway pressure for treating obstructive sleep apnoea in pregnancy: A systematic review |
| Nugent, R., A. Wee, L. Kearney and C. de Costa | 2023 | The effectiveness of continuous positive airway pressure for treating obstructive sleep apnoea in pregnancy: A systematic review |
| Pagel, J. F. | 2007 | Obstructive sleep apnea (OSA) in primary care: Evidence-based practice |
| Parati, G., M. F. Pengo and C. Lombardi | 2019 | Obstructive Sleep Apnea and Hypertension: Why Treatment Does Not Consistently Improve Blood Pressure |
| Pengo, M., E. I. Schwarz, F. Barbe, L. Drager, C. Fava, M. S. M. Ip, M. A. Martinez Garcia, D. McEvoy, Y. Peker, C. L. Phillips, D. Soranna, J. Steier, J. Stradling, A. Zambon and G. Parati | 2022 | Effect of CPAP therapy on blood pressure in patients with OSA: A worldwide individual patient data meta-analysis |
| Pengo, M. F., E. I. Schwarz, F. Barbé, P. A. Cistulli, L. F. Drager, C. Fava, F. D. Fuchs, M. S. M. Ip, K. A. Loffler, M. M. S. Lui, M. Martínez-García, D. McEvoy, Y. Peker, C. L. Phillips, T. Quinnell, D. Soranna, J. Steier, J. R. Stradling, A. Zambon and G. Parati | 2025 | Effect of CPAP therapy on blood pressure in patients with obstructive sleep apnoea: a worldwide individual patient data meta-analysis |
| Pengo, M. F., E. I. Schwarz, F. Barbé, P. A. Cistulli, L. F. Drager, C. Fava, F. D. Fuchs, M. S. M. Ip, K. A. Loffler, M. M. S. Lui, M. A. Martínez-García, D. McEvoy, Y. Peker, C. L. Phillips, T. Quinnell, D. Soranna, J. Steier, J. R. Stradling, A. Zambon, G. Parati and A. collaborators | 2025 | Effect of CPAP therapy on blood pressure in patients with obstructive sleep apnoea: a worldwide individual patient data meta-analysis |
| Pengo, M. F., J. Steier, G. Parati, N. T. Ayas, F. Barbé, M. Barnes, F. Campos-Rodriguez, P. Cistulli, P. Lloberes, S. Craig, L. F. Drager, J. Durán-Cantolla, Litvin, A. Evgeniya Elfimova, T. Esra, A. Giontella, C. Fava, S. C. Fuchs, F. D. Fuchs, F. Gagnadoux, F. García-Río, Javaheri, S. Daniel J Gottlieb, G. R. Hong, R. R. Grunstein, C. M. Hoyos, M. Joyeux-Faure, P. Y. Liu, G. Lorenzi-Filho, M. S. M. Ip, M. M. S. Lui, M. Á. Martínez-García, D. McEvoy, R. Mehra, C. Monasterio, A. McMillan, M. J. Morrell, S. Pamidi, G. Bilo, C. Lombardi, Y. Peker, M. Glos, T. Penzel, J. L. Pépin, J. C. T. Pepperell, C. L. Phillips, S. F. Quan, T. Quinnell, G. F. Salles, N. Salord, M. Sánchez-de-la-Torre, E. Irene Schwarz, J. E. Shaw, G. Shukla, J. Stradling, E. Thunström, J. G. Wang, T. E. Weaver, A. Zambon, D. Soranna and G. Zambra | 2021 | The ANDANTE Project: A Worldwide Individual Data Meta-Analysis of the Effect of Sleep Apnea Treatment on Blood Pressure |
| Pintilie, A. L., D. T. M. Marcu, A. Zabara-Antal, R. I. Arcana, D. G. Iosep, M. Miron, C. A. Afloarei, M. L. Zabara and R. C. Dabija | 2025 | Sleep Apnea: The Slept-Upon Cardiovascular Risk Factor |
| Sáanchez-de-la-Torre, M., E. Gracia-Lavedan, I. D. Benitez, A. Sánchez-de-la-Torre, A. Moncusí-Moix, G. Torres, K. Loffler, R. Woodman, R. Adams, G. Labarca, J. Dreyse, C. Eulenburg, E. Thunström, H. Glantz, Y. Peker, C. Anderson, D. McEvoy and F. Barbé | 2023 | Adherence to CPAP Treatment and the Risk of Recurrent Cardiovascular Events A Meta-Analysis |
| Sánchez-de-la-Torre, M., E. Gracia-Lavedan, I. D. Benitez, A. Sánchez-de-la-Torre, A. Moncusí-Moix, G. Torres, K. Loffler, R. Woodman, R. Adams, G. Labarca, J. Dreyse, C. Eulenburg, E. Thunström, H. Glantz, Y. Peker, C. Anderson, D. McEvoy and F. Barbé | 2023 | Adherence to CPAP Treatment and the Risk of Recurrent Cardiovascular Events: A Meta-Analysis |
| Sarvananda, S., S. Earnshaw, I. Hughes, P. Sivakumaran and K. B. Sriram | 2025 | Effect of positive airway pressure treatment on pulmonary artery pressure in obstructive sleep apnoea and/or obesity hypoventilation syndrome with pulmonary hypertension: a systematic review and meta-analysis |
| Schein, A. S. O., A. C. Kerkhoff, C. C. Coronel, R. D. M. Plentz and G. Sbruzzi | 2014 | Continuous positive airway pressure reduces blood pressure in patients with obstructive sleep apnea; a systematic review and meta-analysis with 1000 patients |
| Shang, W. L., Y. Y. Zhang, L. Liu, F. F. Chen, G. Z. Wang and D. Han | 2022 | Benefits of continuous positive airway pressure on blood pressure in patients with hypertension and obstructive sleep apnea: a meta-analysis |
| Shantha, G. P. S. and S. B. Pancholy | 2015 | Effect of renal sympathetic denervation on apnea-hypopnea index in patients with obstructive sleep apnea: a systematic review and meta-analysis |
| Sun, L., Y. F. Chang, Y. F. Wang, Q. X. Xie, X. Z. Ran, C. Y. Hu, B. Luo and B. Ning | 2024 | Effect of Continuous Positive Airway Pressure on Blood Pressure in Patients with Resistant Hypertension and Obstructive Sleep Apnea: An Updated Meta-analysis |
| Sun, L., Y. F. Chang, Y. F. Wang, Q. X. Xie, X. Z. Ran, C. Y. Hu, B. Luo and B. Ning | 2024 | Effect of Continuous Positive Airway Pressure on Blood Pressure in Patients with Resistant Hypertension and Obstructive Sleep Apnea: An Updated Meta-analysis |
| Sun, X., J. Luo and Y. Wang | 2021 | Comparing the effects of supplemental oxygen therapy and continuous positive airway pressure on patients with obstructive sleep apnea: a meta-analysis of randomized controlled trials |
| Sun, X., J. Luo and Y. Wang | 2021 | Comparing the effects of supplemental oxygen therapy and continuous positive airway pressure on patients with obstructive sleep apnea: a meta-analysis of randomized controlled trials |
| Sun, X. F., J. M. Luo and Y. Xiao | 2014 | Continuous positive airway pressure is associated with a decrease in pulmonary artery pressure in patients with obstructive sleep apnoea: A meta-analysis |
| Sun, Y., Z. Y. Huang, Q. R. Sun, L. P. Qiu, T. T. Zhou and G. H. Zhou | 2016 | CPAP therapy reduces blood pressure for patients with obstructive sleep apnoea: an update meta-analysis of randomized clinical trials |
| Sun, Y., Z. Y. Huang, Q. R. Sun, L. P. Qiu, T. T. Zhou and G. H. Zhou | 2016 | CPAP therapy reduces blood pressure for patients with obstructive sleep apnoea: an update meta-analysis of randomized clinical trials |
| Umeda, A., K. Miyagawa, A. Mochida, H. Takeda, K. Takeda, Y. Okada and D. Gozal | 2020 | Effects of Normoxic Recovery on Intima-Media Thickness of Aorta and Pulmonary Artery Following Intermittent Hypoxia in Mice |
| Umeda, A., K. Miyagawa, A. Mochida, H. Takeda, K. Takeda, Y. Okada and D. Gozal | 2020 | Effects of Normoxic Recovery on Intima-Media Thickness of Aorta and Pulmonary Artery Following Intermittent Hypoxia in Mice |
| 邓刚, 邱占东, 李大勇, 方瑜 and 张苏明 | 2016 | Effects of Continuous Positive Airway Pressure Therapy on Plasma Aldosterone Levels in Patients with Obstructive Sleep Apnea:A Meta-analysis |
| 方明亮, 李松桃, 刘家勉, 张文勇, 杨炜 and 刘剑雄 | 2016 | 持续气道正压通气对合并阻塞性睡眠呼吸暂停的难治性高血压疗效的Meta分析 |
| 方明亮, 李松桃, 刘家勉, 张文勇, 杨炜 and 刘剑雄 | 2016 | 持续气道正压通气对合并阻塞性睡眠呼吸暂停的难治性高血压疗效的Meta分析 |
| 符翠萍, 朱芬, 刘子龙, 励雯静, 吴晓丹, 吴旭, 张舒琪, 白春学, 周新 and 李善群 | 2014 | 持续气道正压通气对阻塞性睡眠呼吸暂停低通气综合征患者血压影响的荟萃分析 |
| 韩媛媛, 孟凡华, 张俊仕, 珠勒皮亚.司马义, 徐新娟, 张向阳 and 陈玉岚 | 2016 | 持续气道正压通气治疗顽固性高血压伴阻塞性睡眠呼吸暂停疗效的Meta分析 |
| 韩媛媛, 孟凡华, 张俊仕, 珠勒皮亚·司马义, 徐新娟, 张向阳 and 陈玉岚 | 2016 | 持续气道正压通气治疗顽固性高血压伴阻塞性睡眠呼吸暂停疗效的Meta分析 |
| 胡鑫渝 | 2015 | 持续正压通气治疗阻塞性睡眠呼吸暂停综合征合并高血压的降压疗效 |
| 雷强, 吕云辉, 李凯, 马磊, 杜国栋, 相艳 and 李许卿 | 2016 | 持续正压通气对阻塞性睡眠呼吸暂停合并难治性高血压患者血压影响的Meta分析 |
| 雷强, 吕云辉, 李凯, 马磊, 杜国栋, 相艳 and 李许卿 | 2016 | 持续正压通气对阻塞性睡眠呼吸暂停合并难治性高血压患者血压影响的Meta分析 |
| 雷强, 吕云辉, 李凯, 孙睿, 黄代金, 相艳, 马磊 and 苗志斌 | 2017 | 基于Meta分析的CPAP联合常规降压药物对OSAHS合并高血压患者的综合疗效分析 |
| 雷强, 吕云辉, 李凯, 孙睿, 黄代金, 相艳, 马磊 and 苗志斌 | 2017 | 基于Meta分析的CPAP联合常规降压药物对OSAHS合并高血压患者的综合疗效分析 |
| 李君, 李晓艳, 蒋学俊, 万为国 and 刘万里 | 2015 | 持续气道正压通气对阻塞性睡眠呼吸暂停综合征患者降压疗效的Meta分析 |
| 李君, 李晓艳, 蒋学俊, 万为国 and 刘万里 | 2015 | 持续气道正压通气对阻塞性睡眠呼吸暂停综合征患者降压疗效的Meta分析 |
| 刘剑雄, 方. A. 李. A. 刘. A. 张. A. 杨. A. | 2016 | 持续气道正压通气对合并阻塞性睡眠呼吸暂停的难治性高血压疗效的Meta分析 |
| 罗英, 宁莉萍, 史菲菲, 何梅, 杨卜凡 and 崔丽君 | 2024 | 阻塞性睡眠呼吸暂停患者持续气道正压通气治疗依从性及影响因素的Meta分析 |
| 苗志斌, 雷. A. 吕. A. 李. A. 孙. A. 黄. A. 相. A. 马. A. | 2017 | 基于Meta分析的CPAP联合常规降压药物对OSAHS合并高血压患者的综合疗效分析 |
| 缪碧芳, 沈宁, 肖建宏, 彭锦芸, 曾林淼 and 胡良平 | 2016 | 持续气道正压通气治疗对阻塞性睡眠呼吸暂停低通气综合征合并高血压患者降压效果的Meta分析 |
| 尹富禹 | 2013 | 持续气道正压通气治疗对阻塞性睡眠呼吸暂停综合症患者血压影响的Meta分析 |

****Thematic Deviation****

|  | 2014 | Clinical effectiveness and cost-effectiveness results from the randomised controlled Trial of Oral Mandibular Advancement Devices for Obstructive sleep apnoea-hypopnoea (TOMADO) and long-term economic analysis of oral devices and continuous positive airway pressure |
| --- | --- | --- |
| Amen, S., B. Rasool, B. S. Al Lami, C. Gamal Shehata, A. N. Mohammad, P. Maaroof, R. M. Abdullah, R. Subedi and R. Al-Lami | 2024 | Obstructive Sleep Apnea and Cardiovascular Diseases: A Systematic Review and Meta-Analysis of Prospective Studies |
| Aslan, G., B. Afsar, D. Siriopol, A. Kanbay, O. Sal, C. Benli, J. Okcuoglu, A. Covic and M. Kanbay | 2018 | Cardiovascular Effects of Continuous Positive Airway Pressure Treatment in Patients With Obstructive Sleep Apnea: A Meta-Analysis |
| Bratton, D. J., T. Gaisl, A. M. Wons and M. Kohler | 2015 | CPAP vs Mandibular Advancement Devices and Blood Pressure in Patients With Obstructive Sleep Apnea A Systematic Review and Meta-analysis |
| Feng, G. F., P. Zhuge, Z. F. Zhang and J. X. Ma | 2024 | The impact of continuous positive airway pressure therapy on cardiovascular events in patients with obstructive sleep apnoea: an updated systematic review and meta-analysis |
| Fridriksson, B., M. Berndtson, H. Hamnered, E. Faeder, D. Zou, J. Hedner and L. Grote | 2023 | Beneficial Effects of Early Intervention Telemedicine-based Follow-Up in Sleep Apnea A Randomized Controlled Multicenter Trial |
| Guo, J., Y. Sun, L. J. Xue, Z. Y. Huang, Y. S. Wang, L. Zhang, G. H. Zhou and L. X. Yuan | 2016 | Effect of CPAP therapy on cardiovascular events and mortality in patients with obstructive sleep apnea: a meta-analysis |
| Kou, C., X. Zhao, X. Lin, X. Fan, Q. Wang and J. Yu | 2022 | Effect of different treatments for obstructive sleep apnoea on blood pressure |
| Kovács, D. K., N. Gede, L. Szabó, P. Hegyi, Z. Szakács, B. Faludi, Á. Sebők, A. Garami, M. Solymár, D. Kósa, L. Hanák, Z. Rumbus and M. Balaskó | 2022 | Weight reduction added to CPAP decreases blood pressure and triglyceride level in OSA: Systematic review and meta-analysis |
| Lei, Q., Y. Lv, K. Li, L. Ma, G. Du, Y. Xiang and X. Li | 2017 | Effects of continuous positive airway pressure on blood pressure in patients with resistant hypertension and obstructive sleep apnea: a systematic review and meta-analysis of six randomized controlled trials |
| Liu, T. W., W. Y. Li, H. Zhou and Z. F. Wang | 2017 | Verifying the Relative Efficacy between Continuous Positive Airway Pressure Therapy and Its Alternatives for Obstructive Sleep Apnea: A Network Meta-analysis |
| Lv, M., J. Mao, S. Wang, C. Zhang, C. Qian, R. Zhu, S. Xiong, Y. Zhang and L. Guo | 2024 | Effect of continuous positive airway pressure on cardiometabolic risk factors in patients with obstructive sleep apnea: A systematic review and meta-analysis |
| McDaid, C., S. Griffin, H. Weatherly, K. Duree, M. van der Burgt, S. van Hout, J. Akers, R. J. O. Davies, M. Sculpher and M. Westwood | 2009 | Continuous positive airway pressure devices for the treatment of obstructive sleep apnoea-hypopnoea syndrome: a systematic review and economic analysis |
| Migueis, D. P., A. Urel, C. C. dos Santos, A. Accetta and M. Burla | 2022 | The cardiovascular, metabolic, fetal and neonatal effects of CPAP use in pregnant women: a systematic review |
| Pengo, M. F., E. I. Schwarz, F. Barbé, P. A. Cistulli, L. F. Drager, C. Fava, F. D. Fuchs, M. S. M. Ip, K. A. Loffler, M. M. S. Lui, M. Á. Martínez-García, D. McEvoy, Y. Peker, C. L. Phillips, T. Quinnell, J. Steier, J. R. Stradling, A. Zambon, G. Parati, N. T. Ayas, M. Barnes, F. Campos-Rodriguez, P. Lloberes, S. Craig, J. Durán-Cantolla, A. Litvin, E. Elfimova, A. Giontella, S. C. Fuchs, F. Gagnadoux, F. García-Río, S. Javaheri, D. J. Gottlieb, R. R. Grunstein, G. R. Hong, A. Gupta, C. M. Hoyos, M. Joyeux-Faure, P. Y. Liu, G. Lorenzi-Filho, R. Mehra, C. Monasterio, A. McMillan, M. J. Morrell, S. Pamidi, G. Bilo, C. Lombardi, M. Glos, T. Penzel, J. L. Pépin, J. C. T. Pepperell, S. F. Quan, G. F. Salles, N. Salord, M. Sánchez-De-La-Torre, J. E. Shaw, G. Shukla, E. Tasali, E. Thunström, J. G. Wang, T. E. Weaver and D. Soranna | 2025 | Effect of CPAP therapy on blood pressure in patients with obstructive sleep apnoea: A worldwide individual patient data meta-analysis |
| Posadas, T., F. Campos-Rodriguez, E. Sapiña-Beltrán, G. Oscullo, G. Torres and M. A. Martinez-Garcia | 2020 | Obstructive Sleep Apnea and Arterial Hypertension: Implications of Treatment Adherence |
| Sawunyavisuth, B., C. Ngamjarus and K. Sawanyawisuth | 2022 | A meta-analysis to identify factors associated with CPAP machine purchasing in patients with obstructive sleep apnea |
| Sun, L., Y. F. Chang, Y. F. Wang, Q. X. Xie, X. Z. Ran, C. Y. Hu, B. Luo and B. Ning | 2024 | Effect of Continuous Positive Airway Pressure on Blood Pressure in Patients with Resistant Hypertension and Obstructive Sleep Apnea: An Updated Meta-analysis |
| Venema, J., G. E. Knol-de Vries, H. van Goor, J. Westra, A. Hoekema and P. J. Wijkstra | 2022 | Cardiovascular and metabolic effects of a mandibular advancement device and continuous positive airway pressure in moderate obstructive sleep apnea: a randomized controlled trial |
| Xia, J. H., Y. Y. Kang, Y. B. Cheng, Q. F. Huang, T. Y. Xu, Y. Li and J. G. Wang | 2021 | Continuous positive airway pressure adherence and blood pressure lowering in patients with obstructive sleep apnoea syndrome and nocturnal hypertension |
| 方明亮, 李松桃, 刘家勉, 张文勇, 杨炜 and 刘剑雄 | 2016 | 持续气道正压通气对合并阻塞性睡眠呼吸暂停的难治性高血压疗效的Meta分析 |
| 韩翠华, 陈丽霞, 陈思涵, 李建华, 郭宁, 徐璐, 马莲莉, 卫来, 张琦, 梁燕 and 纪代红 | 2023 | 非手术干预对阻塞性睡眠呼吸暂停病人血压影响的网状Meta分析 |
| 韩媛媛, 孟凡华, 张俊仕, 珠勒皮亚·司马义, 徐新娟, 张向阳 and 陈玉岚 | 2016 | 持续气道正压通气治疗顽固性高血压伴阻塞性睡眠呼吸暂停疗效的Meta分析 |
| 匡亚辉, 许燕玲, 易红良 and 方芳 | 2016 | 健康教育干预对阻塞性睡眠呼吸暂停低通气综合征患者CPAP治疗依从性Meta分析 |
| 雷强, 吕云辉, 李凯, 马磊, 杜国栋, 相艳 and 李许卿 | 2016 | 持续正压通气对阻塞性睡眠呼吸暂停合并难治性高血压患者血压影响的Meta分析 |
| 雷强, 吕云辉, 李凯, 孙睿, 黄代金, 相艳, 马磊 and 苗志斌 | 2017 | 基于Meta分析的CPAP联合常规降压药物对OSAHS合并高血压患者的综合疗效分析 |
| 路英进 and 丁彦春 | 2015 | 持续气道正压通气对顽固性高血压合并阻塞性睡眠呼吸暂停低通气综合征患者降压效果的Meta分析 |
| 缪碧芳, 沈宁, 肖建宏, 彭锦芸, 曾林淼 and 胡良平 | 2016 | 持续气道正压通气治疗对阻塞性睡眠呼吸暂停低通气综合征合并高血压患者降压效果的Meta分析 |

****Review****

| Agaltsov, M. V. and L. S. Korostovtseva | 2021 | Obstructive sleep apnea and cardiovascular comorbidity: modern discordance in assessing the effectiveness of CPAP-therapy against the pathogenetic mechanisms and cardiovascular diseases |
| --- | --- | --- |
| Ahmad, A. and S. C. Didia | 2020 | Effects of Sleep Duration on Cardiovascular Events |
| Ando, S. I. | 2018 | Influence of hypoxia induced by sleep disordered breathing in case of hypertension and atrial fibrillation |
| Askland, K., L. Wright, D. R. Wozniak, T. Emmanuel, J. Caston and I. Smith | 2020 | Educational, supportive and behavioural interventions to improve usage of continuous positive airway pressure machines in adults with obstructive sleep apnoea |
| Baguet, J. P., G. Barone-Rochette and J. L. Pépin | 2009 | Obstructive sleep apnea syndrome, hypertension and artery |
| Bradicich, M., M. F. Pengo, J. Steier and E. I. Schwarz | 2025 | Cardiovascular effects of obstructive sleep apnoea and effects of continuous positive airway pressure therapy: evidence from different study models |
| Bughin, F., M. Mendelson, D. Jaffuel, J. L. Pépin, F. Gagnadoux, F. Goutorbe, B. Abril, B. Ayoub, A. Aranda, K. Alagha, P. Pomiès, F. Roubille, J. Mercier, N. Molinari, Y. Dauvilliers, N. Hèraud and M. Hayot | 2023 | Impact of a telerehabilitation programme combined with continuous positive airway pressure on symptoms and cardiometabolic risk factors in obstructive sleep apnea patients |
| Calhoun, D. A. | 2010 | Obstructive Sleep Apnea and Hypertension |
| Chang, J. L., A. N. Goldberg, J. A. Alt, A. Mohammed, L. Ashbrook, D. Auckley, I. Ayappa, H. Bakhtiar, J. E. Barrera, B. L. Bartley, M. E. Billings, M. S. Boon, P. Bosschieter, I. Braverman, K. Brodie, C. Cabrera-Muffly, R. Caesar, M. B. Cahali, Y. Cai, M. Cao, R. Capasso, S. M. Caples, L. M. Chahine, C. P. Chang, K. W. Chang, N. Chaudhary, C. S. J. Cheong, S. Chowdhuri, P. A. Cistulli, D. Claman, J. Collen, K. C. Coughlin, J. Creamer, E. M. Davis, K. L. Dupuy-McCauley, M. L. Durr, M. Dutt, M. E. Ali, N. M. Elkassabany, L. J. Epstein, J. A. Fiala, N. Freedman, K. Gill, M. B. Gillespie, L. Golisch, N. Gooneratne, D. J. Gottlieb, K. K. Green, A. Gulati, I. Gurubhagavatula, N. Hayward, P. T. Hoff, O. M. G. Hoffmann, S. J. Holfinger, J. Hsia, C. Huntley, K. C. Huoh, P. Huyett, S. Inala, S. L. Ishman, T. K. Jella, A. M. Jobanputra, A. P. Johnson, M. R. Junna, J. T. Kado, T. M. Kaffenberger, V. K. Kapur, E. J. Kezirian, M. Khan, D. B. Kirsch, A. Kominsky, M. Kryger, A. D. Krystal, C. A. Kushida, T. J. Kuzniar, D. J. Lam, C. J. Lettieri, D. C. Lim, H. C. Lin, S. Y. C. Liu, S. G. MacKay, U. J. Magalang, A. Malhotra, M. P. Mansukhani, J. T. Maurer, A. M. May, R. B. Mitchell, B. Mokhlesi, A. E. Mullins, E. M. Nada, S. Naik, B. Nokes, M. D. Olson, A. I. Pack, E. B. Pang, K. P. Pang, S. P. Patil, E. Van de Perck, J. F. Piccirillo, G. W. Pien, A. J. Piper, A. Plawecki, M. Quigg, M. J. L. Ravesloot, S. Redline, B. W. Rotenberg, A. Ryden, K. F. Sarmiento, F. Sbeih, A. E. Schell, C. N. Schmickl, H. M. Schotland, R. J. Schwab, J. Seo, N. Shah, A. V. Shelgikar, I. Shochat, R. J. Soose, T. O. Steele, E. Stephens, C. Stepnowsky, K. P. Strohl, K. Sutherland, M. V. Suurna, E. Thaler, S. Thapa, O. M. Vanderveken, N. de Vries, E. M. Weaver, I. D. Weir, L. F. Wolfe, B. T. Woodson, C. H. J. Won, J. Xu, P. Yalamanchi, K. Yaremchuk, Y. Yeghiazarians, J. L. Yu, M. Zeidler and I. M. Rosen | 2023 | International Consensus Statement on Obstructive Sleep Apnea |
| Chen, R. K., C. Hong, Y. M. Zhou, A. L. Kuang, Y. T. Zhang, S. M. Qing, C. L. Liu and N. F. Zhang | 2017 | [Severe obstructive sleep apnea-hypopnea syndrome with dilated cardiomyopathy leading to pulmonary hypertension: case report and literature review] |
| Crinion, S. J., S. Ryan and W. T. McNicholas | 2017 | Obstructive sleep apnoea as a cause of nocturnal nondipping blood pressure: recent evidence regarding clinical importance and underlying mechanisms |
| Dissanayake, H. U., J. T. Colpani, K. Sutherland, W. Loke, A. Mohammadieh, Y. H. Ou, P. de Chazal, P. A. Cistulli and C. H. Lee | 2021 | Obstructive sleep apnea therapy for cardiovascular risk reduction-Time for a rethink? |
| Durán-Cantolla, J., F. Aizpuru, C. Martínez-Null and F. Barbé-Illa | 2009 | Obstructive sleep apnea/hypopnea and systemic hypertension |
| Feldstein, C. A. | 2016 | Blood pressure effects of CPAP in nonresistant and resistant hypertension associated with OSA: A systematic review of randomized clinical trials |
| Floras, J. S. and T. D. Bradley | 2007 | Treating obstructive sleep apnea: is there more to the story than 2 millimeters of mercury? |
| Friedman, O. and A. G. Logan | 2009 | The price of obstructive sleep apnea-hypopnea: hypertension and other ill effects |
| Jhamb, M., L. K. Brown and M. Unruh | 2014 | Resistant Hypertension in Obstructive Sleep Apnea: Is Continuous Positive Airway Pressure the Next Step? |
| Kasiakogias, A., C. Tsioufis, C. Thomopoulos, D. Aragiannis, M. Alchanatis, D. Tousoulis, V. Papademetriou, J. S. Floras and C. Stefanadis | 2013 | Effects of continuous positive airway pressure on blood pressure in hypertensive patients with obstructive sleep apnea: a 3-year follow-up |
| Lorenzi, G., F. R. Almeida and P. J. Strollo | 2017 | Treating OSA: Current and emerging therapies beyond CPAP |
| Marrone, O. and M. R. Bonsignore | 2019 | Decrease in blood pressure during continuous positive airway pressure treatment for obstructive sleep apnoea: still searching for predictive factors |
| Militi, A., R. Nucera, G. Chirieleison, L. Fiorillo, G. Cervino and M. Portelli | 2022 | Down Syndrome children with obstructive sleep apnea |
| Moro, J. A., L. Almenar, E. Fernandez-Fabrellas, S. Ponce, R. Blanquer and A. Salvador | 2009 | Hypertension and sleep apnea-hypopnea syndrome: changes in echocardiographic abnormalities depending on the presence of hypertension and treatment with CPAP |
| Osanai, S. | 2023 | Clinical Question: Can CPAP suppress cardiovascular events in resistant hypertension patients with obstructive sleep apnea? |
| Oscullo, G., E. Sapiña-Beltrán, G. Torres, E. Zaldivar, F. Barbé and M. A. Martinez-Garcia | 2019 | The Potential Role of Obstructive Sleep Apnoea in Refractory Hypertension |
| Oscullo, G., G. Torres, F. Campos-Rodriguez, T. Posadas, A. Reina-González, E. Sapiña-Beltrán, F. Barbé and M. A. Martinez-Garcia | 2019 | Resistant/Refractory Hypertension and Sleep Apnoea: Current Knowledge and Future Challenges |
| Parati, G., M. F. Pengo and C. Lombardi | 2019 | Obstructive Sleep Apnea and Hypertension: Why Treatment Does Not Consistently Improve Blood Pressure |
| Patil, S. P., I. A. Ayappa, S. M. Caples, R. J. Kimoff, S. R. Patel and C. G. Harrod | 2019 | Treatment of Adult Obstructive Sleep Apnea with Positive Airway Pressure: An American Academy of Sleep Medicine Clinical Practice Guideline |
| Pengo, M. F., J. Steier, G. Parati, N. T. Ayas, F. Barbé, M. Barnes, F. Campos-Rodriguez, P. Cistulli, P. Lloberes, S. Craig, L. F. Drager, J. Durán-Cantolla, Litvin, A. Evgeniya Elfimova, T. Esra, A. Giontella, C. Fava, S. C. Fuchs, F. D. Fuchs, F. Gagnadoux, F. García-Río, Javaheri, S. Daniel J Gottlieb, G. R. Hong, R. R. Grunstein, C. M. Hoyos, M. Joyeux-Faure, P. Y. Liu, G. Lorenzi-Filho, M. S. M. Ip, M. M. S. Lui, M. Á. Martínez-García, D. McEvoy, R. Mehra, C. Monasterio, A. McMillan, M. J. Morrell, S. Pamidi, M. Pengo, G. Bilo, C. Lombardi, Y. Peker, M. Glos, T. Penzel, J. L. Pépin, J. C. T. Pepperell, C. L. Phillips, S. F. Quan, T. Quinnell, G. F. Salles, N. Salord, M. Sánchez-de-la-Torre, E. Irene Schwarz, J. E. Shaw, G. Shukla, J. Stradling, E. Thunström, J. G. Wang, T. E. Weaver, A. Zambon, D. Soranna and G. Zambra | 2021 | The ANDANTE Project: A Worldwide Individual Data Meta-Analysis of the Effect of Sleep Apnea Treatment on Blood Pressure |
| Pintilie, A. L., D. T. M. Marcu, A. Zabara-Antal, R. I. Arcana, D. G. Iosep, M. Miron, C. A. Afloarei, M. L. Zabara and R. Crisan Dabija | 2025 | Sleep Apnea: The Slept-Upon Cardiovascular Risk Factor |
| Praud, J. P. | 2022 | Sleep-Disordered Breathing-Year in Review 2021 |
| Qaseem, A., J. E. C. Holty, D. K. Owens, P. Dallas, M. Starkey, P. Shekelle and P. Amer Coll | 2013 | Management of Obstructive Sleep Apnea in Adults: A Clinical Practice Guideline From the American College of Physicians |
| Rao, M., G. Rajda, S. Uppuluri, G. R. Beck, L. Liu and J. D. Bisognano | 2010 | The role of continuous positive airway pressure in the treatment of hypertension in patients with obstructive sleep apnea-hypoapnea syndrome: a review of randomized trials |
| Romigi, A. | 2022 | Deprescribing antihypertensive drugs after starting OSA primary therapy: "first do no net harm?" |
| Rosenberg, R., P. K. Schweitzer, J. Steier and J. L. Pepin | 2021 | Residual excessive daytime sleepiness in patients treated for obstructive sleep apnea: guidance for assessment, diagnosis, and management |
| Sapiña, E., G. Torres, F. Barbé and M. Sánchez-de-la-Torre | 2018 | The Use of Precision Medicine to Manage Obstructive Sleep Apnea Treatment in Patients with Resistant Hypertension: Current Evidence and Future Directions |
| Schwarz, E. I. | 2019 | Cardiovascular consequences of obstructive sleep apnea in different study models and novel perspectives |
| Semelka, M., J. Wilson and R. Floyd | 2016 | Diagnosis and Treatment of Obstructive Sleep Apnea in Adults |
| Seravalle, G. and G. Grassi | 2022 | Sleep Apnea and Hypertension |
| Shiina, K. | 2024 | Obstructive sleep apnea -related hypertension: a review of the literature and clinical management strategy |
| Sova, M., E. Sovova, M. Hobzova, J. Zapletalova, M. Kamasova and V. Kolek | 2015 | The effect of continuous positive airway pressure therapy on the prevalence of masked hypertension in obstructive sleep apnea patients |
| Tamisier, R. and P. Lévy | 2017 | Management of hypertension in obstructive sleep apnoea: predicting blood pressure reduction under continuous positive airway pressure |
| Tien, H. A. | 2019 | Arrhythmia and sleep apnea syndrome: State of the art |
| Uwishema, O., A. Nazir, I. Munyangaju, S. Shariff, O. Al Komi, N. Chibueze and M. Wojtara | 2024 | The pulse of sleep: Novel interventions in understanding the sleep-cardiovascular connection: A literature review |
| Wang, A. Y. M. | 2014 | Sleep-Disordered Breathing and Resistant Hypertension |
| Wons, A. M. and M. Kohler | 2015 | Established vascular effects of continuous positive airway pressure therapy in patients with obstructive sleep apnoea-an update |
| Yang, S. J., X. T. Jiang, X. B. Zhang, X. W. Yin and W. X. Deng | 2016 | Does continuous positive airway pressure reduce aldosterone levels in patients with obstructive sleep apnea? |
| Zhao, Y. Y. and S. Redline | 2015 | Impact of Continuous Positive Airway Pressure on Cardiovascular Risk Factors in High-Risk Patients |
| 陈海清 | 2011 | 阻塞型睡眠呼吸暂停综合征的临床观察研究 |
| 贾楠 and 李晓波 | 2014 | 顽固性高血压病因之一睡眠呼吸暂停综合征 |
| 马庆春 | 2016 | 心源性晕厥原来另有原因--高血压、冠心病合并阻塞性睡眠呼吸暂停综合征一例 |

****Search term****

**PubMed**

1. Continuous Positive Airway Pressure[MeSH Terms]

2.((((((((constant positive airway pressure[Title/Abstract]) OR (constant positive pressure breathing[Title/Abstract])) OR (constant positive pressure ventilation[Title/Abstract])) OR (continous positive airway pressure[Title/Abstract])) OR (continuous positive airway pressure ventilation[Title/Abstract])) OR (continuous positive pressure breathing[Title/Abstract])) OR (continuous positive pressure ventilation[Title/Abstract])) OR (CPAP[Title/Abstract])) OR (continuous positive airway pressure[Title/Abstract])

3.#1 OR #2

4.hypertension[MeSH Terms]

5.((((blood pressure, high[Title/Abstract]) OR (high blood pressure[Title/Abstract])) OR (HTN (hypertension[Title/Abstract]))) OR (hypertensive disease[Title/Abstract])) OR (hypertension[Title/Abstract])

6.#4 OR #5

7.(((((((sleep apnea syndromes[MeSH Terms]) OR (apnea during sleep[Title/Abstract])) OR (apnea syndrome[Title/Abstract])) OR (apnea syndromes[Title/Abstract])) OR (apnea, sleep[Title/Abstract])) OR (apneas during sleep[Title/Abstract])) OR (apnoea, sleep[Title/Abstract])) OR (hypopnea during sleep[Title/Abstract])

8.(((((((obstructive sleep apnea[MeSH Terms]) OR (obstructive apnea[Title/Abstract])) OR (obstructive apnea during sleep[Title/Abstract])) OR (obstructive apneas during sleep[Title/Abstract])) OR (obstructive apnoea[Title/Abstract])) OR (obstructive sleep apnea hypopnea syndrome[Title/Abstract])) OR (obstructive sleep apnea syndrome[Title/Abstract])) OR (obstructive sleep apneas[Title/Abstract])

9.#7 OR #8

10.(((((((Meta-Analysis[MeSH Terms]) OR ("systematic review"[Title/Abstract])) OR ("systematic literature review"[Title/Abstract])) OR ("meta analysis"[Title/Abstract])) OR (metaanalysis[Title/Abstract])) OR ("pooled analysis"[Title/Abstract])) OR ("data synthesis"[Title/Abstract])) OR ("systematic meta-analysis"[Title/Abstract])

11. #3 AND #6 AND #9 AND #10

**Web of science**

1. Continuous Positive Airway Pressure (Topic) or constant positive airway pressure (Topic) or constant positive pressure breathing (Topic) or constant positive pressure ventilation (Topic) or continous positive airway pressure (Topic) or continuous positive airway pressure ventilation (Topic) or continuous positive pressure breathing (Topic)
2. hypertension (Topic) or blood pressure, high (Topic) or high blood pressure (Topic) or hypertensive disease (Topic) or hypertension (Topic)
3. sleep apnea syndromes (Topic) or apnea during sleep (Topic) or apnea syndrome (Topic) or apnea syndromes (Topic) or apnea, sleep (Topic) or apneas during sleep (Topic) or apnoea, sleep (Topic)
4. obstructive sleep apnea (Topic) or obstructive apnea (Topic) or obstructive apnea during sleep (Topic) or obstructive apneas during sleep (Topic) or obstructive apnoea (Topic) or obstructive sleep apnea hypopnea syndrome (Topic) or obstructive sleep apnea syndrome (Topic) or obstructive sleep apneas (Topic)
5. Meta-Analysis (Topic) or systematic review (Topic) or systematic literature review (Topic) or meta analysis (Topic) or metaanalysis (Topic) or data synthesis (Topic) or systematic meta-analysis (Topic)
6. #3 OR #4
7. #1 AND #2 AND #5 AND #6

**Embase**

1. 'continuous positive airway pressure'/exp
2. ‘constant positive airway pressure':ti,ab,kw OR 'constant positive pressure breathing':ti,ab,kw OR 'constant positive pressure ventilation':ti,ab,kw OR 'continous positive airway pressure':ti,ab,kw OR 'continuous positive airway pressure ventilation':ti,ab,kw OR 'continuous positive pressure breathing':ti,ab,kw
3. #1 OR #2
4. 'hypertension'/exp
5. 'blood pressure,high':ti,ab,kw OR 'high blood pressure':ti,ab,kw OR 'hypertensive disease':ti,ab,kw OR 'hypertension':ti,ab,kw
6. #4 OR #5
7. 'sleep apnea syndromes'/exp
8. 'apnea during sleep':ti,ab,kw OR 'apnea syndrome':ti,ab,kw OR 'apnea syndromes':ti,ab,kw OR 'apnea, sleep':ti,ab,kw OR 'apneas during sleep':ti,ab,kw OR 'apnoea, sleep':ti,ab,kw
9. 'obstructive sleep apnea'/exp
10. 'obstructive apnea':ti,ab,kw OR 'obstructive apnea during sleep':ti,ab,kw OR 'obstructive apneas during sleep':ti,ab,kw OR 'obstructive apnoea':ti,ab,kw OR 'obstructive sleep apnea hypopnea syndrome':ti,ab,kw OR 'obstructive sleep apnea syndrome':ti,ab,kw OR 'obstructive sleep apneas':ti,ab,kw
11. #7 OR #8 OR #9 OR #10
12. 'meta analysis'/exp
13. ' meta analysis ':ti,ab,kw OR ' metaanalysis ':ti,ab,kw OR ' pooled analysis ':ti,ab,kw OR ' data synthesis ':ti,ab,kw
14. 'systematic review'/exp
15. 'systematic review ':ti,ab,kw OR ' systematic literature review ':ti,ab,kw OR ' systematic meta-analysis':ti,ab,kw
16. #12 OR #13 OR #14 OR #15
17. #3 AND #6 AND #11 AND #16

**Cochrane Library**

1. MeSH descriptor: [Continuous Positive Airway Pressure] explode all trees
2. (constant positive airway pressure):ti,ab,kw OR (constant positive pressure breathing):ti,ab,kw OR (constant positive pressure ventilation):ti,ab,kw OR (continous positive airway pressure):ti,ab,kw OR (continuous positive airway pressure ventilation):ti,ab,kw
3. #1 OR #2
4. MeSH descriptor: [Hypertension] explode all trees
5. (blood pressure,high):ti,ab,kw OR (high blood pressure):ti,ab,kw OR (hypertensive disease):ti,ab,kw OR (hypertension):ti,ab,kw
6. #4 OR #5
7. MeSH descriptor: [Sleep Apnea Syndromes] explode all trees
8. (apnea during sleep):ti,ab,kw OR (apnea syndrome):ti,ab,kw OR (apnea syndromes):ti,ab,kw OR (apnea, sleep):ti,ab,kw OR (apneas during sleep):ti,ab,kw
9. MeSH descriptor: [Sleep Apnea, Obstructive] explode all trees
10. (obstructive apnea):ti,ab,kw OR (obstructive apnea during sleep):ti,ab,kw OR (obstructive apneas during sleep):ti,ab,kw OR (obstructive apnoea):ti,ab,kw OR (obstructive sleep apnea hypopnea syndrome):ti,ab,kw
11. #7 OR #8 OR #9 OR #10
12. MeSH descriptor: [Meta-Analysis as Topic] explode all trees
13. (meta analysis):ti,ab,kw OR (metaanalysis):ti,ab,kw OR (pooled analysis):ti,ab,kw OR (data synthesis):ti,ab,kw
14. MeSH descriptor: [Systematic Reviews as Topic] explode all trees
15. (systematic review):ti,ab,kw OR (systematic literature review):ti,ab,kw OR (systematic meta-analysis):ti,ab,kw
16. #12 OR #13 OR #14 OR #15
17. #3 AND #6 AND #11 AND #16

**知网**

(SU=睡眠呼吸暂停综合征 OR SU=睡眠呼吸暂停症状 OR SU=睡眠呼吸暂停综合症 OR SU=睡眠呼吸暂停症 OR SU='阻塞性睡眠呼吸暂停(osa)' OR SU=重度阻塞性睡眠呼吸暂停 OR SU=阻塞性睡眠呼吸暂停 OR SU=睡眠呼吸暂停症 OR SU=成人阻塞性睡眠呼吸暂停 OR SU= 儿童睡眠呼吸暂停 OR SU=老年睡眠呼吸暂停) AND (SU=meta OR SU=meta OR SU=meta分析 OR SU='meta-analysis' OR SU='meta-分析' OR SU=meta分析法 OR SU=系统评价 OR SU=系统评价方法 OR SU=系统评价研究 OR SU=系统评价和meta分析 OR SU='系统评价/meta分析' OR SU=系统评价与meta分析) AND (SU=持续性正压通气 OR SU=持续性正压通气治疗 OR SU='持续性正压通气(cpap)' OR SU=cpap OR SU=cpap治疗 OR SU=cpap通气 OR SU=cpap模式) AND (SU=高血压 OR SU=高血压病 OR SU=高血压综合征 OR SU=高血压患者 OR SU=高血压病患者 OR SU=高血压疾病)

**万方**

主题:(睡眠呼吸暂停综合征 or 睡眠呼吸暂停症状 or 睡眠呼吸暂停综合症 or 睡眠呼吸暂停症 or 重度阻塞性睡眠呼吸暂停 or 阻塞性睡眠呼吸暂停 or 睡眠呼吸暂停症 or 成人阻塞性睡眠呼吸暂停 or 儿童睡眠呼吸暂停 or 老年睡眠呼吸暂停) and 主题:(meta or meta分析 or 系统评价 or 系统评价方法 or 系统评价研究 or 系统评价和meta分析 or 系统评价与meta分析) and 主题:(持续性正压通气 or 持续性正压通气治疗 or cpap or cpap治疗 or cpap通气 or cpap模式) and 主题:(高血压 or 高血压病 or 高血压综合征 or 高血压患者 or 高血压病患者 or 高血压疾病)

**维普**

(M=睡眠呼吸暂停综合征 OR M=睡眠呼吸暂停症状 OR M=睡眠呼吸暂停综合症 OR M=睡眠呼吸暂停症 OR M=重度阻塞性睡眠呼吸暂停 OR M=阻塞性睡眠呼吸暂停 OR M=睡眠呼吸暂停症 OR M=成人阻塞性睡眠呼吸暂停 OR M= 儿童睡眠呼吸暂停 OR M=老年睡眠呼吸暂停) AND (M=meta OR M=meta OR M=meta分析 OR M=meta analysis OR M=meta 分析 OR M=meta分析法 OR M=系统评价 OR M=系统评价方法 OR M=系统评价研究 OR M=系统评价和meta分析 OR M=系统评价/meta分析 OR M=系统评价与meta分析) AND (M=持续性正压通气 OR M=持续性正压通气治疗 OR M=cpap OR M=cpap治疗 OR M=cpap通气 OR M=cpap模式) AND (M=高血压 OR M=高血压病 OR M=高血压综合征 OR M=高血压患者 OR M=高血压病患者 OR M=高血压疾病)

**中国生物医学**

1. "睡眠呼吸暂停综合征"[不加权:扩展]
2. "睡眠呼吸暂停, 阻塞性"[不加权:扩展]
3. "睡眠呼吸暂停症状"[标题:智能] OR "睡眠呼吸暂停综合症"[标题:智能] OR "睡眠呼吸暂停症"[标题:智能]
4. "重度阻塞性睡眠呼吸暂停"[标题:智能] OR "阻塞性睡眠呼吸暂停"[标题:智能] OR "睡眠呼吸暂停症"[标题:智能] OR "成人阻塞性睡眠呼吸暂停"[标题:智能] OR "儿童睡眠呼吸暂停"[标题:智能] OR "老年睡眠呼吸暂停"[标题:智能]
5. (#1) OR (#2) OR (#3) OR (#4)
6. "连续气道正压通气"[不加权:扩展]
7. "持续性正压通气"[标题:智能] OR "持续性正压通气治疗"[标题:智能] OR "cpap"[标题:智能] OR "cpap治疗"[标题:智能] OR "cpap通气"[标题:智能] OR "cpap模式"[标题:智能]
8. (#6) OR (#7)
9. "Meta分析"[不加权:扩展]
10. ("系统评价(主题)"[不加权:扩展])
11. "meta"[标题:智能] OR "元分析"[标题:智能] OR "荟萃分析"[标题:智能] OR "网状meta"[标题:智能]
12. "系统评价方法"[标题:智能] OR "系统评价研究"[标题:智能]
13. (#9) OR (#10) OR (#11) OR (#12)
14. (#5) AND (#8) AND (#13)
