# Supplementary material for: Efficacy and safety of continuous positive airway pressure on blood pressure in patients with obstructive sleep apnea: an overview of systematic reviews and meta analyses
Source: Front Med (Lausanne). 2026 Mar 24;13:1785996. doi: 10.3389/fmed.2026.1785996 (PMC13055550; doi:10.3389/fmed.2026.1785996)
Supplement: Supplementary file 1 [file Table_1.DOCX]

Editorial Office

Frontiers in Medicine

Dear Editor,

I am pleased to submit our manuscript entitled, “Efficacy and safety of Continuous Positive Airway Pressure on Blood Pressure in Patients with Obstructive Sleep Apnea: an overview of systematic reviews and meta-analyses,” for consideration for publication in Frontiers in Medicine.

This study presents a comprehensive overview of systematic reviews and meta-analyses examining the impact of CPAP therapy on blood pressure in patients with OSA. Unlike previous narrative or single meta-analyses, our work synthesizes and critically appraises the existing evidence body using rigorous tools (ROBIS, AMSTAR-2, PRISMA 2020, and GRADE), providing a higher-level evaluation of evidence quality, methodological soundness, and clinical reliability. Key innovations include:

·Evidence integration across overlapping studies, quantified via corrected covered area (CCA) analysis, to address redundancy and enhance synthesis validity.

·Identification of phenotype-specific responders, highlighting CPAP’s pronounced benefits in patients with severe nocturnal hypoxia, higher AHI, and better adherence.

·Novel mechanistic insights into CPAP’s differential effects on nocturnal vs. daytime blood pressure, supporting its role in circadian blood pressure regulation and long-term cardiovascular risk modification.

Our findings offer a refined, evidence-based perspective for optimizing CPAP use in OSA-related hypertension, aligning with Frontiers in Medicine’s focus on translational clinical research.

All co-authors have approved the manuscript and contributed meaningfully to this work. This manuscript is original, has not been published previously, and is not under consideration elsewhere.This manuscript is original, has not been published previously, and is not under consideration elsewhere. All authors have approved the final version and declare no conflicts of interest. The study protocol was registered on PROSPERO (CRD420251239607).

We sincerely appreciate the time and expertise of the editors and reviewers and look forward to your evaluation.

Sincerely,

Donghui Yue

College of Traditional Chinese Medicine,

Changchun University of Chinese Medicine,

Changchun, China

Email: yuedonghui7776@outlook.com

Phone: +86 18804310249
